# Supplementary material for: Breast cancer quantitative proteome and proteogenomic landscape
Source: Nat Commun. 2019 Apr 8;10:1600. doi: 10.1038/s41467-019-09018-y (PMC6453966; doi:10.1038/s41467-019-09018-y)
Supplement: Supplementary file 1 — Supplementary Information [file 41467_2019_9018_MOESM1_ESM.pdf]

Supplementary Information

Breast cancer quantitative proteome and proteogenomic landscape.

Johansson *et al*., 2019 Nature Communications.

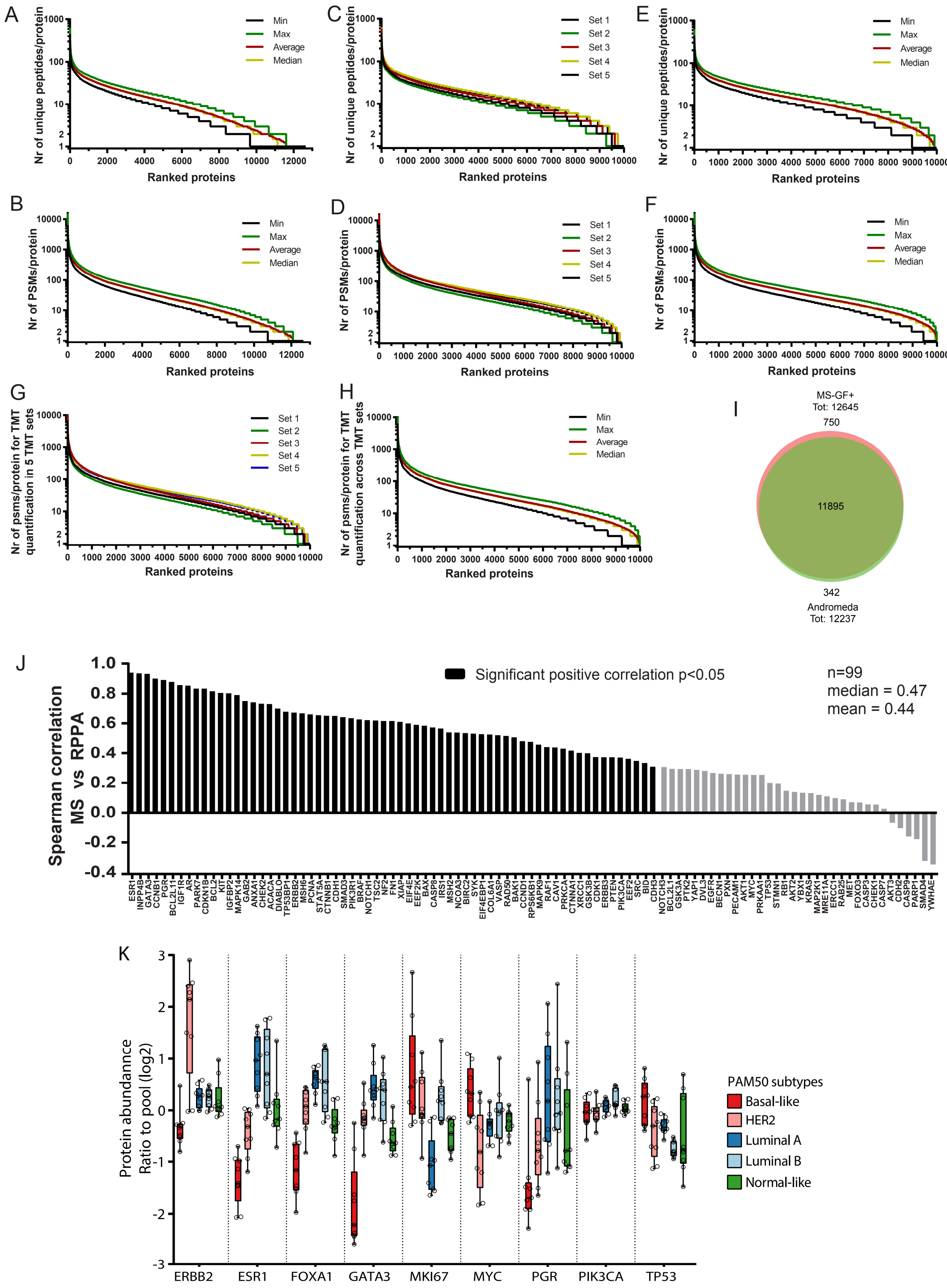

Supplementary Figure 1. Robustness evaluation of MS proteomics data, related to figure 1. MS proteomics data visualized by (**A**) identified unique peptides per protein, and (**B**) peptide spectrum matches (PSM) per protein across all five TMT10 sets (1 % protein FDR). (**C-H**) MS

proteomics data visualized for 9995 proteins with TMT quantification across all 45 tumors (dataset used for multi-level omics analysis). (**C**) Nr of unique peptides per protein, (**D**) PSMs per protein for identifications in each TMT set, and (**E**) Unique peptides and (**F**) PSMs for identification across all five TMT sets. (**G**) PSMs per protein for quantification of the 9995 proteins (gene symbols) in each TMT set and (**H**) across all 5 TMT10 sets. For protein quantification in the 9995 protein set used for analysis, 98% of all protein quantitative values have 2 or more PSMs used for ratio calculation. (**I**) Comparison of protein identifications (based on gene symbols) between Andromeda in Maxquant (MQ) and our search pipeline with MS-GF+. Comparisons are done with proteins below 1% protein FDR. (**J**) Correlation (Spearman) between mass spectrometry (MS) based proteomics quantification and antibody based reverse phase protein array (RPPA) quantification. Some of the lower correlations can be attributed to: (i) differences in epitopes recognised by antibody versus peptides detected by MS, (ii) to members of gene families where the antibody is isoform nonselective (i.e. AKT, GSK3, CDH), (iii) to proteins known to be cleaved (i.e. PARP1, CASP3, CASP8 with a cleavage selective antibody used), (iv) as well as to differences due to quantification performed in neighboring pieces of the tumor. (**K**) Boxplots of MS based quantification grouped by PAM50 subtype for BC histopathological PAM50 surrogate markers and genes of interest in BC.

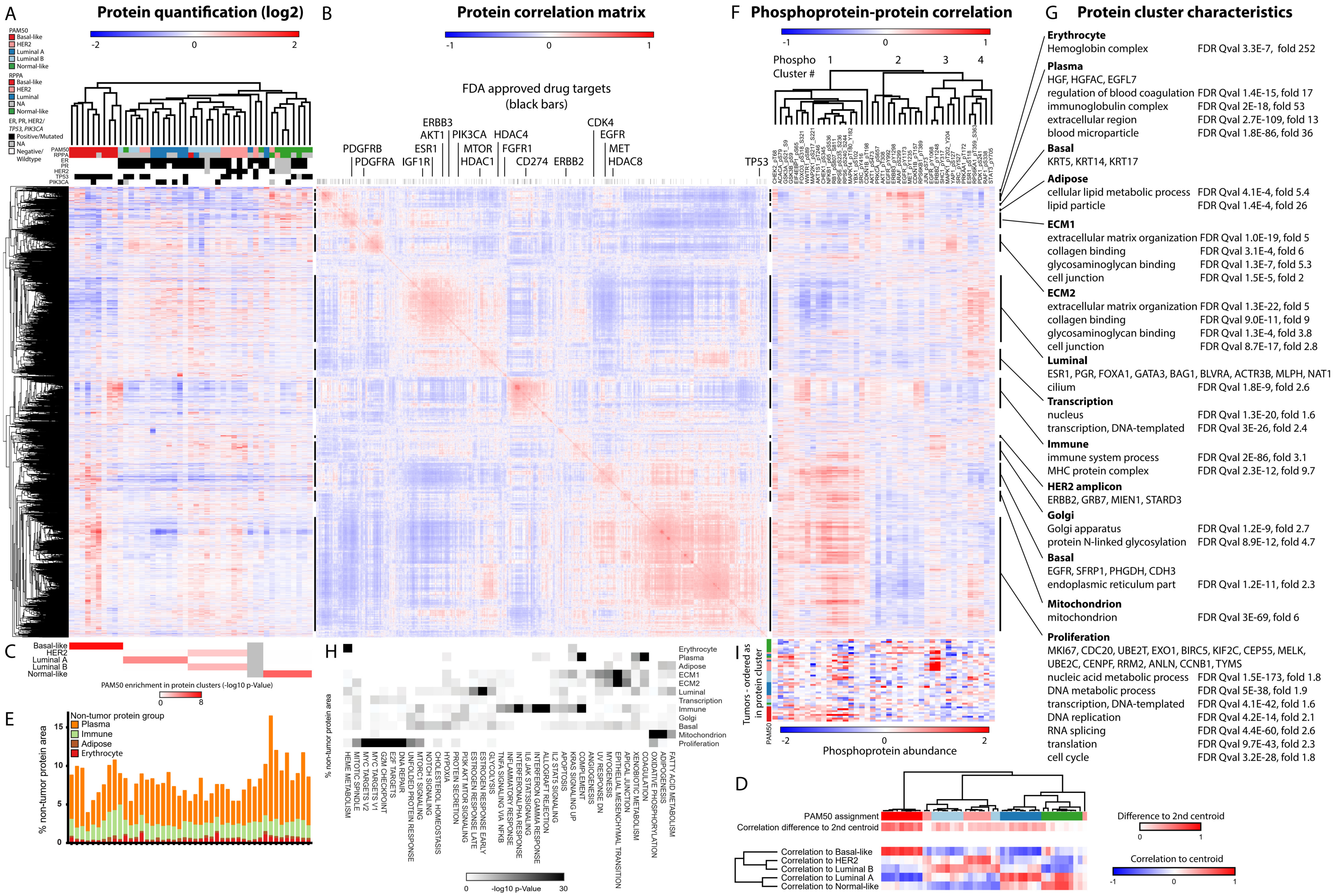
A
Protein quantification (log2)
-2
0
2
PAM50
Basal-like
HER2
Luminal A
Luminal B
Normal-like
RPPA
Basal-like
HER2
Luminal
NA
Normal-like
ER, PR, HER2/
TP53, PIK3CA
Positive/Mutated
NA
Negative/
Wildtype
PAM50
RPPA
ER
PR
HER2
TP53
PIK3CA
B
Protein correlation matrix
-1
0
1
FDA approved drug targets
(black bars)
PDGFRB
PDGFRA
ERBB3
AKT1
ESR1
IGF1R
PIK3CA
MTOR
HDAC1
HDAC4
FGFR1
CD274
ERBB2
CDK4
EGFR
MET
HDAC8
TP53
F
Phosphoprotein-protein correlation
-1
0
1
Phospho
Cluster #
1
2
3
4
G
Protein cluster characteristics
Erythrocyte
Hemoglobin complex FDR Qval 3.3E-7, fold 252
Plasma
HGF, HGFAC, EGFL7
regulation of blood coagulation FDR Qval 1.4E-15, fold 17
immunoglobulin complex FDR Qval 2E-18, fold 53
extracellular region FDR Qval 2.7E-109, fold 13
blood microparticle FDR Qval 1.8E-86, fold 36
Basal
KRT5, KRT14, KRT17
Adipose
cellular lipid metabolic process FDR Qval 4.1E-4, fold 5.4
lipid particle FDR Qval 1.4E-4, fold 26
ECM1
extracellular matrix organization FDR Qval 1.0E-19, fold 5
collagen binding FDR Qval 3.1E-4, fold 6
glycosaminoglycan binding FDR Qval 1.3E-7, fold 5.3
cell junction FDR Qval 1.5E-5, fold 2
ECM2
extracellular matrix organization FDR Qval 1.3E-22, fold 5
collagen binding FDR Qval 9.0E-11, fold 9
glycosaminoglycan binding FDR Qval 1.3E-4, fold 3.8
cell junction FDR Qval 8.7E-17, fold 2.8
Luminal
ESR1, PGR, FOXA1, GATA3, BAG1, BLVRA, ACTR3B, MLPH, NAT1
cilium FDR Qval 1.8E-9, fold 2.6
Transcription
nucleus FDR Qval 1.3E-20, fold 1.6
transcription, DNA-templated FDR Qval 3E-26, fold 2.4
Immune
immune system process FDR Qval 2E-86, fold 3.1
MHC protein complex FDR Qval 2.3E-12, fold 9.7
HER2 amplicon
ERBB2, GRB7, MIEN1, STARD3
Golgi
Golgi apparatus FDR Qval 1.2E-9, fold 2.7
protein N-linked glycosylation FDR Qval 8.9E-12, fold 4.7
Basal
EGFR, SFRP1, PHGDH, CDH3
endoplasmic reticulum part FDR Qval 1.2E-11, fold 2.3
Mitochondrion
mitochondrion FDR Qval 3E-69, fold 6
Proliferation
MKI67, CDC20, UBE2T, EXO1, BIRC5, KIF2C, CEP55, MELK,
UBE2C, CENPF, RRM2, ANLN, CCNB1, TYMS
nucleic acid metabolic process FDR Qval 1.5E-173, fold 1.8
DNA metabolic process FDR Qval 5E-38, fold 1.9
transcription, DNA-templated FDR Qval 4.1E-42, fold 1.6
DNA replication FDR Qval 4.2E-14, fold 2.1
RNA splicing FDR Qval 4.4E-60, fold 2.6
translation FDR Qval 9.7E-43, fold 2.3
cell cycle FDR Qval 3.2E-28, fold 1.8
C
Basal-like
HER2
Luminal A
Luminal B
Normal-like
PAM50 enrichment in protein clusters (-log10 p-Value)
0
8
E
Non-tumor protein group
Plasma
Immune
Adipose
Erythrocyte
% non-tumor protein area
15
10
5
0
H
% non-tumor protein area
Erythrocyte
Plasma
Adipose
ECM1
ECM2
Luminal
Transcription
Immune
Golgi
Basal
Mitochondrion
Proliferation
HEME METABOLISM
MITOTIC SPINDLE
MYC TARGETS V2
MYC TARGETS V1
G2M CHECKPOINT
E2F TARGETS
DNA REPAIR
UNFOLDED PROTEIN RESPONSE
MTORC1 SIGNALING
NOTCH SIGNALING
CHOLESTEROL HOMEOSTASIS
HYPOXIA
PROTEIN SECRETION
PI3K AKT MTOR SIGNALING
ESTROGEN RESPONSE LATE
ESTROGEN RESPONSE EARLY
GLYCOLYSIS
TNFA SIGNALING VIA NFKB
INFLAMMATORY RESPONSE
INTERFERONALPHA RESPONSE
IL6 JAK STAT3SIGNALING
INTERFERON GAMMA RESPONSE
ALLOGRAFT REJECTION
IL2 STAT5 SIGNALING
APOPTOSIS
KRAS SIGNALING UP
COMPLEMENT
ANGIOGENESIS
UV RESPONSE DN
MYOGENESIS
EPITHELIAL MESENCHYMAL TRANSITION
APICAL JUNCTION
XENOBIOTIC METABOLISM
COAGULATION
OXIDATIVE PHOSPHORYLATION
ADIPOGENESIS
FATTY ACID METABOLISM
0
-log10 p-Value
30
I
Tumors - ordered as
in protein cluster
PAM50
-2
0
2
Phosphoprotein abundance
D
PAM50 assignment
Correlation difference to 2nd centroid
Correlation to Basal-like
Correlation to HER2
Correlation to Luminal B
Correlation to Luminal A
Correlation to Normal-like
Difference to 2nd centroid
0
1
Correlation to centroid
-1
0
1

Supplementary Figure 2. Proteome clustering, relation to RPPA and histopathology, related to figure 2. (**A**) Hierarchical clustering (Pearson correlation, average linkage) of proteins from 9995 gene symbols and the 45 tumors.(**B**) Protein correlation matrix showing correlation across 45 tumors with retained protein order as in (A). FDA approved drugs are indicated as black bars on top, highlighting targets of particular interest in breast cancer. (**C**) PAM50 enrichment in protein clusters. Horizontal lines (colored red) of enrichment represent aligned tumors and clusters from (A) above. (**D**) Correlation of mRNA to PAM50 centroids for PAM50 assignments. (**E**) Non-tumor protein area percentage for each tumor, in relation to protein clusters aligned vertically from (A) via (C) above. (**F**) Visualization of correlation for RPPA phosphoproteins (n=41) levels to all MS protein quantifications. Proteins in the heatmap are ordered in the same way as in (A) and RPPA phosphoproteins are clustered. (**G**) Protein cluster characteristics by GO enrichment and genes included in PAM50 panel are named together with selected genes discussed in following data analysis. (**H**) Enrichment of MSigDB hallmarks in the protein clusters from (D). (**I**) RPPA phosphoprotein quantitative values. Ordered as in dendrogram in (F).

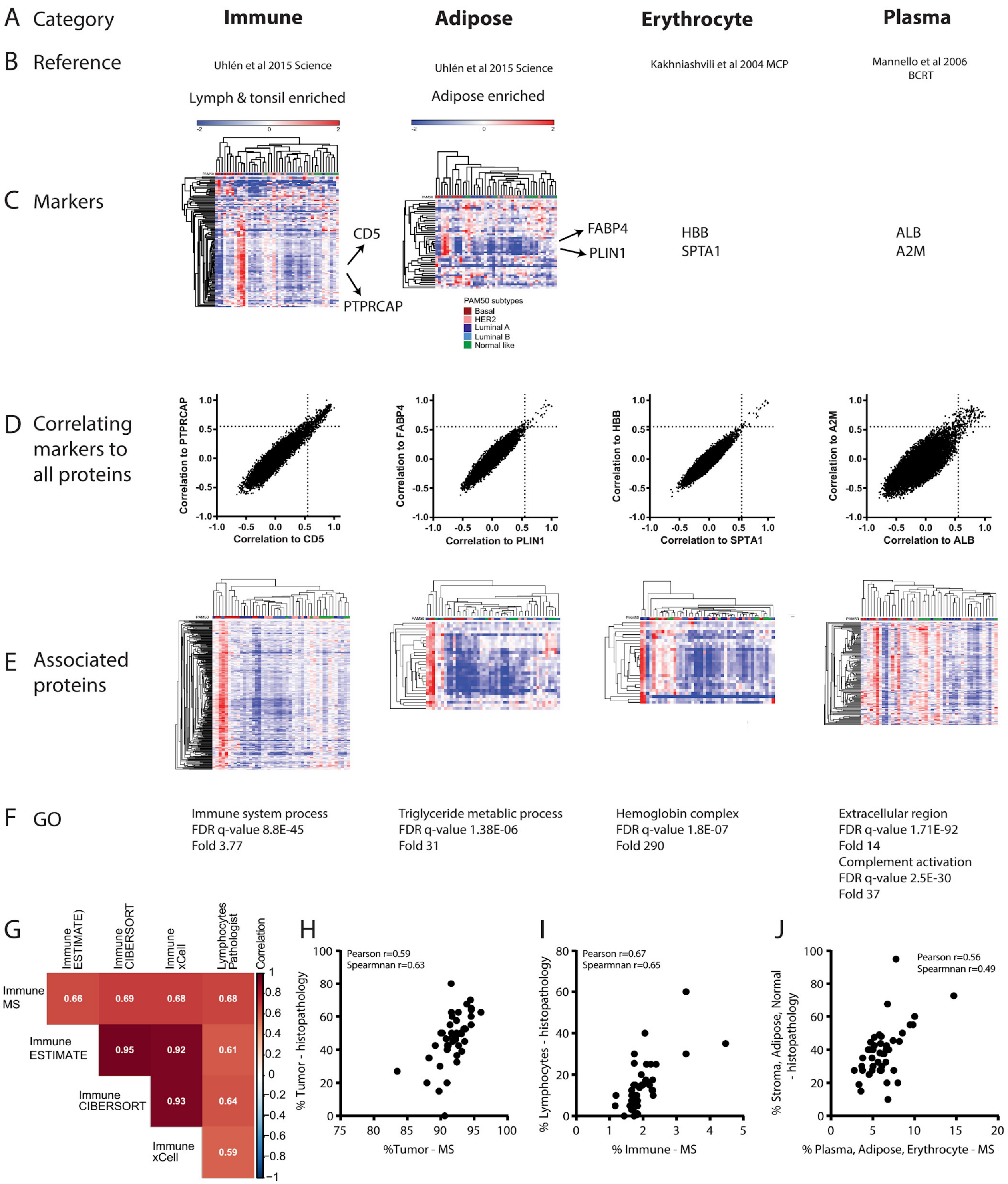

Supplementary Figure 3. Defining non-tumor proteins and association to histopathology, related to figure 2.

(**A**) The 4 categories considered non-tumor. (**B**) Reference for selecting proteins considered enriched or markers for a category. (**C**) Markers selected based on enrichment or literature. (**D**) Scatter plot showing correlation of marker proteins from C to all proteins. Proteins are considered to belong to a non-tumor category if they display >0.55 pearson correlation to both

markers. (**E**) Heatmaps of proteins in each category defined in D. Immune (n=295), adipose (n=30) erythrocyte (n=28), plasma (n=137). (**F**) GO enrichment (GOrilla) for the categories compared to the whole list of proteins. (**G**) Correlation between immune genes defined above to two enrichment tools, ESTIMATE and xCell, and a deconvolution algorithm, CIBERSORT, and histopathology. (**H-J**) Comparison between MS based quantification and histopathology based analysis of tumor components. MS defined tumor cell % is estimated based on proteins defined as tumor only above, and tumor % from histopathology (DCIS and invasive carcinoma). (**H**). Tumor cell content. (**I**) Lymphocyte content. (**J**) Comparison between other non-tumor cell components (stroma, adipose, normal cells plasma, and erythrocyte) quantification based on histopathology and MS.

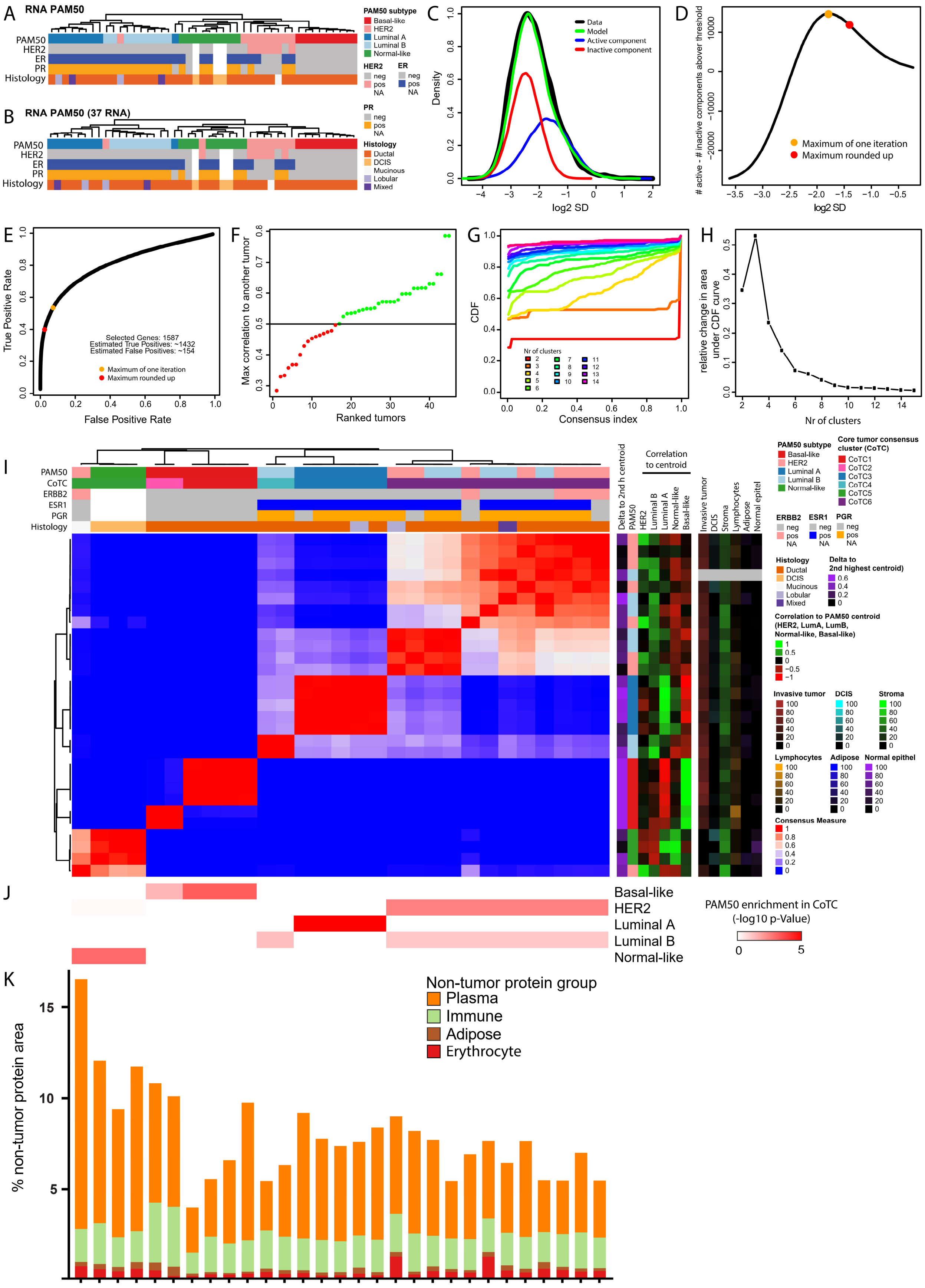
A RNA PAM50
B RNA PAM50 (37 RNA)
PAM50
HER2
ER
PR
Histology
PAM50 subtype
Basal-like
HER2
Luminal A
Luminal B
Normal-like
neg
pos
NA
Ductal
DCIS
Mucinous
Lobular
Mixed
C
Data
Model
Active component
Inactive component
Density
log2 SD
D
active - # inactive components above threshold
Maximum of one iteration
Maximum rounded up
E
True Positive Rate
False Positive Rate
Selected Genes: 1587
Estimated True Positives: ~1432
Estimated False Positives: ~154
F
Max correlation to another tumor
Ranked tumors
G
CDF
Consensus index
Nr of clusters
H
relative change in area under CDF curve
Nr of clusters
I
CoTC
ERBB2
ESR1
PGR
Delta to 2nd h centroid
Correlation to centroid
Invasive tumor
Stroma
Lymphocytes
Adipose
Normal epitel
Core tumor consensus cluster (CoTC)
CoTC1
CoTC2
CoTC3
CoTC4
CoTC5
CoTC6
Delta to 2nd highest centroid)
Correlation to PAM50 centroid (HER2, LumA, LumB, Normal-like, Basal-like)
Normal epithel
Consensus Measure
J
PAM50 enrichment in CoTC (-log10 p-Value)
K
Non-tumor protein group
Plasma
Immune
Adipose
Erythrocyte
% non-tumor protein area

Supplementary Figure 4. PAM50 gene clustering and defining proteome based core tumor clusters (CoTC), related to figure 2. (**A**) RNA clustering of the PAM50 genes (n=50). (**B**) RNA clustering of the same genes as for protein in Fig. 2B (n=37). Clustering using Pearson, average linkage. (**C**). Selection of high variance proteins based gaussian mixture model detect a high and a low component distribution of standard deviations (SD) from the proteome data. (**D**) Identification of a SD threshold using the maximum number of proteins from the high component minus the number of proteins from the low component, above a certain SD threshold. Data represent one iteration from the Gaussian mixture model and is rounded up due to slight variations in the model to obtain a stable threshold. (**E**) ROC representation (average of the iterations) of the threshold generated in (D). (**F**) Selection of core tumors for consensus clustering by including only tumors with Pearson correlation above 0.5 to at least one other tumor. (**G**) Consensus matrix, and (**H**) delta plot for cumulative distribution function (CDF) to assess number of tumor clusters based on CDF difference between different number of clusters. Six core tumor clusters (CoTC) were chosen due to the large delta CDF to 6 clusters followed by leveling off. (**I**) Consensus clustering with 6 clusters, based on SD selected proteins (C-E), without “non-tumor” proteins, and tumors with >0.5 Pearson correlation (F). (**J**) PAM50 enrichment in protein clusters. Horizontal lines (colored red) of enrichment represent aligned tumors and clusters from (I) above. (**K**) Non-tumor protein area percentage for each tumor, in relation to protein clusters aligned vertically from (I,J) above.

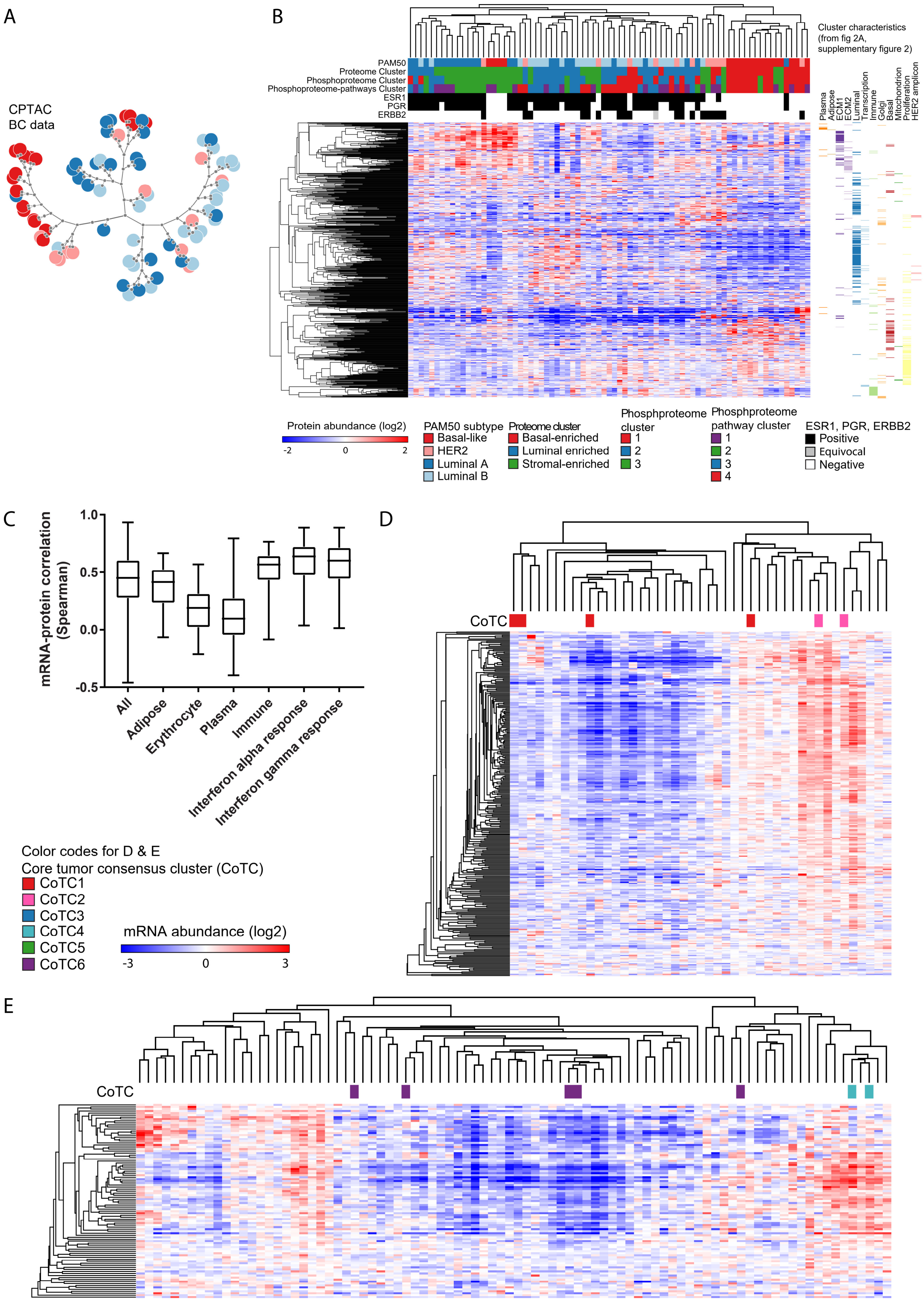

A
CPTAC
BC data
B
Cluster characteristics
(from fig 2A,
supplementary figure 2)
PAM50
Proteome Cluster
Phosphoproteome Cluster
Phosphoproteome-pathways Cluster
ESR1
PGR
ERBB2
Plasma
Adipose
ECM1
ECM2
Luminal
Transcription
Immune
Golgi
Basal
Mitochondrion
Proliferation
HER2 amplicon
Protein abundance (log2)
-2
0
2
PAM50 subtype
Basal-like
HER2
Luminal A
Luminal B
Proteome cluster
Basal-enriched
Luminal enriched
Stromal-enriched
Phosphproteome cluster
1
2
3
Phosphproteome pathway cluster
1
2
3
4
ESR1, PGR, ERBB2
Positive
Equivocal
Negative
C
mRNA-protein correlation (Spearman)
1.0
0.5
0.0
-0.5
All
Adipose
Erythrocyte
Plasma
Immune
Interferon alpha response
Interferon gamma response
D
CoTC
Color codes for D & E
Core tumor consensus cluster (CoTC)
CoTC1
CoTC2
CoTC3
CoTC4
CoTC5
CoTC6
mRNA abundance (log2)
-3
0
3
E
CoTC

Supplementary Figure 5. Clustering of CPTAC BC MS data and presence of CoTC2 and CoTC4 like tumors in Oslo2 cohort, related to figure 2. (**A**) Clustering of CPTAC BC MS data of 77 tumors using overlapping proteins with quantification (632 of 1334) used for consensus clustering in Supplementary Fig. 4I and 2C. Colors according to PAM50 subtypes. (**B**) Clustering as in A with heatmap, and indication of Proteome cluster, Phosphoproteome and Phosphoproteome pathway cluster assignments from Mertins et al. (**C**) mRNA-protein correlation for non-tumor components, adipose, erythrocyte, plasma and immune, defined in Supplementary Fig. 3. Immune and interferon alpha response being characteristic of CoTC2 and CoTC4, respectively (Fig 2A, E, 3D, S2) (**D**) Oslo2 basal-like mRNA expression of immune defined genes from the proteomics data (Supplementary Fig. 3), characteristic of CoTC2 (Fig 2A, E, 3D, Supplementary Fig. 2). (**E**) Oslo2 luminal B mRNA expression for interferon alpha response genes enriched in CoTC4 (Fig 2 E, 3D).

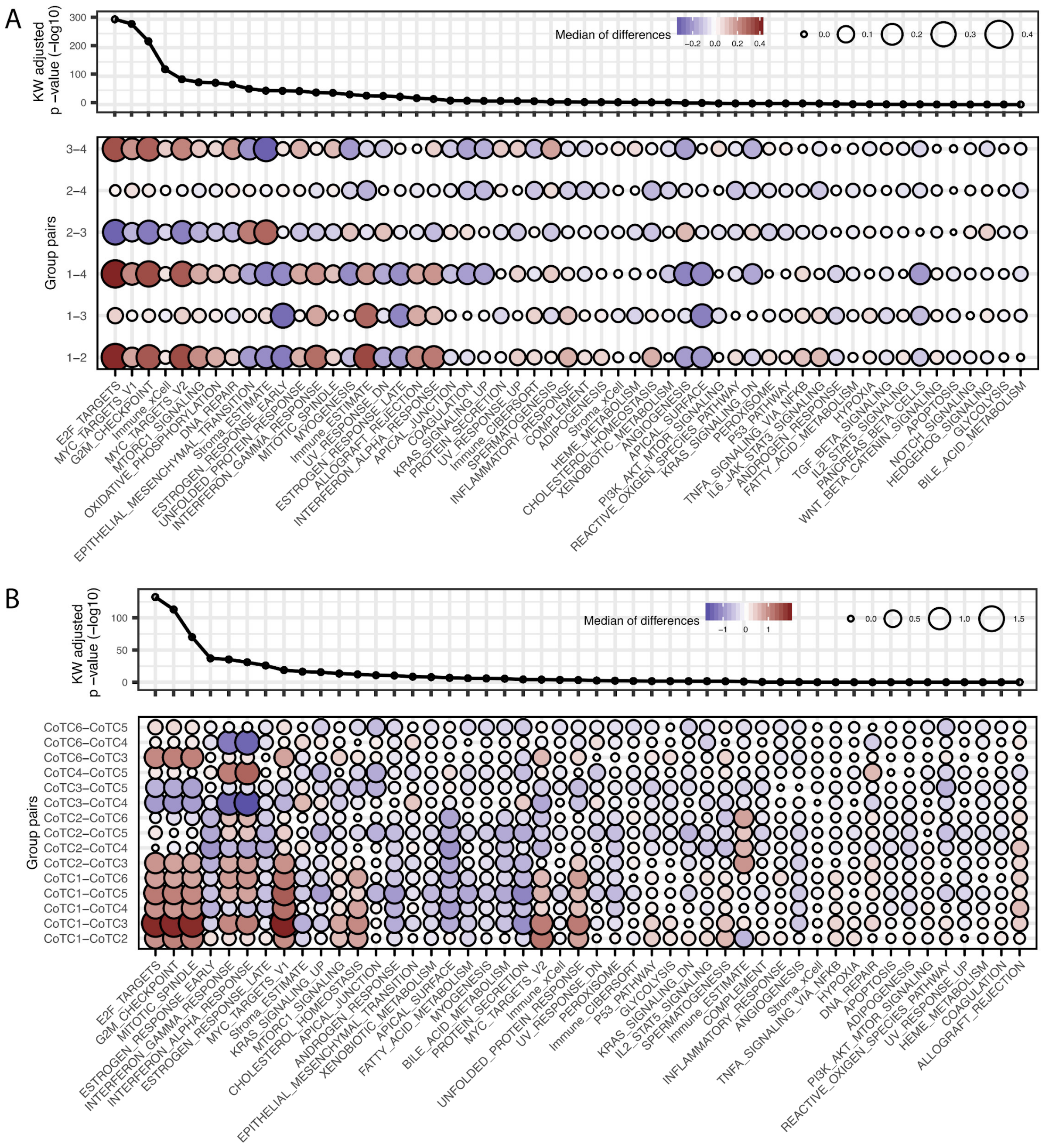

Supplementary Figure 6. Inter-variability between tumor groups, related to figure 2. (**A, B**) Ranking of inter tumor cluster variability based on non-parametric Kruskal-Wallis test (top). Post-hoc Wilcoxon signed-rank was used to assign pairwise significance between clusters (bottom), and used the median of differences as an effect size (color code and circle size). (**A**) Proteome clusters from figure 2A and Supplementary Fig. 2. (**B**) CoTC from figure 2C and Supplementary Fig. 4).

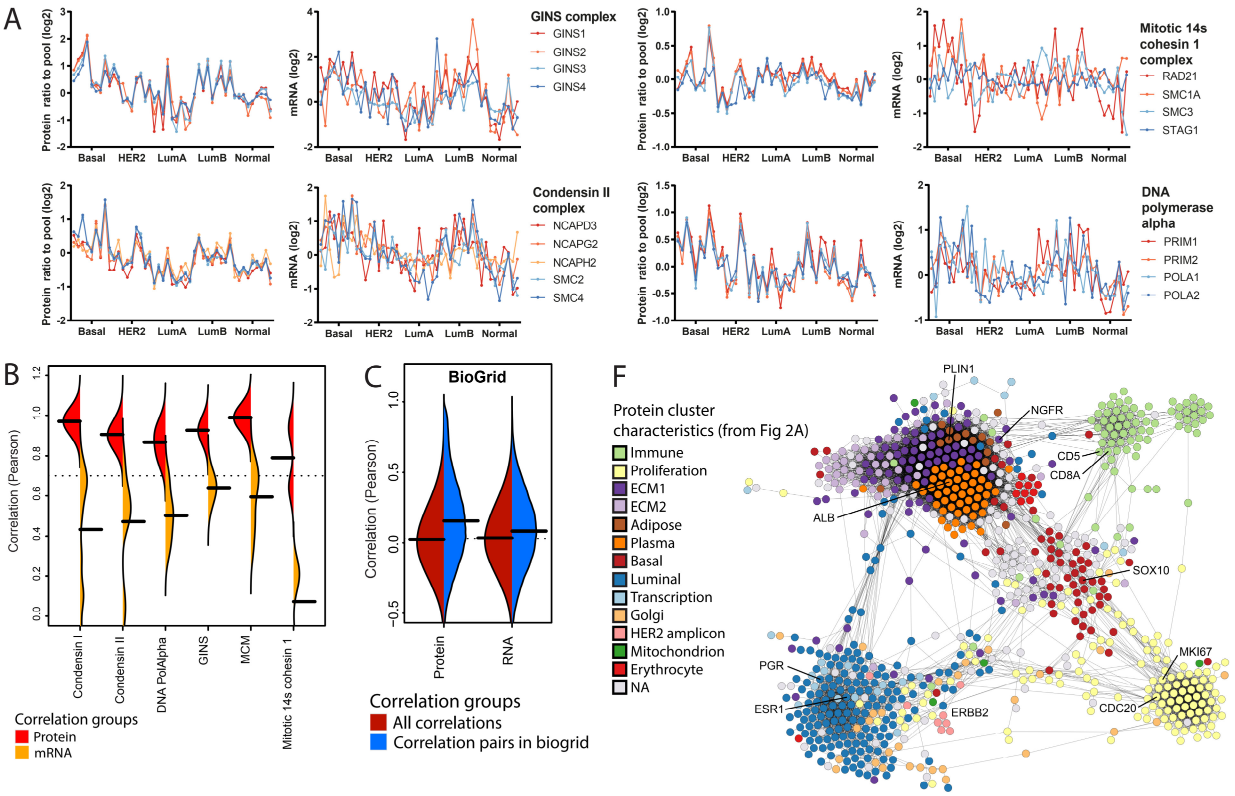

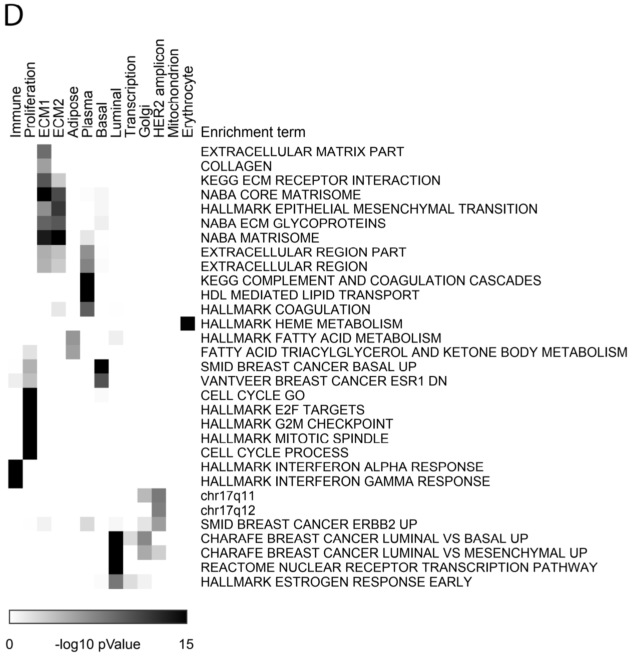

E

Cluster	Description	P-value	FDR q-value	Fold
**Immune**	immune system process	4E-17	3E-13	3
	type I interferon signaling pathway	2E-16	5E-13	13
	interferon-gamma-mediated signaling pathway	4E-10	3E-07	10
	lymphocyte activation	2E-09	9E-07	7
**Proliferation**	mitotic cell cycle process	1E-33	8E-30	5
	cell cycle process	2E-33	7E-30	4
	chromosome organization	8E-26	2E-22	5
	cell division	2E-25	5E-22	5
	cell cycle	1E-14	1E-11	4
	DNA repair	2E-12	9E-10	5
	cytokinesis	7E-10	2E-07	6
**ECM1**	extracellular matrix	9E-14	8E-11	3
	collagen-containing extracellular matrix	6E-13	2E-10	4
	extracellular region	5E-11	1E-08	2
	collagen trimer	3E-09	6E-07	7
	basement membrane	4E-06	4E-04	5
**ECM2**	extracellular matrix	6E-11	6E-08	4
	collagen-containing extracellular matrix	1E-05	4E-03	3
**Adipose**	regulation of fatty acid oxidation	2E-05	9E-02	43
**Plasma**	blood microparticle	1E-35	1E-32	12
	extracellular region	2E-28	1E-25	4
	high-density lipoprotein particle	3E-07	2E-05	11
**Basal**	no enrichment			
**Luminal**	no enrichment			
**Transcription**	no enrichment			
**Golgi**	organelle membrane	1E-06	1E-03	3
	bounding membrane of organelle	2E-05	1E-02	3
	Golgi membrane	2E-04	6E-02	4
**HER2 amplicon**	ERBB2 signaling pathway	2E-04	1E+00	79
	ERBB signaling pathway	1E-03	1E+00	40
**Mitochondrion**	mitochondrial part	7E-08	6E-05	11
	mitochondrial matrix	1E-05	7E-03	14
	mitochondrial membrane part	3E-05	1E-02	41
**Erythrocyte**	hemoglobin complex	1E-06	1E-03	84

Supplementary Figure 7. Protein levels for protein complex members and protein correlation network characteristics, related to figure 3. (**A**) Protein and RNA quantification values for complexes identified during manual inspection of clusters from Fig 2A. Tumors are sorted in the same order in all examples. Basal indicate basal-like and normal indicate normal-like PAM50 subtype. (**B**) Correlation (Pearson) between protein complex members from Fig 2A and (A)**,** for

protein and mRNA. (**C**) Comparison of correlations of quantitative protein and RNA levels across the 45 tumors to correlations between proteins with known protein-protein interactions (based on Biogrid). Protein displayed 0.13 correlation difference between all protein correlations and correlations in Biogrid, while mRNA displayed 0.05 correlation difference. Comparisons are done with the overlapping gene symbols between all three data sets. (**D, E**) Enrichment of proteins from the clusters in fig 2A present in the correlation network using (D) GeneListEnrich shiny tool and (E) Gorilla GO. (**F**) Breast cancer protein correlation network of overlapping (691 of 1445 proteins in Oslo2 correlation network) MS data from CPTAC. Correlation network is constructed using >0.5 Pearson correlation and KCore >1 cutoff. Protein groups are defined and color coded based on GO enrichments in fig 2A and Supplementary Fig. 2.

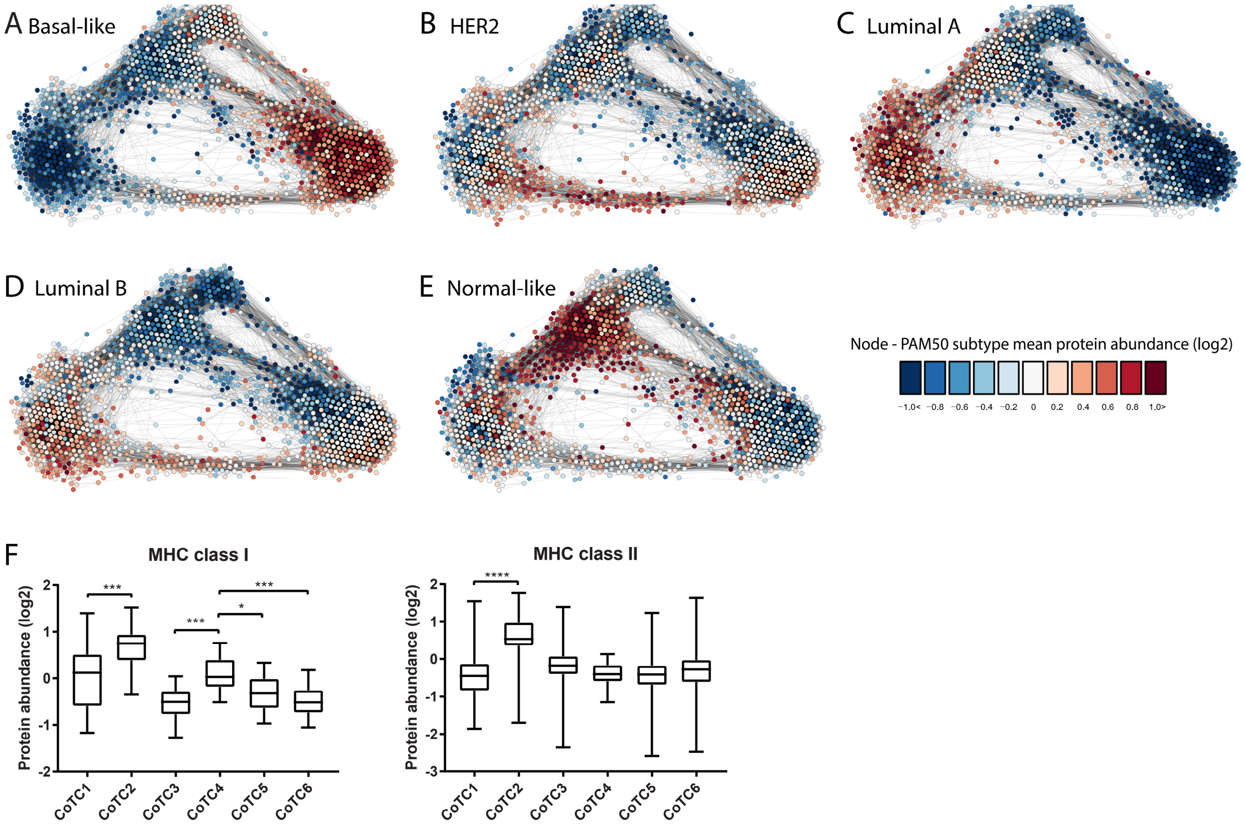

Supplementary Figure 8. Proteome characteristics associate with subtypes, related to figure 3. (**A-E**) Visualization of average protein quantification (node color) for each PAM50 subtype in the protein correlation network. (**F**) Protein levels for MHC class I and II in CoTCs. ANOVA, Bonferroni correction, *p<0.05, ***p<0.001, ****p<0.0001.

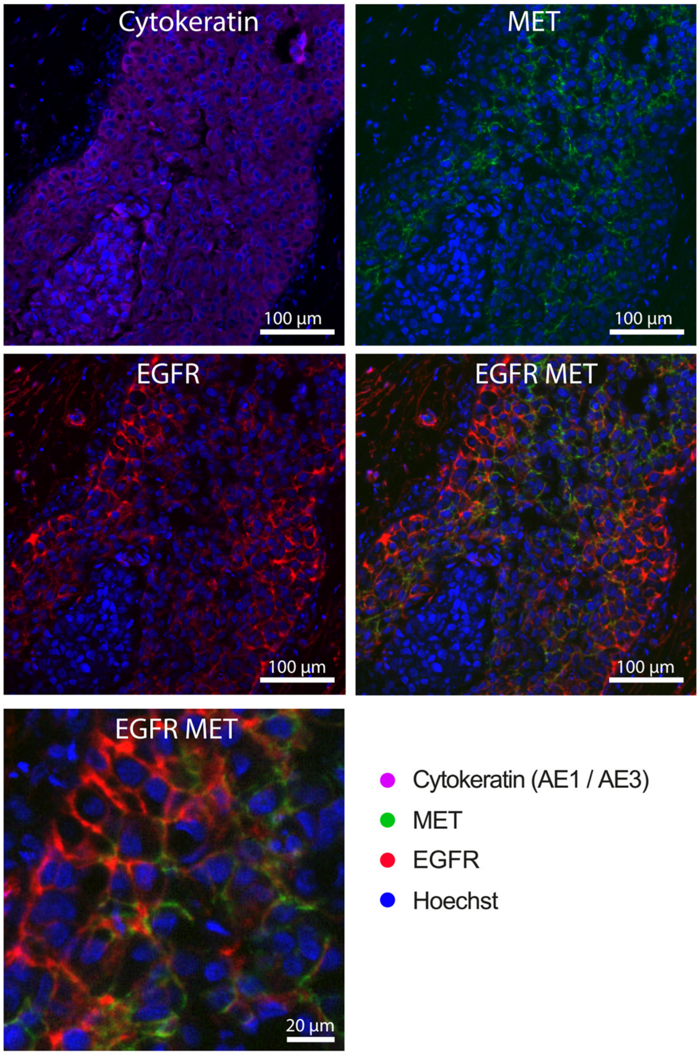

Supplementary Figure 9. Example of immunofluorescence (IF) staining of EGFR and MET in an *in situ* region of a normal-like tumor.

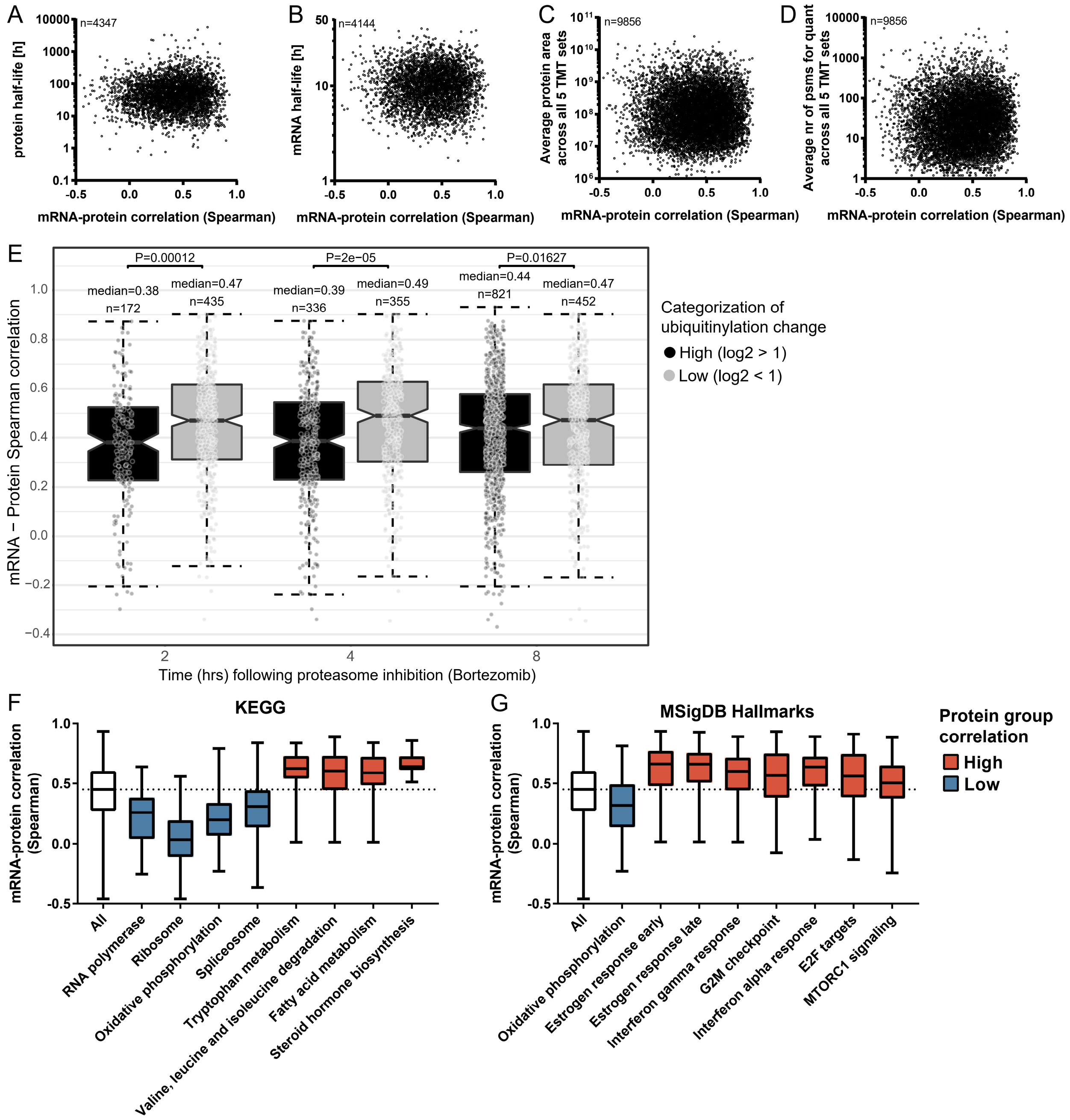

Supplementary Figure 10. RNA-protein correlation analysis, related to figure 5. Scatter plots of (**A**) protein half-life and (**B**) mRNA half-life vs. mRNA-protein correlation. Protein half-life and mRNA-half-life data from Schwanhäusser *et al.*[1]. (**C**) Scatter plots of average protein area across all 5 TMT sets, and (**D**) average number of PSMs for quantification across all 5 TMT sets, vs. mRNA-protein correlation. (**E**) Association of mRNA-protein correlation to ubiquitinylation. mRNA-protein correlations are categorized into low (grey) and high (black) ubiquitinylation based on time series data following proteasomal inhibition by bortezomib. Non-parametric Wilcoxon test was used to compare low and high ubiquitinylation categories. Ubiquitylation data from Kim *et al.*[2]. (**F-G**) Boxplot visualisation of significant results from ranked gene set enrichment analysis of mRNA-protein correlations. (**F**) KEGG pathways and (**G**)

MSigDB Hallmarks. See suppl Supplementary Data 4 for full list and p-values. All protein groups have p-value below 2e-4.

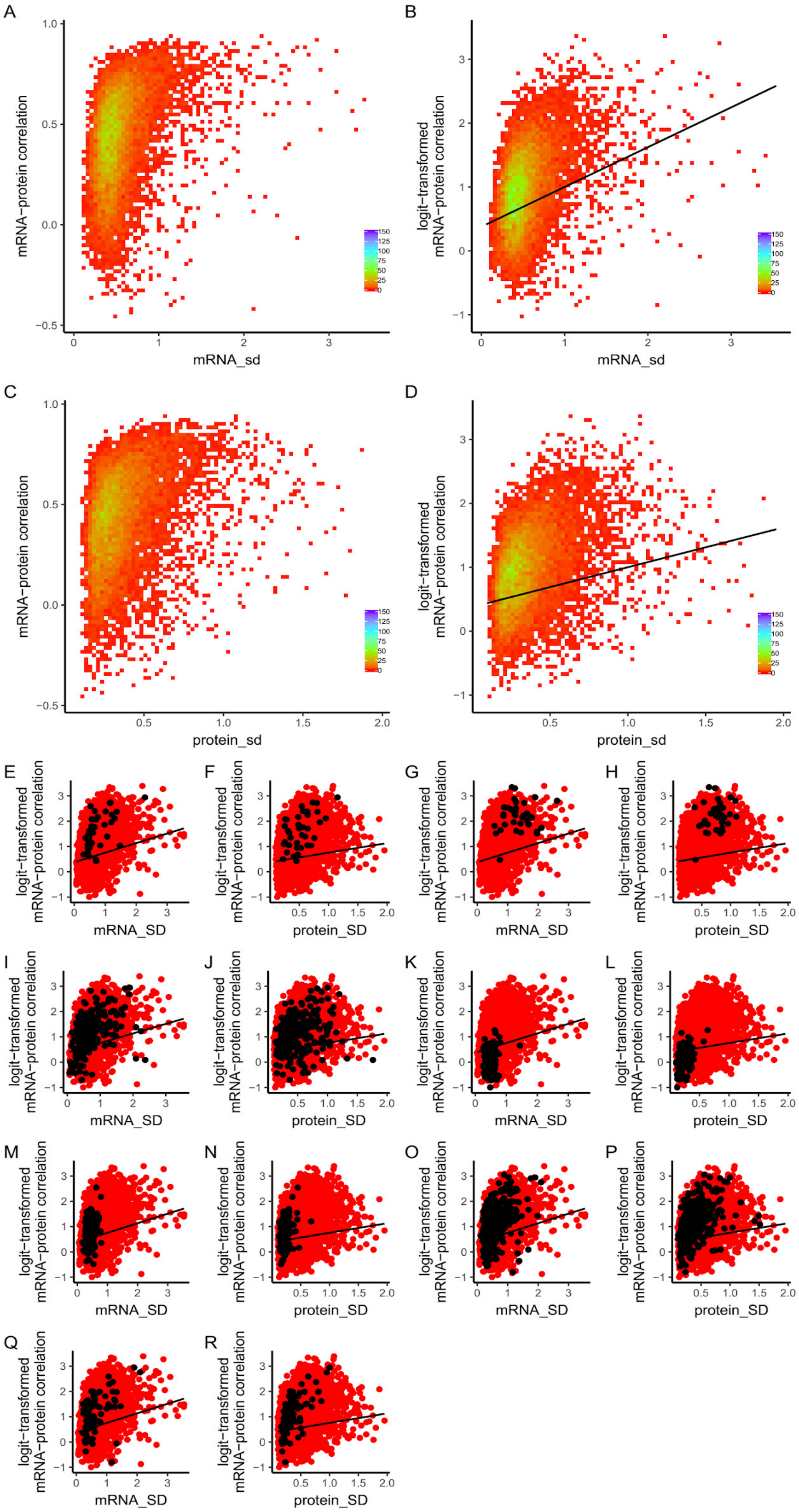

Supplementary Figure 11. RNA-protein correlation and relation to variability, related to figure 5. (**A-D**) Scatterplots of Spearman mRNA-protein correlation as a function of standard deviation

(sd) for mRNA (A-B) and protein (C-D) expression values. The y-axis of A and C shows untransformed Spearman correlation coefficients, while in B and C it shows logit-transformed Spearman correlation coefficients; colors indicate the density of points. The black line in B and D shows the regression line for sdmRNA and sdprotein, respectively, from the bivariate generalized linear model (GLM) modeling Spearman mRNA-protein correlation as a function of both sdmRNA and sdprotein. (**E-R**) Distribution of selected protein groups in scatterplots from B and D. (E-F) Mammaprint (Tian_2010), (G-H) PAM50, (I-J) Protein targets of FDA approved Drug targets, (K-L) Ribosome, (M-N) Spliceosome, (O-P) COSMIC database and Nik-Zainal 93 BC drivers, (Q-R) COSMIC BC drivers and Nik-Zainal 93 BC drivers. Black dots indicate the specified protein group, while red dots represent all other proteins.

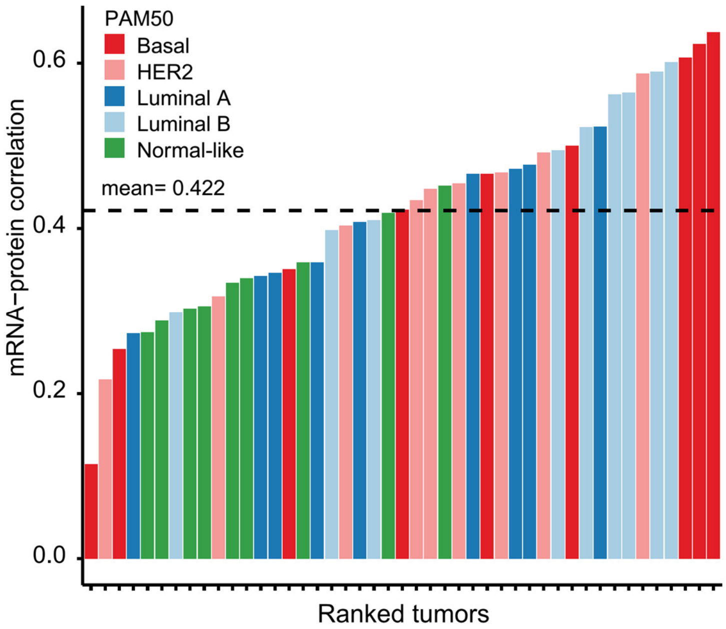

Supplementary Figure 12. Analysis of mRNA-protein correlation (Spearman) for each tumor. Correlations were calculated using all 9856 genes which overlapped between proteome and transcriptome. Colors denote PAM50.

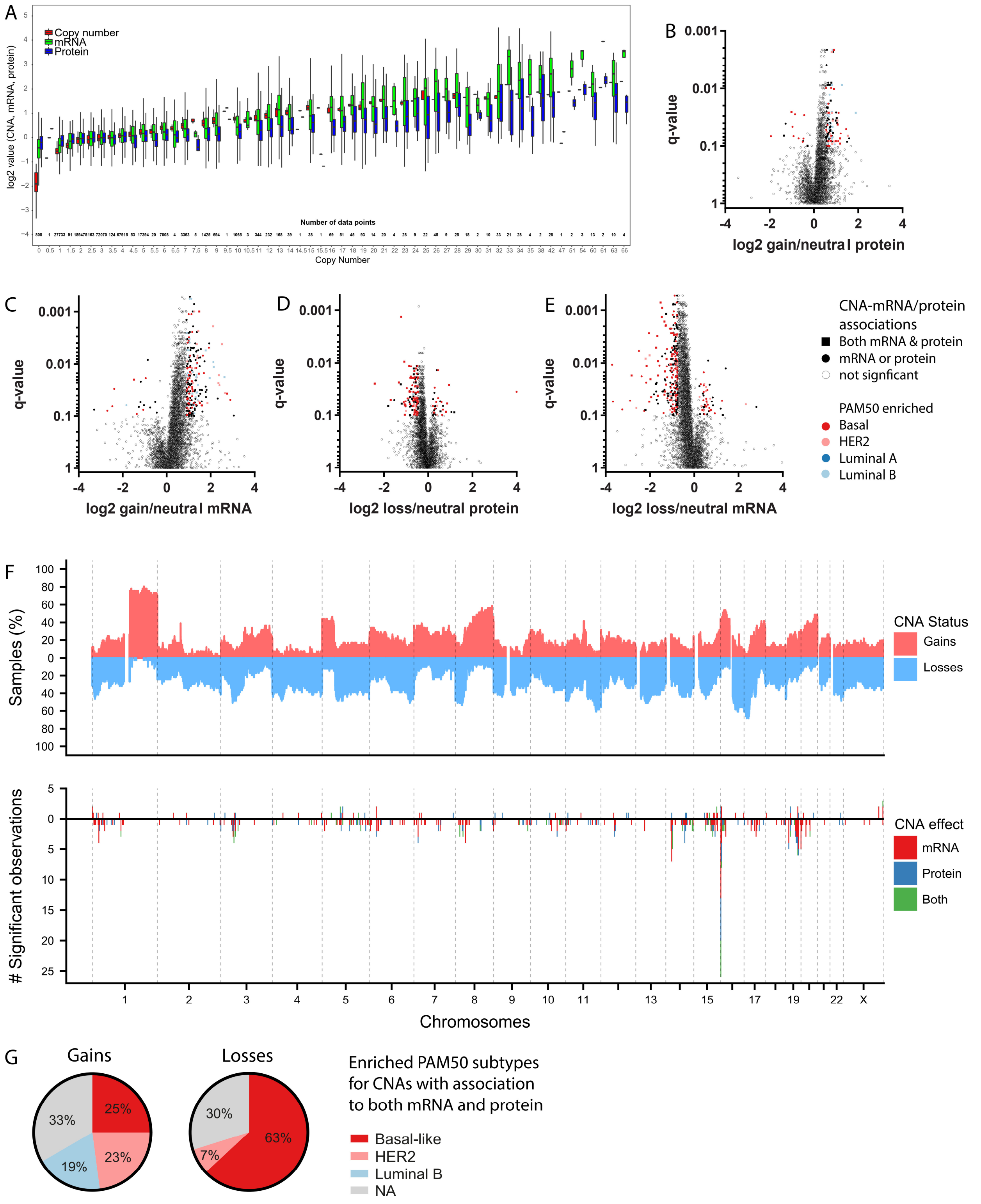

Supplementary Figure 13. Gene copy number effects on mRNA or protein, related to figure 6. (**A**) Distribution of copy numbers, mRNA and protein abundances based on ploidy ranking. Protein and mRNA log2 relative values are displayed for each ASCAT estimated ploidy state for corresponding genes. Copy number distribution is based on logR values. (**B-E**). Permutation

based Wilcoxon rank sum test and fold change (<5 or >95 percentile) were used to define CNA-mRNA and protein associations. Gains (**B, C**) or losses (**D, E**) were compared to neutral events, defined by the ASCAT algorithm, for protein (**B, D**) and RNA (**C, E**). (**F**) Genomic distribution of CNAs and CNA effects of losses defined in (D, E) and visualized in Fig 6B. (**G**) PAM50 subtype enrichment for CNAs with both mRNA and protein associations from fig 6A and B. Supplementary Equation 5.

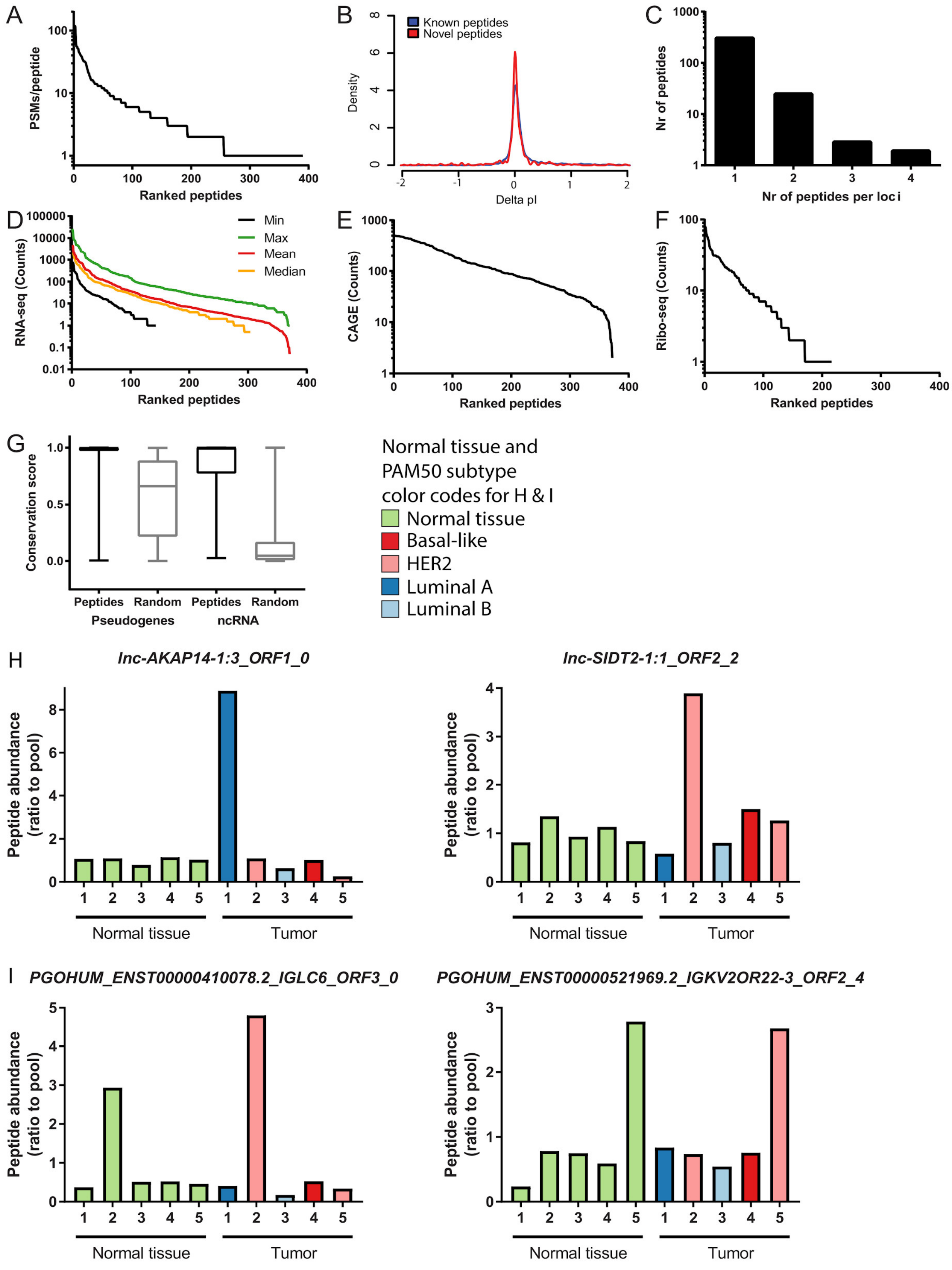

Supplementary Figure 14. Novel peptides validation, and examples in tumors and adjacent normal tissue, related to figure 7. (**A**) Number of PSMs identified per novel peptide. (**B**)

Isoelectric point (pI) distribution for novel peptides (**C**) Number of novel peptides identified per loci (within 10 kb distance). (**D**) RNA-seq support for novel peptides from 20 tumors from Eswaran *et al*.[3]. (**E**) Novel peptides with CAGE reads within 500 bases at the 5' site of the peptide[4]. (**F**) Novel peptides with supporting ribosomal profiling data from tissue/cells from Fritsch *et al*.[5]. (**G**) Conservation score for pseudogene and lncRNA peptides and 1000 randomly selected sites among pseudogenes and lncRNA. (**H**) Examples of lncRNAs with peptide abundance only in tumor. Numbers on x-axis indicate tumor and matched adjacent normal tissue pairs. (**I**) Examples of pseudogenes with peptide abundance in tumor and adjacent normal tissue. Numbers on x-axis indicate tumor and matched adjacent normal tissue pairs.

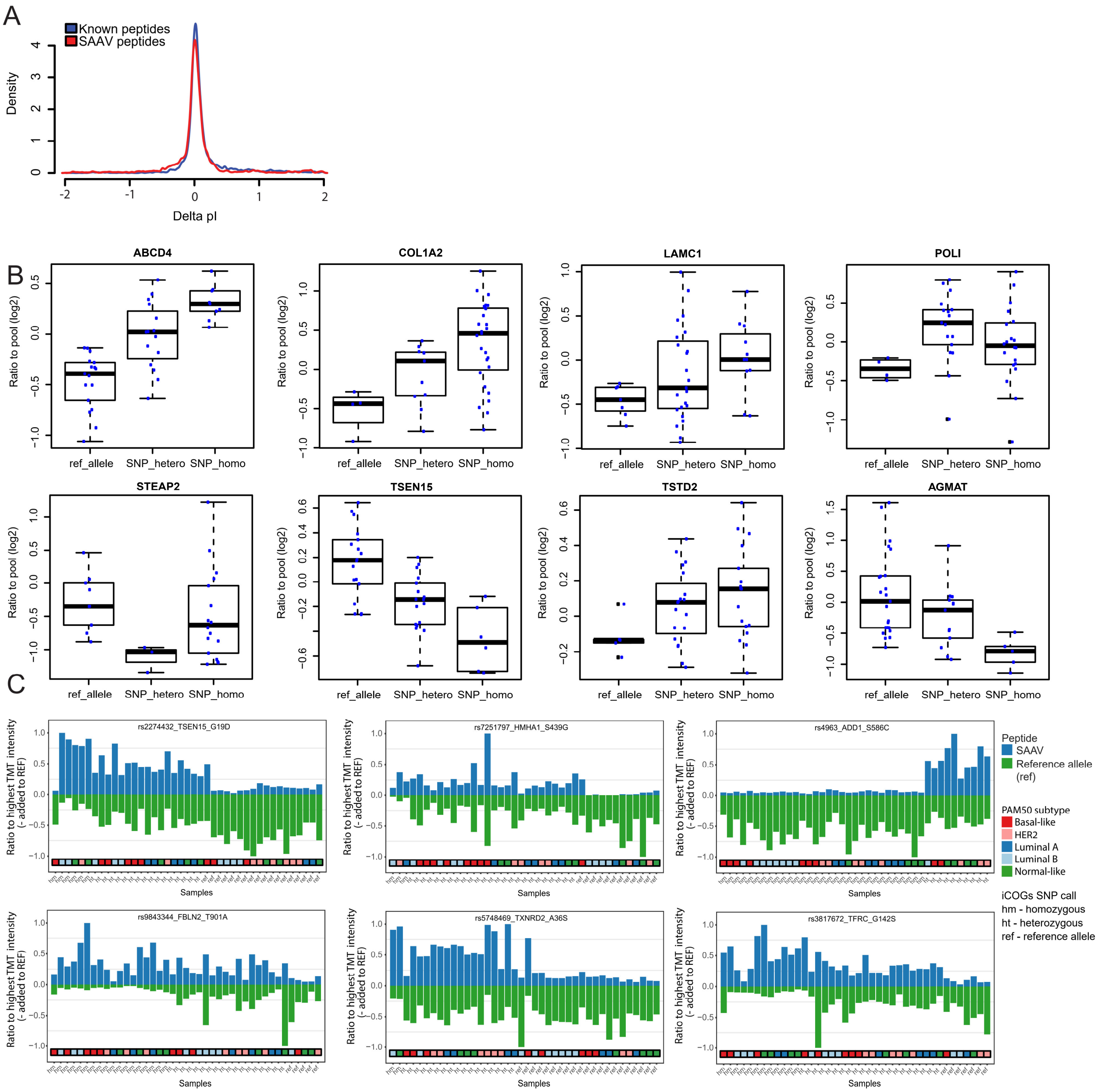

Supplementary Figure 15. SAAV validation, and examples of SNP impact on protein expression and potential allele expression, related to figure 7. (**A**) pI distribution for SAAV peptides. (**B**) Examples of nsSNPs impact on protein expression. SNPs are called from the iCogs. Abbreviations: ref_allele;reference allele, SNP_hetero;heterozygous SNP, SNP_homo;homozygous SNP. (**C**) Examples of potential allele expression, comparing of SAAV peptide (top) expression to its match reference peptide (inverted at the bottom). The ratios to pool were scaled between 0 to 1, by dividing all ratios with maximum ratios observed in 45 tumors. The ratios of reference peptides were multiplied by -1.

Supplementary methods

Tumors and normal samples for proteomics

The tumors from the Oslo2 cohort used in this study have previously been described[6,7]. For MS proteomics analysis, we acquired 9 tumor samples from each of the PAM50 subtypes (basal-like, luminal A, luminal B, HER2, normal-like) that had previously been analyzed for metabolite levels using HR MAS[8].
Pathologists, T.S and Ø.G provided representative tumor tissues, fresh and paraffin embedded, routine pathological macro- and micro work up as well as quality control and assessment with reexamination of all tissue samples.
All patients in this study have given written consent for the use of material and data for research. The study has been approved by the regional committee for medical and health research, REC South East in Norway (approval number 2007.1125, 2016/433).
Tumors and matched normal tissue were from patients operated at Akershus University Hospital, Lørenskog, Norway in 2013 and 2014[9]. The tumor was split in two by the surgeon, one part was put in formalin and used for standard pathological diagnostics while the other part was placed on dry ice. The normal sample was taken at least 2 cm from the tumor and also placed on dry ice. The frozen samples were stored at -80C. All patients gave consent to the use of material for research purposes and the study is approved by the Regional Committees for Medical and Health Research Ethics (REC) for South-eastern Norway.

HiRIEF-nanoLC-MS/MS based proteomics

Sample preparation for mass spectrometry.

Tumor samples, from the Oslo2 cohort were lysed with 4% SDS, 25 mM HEPES, 1 mM DTT. Samples were prepared using a modified version of the spin filter aided sample preparation protocol[10,11]. Lysates were heated to 95°C for 5 min followed by sonication for 1 min and centrifugation at 14,000g for 15 min. The supernatant was mixed with 1 mM DTT, 8 M urea, 25 mM HEPES, pH 7.6 and transferred to a 10-kDa cut-off centrifugation filtering unit (Pall, Nanosep®), and centrifuged at 14,000g for 15 min. Proteins were alkylated by 50 mM iodoacetamide (IAA) in 8 M urea, 25 mM HEPES for 10 min. The proteins were then centrifuged at 14,000g for 15 min followed by 2 more additions and centrifugations with 8 M urea, 25 mM HEPES. Trypsin (Promega) in 250 mM urea, 50 mM HEPES was added to the cell lysate at a ratio of 1:50 trypsin:protein and incubated overnight at 37°C with gentle shaking. The filter units were centrifuged at 14,000g for 15 min followed by another centrifugation with MQ and the flow-through was collected. Peptides were labelled with TMT10plex reagent according to the manufacturer's protocol (Thermo Scientific) and cleaned by a strata-X-C-cartridge (Phenomenex).

IPG-IEF of peptides.

TMT labelled peptides were separated by immobilized pH gradient - isoelectric focusing (IPG-IEF) on pH 3.7-4.9 and 3-10 strips (300 µg peptides per strip) as described by Branca *et al* [10]. Peptides were extracted from the strips by a prototype liquid handling robot, supplied by GE

Healthcare Bio-Sciences AB. A plastic device with 72 wells was put onto each strip and 50 µl of MQ was added to each well. After 30 minutes incubation, the liquid was transferred to a 96 well plate and the extraction was repeated 2 more times. The extracted peptides were dried in speed vac for storage and dissolved in 3% acetonitrile (ACN), 0.1 % formic acid before MS analysis.

Q Exactive analysis.

Before analysis on the Q Exactive (Thermo Fisher Scientific, San Jose, CA, USA), peptides were separated using an Ultimate 3000 RSLCnano system. Samples were trapped on an Acclaim PepMap nanotrap column (C18, 3 µm, 100Å, 75 µm x 20 mm), and separated on an Acclaim PepMap RSLC column (C18, 2 µm, 100Å, 75 µm x 50 cm), (Thermo Scientific). Peptides were separated using a gradient of A (5% DMSO, 0.1% FA) and B (90% ACN, 5% DMSO, 0.1% FA), ranging from 6 % to 37 % B in 30-90 min (depending on IPG-IEF fraction complexity) with a flow of 0.25 µl/min. The Q Exactive was operated in a data dependent manner, selecting top 10 precursors for fragmentation by HCD. The survey scan was performed at 70,000 resolution from 400-1600 m/z, with a max injection time of 100 ms and target of 1 x 10^6 ions. For generation of HCD fragmentation spectra, a max ion injection time of 140 ms and AGC of 1 x 10^5 were used before fragmentation at 30% normalized collision energy, 35,000 resolution. Precursors were isolated with a width of 2 m/z and put on the exclusion list for 70 s. Single and unassigned charge states were rejected from precursor selection. MS data can be downloaded from jPOSTrepo with ID JPST000265 or from ProteomeXchange with ID PXD008841.

Peptide and protein identification.

Orbitrap raw MS/MS files were converted to mzML format using msConvert from the ProteoWizard tool suite. Spectra were then searched using MSGF+ (v10072)[12] and Percolator (v2.08) [13], where search results from 8 subsequent fraction were grouped for Percolator target/decoy analysis. All searches were done against the human protein subset of Ensembl 75 in the Galaxy platform[14]. MSGF+ settings included precursor mass tolerance of 10 ppm, fully-tryptic peptides, maximum peptide length of 50 amino acids and a maximum charge of 6. Fixed modifications were TMT10plex on lysines and peptide N-termini, and carbamidomethylation on cysteine residues, a variable modification was used for oxidation on methionine residues. Quantification of TMT10plex reporter ions was done using OpenMS project's IsobaricAnalyzer (v2.0). PSMs found at 1% FDR (false discovery rate) were used to infer gene identities. Protein false discovery rates were calculated using the picked-FDR method using gene symbols as protein groups and limited to 1% FDR[15].

Protein quantification by TMT10plex reporter ions was calculated using TMT PSM ratios to the tumor sample pool and each tumor were normalized to its median ratio. The median PSM TMT reporter ratio from peptides unique to a gene symbol was used for quantification. Protein ratios to pool of samples is denoted as protein abundance or protein levels in figures and text. For subsequent bioinformatic analysis we used a subset of 9995 gene symbols, representing proteins, with TMT quantification across all sets.

Bioinformatics analysis

Robustness evaluation of MS data

Number of PSMs and unique peptides per protein for identification and quantification were plotted using Graphpad Prism 7.01. We also searched the MS data with Andromeda in Maxquant V1.5.2.8 for comparison to our MSGF+ searches in the Galaxy search pipeline. Similar search settings were used for the Maxquant search, limiting results to 1% protein FDR. Maxquant specific settings included, MS/MS tolerance 20 ppm and acetylation of protein N-terminus. Search results was compared using gene symbols.
For comparison between MS protein quantification and RPPA quantification (Aure et al., 2017), overlapping proteins from 42 tumors were correlated using Spearman´s rho in R. Two tumors were removed from this analysis since they did not pass the RPPA quality control, and one tumor was not present in the RPPA data-set.

Proteome clustering using all and PAM50 genes, and association to phosphoprotein

Clustering and heatmap visualization using all 9995 proteins (gene symbol centric), PAM50 genes, and correlation matrix, phosphoprotein levels and correlations were done using Gene-E (https://software.broadinstitute.org/GENE-E/index.html). Pearson correlation and average linkage was used to cluster tumors and proteins.
To further investigate association of phosphoprotein levels to MS protein based clusters, we correlated the phosphoprotein RPPA quantification to all MS quantified proteins from 42 tumors using Pearson correlation. Correlations were then clustered at the phosphoprotein level (Pearson correlation, average linkage) and ordered in the same way as in the protein clusters for the protein level.

Gene list and ranked gene list enrichment

Protein clusters from clustering using all 9995 proteins were identified using the correlation matrix and cluster characteristics were assigned using GO enrichment by Gorilla (http://cbl-gorilla.cs.technion.ac.il) and enrichment in MsigDB by hypergeometric testing. The fulll list of 9995 proteins were used as background list. MSigDB gene list database was downloaded from the GSEA website (http://software.broadinstitute.org/gsea/downloads.jsp). Genes from CNA-mRNA and -protein associations where screened for enrichment in MSigDB and chromosome positions using the hypergeometric testing implemented in the GeneListEnrich shiny tool (https://github.com/aleferna/BCLandscape_Shiny/tree/master/GeneListCompare). Ranked gene set enrichments for CoTC and PAM50 subtypes was performed using Mann-Whitney tests implemented in the GeneRankEnrich shiny tool (https://github.com/aleferna/BCLandscape_Shiny/tree/master/GeneListCompareRank).

Defining non-tumor proteins, and associating MS quantitative levels to histopathology

To define groups of proteins considered non-tumor, we first identified 2 marker proteins for each non-tumor category. We used transcripts from ProteinAtlas project considered enriched in adipose, and a combined list from lymph & tonsil to define immune markers and selected *FABP4* and *PLIN1*, and *CD5* and *PTPRCAP*, respectively[16]. Markers from literature were

selected for erythrocyte, HBB and SPTA1[17], and for plasma ALB and A2M[18]. We considered proteins belonging to a non-tumor group if they correlated (Pearson) above 0.55 to both markers. Spearman correlation of the marker proteins with their respective transcripts are plotted along with the genome-wide and the immune alpha, gamma response mRNA - protein correlations.
Histopathological evaluation of % of cells and structures was assessed for the 45 tumors by 2 pathologists (Ø.G, T.S). For MS data, protein area was used as a measure of the amount of protein that is present in relation to other proteins. Protein area for each tumor was calculated as the fraction a ratio represented of the summed ratios in one TMT10 plex. Protein areas for each protein group was summed and their percentage calculated from the sum of all proteins in the tumor. Same protein area percentages were used for visualizing non-tumor protein percentage in relation to protein clusters based on all 9995 proteins and CoTC.

Comparison with tools for tissue separation into cell types
We compared the marker-based tissue contamination with two enrichment tools –ESTIMATE and xCell–, and a deconvolution algorithm –CIBERSORT–, all algorithms applicable to microarray data. For ESTIMATE, we used the log2 transformed, quantile normalized transcriptomics data to acquire 'Stroma' and 'Immune' contexture of the 45 tumour samples. For CIBERSORT, we set the unlogged, quantile normalized transcriptomics data as the mixture matrix, and the LM22 signature (signature of 22 immune cell types) as the signature gene list. 'Immune' results were obtained after 1000 permutations under the form of the absolute immune fraction –calculated as the proportion of median expression attributed to all genes in the signature matrix. For xCell, we applied the *xCellAnalysis* function on the log2 transformed, quantile normalized transcriptomics data with default parameters. 'Stroma' and 'Immune' infiltration scores were estimated as the sum of the enrichment scores for the respective cell types as per the original manuscript.
Correlations plots were generated using *corrplot* package and Pearson correlation coefficients were coloured according to their magnitude.

Defining proteome based tumor subtypes - CoTCs

Cluster Detection - overview
In order to identify the strongest subtypes in the protein dataset, we first identified and filtered out unmodulated genes (genes with low standard deviation). From this list we then removed genes that could be associated to adipose, plasma, erythrocytes and immune pathways from the lists defined above, to obtain as pure list of tumor proteins as possible. Next we identified and ignored all outlier samples that could weaken the cluster generation. The remaining samples / genes were then subjected to consensus clustering in order to identify consistent subtypes in the dataset. All code can be found in "Generate_Clustering_and_Network.R" script.

Identification of modulated/unmodulated genes
To define proteins with variation between tumors we calculated a modified "quantile" standard deviation (sdQ) for each protein by ignoring the lowest and highest values of each protein. The distribution of sdQ was modeled as a mixture of gaussian distributions and we used an expectation maximization method (EM) to estimate the different mixture components using the

package mixtools (https://CRAN.R-project.org/package=mixtools). The EM process converged in a 2 distribution solution which we assumed to represent the “Modulated” and “unModulated” proteins. Using this model we estimated the number of “Modulated” and “unModulated” proteins and selected a sdQ threshold which optimized the number of Modulated minus unModulated proteins. As the EM process inevitably produces slightly different thresholds every time it is executed, we performed 10 iterations and rounded the mean of the iterations to the nearest 0.5 in order to have a fully reproducible solution.

Tumor Outlier detection
Given the variation present within a relatively limited number of samples, we selected the samples that were at least similar to a different sample in the datasets. For this we calculated the Pearson correlation matrix between all samples and identified the maximum correlation (outside the diagonal). The 29 samples with Pearson correlation greater than 0.5 to any other tumor were selected and used to identify the protein subtypes.

Clustering - subtype detection
The filtered gene list and 29 tumors were then subjected to consensus clustering as provided by the “ConsensusClusterPlus” R package. This function performs typical hierarchical clustering but does so iteratively, subsampling the genes (80%) and the samples (80%) at each cycle. The result is a measure of how likely 2 samples are found within the same cluster and thus represents a robust clustering that is unlikely to be a product of a few genes. The package generates a report on the cumulative distribution of the consensus index and the Delta area under the curve. This report shows that a cluster number of 6 is optimal as it explains most of the variation and increasing it provides little gains in the Delta area plot.

Clustering of CPTAC BC MS data
BC MS data from Clinical Proteomic Tumor Analysis Consortium (CPTAC) were downloaded (https://cptac-data-portal.georgetown.edu/cptac/s/S015) and filtered to include only proteins with quantification based on unique peptides to a gene and with quantification across all 77 tumors used by Mertins *et al*[19]. Proteins overlapping our list of high variance proteins (632 of 1334), defined by the gaussian mixture model above, were then used to cluster the CPTAC data using Gene-E (Pearson, average linkage).

Confirmation of CoTC2 and CoTC4 in Oslo2 transcriptomics data
To confirm the validity of the CoTCs groups with only 2 members (CoTC2 and 4) we first identified protein groups that are characteristic of the groups. Both CoTC groups are immune enriched, CoTC2 for the whole immune cluster and CoTC4 for interferon alpha response (Fig 2A, F, 3D, Supplementary Fig. 2), and both showed good mRNA-protein correlation. Since CoTC2 is composed of basal-like tumors and CoTC4 of luminal B tumors we selected out these tumor subtypes from the whole Oslo2 cohort. Clustering was done using Gene-E (Pearson, average linkage).

Cluster driver analysis
To quantify the cluster drivers, we compared the inter-subtype/cluster variability of samples via a non-parametric Kruskal-Wallis test. We deployed the 50 hallmark gene sets from the Molecular Signature database (MSigDB, http://software.broadinstitute.org/gsea/msigdb/) as

representatives of core cancer processes, along with the Immune and Stroma component-related proteins from enrichment-based and deconvolution tools used for cell type composition analysis (see relevant part above). For each gene set with at least 5 proteins included in the clustered proteome, we ran a Kruskal-Wallis test, where protein log2 ratios were treated as the continuous dependent variable and clusters/subtypes as the categorical independent variable. P-values were Bonferroni corrected for multiple hypothesis testing. By construction, Kruskal-Wallis test fails to identify groups that are significantly different from the others, despite sufficient variability among them. This is further exacerbated by the large number of observations causing inflated p-values. To account for this, we performed a post-hoc Wilcoxon signed-rank test to assess the significance of pairwise - differences between the groups and used the median of differences as an effect size.

Metabolite analysis

High-resolution magic-angle spinning magnetic resonance spectroscopy (HR MAS MRS) data was previously acquired for the same tumor piece as proteomics was performed on[8]. Processed HR MAS spectral readouts for each metabolite were centered on the median and normalized to the inter-quartile range. Hierarchical clustering applied Ward's linkage method (ward.D2 in R) to pairwise distance measurements calculated as one minus the Pearson correlation. Significant differences between the glycolytic CoTC6 cluster and the CoTC3/4/5 groups were determined were done using a one-tailed Student's t-tests.

Associating protein correlation with protein interactions

Protein core complex information was downloaded from CORUM site (http://mips.helmholtz-muenchen.de/corum/#download) on 30/10/2016. All complex members are assumed to interact with each other. A custom script is used to convert complex information into a pairwise interaction matrix after filtering all genes found in both the RNA and Protein datasets. BioGrid data (BIOGRID-ALL-3.4.137.GenePairs) was downloaded from https://thebiogrid.org/download.php. Data was filtered as to remove any entries containing genes not found in both the RNA and Protein Dataset.

The BioGrid/Corum interaction pairs is then used as a proxy for “known” protein-protein interactions. In the analysis the distribution of the correlation between any 2 different protein’s / mRNA’s was compared to the distribution of pairs known to have protein-protein interactions. Documentation of data processing and figure generation can be found in the PearsonVsBioGrid.R script.

Protein correlation network

Protein data is processed and annotated using the CoreNet_and_Clustering.R script. The protein correlation network is generated by calculating the Pearson correlation matrix for high variance proteins (see identification of modulated genes above) genes and filtering away any protein pair with a Pearson correlation lower than the mean correlation of all pairs + 1.96 standard deviations away (> ~0.5 Pearson) (n=1445). Negative correlations were not used as they confound the visual representation. Data was then exported in GML and imported into Gephi 8.2. Outlier nodes (KCore < 2) were removed and the ForceAtlas algorithm (Scaling 1, Gravity 5, PreventOverlap) was used to layout the network structures. The correlation network figures were generated using the gephi.script file used in the console tool.

To visualize protein levels from CoTC and PAM50 subtypes in the correlation network, the average protein level was calculated for each subtype, and the ratio plotted as a node color. Protein correlation network with CPTAC BC MS data was generated in the same way using overlapping proteins based on gene symbols (691 of 1445).

FDA target analysis

Correlation matrix to visualize correlation between FDA approved drug targets was made using Morpheus (https://software.broadinstitute.org/morpheus/). Pearson and average linkage was used. Gene list with FDA approved drug targets was downloaded from ProteinAtlas (http://www.proteinatlas.org/humanproteome/druggable). BC RNA-seq data for comparison between PAM50 subtypes was downloaded from Genomics Data Commons site (https://gdc.cancer.gov/). RPPA data for Oslo2 were from Aure et al and Haukaas *et al*.[6,8]. To validate MS correlations of FDA targets in in RPPA data 6 proteins were selected based on overlap between MS and RPPA data from Oslo2 and TCGA and visualized using correlation matrices done in Gene-E. Scatter and box plots were done using Graphpad Prism 7.01.

Validation of EGFR and MET in Oslo1 and Oslo2

Immunohistochemical analysis of MET and EGFR was performed on whole sections from Oslo2 and tumor tissue microarrays (TMA) from the Oslo1 cohort. The Oslo1 cohort of primary tumors from early stage breast cancer patients is described in detail by Wiedswang *et al*.[20] and PAM50 classification of the tumors are recently published[21]. Two monoclonal rabbit antibodies (SP44 for MET and D38B1 for EGFR) were chosen and their reaction conditions and specificity were optimized on normal kidney, lung, skeletal muscle, skin and breast tissue sections.
Sections and tumor microarrays were processed for multiplex immunofluorescence, performed with the TSA “denaturing” technique. For every immunohistochemistry running, one positive control and 3 negative control were added, in order to check for specificity of reaction and avoiding over spilling of different fluorescent channels.
Slides were deparaffinized and rehydrated in serial passages of xylene and decreasing ethanol concentrations. The epitope retrieval was performed by heating slides for 20 minutes at 100°C in tris-EDTA pH9 antigen retrieval solution (TBST - 10 mM tris base, 1 mM EDTA, 0.05% Tween 20). After this, slides were washed with deionized water, sections were encircled with a pap pen and then washed with TBST.
The endogenous peroxidase quenching was performed incubating sections with freshly prepared 0.3% hydrogen peroxide in TBST for 30 minutes at room temperature; after, slides were washed in TBST and then incubated with BSA 1% for 30 minutes at room temperature in order to block nonspecific binding of antibodies. Then slides were incubated overnight at 4 °C with the anti-EGFR primary antibody (rabbit monoclonal antibody, clone D38B1, Cell Signaling Technology #4267, lot 11, dilution 1:200, concentration 85 ng/ml, diluted in 1% BSA TBST) or 1% BSA TBST (for the negative control).
After overnight incubation, slides were washed in TBST and incubated with TNB blocking reagent (Perkin Elmer, FP1012) for 30 minutes at room temperature. After this passage, sections were incubated with the polyclonal swine anti-rabbit HRP-conjugated secondary antibody (Dako, cat. P0217, lot. 20020160, dilution 1:200) for 30 minutes at room temperature, then washed with TBST. Slides were incubated with TSA plus cyanine 3.5 (Perkin-Elmer, cat. NEL763B001KT, dilution 1:150) for 15 minutes at room temperature and washed with TBST. In

order to remove the primary-secondary antibody complexes, slides were again boiled for 20 minutes in tris-EDTA pH9 buffer (same conditions as antigen retrieval) and washed with TBST. Incubation with BSA 1% TBST was again performed as previously described, followed by overnight incubation with the anti-MET primary antibody (rabbit monoclonal antibody, clone SP44, Spring Bioscience M3440, lot 160805A, dilution 1:100 final concentration 1,1 µg/ml in 1% BSA TBST) or 1% BSA TBST (for the negative control) at 4°C overnight.
Again, after the overnight incubation slides were again washed with TBST, blocked with TNB blocking agent and incubated with the same anti-rabbit HRP-conjugated secondary antibody as described in the previous section. After washing, slides were incubated with TSA plus fluorescein (Perkin-Elmer, NEL741B001KT, dilution 1:150) for 15 minutes at room temperature. Slides were then washed with TBST and boiled for 20 minutes in the same epitope retrieval buffer in order to detach primary-secondary antibody complexes. After that, sections were rinsed in TBST and incubated with BSA 1% TBST for 30 minutes at room temperature. Then, slides were incubated with the anti-cytokeratin primary antibody solution (mouse monoclonal antibody cocktail, clones AE1 and AE3, Santa Cruz Biotechnology sc-81714, lot B1015, dilution 1:200, concentration 1 µg/ml, diluted in BSA 1% TBST) or BSA 1% TBST (for the negative control) for 1 hour at room temperature. Slides were then washed with TBST and slides incubated with TNB blocking reagent (added with Hoechst 33342, concentration 4 µM). Then, slides were incubated with the peroxidase-conjugated donkey anti-mouse IgG (H+L) (Jackson Immunoresearch, cat. 715-035-150, lot 124592, dilution 1:100, concentration 4 g/ml in TNB blocking agent) for 30 minutes at room temperature. After incubation slides were washed with TBST and incubated with TSA plus cyanine 5 (Perkin-Elmer, cat. NEL745B001KT, dilution 1:100) for 15 minutes at room temperature. After washing with TBST and rinsing with deionized water, slides were mounted with PVA-DABCO mounting agent and let dry for some minutes before observation.
Neighboring sections from the same blocks as used for immunofluorescence were stained with hematoxylin and eosin in order to have a morphological reference of tissues.
Both fluorescence and hematoxylin/eosin were acquired with a slide scanner (Metasystems, Metafer v 3.12.7 mounted on a Zeiss fluorescent microscope Imager.Z2) at 20X magnification for Oslo1 tumor microarrays and 10X for the Oslo2 whole sections, acquiring fluorescent channels in DAPI, FITC, CY 3.5 and Cy 5, and brightfield sections in RGB format. The 8-bit TIFF images produced were employed for the building of virtual slides with the software VSlide (Metasystems, v 1.1.09).
Manual annotation of both Oslo1 and Oslo2 cohort virtual slides were performed by pathologists (F.S, H.G.R, Ø.G) employing the software VSViewer (Metasystems). Using hematoxylin/eosin slides and the corresponding Hoechst and CK channels in the IHC, the epithelial part of every tumor bulk was evaluated for invasive, DCIS, preneoplastic, cystic and normal components, assigning them a percentage. The EGFR staining was particularly useful for recognizing the basal cell layer in DCIS and helping for the differential diagnosis between DCIS and invasive carcinoma. Positivity scoring for MET and EGFR was evaluated on invasive carcinoma, DCIS inside and outside the tumor bulk, preneoplastic, cystic and normal components. The criteria followed for Met and EGFR evaluation in both DCIS and invasive components were:
- 0: not or faint signal could be observed;
- 1+: a weak signal could be detected, usually involving only a part of the cell membrane (not

the entire perimeter)
- 2+: a moderate signal intensity was observed, usually involving the entire membrane in a minority of neoplastic cells (at least 10% of positive cells)
- 3+: moderate/strong signal could be detected, usually involving the entire cell membrane in the majority of cells (at least 50% of positive cells)
- The positivity for EGFR of basal cells in DCIS was not considered in the computation of positivity percentage.

Super-resolution microscopy

The super-resolution microscopy analysis was performed on 2 normal-like neoplasms (OSL.4FA and OSL.441), employing the already described anti-MET monoclonal rabbit antibody (clone SP44) and a mouse monoclonal antibody against the extracellular portion of EGFR (clone EGFR.113).
Briefly, sections were deparaffinized, rehydrated and submitted for epitope retrieval as described in the precedent section. After 30 minutes of protein blocking with BSA 1% TBST, slides were incubated at 4°C overnight with an antibody solution of anti-MET (SP44, same concentration, dilution and batch as before) and anti-EGFR (monoclonal mouse antibody, clone EGFR.113, Leica Biosystems, cat NCL-L-EGFR, lot 6057384, dilution 1:20, concentration 1.3 µg/ml, diluted in 1% BSA TBST); negative control slides were incubated with only one of the 2 antibodies, diluted in 1% BSA TBST.
After overnight incubation, slides were washed in TBST and incubated with a secondary antibody solution containing both anti-rabbit (STAR 635P-conjugated goat anti-rabbit IgG, Abberior, cat. 2-0012-007-2, lot. 05122017Hp, dilution 1:200, concentration 5 µg/ml) and anti-mouse (Alexa Fluor 594-conjugated goat anti-mouse IgG (H+L), Jackson Immunoresearch, cat. 111-585-146, lot. 129501, dilution 1:200, concentration 3.75 µg/ml) fluorescent immunoglobulines for 30 minutes at room temperature. After incubation slides were washed 3 times with TBST, mounted with a PVA-DABCO mounting agent and let dry for some minutes before observation.
Super-resolution STED microscopy was performed on a Leica TCS SP8 STED 3X (Leica Microsystems) equipped with a HC PL APO 100X/1.40 Oil STED WHITE objective. In brief, the fluorophores Alexa Fluor 594 and Abberior Star 635P were excited at 590 and 642 nm, respectively, and STED was performed at 775 nm for both color channels. The channels were recorded line sequentially. All images are raw data. No image processing, except for contrast stretching, was applied.

Correlation of mRNA and protein

We performed Spearman correlation analysis of mRNA and protein expression for matching gene symbols. The analysis was limited to the set of gene symbols (n=9856) for which both mRNA and protein expression was available. The RNA data from the microarrays were log2-transformed, quantile normalized and hospital-adjusted by subtracting from each probe value the mean probe value among samples from the same hospital as described by Aure *et al.*[6]. Firstly, for each gene, we correlated the mRNA and protein expression over all tumors to investigate the distribution of correlation over genes. We compared the distribution of correlation values between different a priori defined protein groups, namely Pred_membrane, GPCR, Ribosomal, Mitochondrial, Cytoskeleton, Transporters, Pred_Secreated, Transcription_factors,

Protein_targets_of_FDA_appr._Drugs, Potential_drug_targets, Spliceosome, Kinome (http://www.proteinatlas.org), PAM50, Oncotype_DX , Mammaprint_(Tian_2010), to that over all genes using a two-sided Wilcoxon ranked-sum test. Next, we used regression analysis to investigate if the observed differences could simply be an effect of gene-specific variation, as measured by the standard deviation (*sd*) in mRNA expression (sd_{RNA}) and protein expression ($sd_{protein}$). As correlation values are bounded in the interval [-1,1], linear regression is inappropriate. Hence, we used a generalized linear model (GLM) with a logit link function and residuals modeled by a binomial distribution (following, e.g., Lesaffre *et al*. 2007[22]). Formally, let y denote the mRNA protein correlation, x denote sd_{RNA} and z denote $sd_{protein}$. Since the logit function is not defined for negative values, the correlation values, y, was rescaled to the interval [0,1] by (1), $y' = (y+1)/2$ and y' was used in the GLM.
We started by investigating the individual univariate regression GLMs for sd_{RNA} and $sd_{protein}$, that is, (2), $logit(y') = \alpha + \beta x + \epsilon$ and (3), $logit(y') = \alpha + \gamma z + \epsilon$, where ϵ denotes the binomially distributed error term (i.e, the residuals). As these indicated relevance of both sd_{RNA} and $sd_{protein}$ for mRNA-protein correlation (Supplementary Fig. 11B,D), the final model became a bivariate GLM including both these variables, that is, (4), $logit(y') = \alpha + \beta x + \gamma z + \epsilon$. The lower values of β and γ, the effect sizes of sd_{RNA} and $sd_{protein}$ (Supplementary Data 4), from the bivariate regression compared to the univariate one reveals some overlap in the signal of the two *sd* measures. However, none goes to zero suggesting that they also provide independent signals.
After fitting this bivariate GLM, we used the residuals of the data (i.e., the difference of the observed $logit(y')$ and those expected from the fitted values of $\alpha+\beta x+\gamma z$) to investigate if specified protein groups displayed significantly different relations of mRNA–protein expression correlation and the two *sd* measures. We compared the distribution of the residuals for the protein group of interest against the residual distribution of all other genes using a one-sided Wilcoxon ranked-sum test for greater or less distributions, as appropriate from the sign of the mean residual value of the protein group.
To find enrichments among low or high mRNA-protein correlations we used ranked-based gene set enrichment, using databases from KEGG pathways or MSigDB.
Secondly, for each tumor, we correlated (Spearman) the mRNA and protein expression for matching genes to investigate the distribution of correlation for individual tumors.
To investigate what could be the cause of the different tumor mRNA-protein correlations, we ranked the tumor mRNA-protein correlations. The ranked tumor mRNA-protein correlations were then correlated (Spearman) to protein abundance using Gene-E. Ranked GSEA analysis of the correlations were performed against GO and MSigDB Hallmark terms using GeneRankEnrich shiny tool (https://github.com/aleferna/BCLandscape_Shiny/tree/master/GeneListCompareRank).

Association of mRNA-protein correlation to ubiquitinylation

Log2 ratios of ubiquitinated sites for bortezomib treated versus untreated HCT116 cells at four time points (0, 2, 4, 8 hours) were downloaded from Kim *et al*.[2], Table S4. In order to acquire gene symbol centric data, sites corresponding to the same protein were merged together and the median value of the log2 ratios was used for the downstream analysis. Overlapping gene symbols with our study were retained. For each of the 2, 4, and 8 hours time points, proteins were categorized as high or low differentially ubiquitinated based on an absolute value of log2

ratio greater than 1, as defined in the original manuscript. A non-parametric two-sided Wilcoxon test was used to estimate the significance of the mRNA-Protein Spearman correlation differences between the groups across the three time points.

CNA analysis

Data processing

LogR and BAF values were extracted from raw CEL-files from Genome-Wide Human SNP Array 6.0 (Affymetrix) using Affymetrix power tools. Segmented copy number values were generated and non-aberrant cell fraction and ploidy was calculated using the Allele-Specific Copy number Analysis of Tumors (ASCAT) package[23]. Segmented copy number data (adjusted for non-aberrant cell admixture and ploidy) were log2-transformed and made "probe-centric" based on the mRNA expression array probe locations. In order to generate gene-centric CNA data, probes that did not correspond to a gene symbol were discarded. In cases where multiple probes corresponded to the same gene, the median of the probes' expression value was assigned as the gene symbol's equivalent. Discrete CNA data -"Loss", "Neutral" or "Gain'- were called based on the absolute CNA values and a threshold above(below) sample-specific ASCAT estimated ploidy +(-) 0.6 as previously defined[23]. Gene symbol-centric protein ratios were log2 transformed. Gene symbol-centric mRNA expression values were derived from mRNA probes using the median value for multiple probes matching to the same gene symbol. Common genes and tumour samples among the three datasets (CNA, mRNA, proteins) were retained for the subsequent analysis. Each of the final datasets consists of 9533 (genes) x 41 (tumour samples, 4 tumours lacked CNA data).

Copy number driven "cis" expression analysis

To identify genes whose mRNA/Protein expression aligned with the CNA profile, mRNA/Protein expression values were assigned to their corresponding CNA status. For each gene, non-parametric Wilcoxon rank-sum test and Log2 fold changes were estimated at two levels of comparison: "Gain vs Neutral" and "Loss vs Neutral" by requesting a minimum of 3 tumor samples per case. Adjusted P values were computed according to the Benjamini–Hochberg approach. Genes with both adjusted P values <= 0.1 and Log2 fold changes below(or above) the 5% (95%) percentile of the Log2 fold change distribution were considered significant.

PAM50 Subtype enrichment

To estimate the enrichment of a PAM50 subtype in a gene's copy number aberration profile, we estimated the probability of observing by chance at least s instances of a subtype among the S tumour samples associated with a CNA "Gain" ("Loss"). Given the total number of tumour samples N, if n of them are associated to the given subtype, then the probability distribution is approximated by the hypergeometric one and can be quantified by the formula:

(5), $P\{X \geq s\} = \sum_{x=s}^{\infty} \binom{n}{x}\binom{N-n}{S-x} / \binom{N}{S}$

For each of the five intrinsic subtypes enrichment, a nominal P-value cutoff of 0.05 was used. The same test was performed to assess the enrichment of the specified subtypes/CoTC clusters in PAM50 subtypes.

IntClust feature set analysis
CNA/mRNA-protein associations were evaluated for the 754 feature set of the IntClust subtype classifier (Curtis et.al, Supplementary Table S42) after converting probes to gene symbols (n=619). Comparisons were restricted to 446 gene symbols that overlapped CNA, mRNA and protein datasets.
Analysis was based on an ANOVA model similar to the one described in Curtis et al . Specifically, the residual mRNA/protein values were used after linear regression on estrogen receptor (ER) status. Unspecified ER status for 3 samples was estimated via logistic regression based on the ER proteomics values. Prior to the model, residual values were converted to ranks. Level of significance was set at a Benjamini-Hochberg adjusted P-value of 0.1.

CNA attenuation analysis - connection to ubiquitination
To investigate how copy number aberrations regulate protein abundance through mRNA expression, we did a protein attenuation analysis similar to Gonçalves *et al.*[24]. For each gene symbol, we devised an attenuation score as the difference between cis CNA – mRNA and CNA – protein Spearman correlation. We used a Gaussian mixture model with 2 mixture components to classify proteins into high and low attenuation groups; proteins with positive attenuation score that belonged to the mixture component with the greater mean were assigned as highly attenuated.
For the new classification, their differential ubiquitination profile across the four time points was done as previously described for the mRNA-protein correlation based ubiquitination plot above.

Proteogenomics

Building pI-restricted databases from six-frame translation (6FT) of human genome
Human genome sequences build 37 (hg19) were downloaded from UCSC genome browser. Nucleotide sequences for each chromosome were in silico translated in six-reading frames (6FT) and in silico digested into peptides following trypsin rules (without missed cleavages, no cleaving on N-terminal side of proline residues). Peptide matches to known proteins were removed and unique peptides with length 8 to 30 amino acids were stored with their chromosome positions. The pI prediction algorithm, PredpI[10] was used to predict isoelectric points of all 6FT theoretical peptides, which were divided into pI-restricted databases with specific pI intervals corresponding to the experimental fractions of IPG strips. Due to both strip manufacturing and strip alignment variations during the process of extraction to 96-well micro-titer plate, the centers of pI intervals may shift slightly run-to-run and were therefore adjusted so that the median value of delta pI (experimental pI minus predicted pI) is equal to 0 for each individual IPG strip (the peptides used to calculate delta pI shift were unique peptides identified with 0% FDR from the standard proteomics search). The pI interval of each pI-restricted database was extended on both sides of the experimental interval with the prediction error margin of ± 0.11, which corresponds to the 95% confidence interval.

Customized peptide database – Human VarDB
Human VarDB contains peptide sequences from pseudogenes, lncRNAs, nsSNPs, somatic mutations and concatenated human known tryptic peptides. Pseudogenes were downloaded from GENCODE release 19 including also consensus pseudogenes predicted by the Yale and

UCSC pipelines. Long non-coding RNAs were downloaded from both GENCODE release 19 and LNCipedia.org v 3.1. These transcripts were translated in three reading frames and digested into peptides by trypsin rule without missed cleavages. Redundant sequences matched to known peptides were discarded. nsSNPs and somatic mutations were downloaded from CanProVar 2.0 and COSMIC release 71. Proteins with substituted amino acid sequences were in silico digested to fully tryptic peptides. The position of substituted amino acid in peptides was noted in order to run SpectrumAI.

Proteogenomics search and Class-specific FDR

MS data from BC landscape cohort and the matched tumor and normal tissue were searched in the same proteogenomics workflow. Peptide spectra were searched in two different databases in parallel: pI restricted 6FT databases and VarDB. Target-decoy separate search strategy was used. Decoy databases were generated from the peptides of corresponding target databases in pseudo-reversed manner (i.e. C-terminal residue is maintained whereas the rest of the target amino acid sequence is reversed). The VarDB search does not require knowledge of peptide pI and therefore can be broadly applied to any shotgun proteomics dataset. A class-specific FDR was estimated separately for novel and single amino acid variant (SAAV) peptides. Novel peptides are defined as sequences specifically from pseudogenes, lncRNA or six-frame translation. All novel peptides were blasted (BLASTP) to Ensembl87+ Uniprot + Refseq+ gencode 24 human proteins to remove known proteins. During revision, the peptides were blasted again against the latest human protein database (Uniprot human proteome 2018 April release + Ensembl92 + GENCODE v28), and recently updated known human proteins are indicated in our novel peptide list. Novel peptides matching to sequences from nsSNPs in CanProVar 2.0 were marked in supplementary Table S6. None of the peptides that could be explained by CanProVar were found in the samples by iCogs SNP array data. SAAV peptides are defined as sequences specifically from CanProVar and COSMIC databases. First, target and decoy matches to known tryptic peptides were discarded (namely deamidations of Asparagine to Aspartic acid and also considering that Isoleucine=Leucine). The FDR of novel (SAAV) peptides was calculated as the number of decoy novel (SAAV) peptides divided by the number of target novel (SAAV) peptides above the score threshold. For quantitative TMT analysis, novel peptide TMT reporter ion ratios were normalized using normalization factors based on median ratio centering calculated from the canonical proteins' table of the standard proteomics search.

SpectrumAI - automated spectra inspection for SAAV peptides with single amino acid substitution

The subset of novel and SAAV peptides with single amino acid substitution identified at 1% class-specific FDR were curated by SpectrumAI, which requires the peptides to fulfill two criteria. First, at least one of the peptide's MS2 spectra must contain ions flanking both sides of the substituted amino acid. For example, if a 12-amino-acid peptide is identified with single amino acid substitution at 8th residue, in order to pass SpectrumAI, it must have matched MS2 peaks (within 10 ppm tolerance) from at least one of the following groups: b7&b8, y4&y5, y4&b7 or y5&b8. Second, the sum intensity of the supporting flanking MS2 ions must be larger than the median intensity of all fragmentation ions. An exception to these criteria is made when the substituted amino acid has a proline residue to its N-terminal side. Because CID/HCD

fragmentation at the C-terminal side of a proline residue is thermodynamically unfavored, SpectrumAI only demands the presence of any b or y ions on the right (C-terminal) side of the substituted position. SpectrumAI is available at:https://github.com/yafeng/SpectrumAI
We observed 3 peptide identifications that mapped to the Y chromosome. Upon closer inspection of these peptides, 2 of them mapped to the X transposed and degenerative regions of the Y chromosome that display high sequence similarity to the X chromosome. One base substitution, for either peptide, on the Y chromosome will produce a single amino acid change and the peptide will map to the X chromosome to a pseudogene and a known gene. The highest scoring peptide of all 3 can be explained as a know nsSNP (rs1726208) in MXRA5 on chromosome X. The third peptide mapped to the Y chromosome displayed 2 amino acid changes in comparison to peptide sequences from 8 known genes (TMEM222, ACTG1, ACTB, POTEM, POTEJ, POTEI, POTEE, POTEF), 4 pseudogenes and one intergenic region. Since synthetic peptide validation confirmed the presence of the assessed novel peptide sequences (File S1), it is likely that acquisition of mutations has generated alternative genomic sites that a peptide can be mapped to. We did not observe any support for SNV changes at the alternative mapping sites from the iCOGs data. However, the iCOGs data is limited to predefined genomic changes.

Orthogonal validation data of proteogenomics searches
We assessed the evolutionary conservation of identified novel coding peptides by calculating mean scores for each region based on the PhastCons 46-way vertebrate multiple alignment tracks available from:
http://hgdownload.cse.ucsc.edu/goldenPath/hg19/phastCons46way/vertebrate
The calculations were done using Perl script and posted at:
https://www.biostars.org/p/16724/#16731. Bigwig tracks of ribosome profiles of THP-1 cell lines published by Fritsch *et al.*[5] and mapped CAGE reads across a panel of biological samples published in Forrest *et al*.[4] were downloaded from UCSC genome browser. RNAseq data was from Eswaran *et al*.[3]. Confirming the presence of our novel peptides in other MS BC data were done using data from Mertins *et al*.[19] by supplementing our novel peptide to the canonical database and searched as described above with VarDB. Synthetic peptides representing novel (n=67) and SAAVs (n=31) were ordered from JPT to confirm peptide sequence identity.

Impact of SAAV on protein levels.
There were 170 nsSNPs detected both in iCOG SNP array data and in MS data (SNP array data was available for 42 of 45 analyzed tumors). In order to study the impact of SNPs on protein expression level, the 42 tumors were grouped based on genotype calling from iCOG array data into three classes: *ref_allele* (both copies of the gene having same nucleotide bases to reference genome hg19), *hetero_SNP* (one of the copy contains the SNP variant), *homo_SNP*(both copies have the SNP variant). We then compared the relative abundance of the 170 proteins (ratio to the pool) among tumors in the three groups. Student t test (two sides) was used to assess if there is significant differential expression between different groups (examples shown in Supplementary Fig. 8-I were filtered by p-values<0.01). Minimum three tumors were required for the test. To do a global analysis of SNP impact shown in Figure 7G, we first calculated an impact score for the 170 SNPs across 42 tumors using same formula described in Zhang *et al*.[25],

(6), Impact score = $(EXP - MEDIAN_{ref\_allele})/MAD_{ref\_allele}$

where EXP is the protein ratio in the sample containing the SNP, and $MEDIAN_{ref\_allele}$ and MAD_{ref_allele} are the median and MAD (median absolute deviation) of the protein ratios in all samples in the ref_allele group. Then the impact scores were divided into the three groups: *ref_allele, hetero_SNP and homo_SNP* to make the cumulative distribution plot (Fig. 7F). Kolmogorov-Smirnov test was used to assess the differences between two distributions. To visualize SNP impact on individual gene expression, we plotted the relative abundance of variant peptide generated from SNP and the wild type peptide in the 42 tumors (Fig. 7G). All values were scaled between 0 to 1 for both the variant and wild type peptide.

NSearch neoantigen candidates in draft proteomes data and MHC binding prediction
We searched the MS data from the draft proteome[26] in a concatenated database including the list of novel peptides identified in our data and the latest known human proteins (Uniprot human proteome 2018 April release + Ensembl92 + GENCODE v28). MHCflurry was used to predict potential high affinity epitopes from our list of novel peptides[27]. Epitopes predicted within top 0.2% percentile binding affinity were considered as high affinity. No HLA typing was performed. The HLA supertype representatives (A01:0, A02:01, A03:01, A24:02, A26:01, B07:02, B08:01, B27:05, B39:01, B40:01, B58:01, B15:01) were used to predict affinity.

Supplementary Table 1. Antibodies and related reagents used.

REAGENT	SOURCE	IDENTIFIER
Anti-EGFR monoclonal antibody	Cell Signaling Technology	clone D38B1, cat #4267, clone RRID: AB_10828841
Anti-EGFR monoclonal antibody	Leica Biosystems	Clone EGFR.113, cat NCL-L-EGFR<br>Clone RRID: AB_563696
Anti-MET monoclonal antibody	Spring Bioscience	clone SP44, cat M3440, RRID:AB_1660951
Anti-cytokeratin monoclonal antibody cocktail	Santa Cruz Biotechnology	Clones AE1 and AE3, cat sc-81714, RRID:AB_2191222
STAR 635P-conjugated goat anti-rabbit secondary IgG	Abberior	cat 2-0012-007-2, RRID: not available
Alexa Fluor 594-conjugated anti-mouse secondary IgG	Jackson Immunoresearch	cat 111-585-146<br>RRID: not available
HRP-conjugated swine anti-rabbit secondary antibody	Dako	Cat P0217, RRID:not available
HRP-conjugated donkey anti-mouse secondary antibody	Jackson Immunoresearch	Cat 715-035-150, RRID:AB_2340770
TSA blocking reagent (10g)	Perkin Elmer	Cat FP1012, RRID: not available
TSA Plus cyanine 3.5 detection kit	Perkin Elmer	Cat NEL763B001KT, RRID:not available
TSA Plus fluorescein detection kit	Perkin Elmer	Cat NEL741B001KT, RRID:not available
TSA Plus cyanine 5 detection kit	Perkin Elmer	Cat NEL745B001KT, RRID:not available

Supplementary Table 2. Softwares and scripts used in the data analysis.

Softwares and scripts	Location
Gene-E	https://software.broadinstitute.org/GENE-E/
Morpheus	https://software.broadinstitute.org/morpheus/
Graphpad Prism 7.01	https://www.graphpad.com/scientific-software/prism/
Association of correlation to protein interactions	https://github.com/aleferna/BCLandscape<br>Script: PearsonVsBioGrid.R script
Correlation network	https://github.com/aleferna/BCLandscape<br>Script: Generate_Clustering_and_Network.R & gephi.script
Consensus clustering	https://github.com/aleferna/BCLandscape<br>Script: Generate_Clustering_and_Network.R
Shiny Web Portal Tools	https://github.com/aleferna/BCLandscape_Shiny
GSEA	http://software.broadinstitute.org/gsea/index.jsp
mRNA-protein correlation	https://doi.org/10.5281/zenodo.1466478
CNA-mRNA/protein	https://github.com/ioasia/Breast-Cancer
Gephi	https://gephi.org/
Proteogenomics analysis	https://github.com/yafeng/proteogenomics_python
SpectrumAI	https://github.com/yafeng/SpectrumAI
Metafer v3.12.7	https://metasystems-international.com/en/products/metafer/
VSlide v1.1.09	https://metasystems-international.com/en/products/solutions/tissue-imaging/
VSViewer	https://metasystems-international.com/en/products/solutions/tissue-imaging/

Supplementary Table 3. Data availability table (* due to patient data protection in Norway)

**Data type**	**Link to data or database**	**Identifier**	**Used in figure(s)**	**Publication**
MS proteomics of 45 breast tumors	http://jpostdb.org	JPST000265 PXD008841	All (except Supplementary Fig. 9)	This paper
MS proteomics tumor and matched normal	http://jpostdb.org	JPST000496 PXD011385	Supplementary Fig. 14	This paper
MS proteomics of breast tumors	https://cptac-data-portal.georgetown.edu/cptac/s/S015	S015	7C, Supplementary Fig. 7F	[19]
MS proteomics of normal tissue	http://proteomecentral.proteomexchange.org	PXD000561	7D	[26]
Synthetic peptides for PSM validation	http://proteomecentral.proteomexchange.org	PXD006291		This paper
RPPA - Oslo2	https://breast-cancer-research.biomedcentral.com/articles/10.1186/s13058-017-0812-y#MOESM1	Additional file 1	Supplementary Fig. 1, 2	[6]
mRNA - Oslo2	https://www.ncbi.nlm.nih.gov/geo/query/acc.cgi?acc=GSE80999	GSE80999	3, 4, 5, 6, Supplementary Fig. 4, 4, 7, 10, 11, 12, 13	[6]
mRNA - TCGA	https://portal.gdc.cancer.gov/projects/TCGA-BRCA	TCGA-BRCA	4D	[28]
CNA	Upon request*		Supplementary Fig. 13,	
iCOGs, SNP	Upon request*		7, Supplementary Fig. 15	
HR-MAS, Metabolite	https://www.ncbi.nlm.nih.gov/pmc/articles/PMC4922058/	Table S2	2	[8]

Supplementary References

1. Schwanhäusser, B. *et al.* Global quantification of mammalian gene expression control. *Nature* **473**, 337–342 (2011).
2. Kim, W. *et al.* Systematic and quantitative assessment of the ubiquitin-modified proteome. *Mol. Cell* **44**, 325–340 (2011).
3. Eswaran, J. *et al.* Transcriptomic landscape of breast cancers through mRNA sequencing. *Sci. Rep.* **2**, 264 (2012).
4. FANTOM Consortium and the RIKEN PMI and CLST (DGT) *et al.* A promoter-level mammalian expression atlas. *Nature* **507**, 462–470 (2014).
5. Fritsch, C. *et al.* Genome-wide search for novel human uORFs and N-terminal protein extensions using ribosomal footprinting. *Genome Res.* **22**, 2208–2218 (2012).
6. Aure, M. R. *et al.* Integrative clustering reveals a novel split in the luminal A subtype of breast cancer with impact on outcome. *Breast Cancer Res.* **19**, 44 (2017).
7. Aure, M. R. *et al.* Integrated analysis reveals microRNA networks coordinately expressed with key proteins in breast cancer. *Genome Med.* **7**, 21 (2015).
8. Haukaas, T. H. *et al.* Metabolic clusters of breast cancer in relation to gene- and protein expression subtypes. *Cancer Metab* **4**, 12 (2016).
9. Quigley, D. A. *et al.* Age, estrogen, and immune response in breast adenocarcinoma and adjacent normal tissue. *Oncoimmunology* **6**, e1356142 (2017).
10. Branca, R. M. M. *et al.* HiRIEF LC-MS enables deep proteome coverage and unbiased proteogenomics. *Nat. Methods* **11**, 59–62 (2014).
11. Manza, L. L., Stamer, S. L., Ham, A.-J. L., Codreanu, S. G. & Liebler, D. C. Sample preparation and digestion for proteomic analyses using spin filters. *Proteomics* **5**, 1742–1745 (2005).
12. Kim, S. & Pevzner, P. A. MS-GF+ makes progress towards a universal database search tool for proteomics. *Nat. Commun.* **5**, 5277 (2014).
13. Granholm, V. *et al.* Fast and accurate database searches with MS-GF+Percolator. *J. Proteome Res.* **13**, 890–897 (2014).
14. Boekel, J. *et al.* Multi-omic data analysis using Galaxy. *Nat. Biotechnol.* **33**, 137–139 (2015).
15. Savitski, M. M., Wilhelm, M., Hahne, H., Kuster, B. & Bantscheff, M. A Scalable Approach for Protein False Discovery Rate Estimation in Large Proteomic Data Sets. *Mol. Cell.*

Proteomics **14**, 2394–2404 (2015).
16. Uhlén, M. *et al.* Proteomics. Tissue-based map of the human proteome. *Science* **347**, 1260419 (2015).
17. Kakhniashvili, D. G., Bulla, L. A., Jr & Goodman, S. R. The human erythrocyte proteome: analysis by ion trap mass spectrometry. *Mol. Cell. Proteomics* **3**, 501–509 (2004).
18. Mannello, F., Tonti, G. A. M. & Papa, S. Human gross cyst breast disease and cystic fluid: bio-molecular, morphological, and clinical studies. *Breast Cancer Res. Treat.* **97**, 115–129 (2005).
19. Mertins, P. *et al.* Proteogenomics connects somatic mutations to signalling in breast cancer. *Nature* **534**, 55–62 (2016).
20. Wiedswang, G. *et al.* Detection of isolated tumor cells in bone marrow is an independent prognostic factor in breast cancer. *J. Clin. Oncol.* **21**, 3469–3478 (2003).
21. Ohnstad, H. O. *et al.* Prognostic value of PAM50 and risk of recurrence score in patients with early-stage breast cancer with long-term follow-up. *Breast Cancer Res.* **19**, 120 (2017).
22. Lesaffre, E., Rizopoulos, D. & Tsonaka, R. The logistic transform for bounded outcome scores. *Biostatistics* **8**, 72–85 (2007).
23. Van Loo, P. *et al.* Allele-specific copy number analysis of tumors. *Proc. Natl. Acad. Sci. U. S. A.* **107**, 16910–16915 (2010).
24. Gonçalves, E. *et al.* Widespread Post-transcriptional Attenuation of Genomic Copy-Number Variation in Cancer. *Cell Syst* **5**, 386–398.e4 (2017).
25. Zhang, B. *et al.* Proteogenomic characterization of human colon and rectal cancer. *Nature* **513**, 382–387 (2014).
26. Kim, M.-S. *et al.* A draft map of the human proteome. *Nature* **509**, 575–581 (2014).
27. O'Donnell, T. J. *et al.* MHCflurry: Open-Source Class I MHC Binding Affinity Prediction. *Cell Syst* **7**, 129–132.e4 (2018).
28. Berger, A. C. *et al.* A Comprehensive Pan-Cancer Molecular Study of Gynecologic and Breast Cancers. *Cancer Cell* **33**, 690–705.e9 (2018).
