# Supplementary material for: Breast cancer quantitative proteome and proteogenomic landscape
Source: Nat Commun. 2019 Apr 8;10:1600. doi: 10.1038/s41467-019-09018-y (PMC6453966; doi:10.1038/s41467-019-09018-y)
Supplement: Supplementary file 2 — Description of Additional Supplementary Files [file 41467_2019_9018_MOESM2_ESM.pdf]

Description of Additional Supplementary Information

File Name: Supplementary Data 1.
Description: Oslo2 45 BC quantitative proteome data, related to figures 1-6.

File Name: Supplementary Data 2.
Description: Correlation matrix of FDA approved drug targets, related to figure 4.

File Name: Supplementary Data 3.
Description: EGFR and MET IF scores, related to figure 4.

File Name: Supplementary Data 4.
Description: mRNA-protein correlation, related to figure 5.

File Name: Supplementary Data 5.
Description: CNA-mRNA/protein association, related to figure 6.

File Name: Supplementary Data 6.
Description: Novel peptide and SAAV identifications, related to figure 7.

File Name: Supplementary Data 7.
Description: Synthetic peptide validation of novel peptides and SAAV, related to figure 7.

File Name: Supplementary Data 8.
Description: Novel peptide identifications in tumor and normal tissue, related to Supplementary Figure 14.
