# Supplementary material for: Breast cancer quantitative proteome and proteogenomic landscape
Source: Nat Commun. 2019 Apr 8;10:1600. doi: 10.1038/s41467-019-09018-y (PMC6453966; doi:10.1038/s41467-019-09018-y)

CBR4_S54C

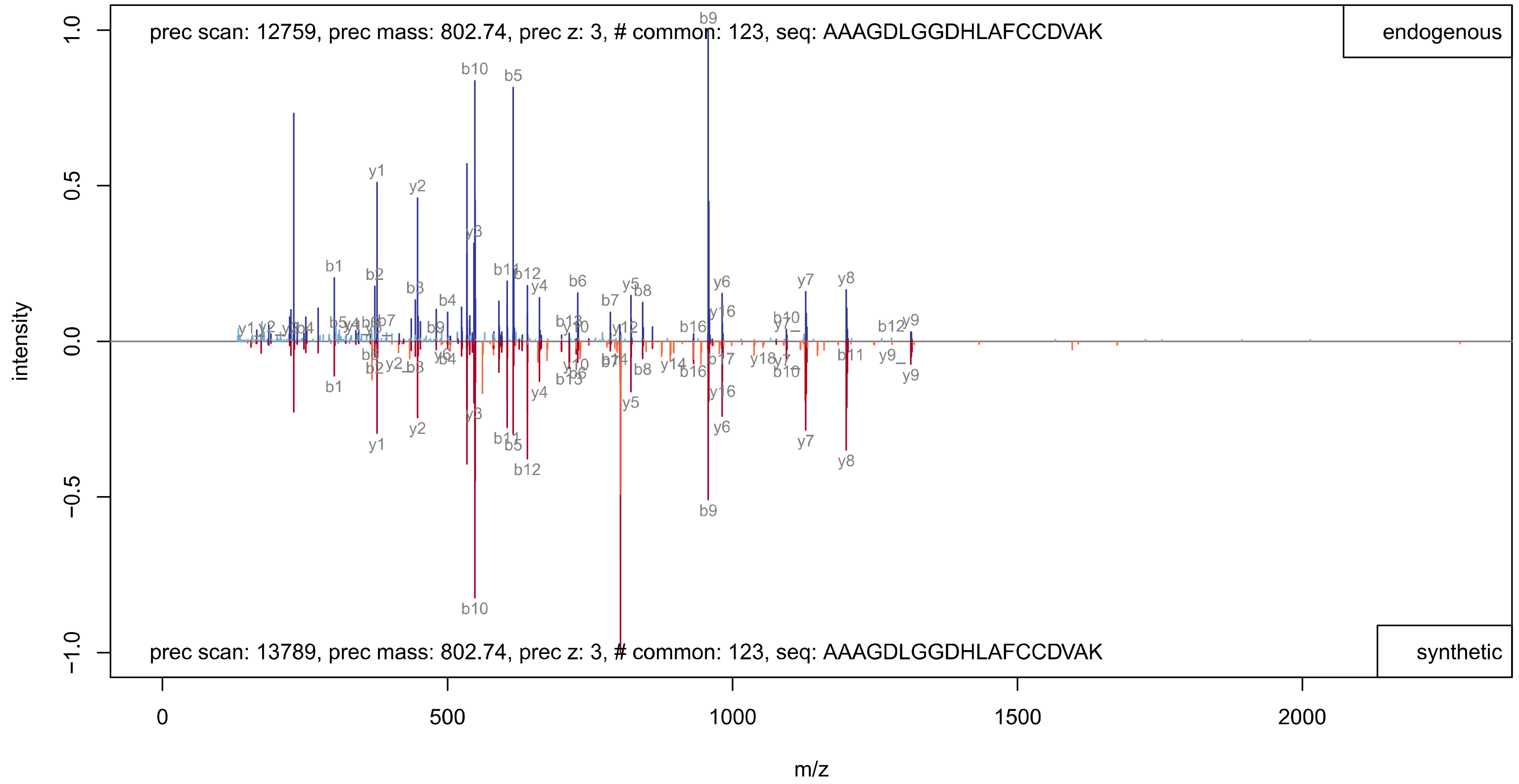

HNRNPA2B1.new.Nterm

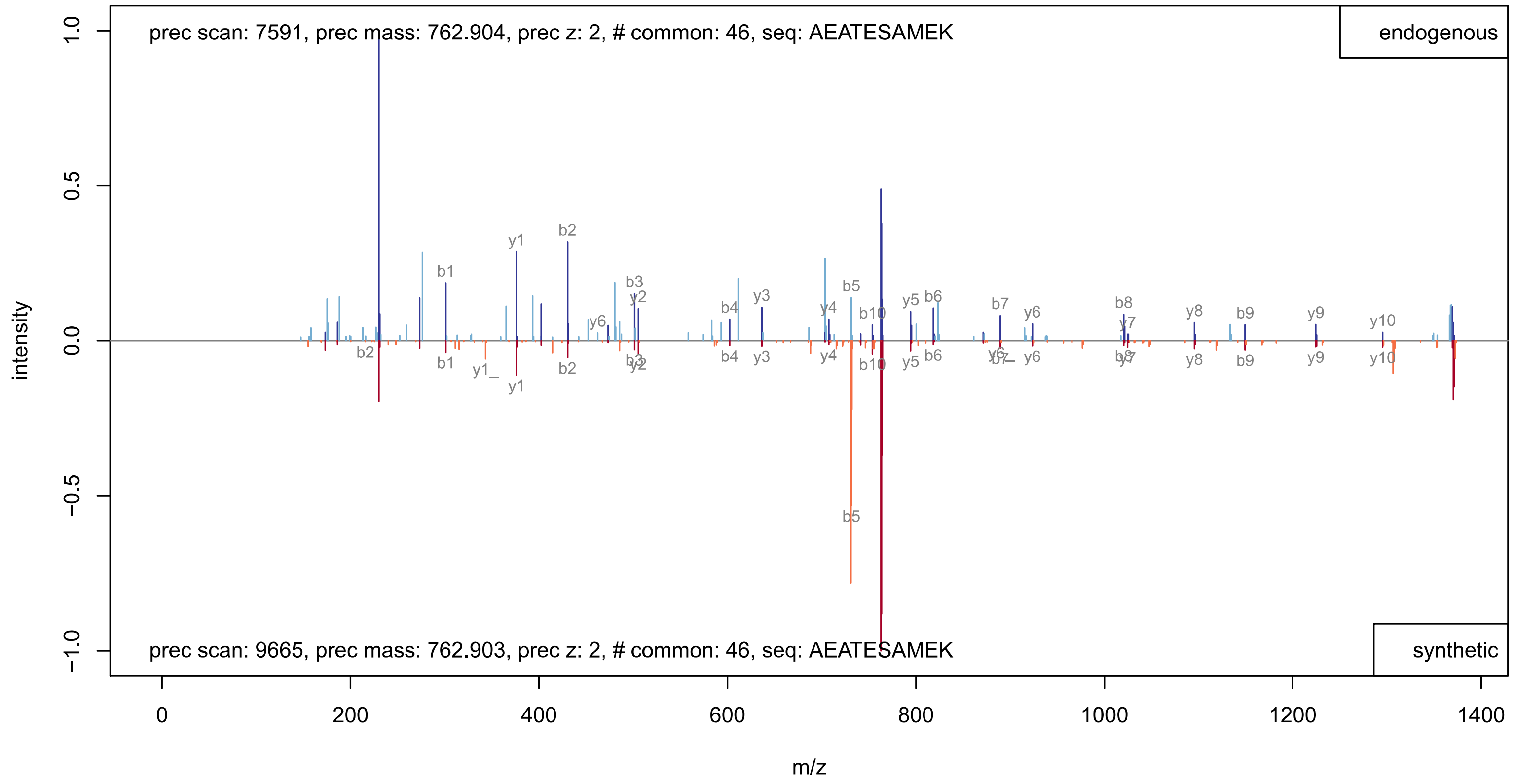

PGOHUM_ENST00000511530.1_GAPDHP71

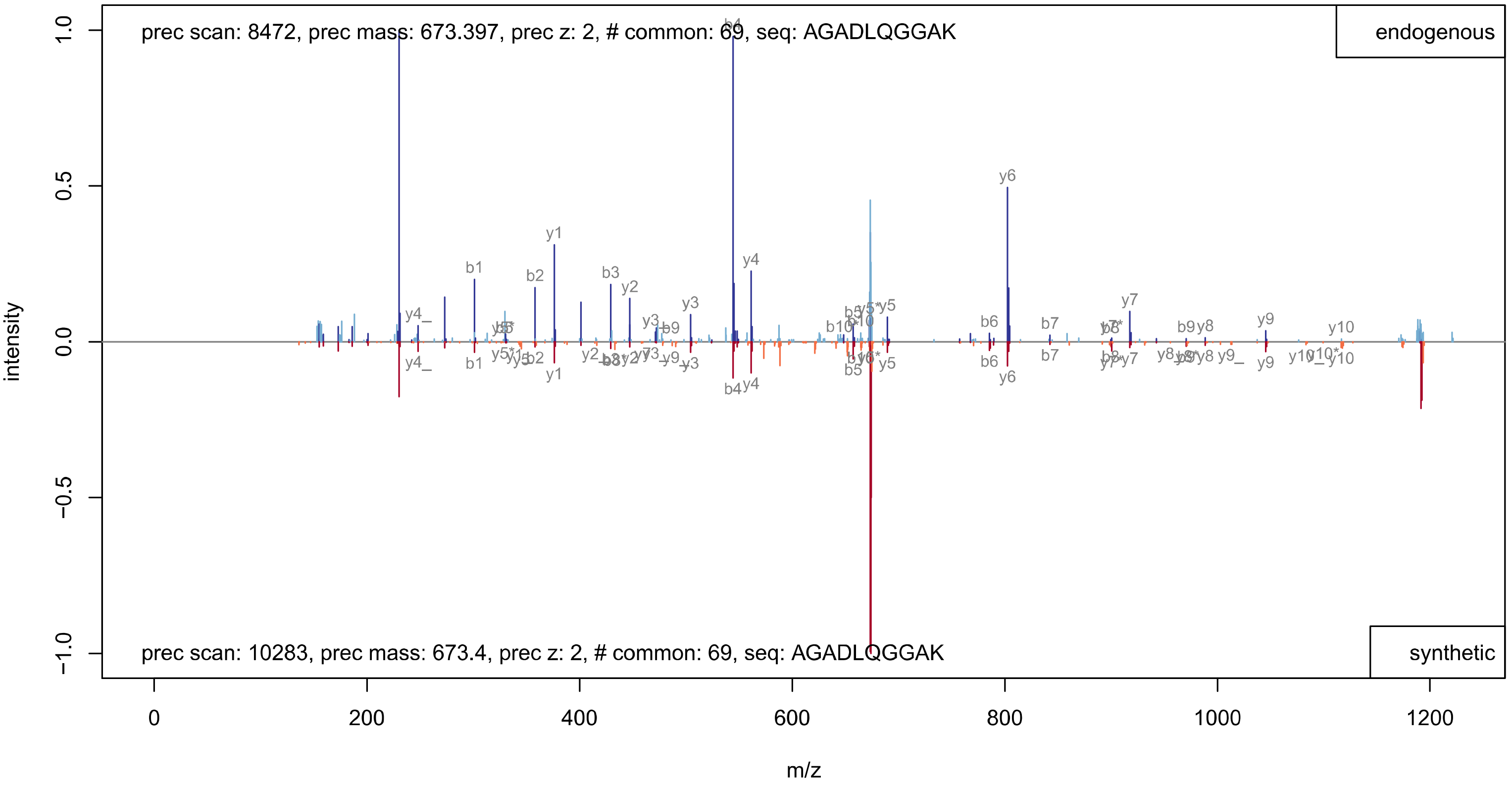

chr5_137089732

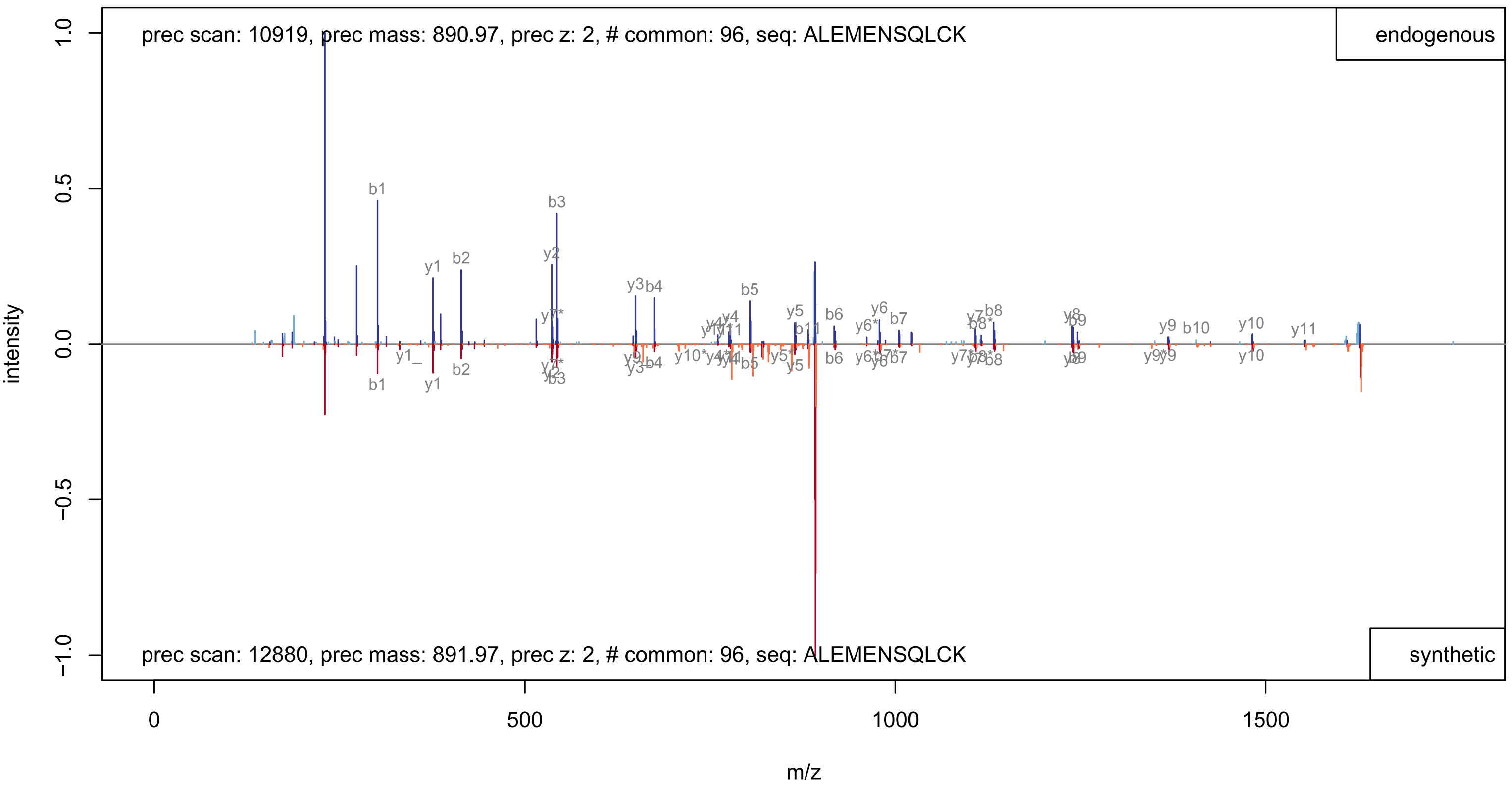

MKI67_G1225V

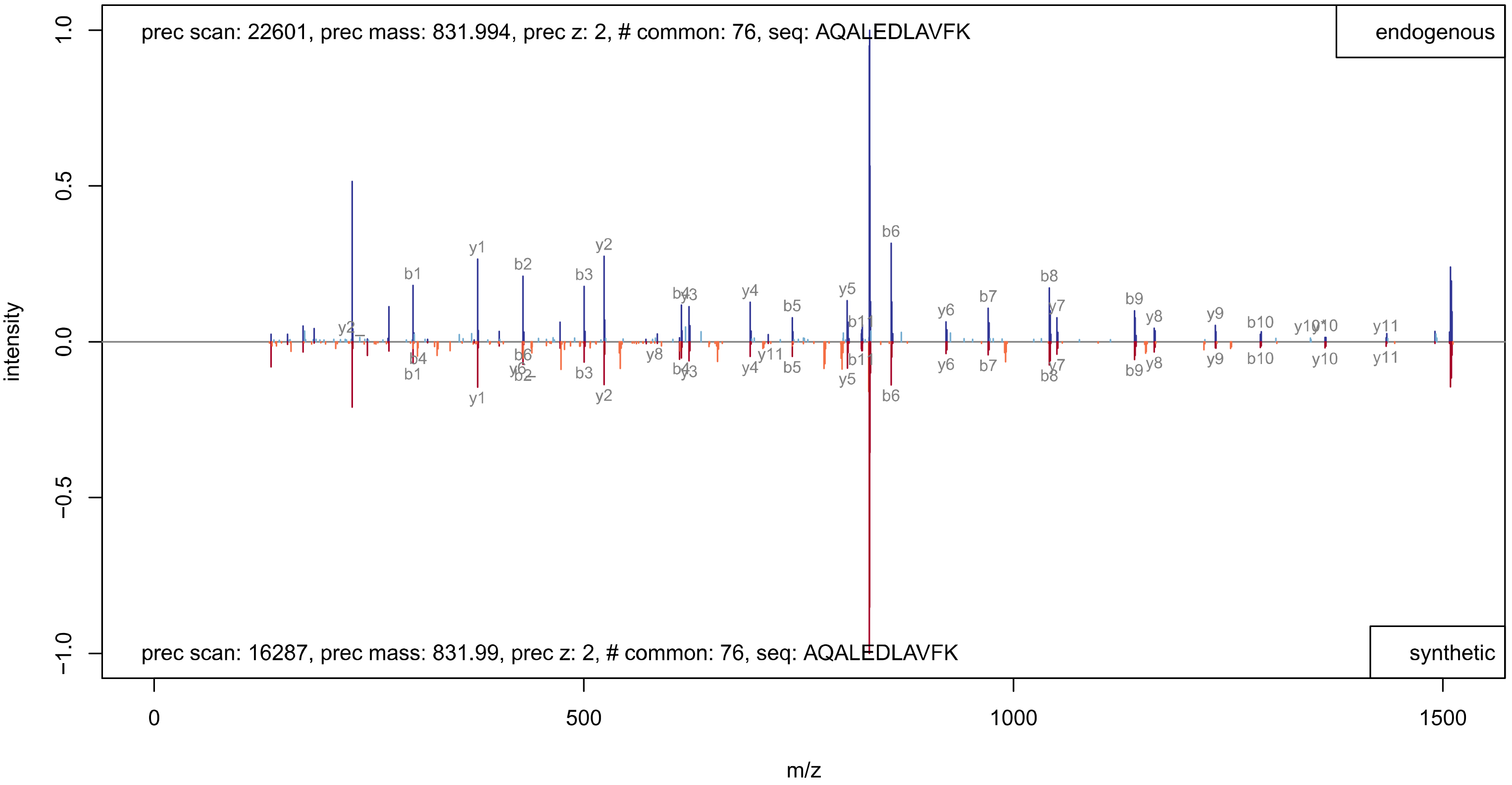

EXO1_E589K

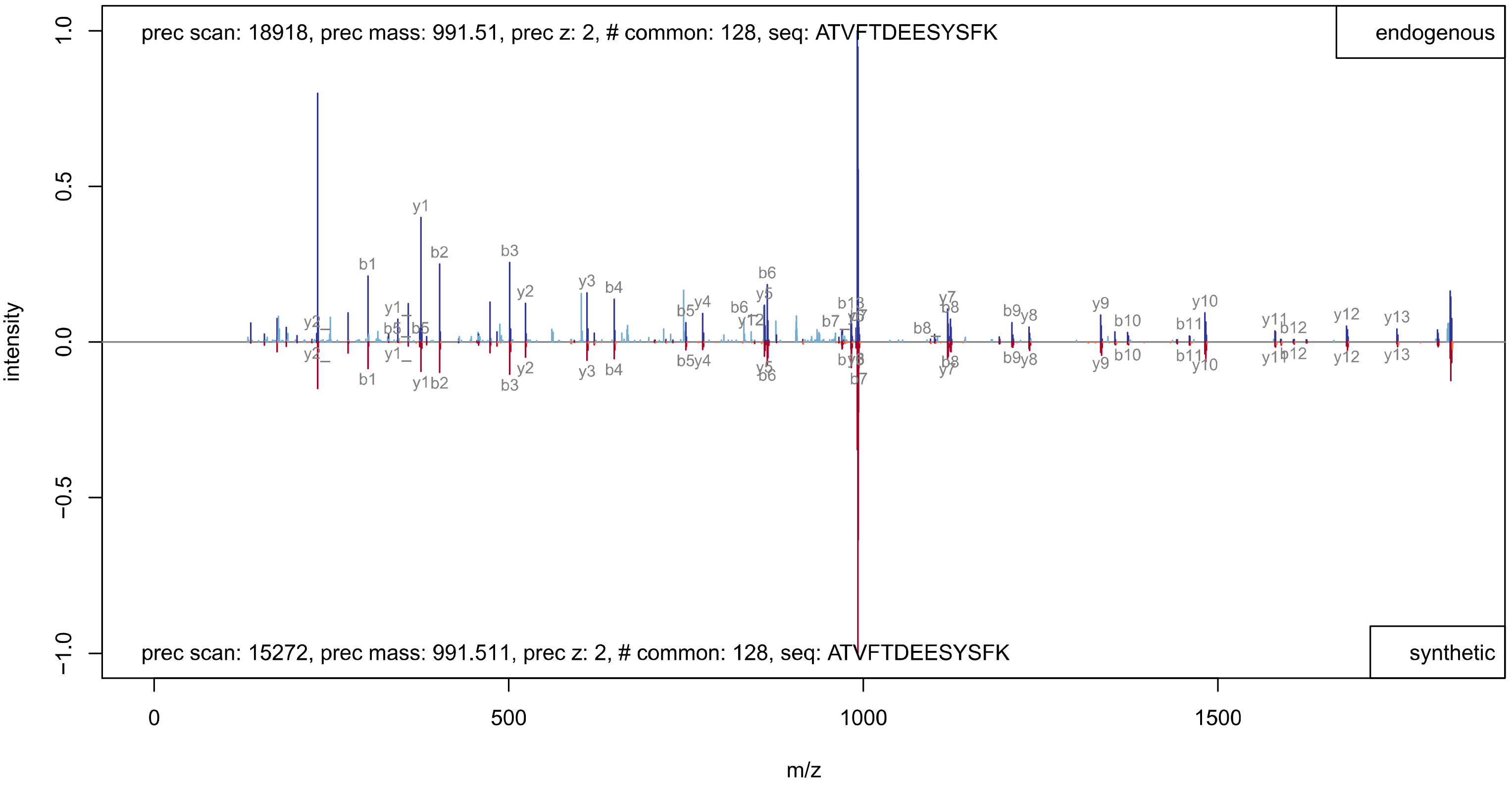

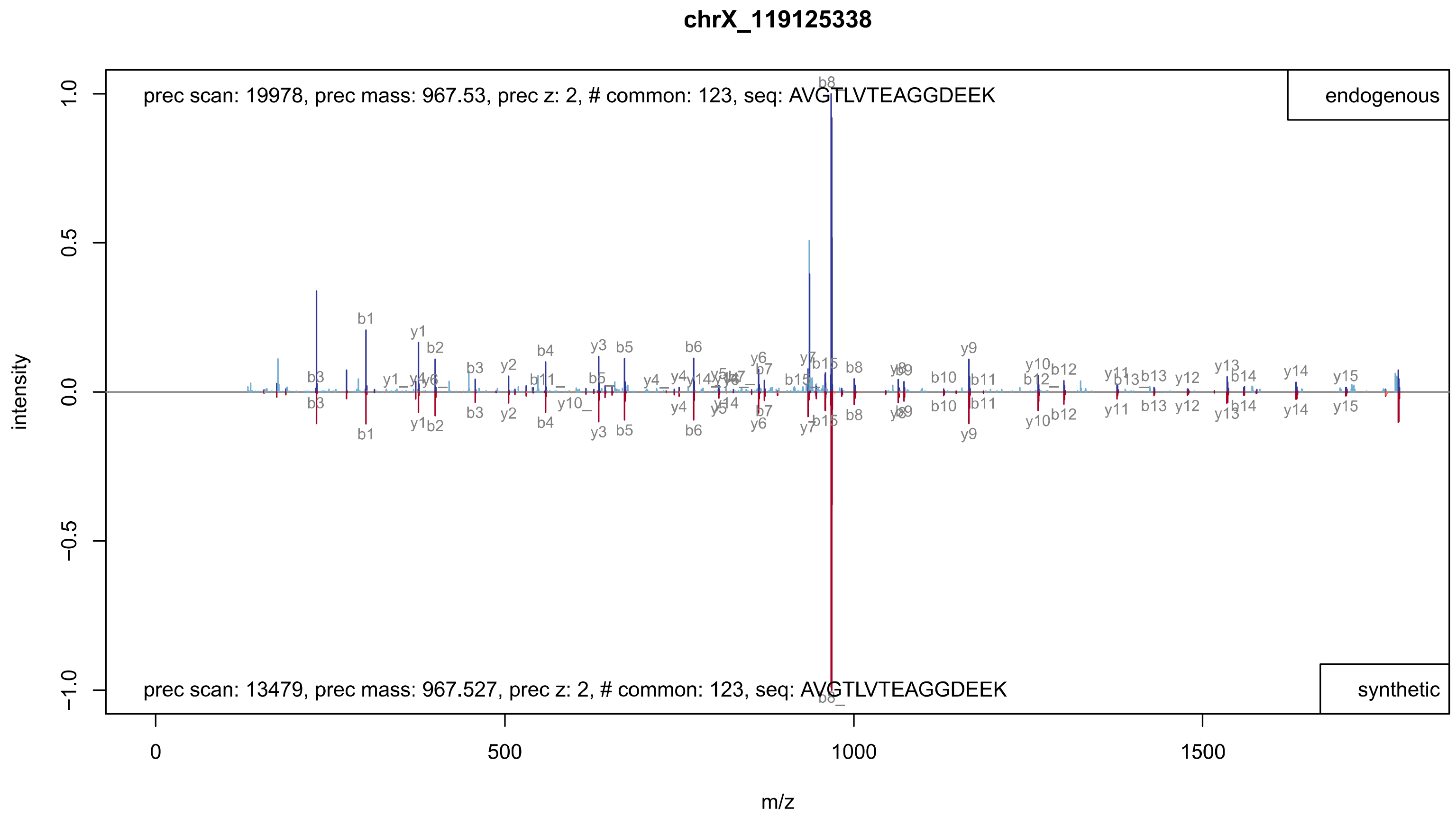
chrX_119125338
prec scan: 19978, prec mass: 967.53, prec z: 2, # common: 123, seq: AVGTLVTEAGGDEEK
endogenous
prec scan: 13479, prec mass: 967.527, prec z: 2, # common: 123, seq: AVGTLVTEAGGDEEK
synthetic
intensity
m/z

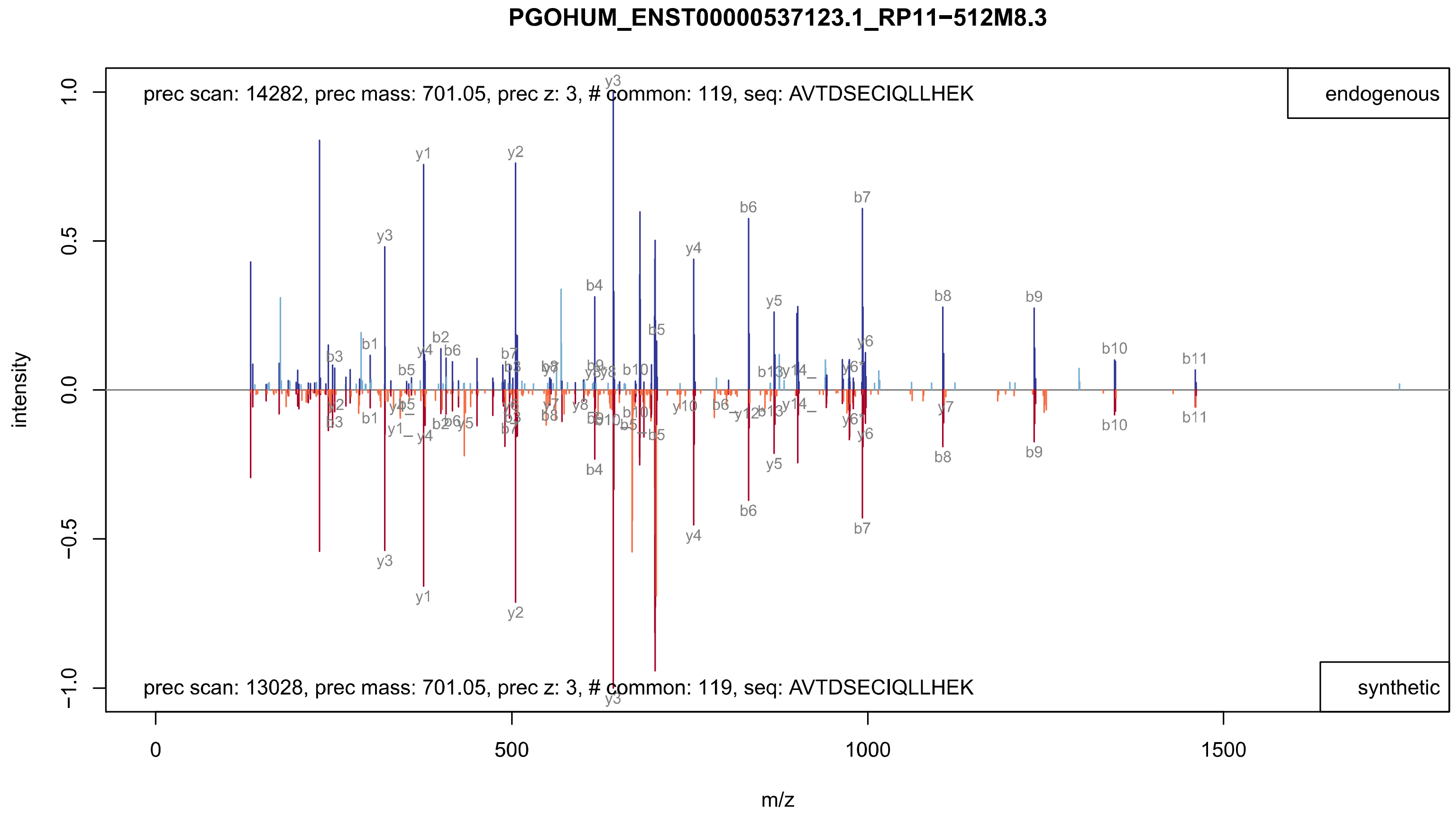
PGOHUM_ENST00000537123.1_RP11−512M8.3
prec scan: 14282, prec mass: 701.05, prec z: 3, # common: 119, seq: AVTDSECIQLLHEK
endogenous
prec scan: 13028, prec mass: 701.05, prec z: 3, # common: 119, seq: AVTDSECIQLLHEK
synthetic
intensity
m/z

chr11_107047730

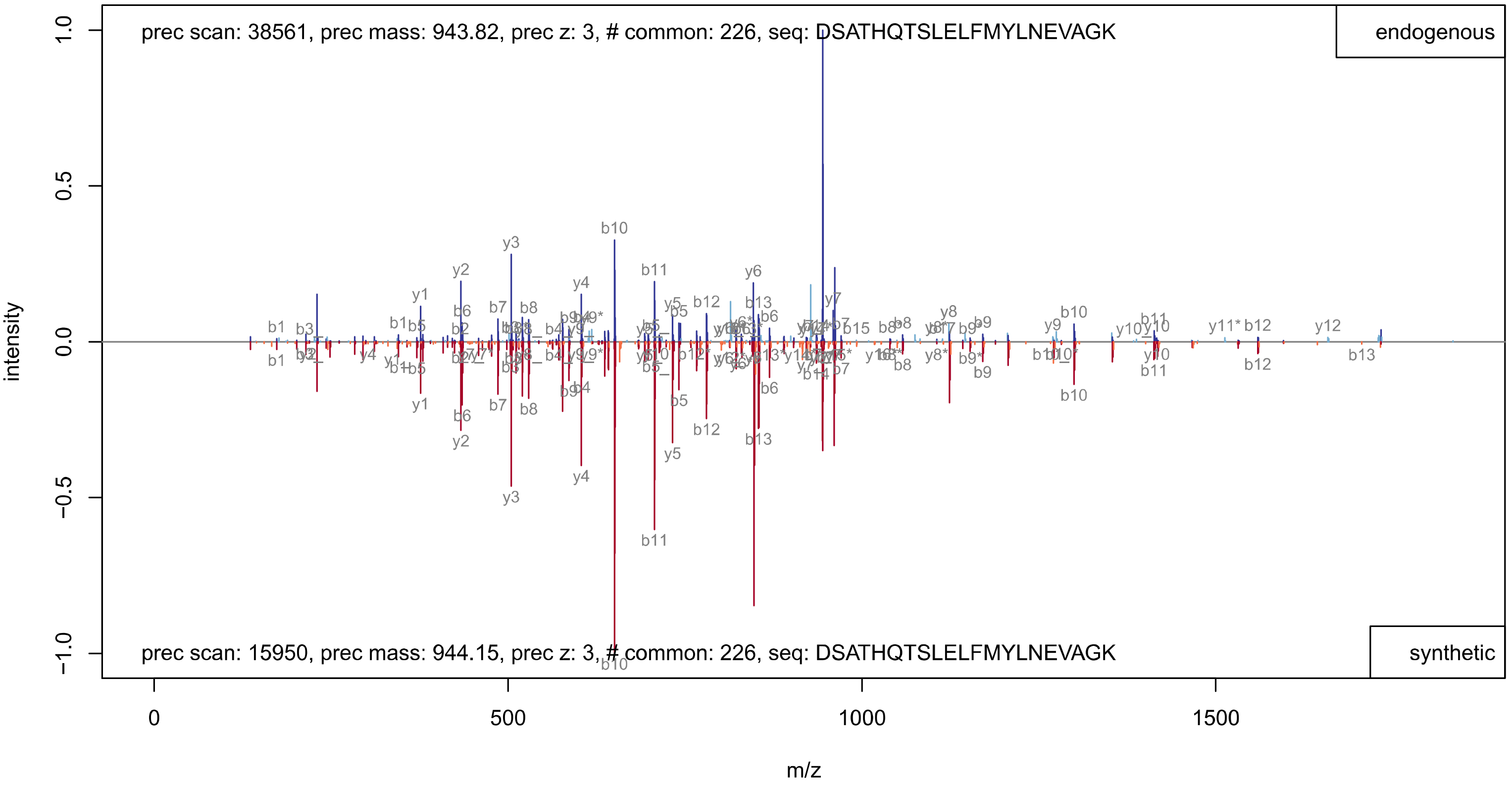

TP53RK_T129A

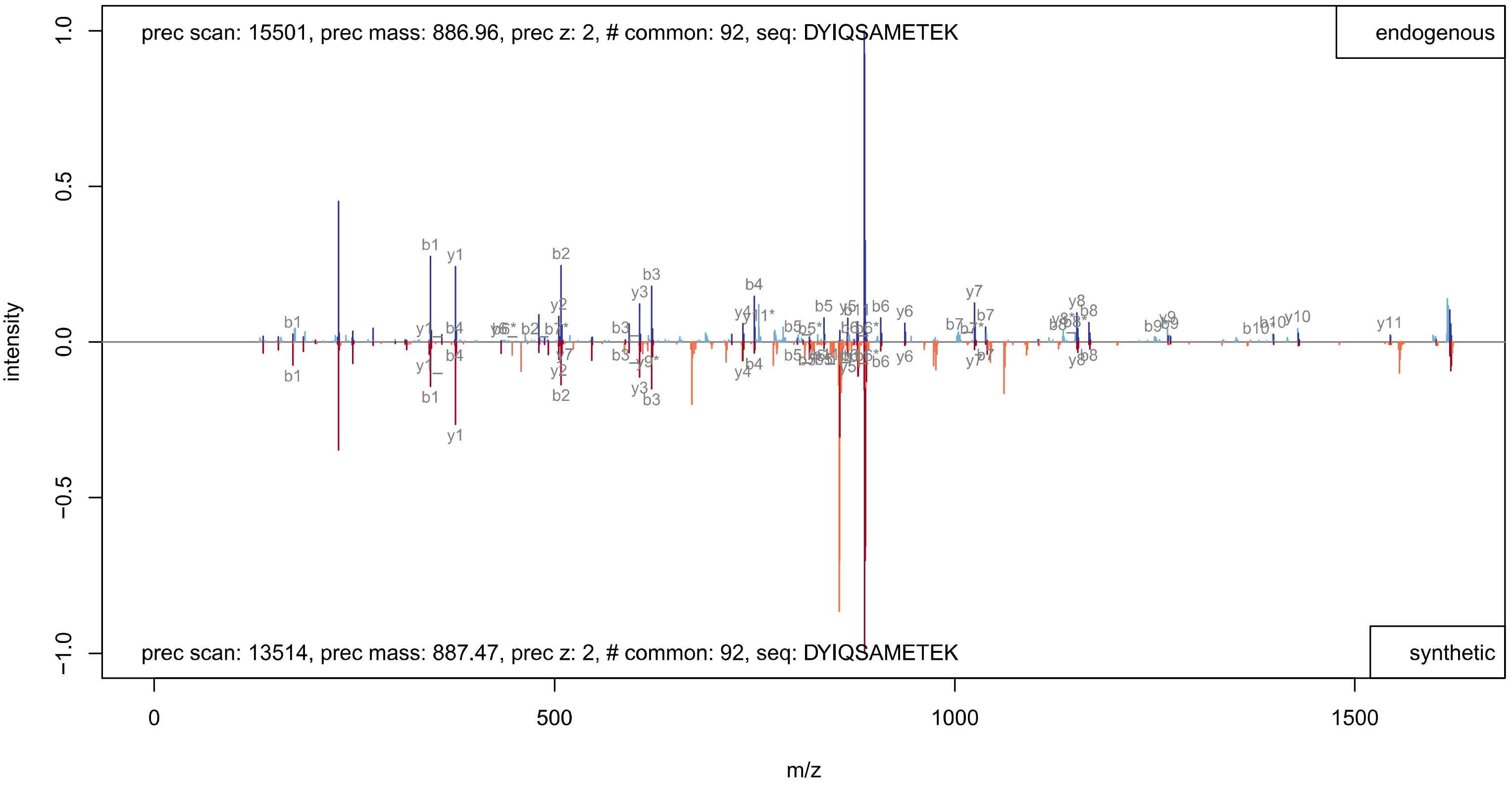

PGOHUM_ENST00000533003.1_KRT8P7

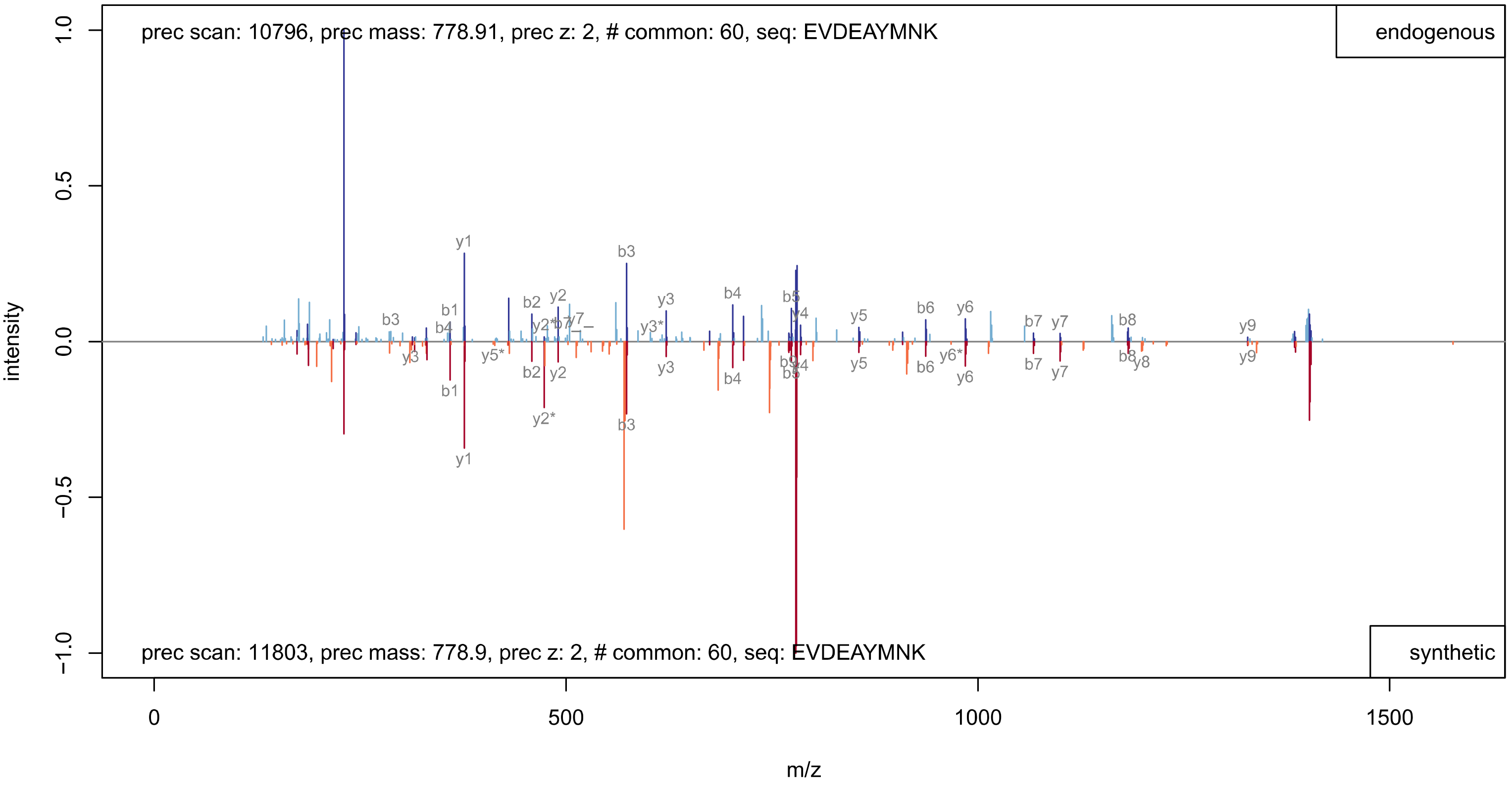

chr2_139659936

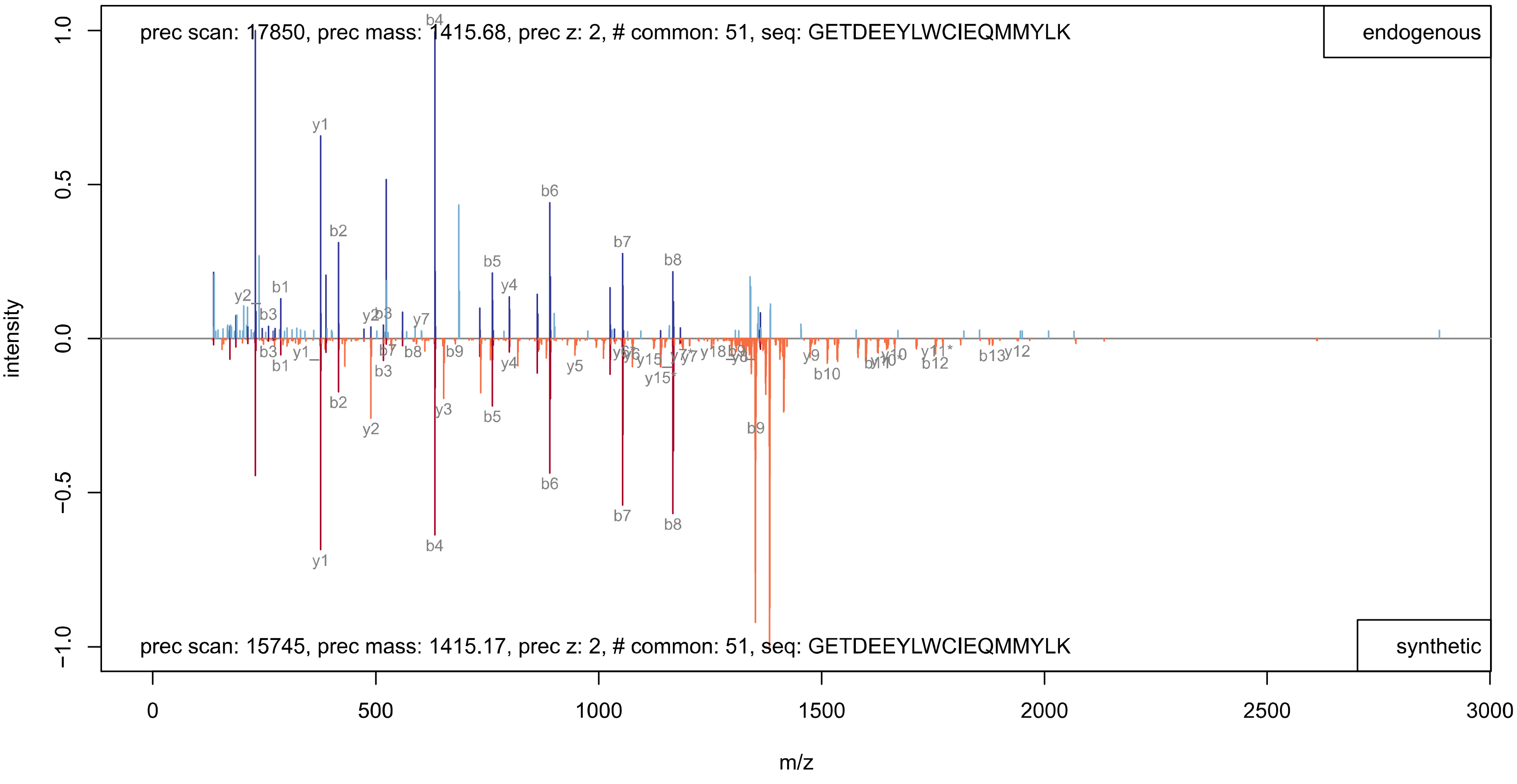

chr9_91025895

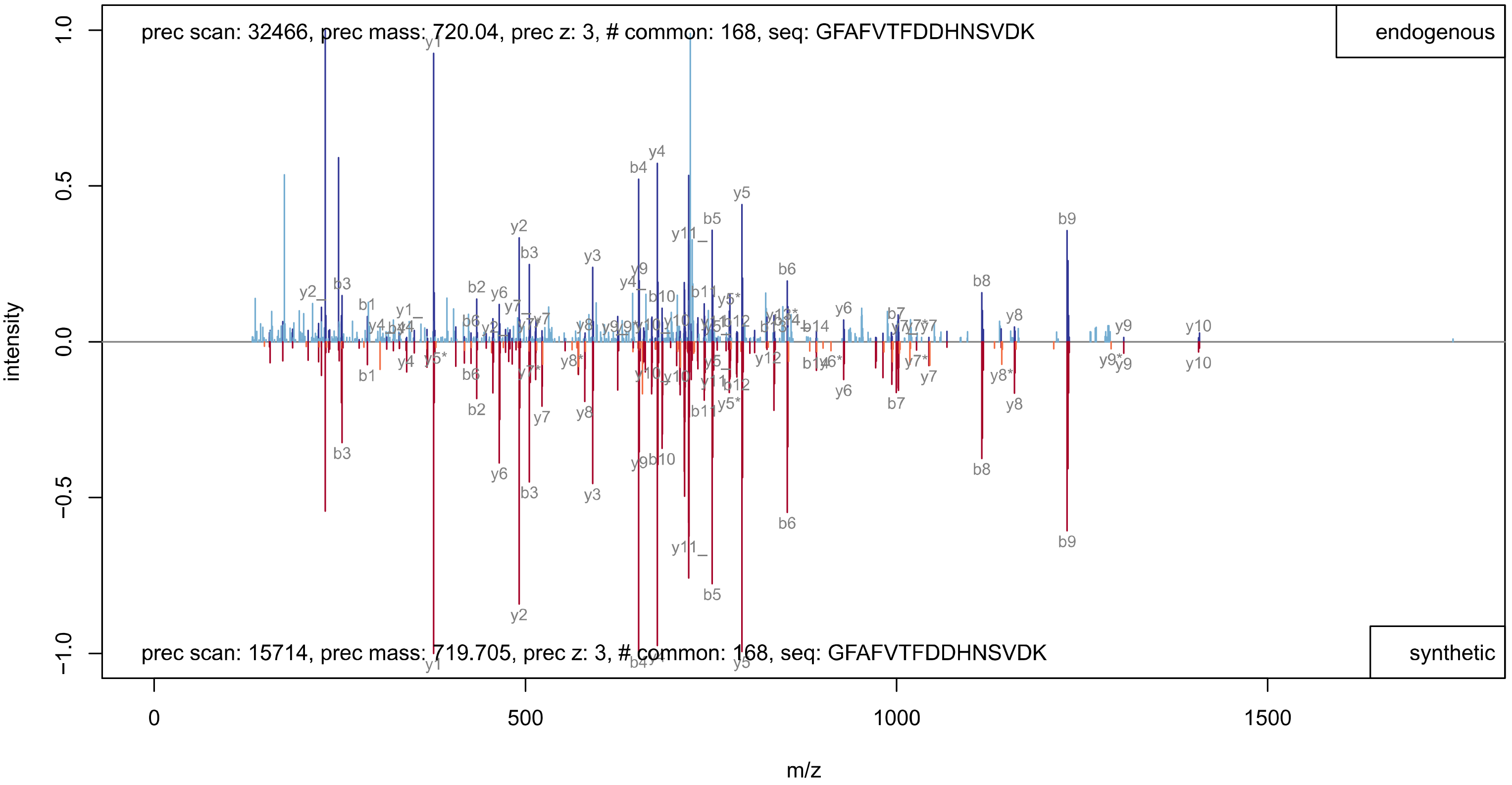

PGOHUM_ENST00000440547.1_HNRNPA1P39

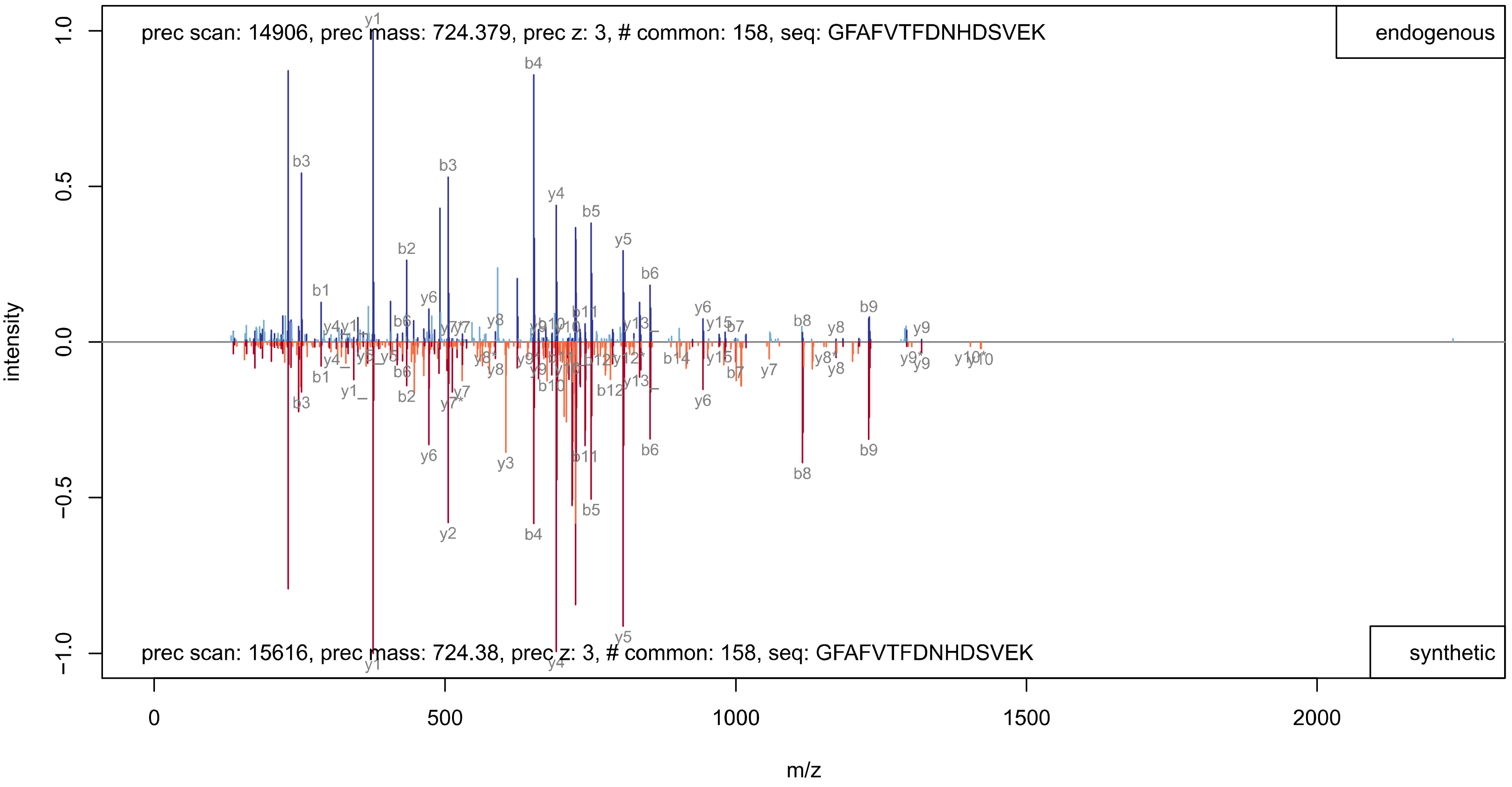

PGOHUM_ENST00000512891.1_RP11−889L3.4

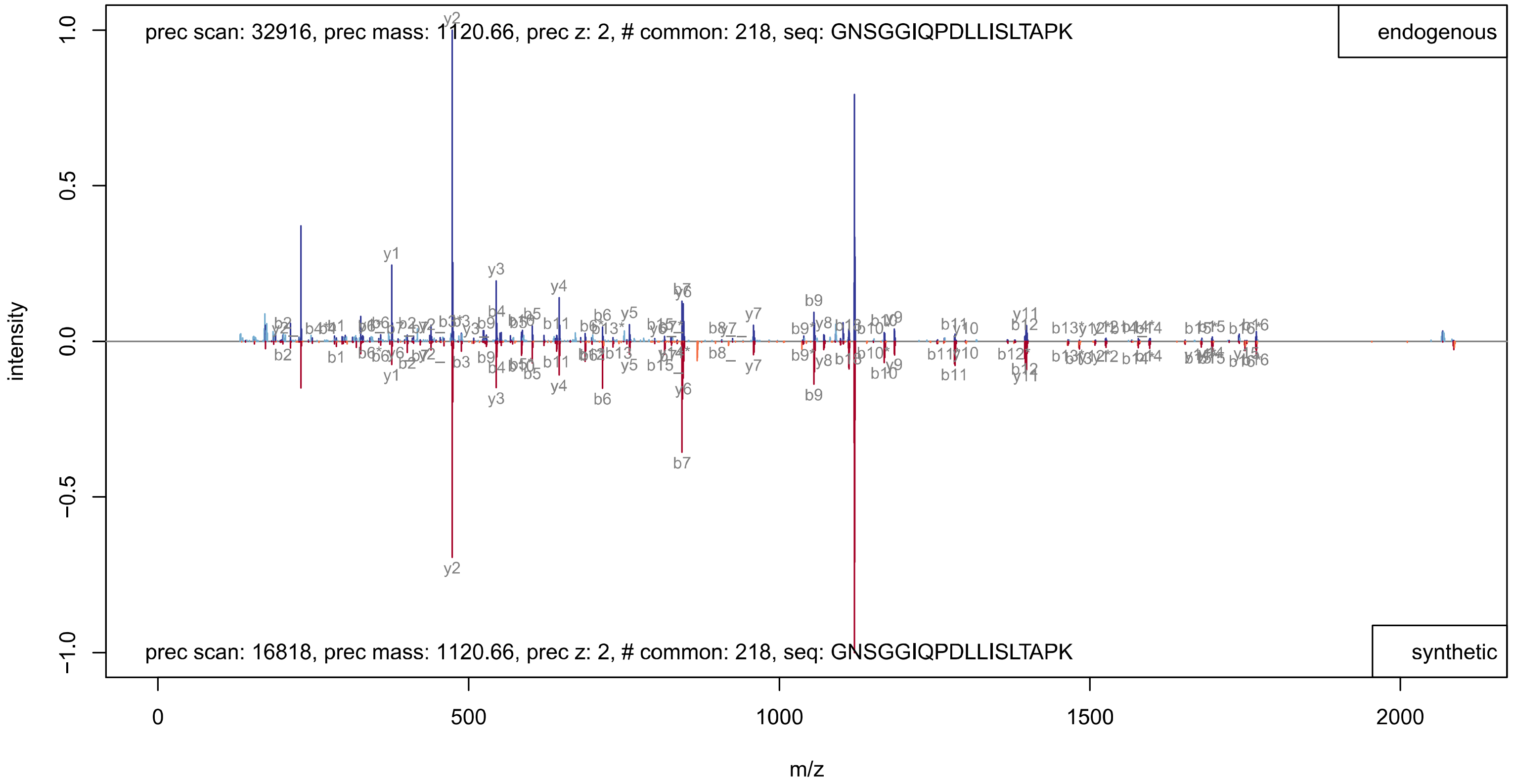

KRT14_V452I

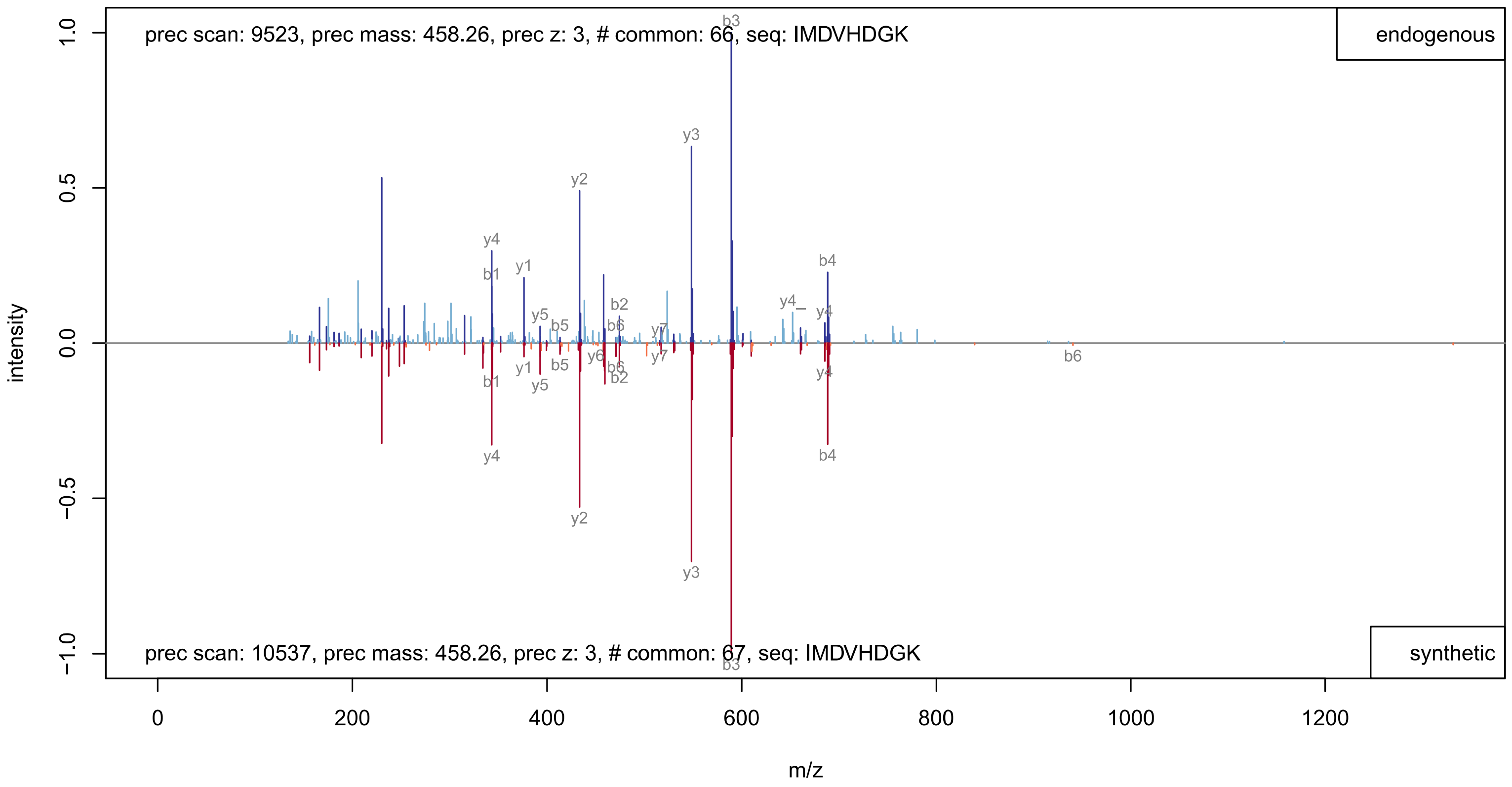

ADARB1_ENST00000389863_E700Q

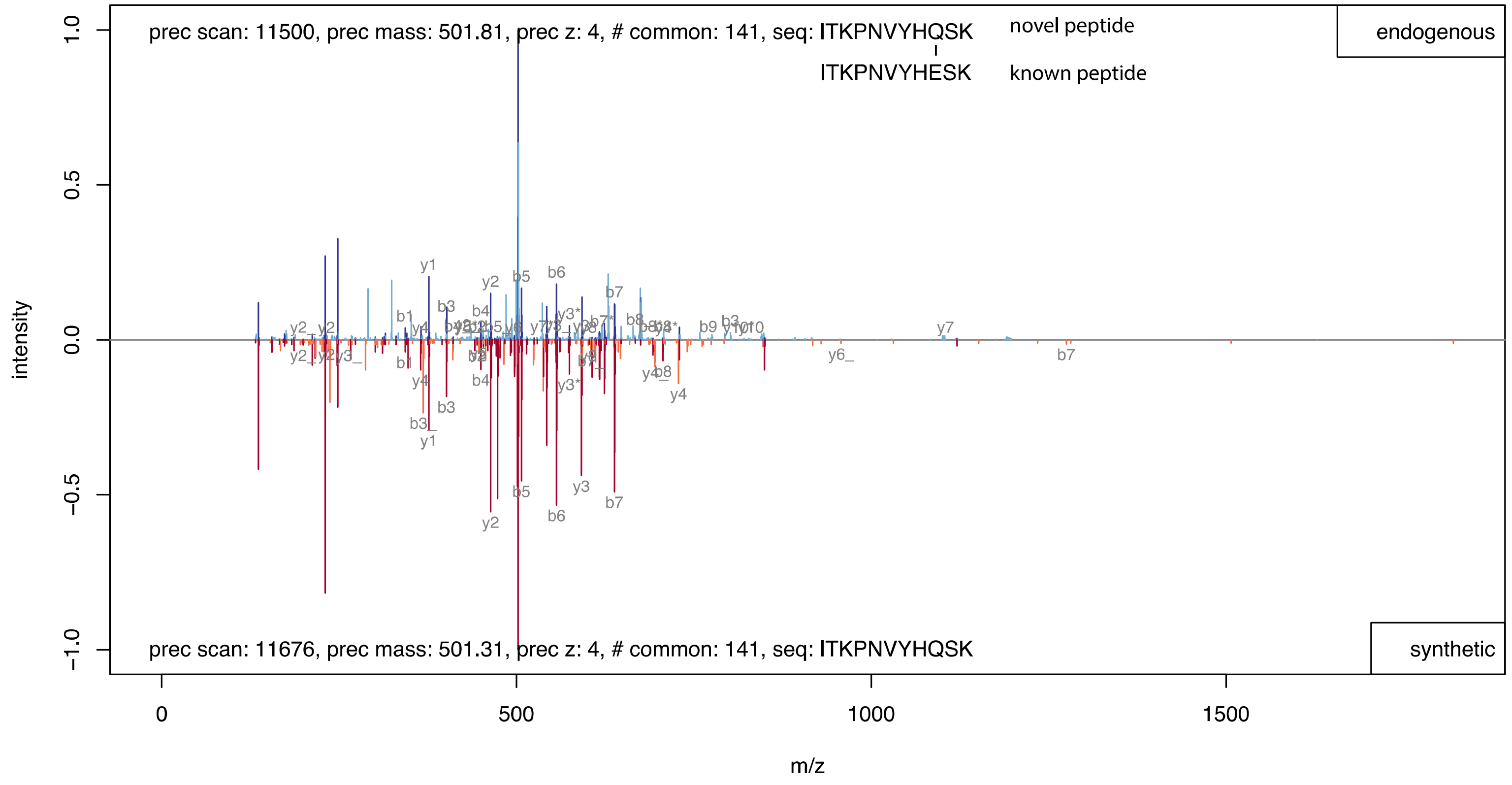

chr12_57108182

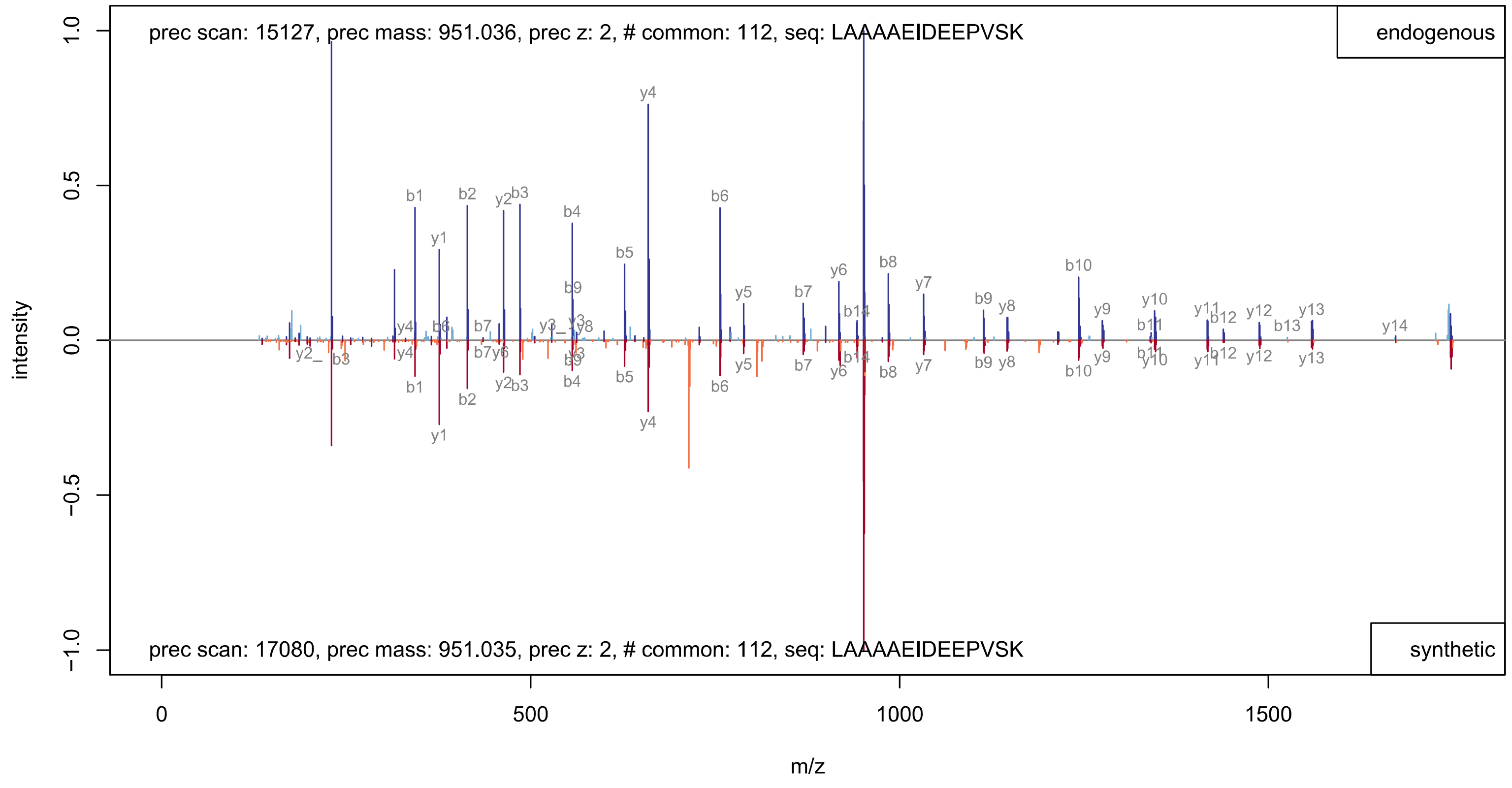

PGOHUM_ENST00000419201.1_AHCYP4

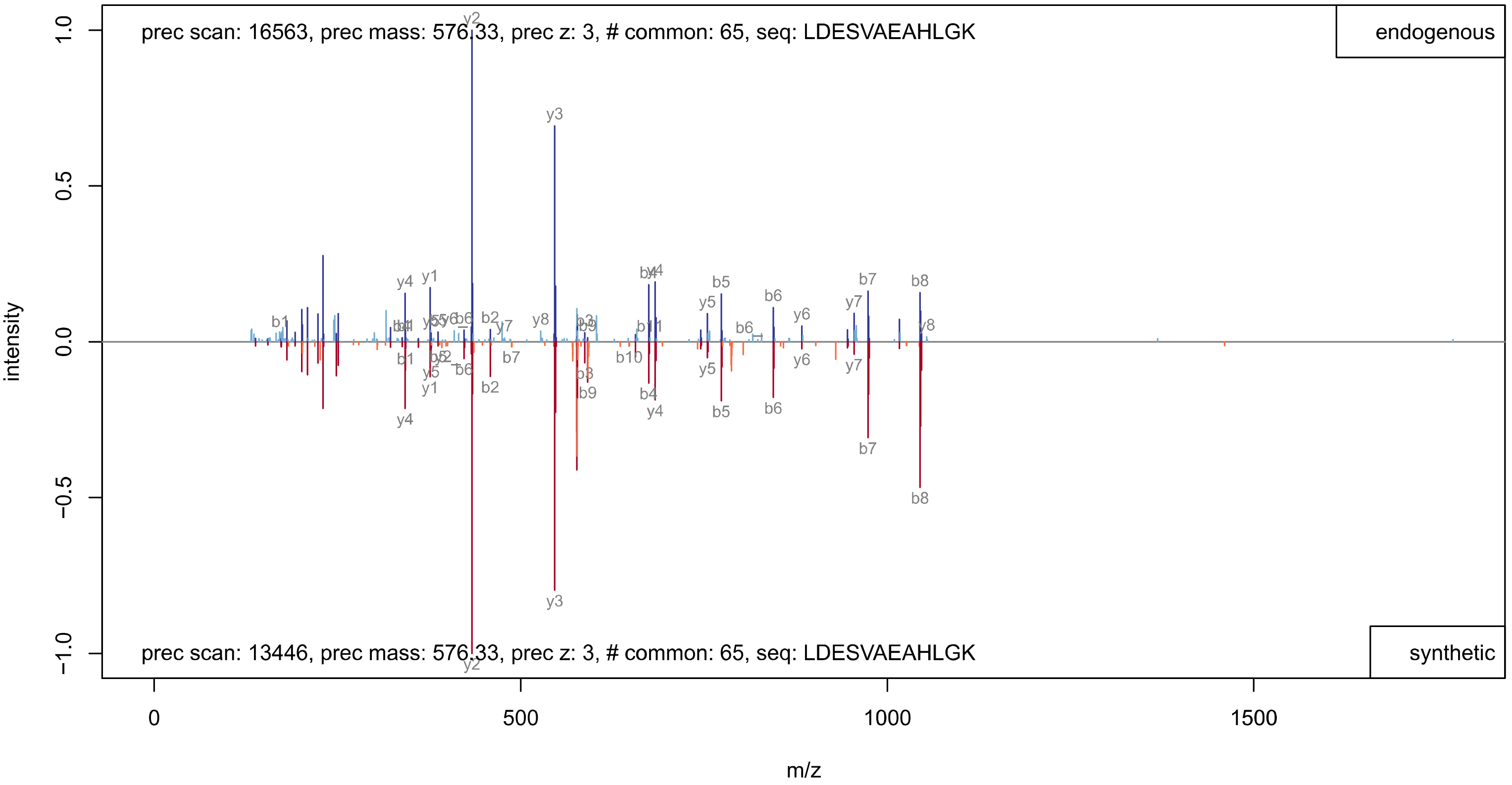

CDH11_V221L

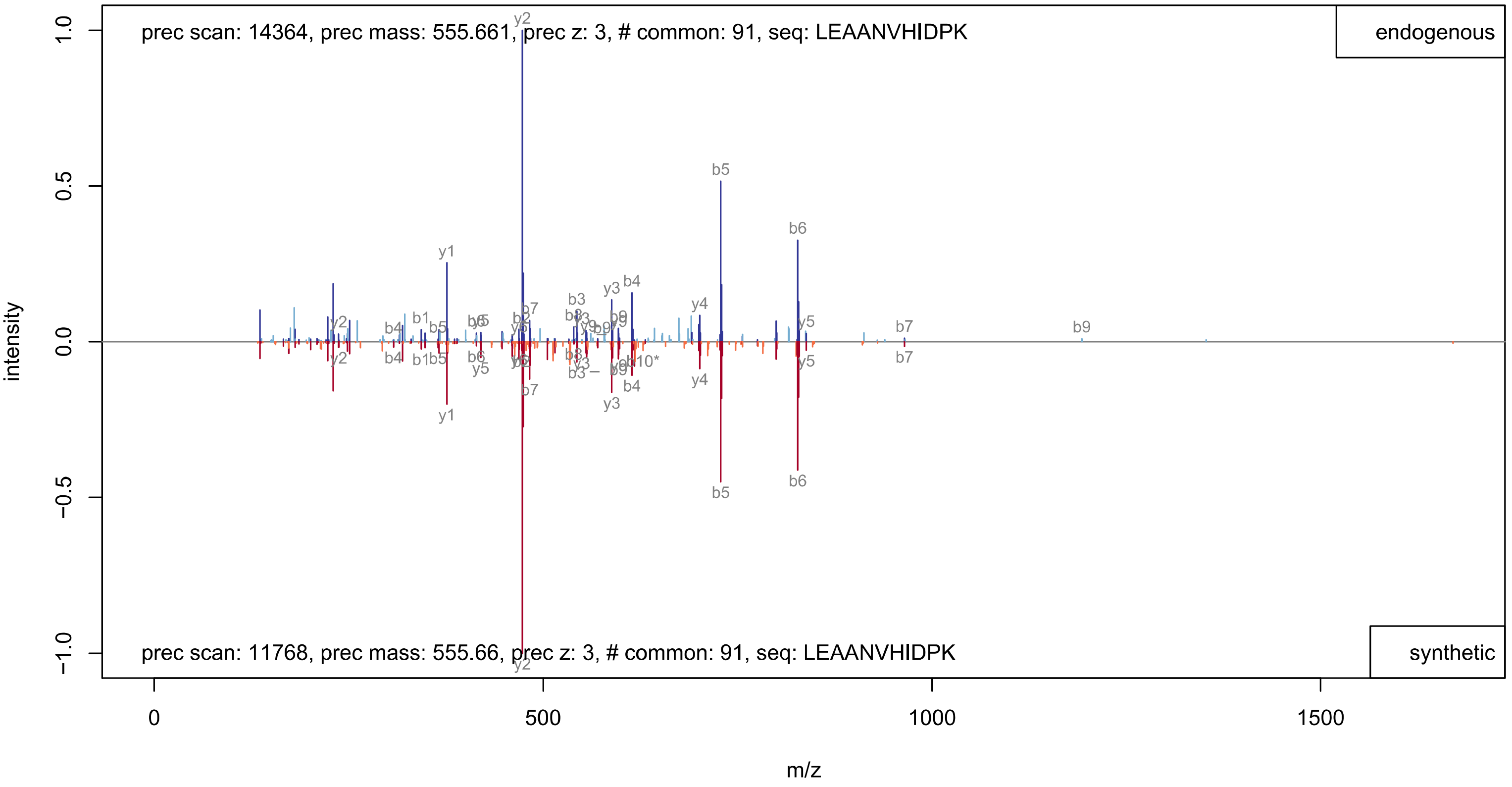

chr19_36258564

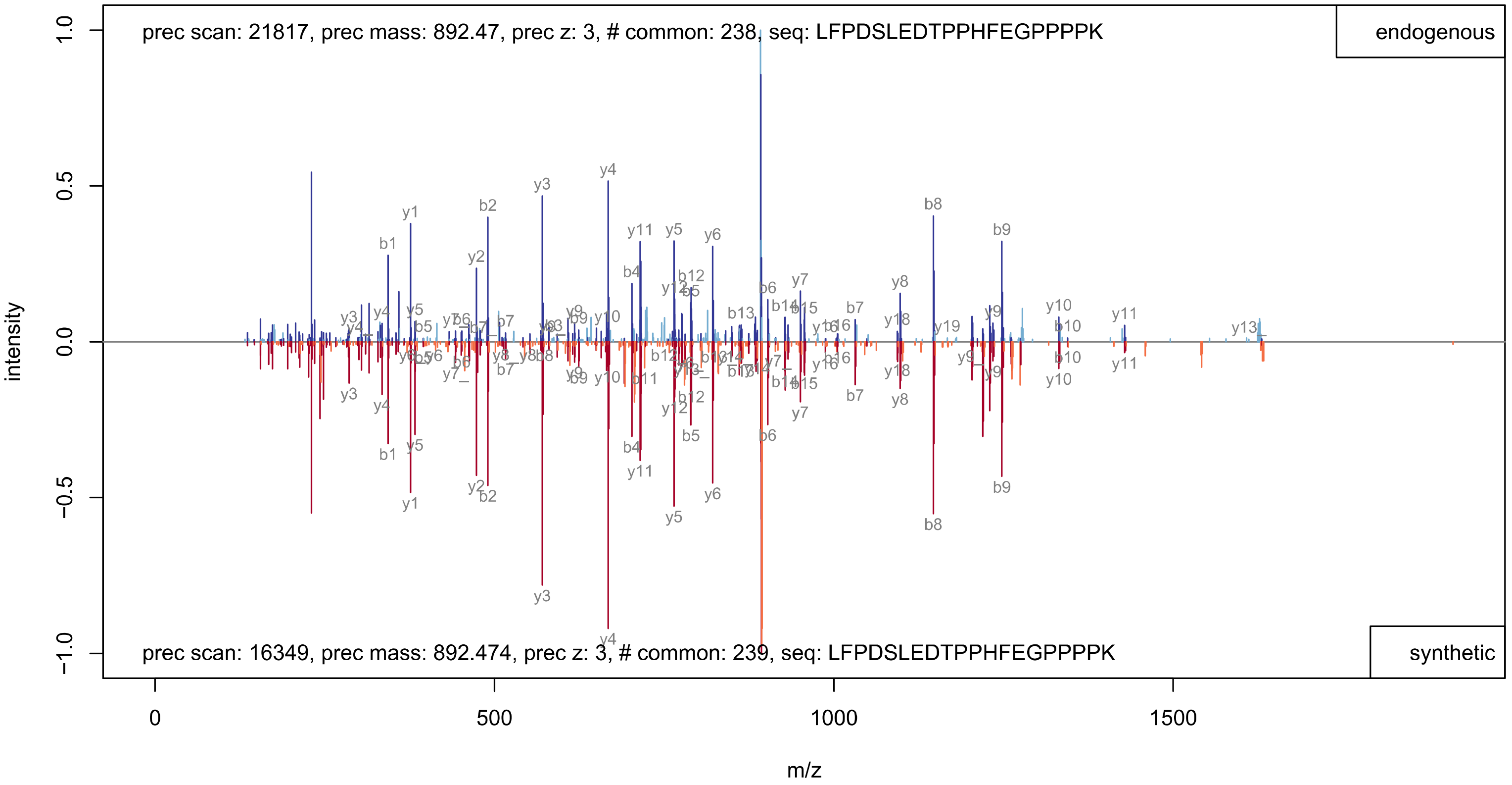

MYBBP1A_A311E

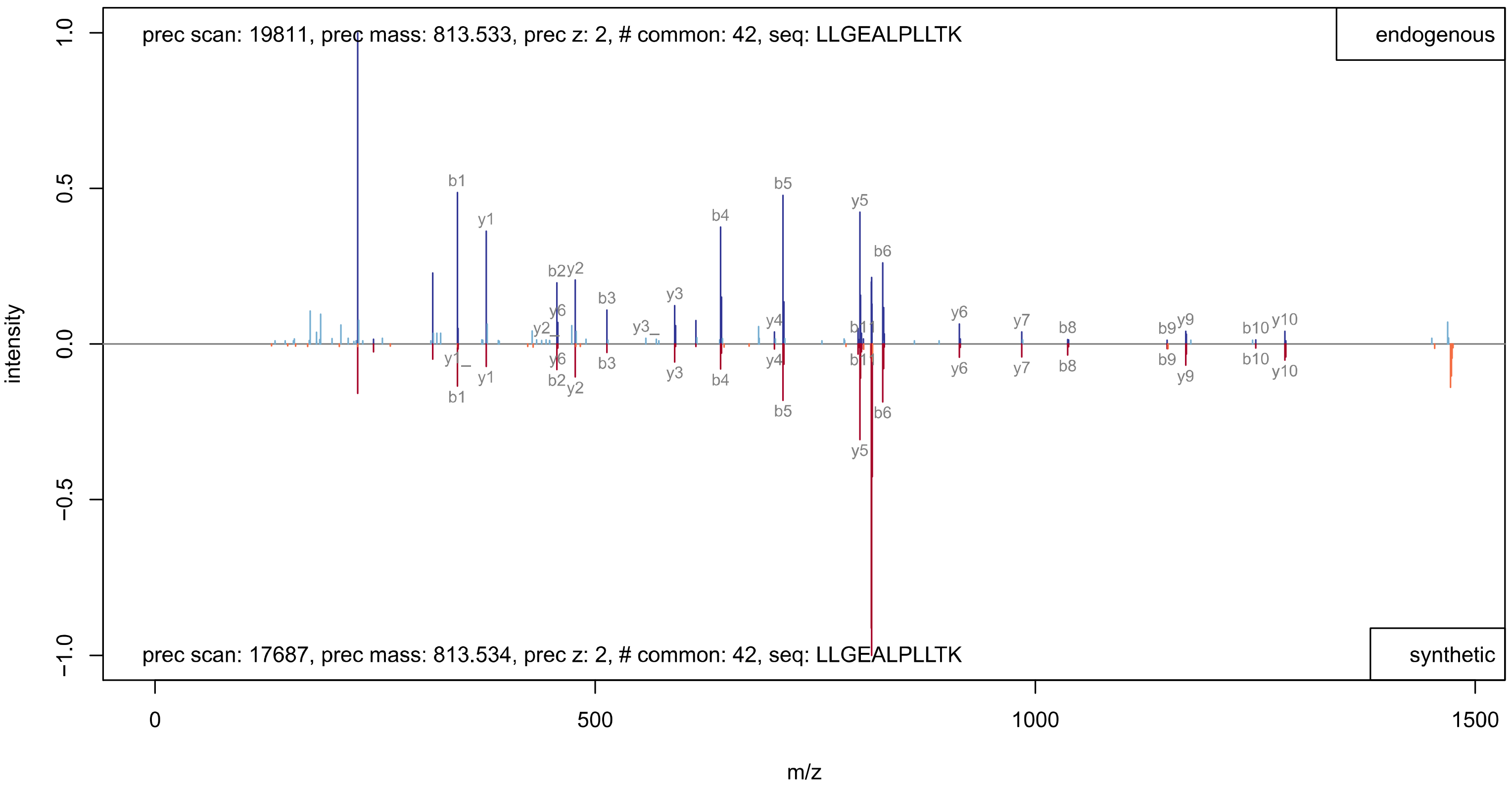

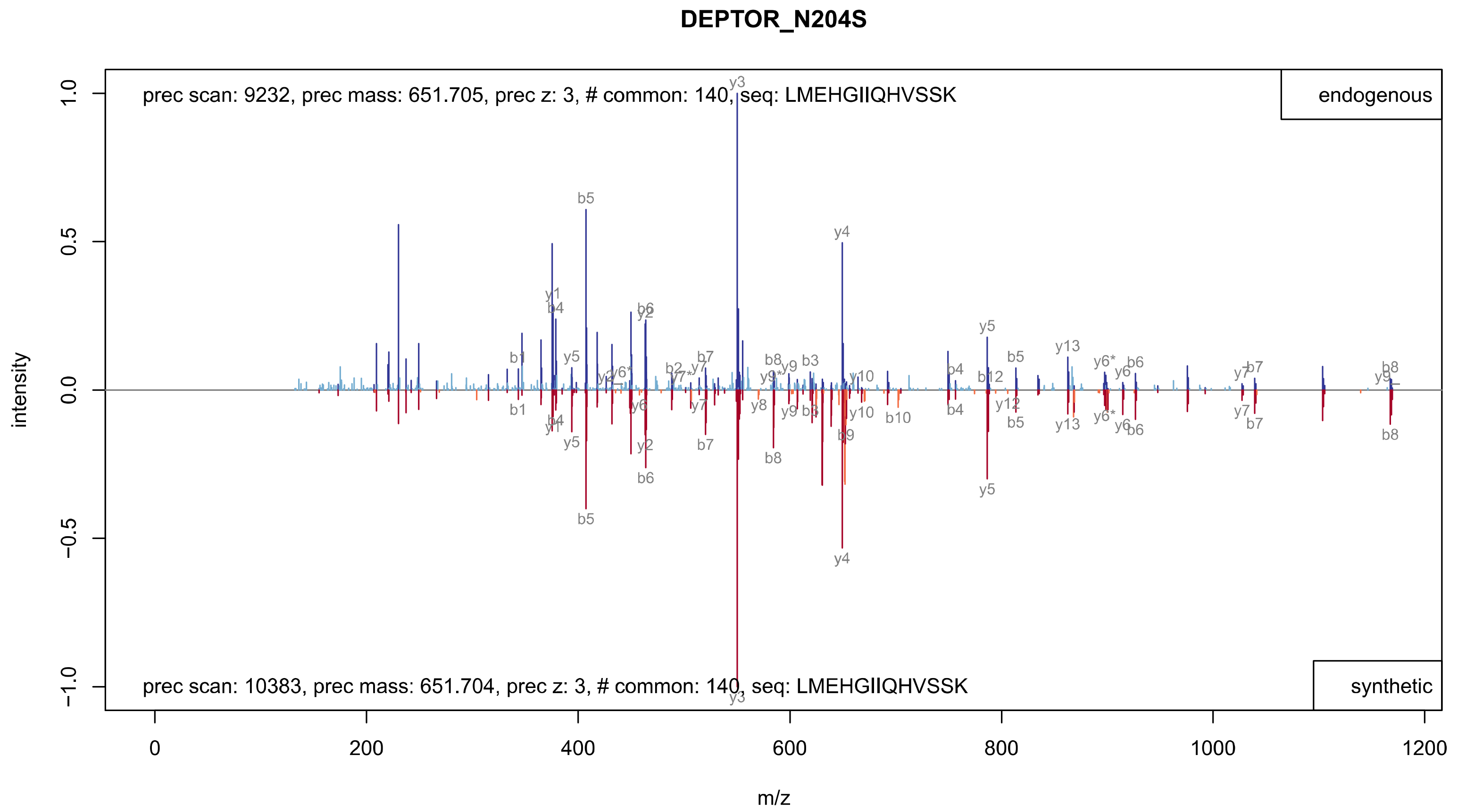
DEPTOR_N204S
prec scan: 9232, prec mass: 651.705, prec z: 3, # common: 140, seq: LMEHGIIQHVSSK
endogenous
prec scan: 10383, prec mass: 651.704, prec z: 3, # common: 140, seq: LMEHGIIQHVSSK
synthetic
intensity
m/z
1.0
0.5
0.0
−0.5
−1.0
0
200
400
600
800
1000
1200

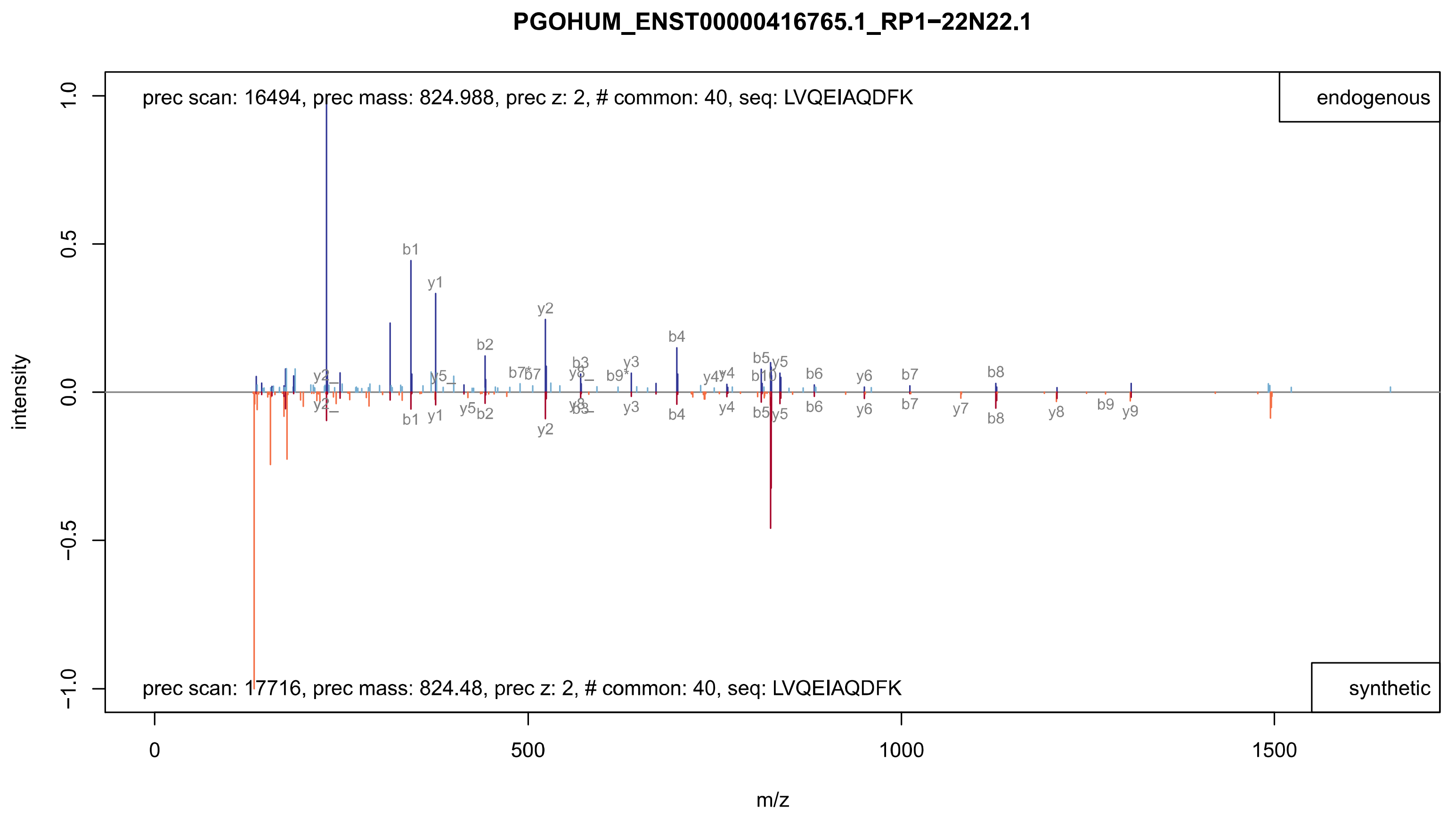
PGOHUM_ENST00000416765.1_RP1−22N22.1
prec scan: 16494, prec mass: 824.988, prec z: 2, # common: 40, seq: LVQEIAQDFK
endogenous
prec scan: 17716, prec mass: 824.48, prec z: 2, # common: 40, seq: LVQEIAQDFK
synthetic
intensity
m/z
1.0
0.5
0.0
−0.5
−1.0
0
500
1000
1500

chr20_30135673

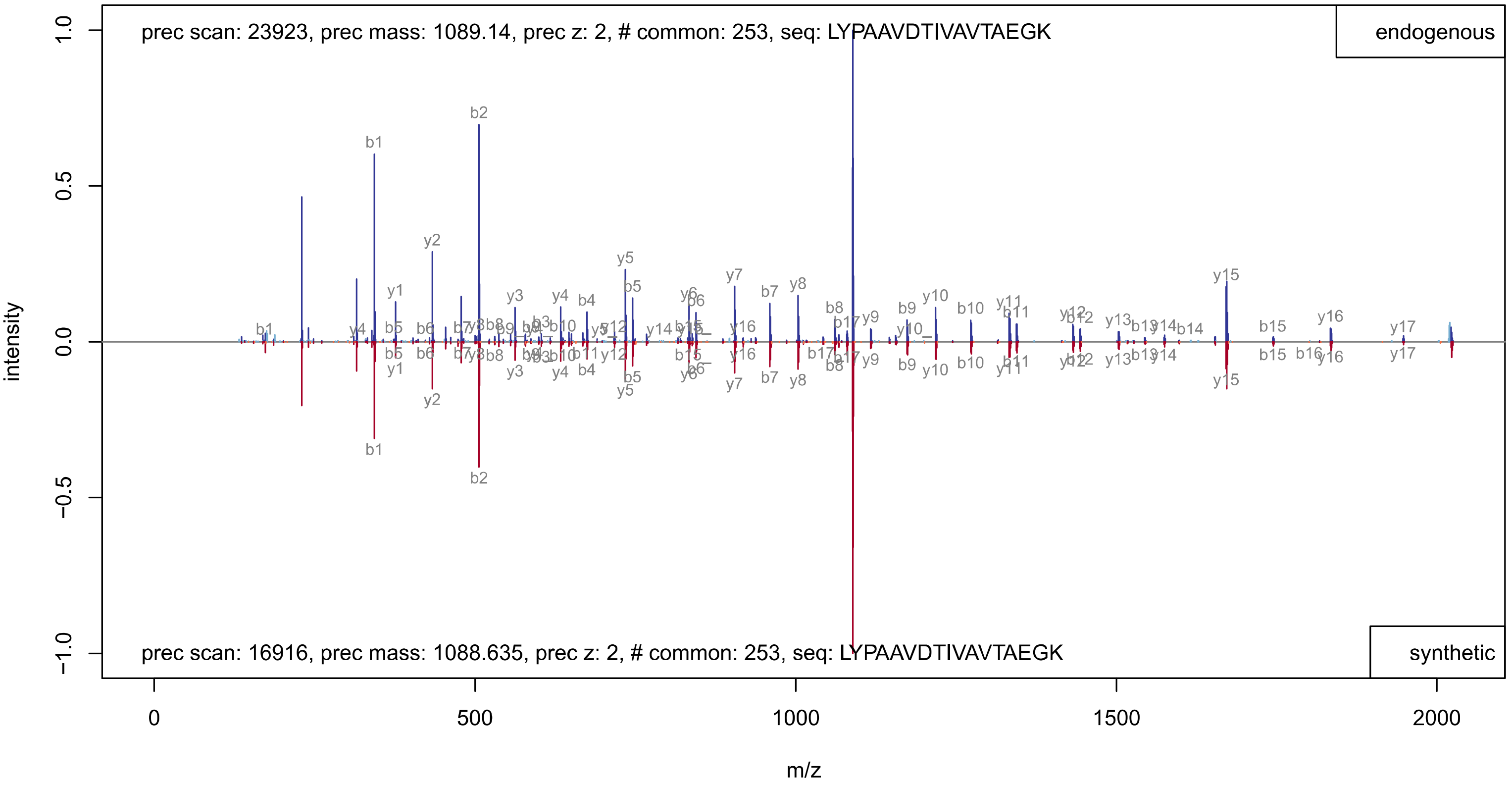

PGOHUM_ENST00000444823.1_GAPDHP23

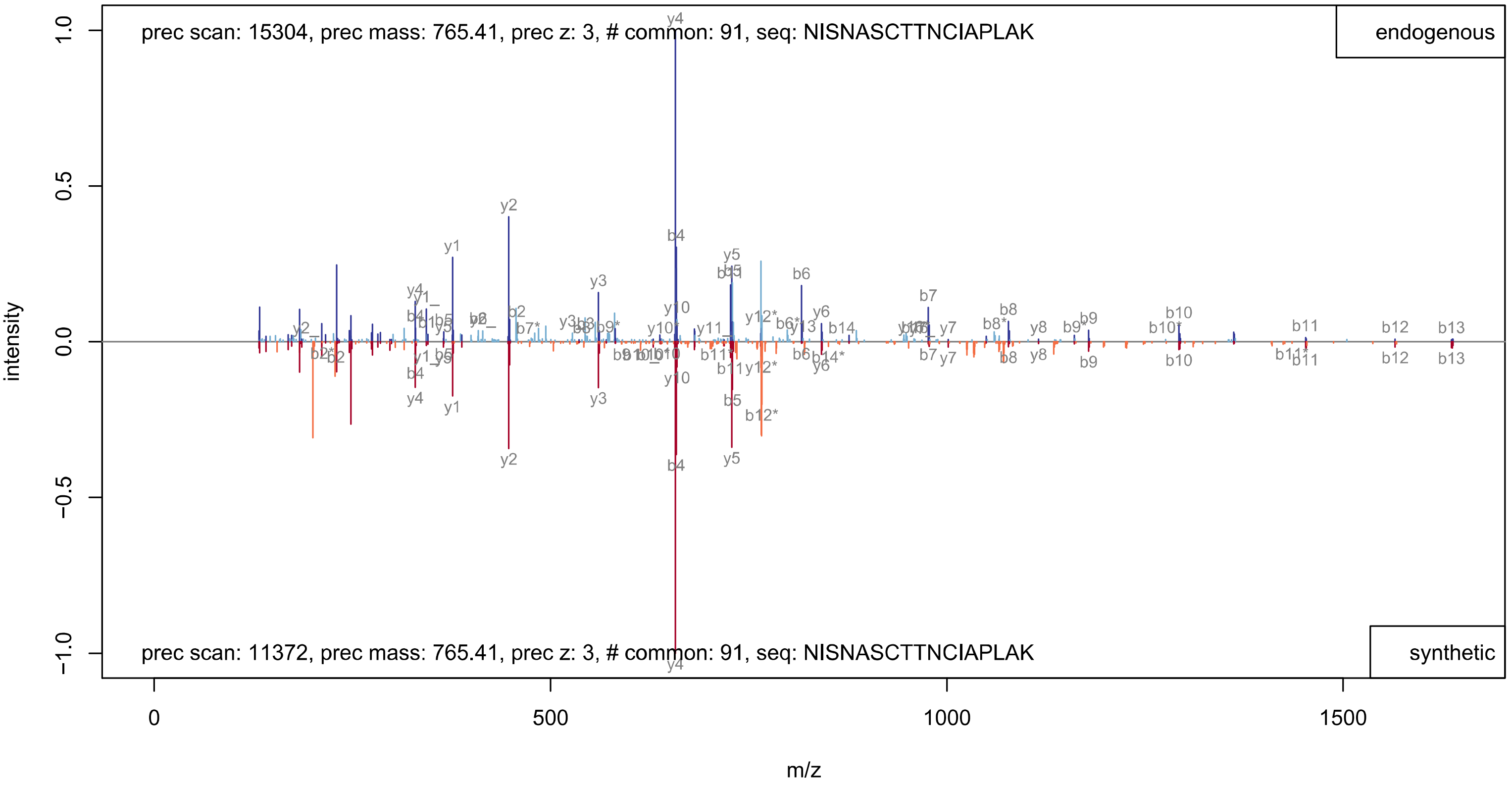

KRT5_A340S

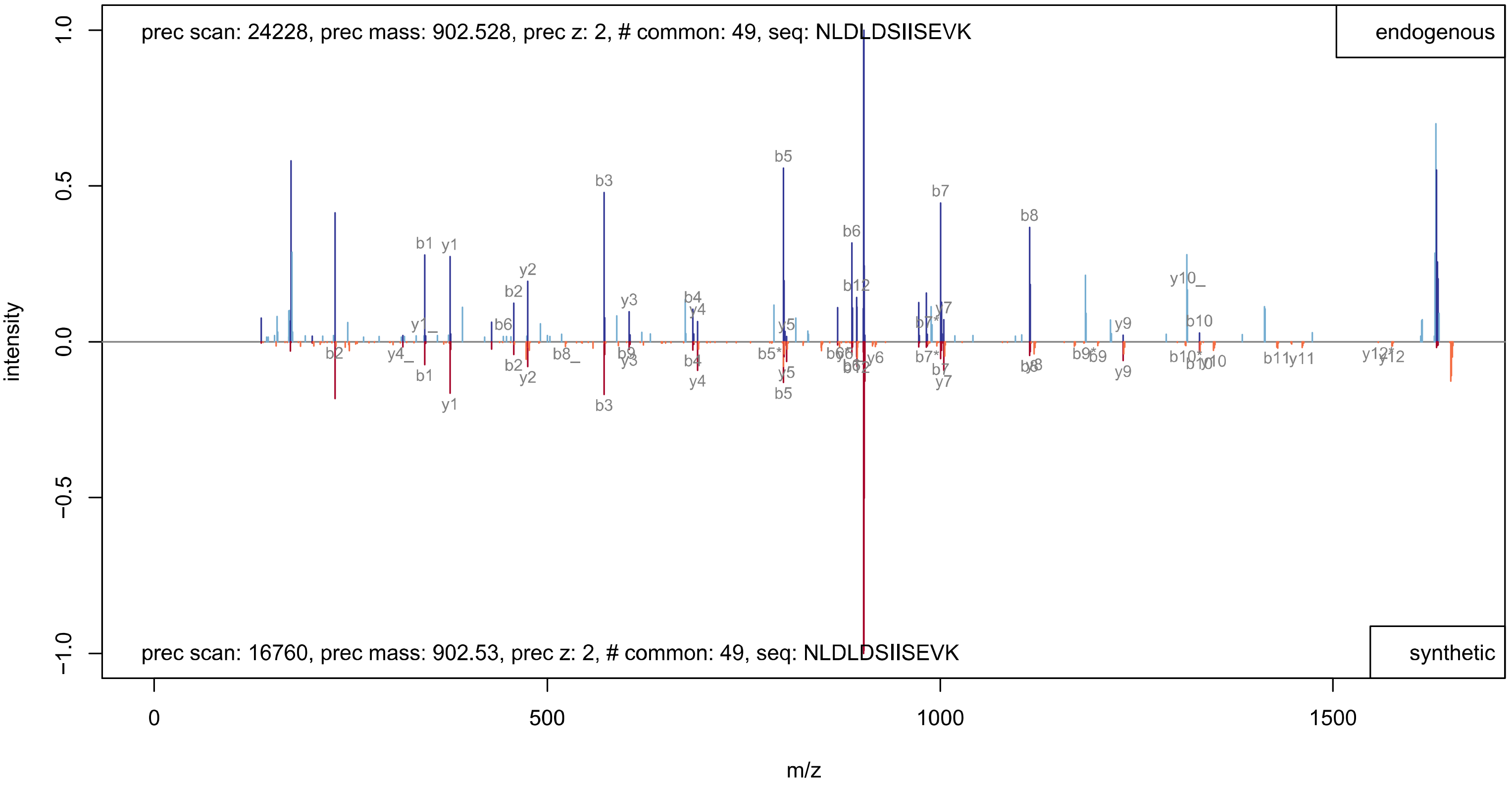

MTA1_V372I

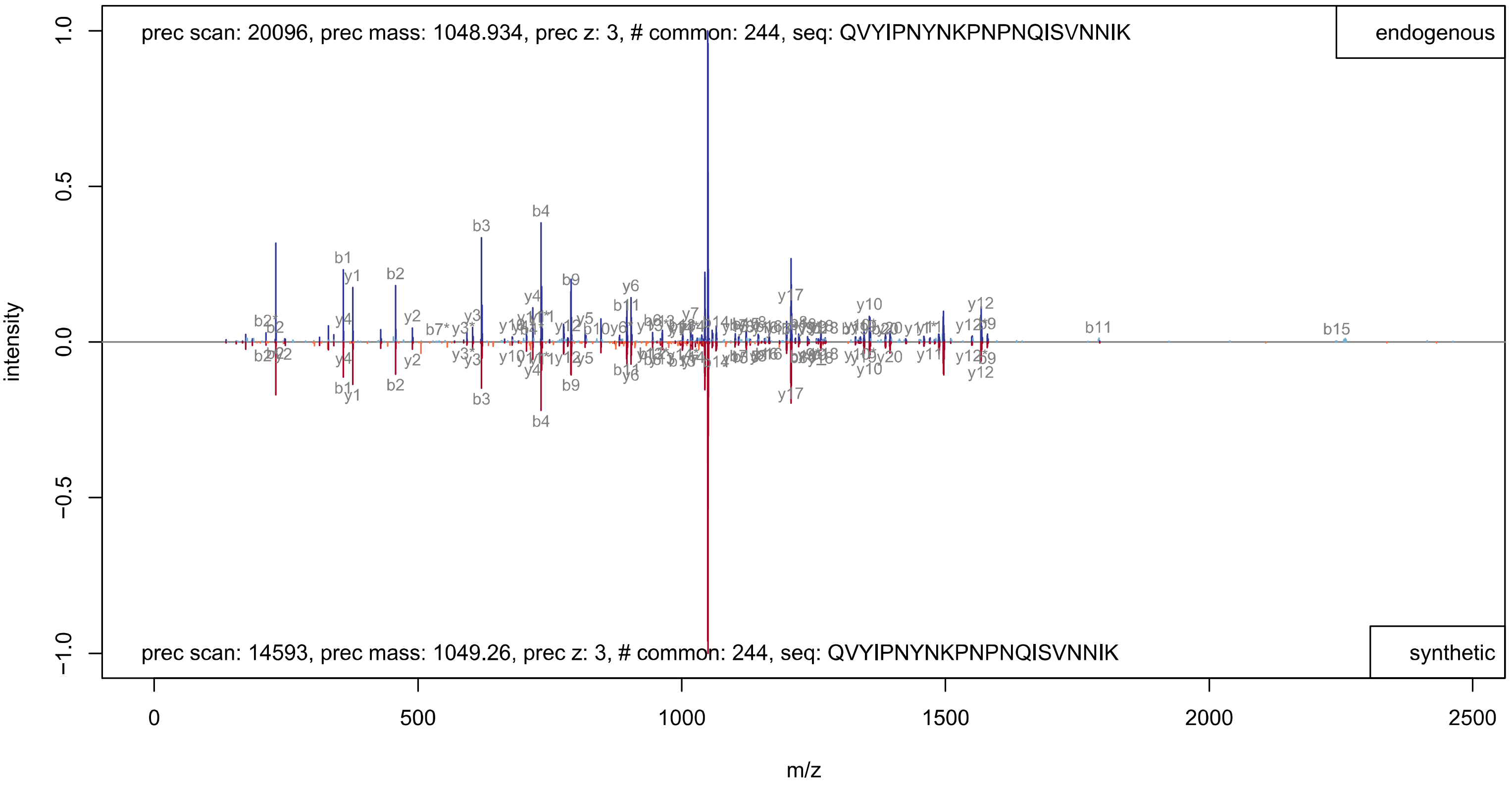

chr17_17041595

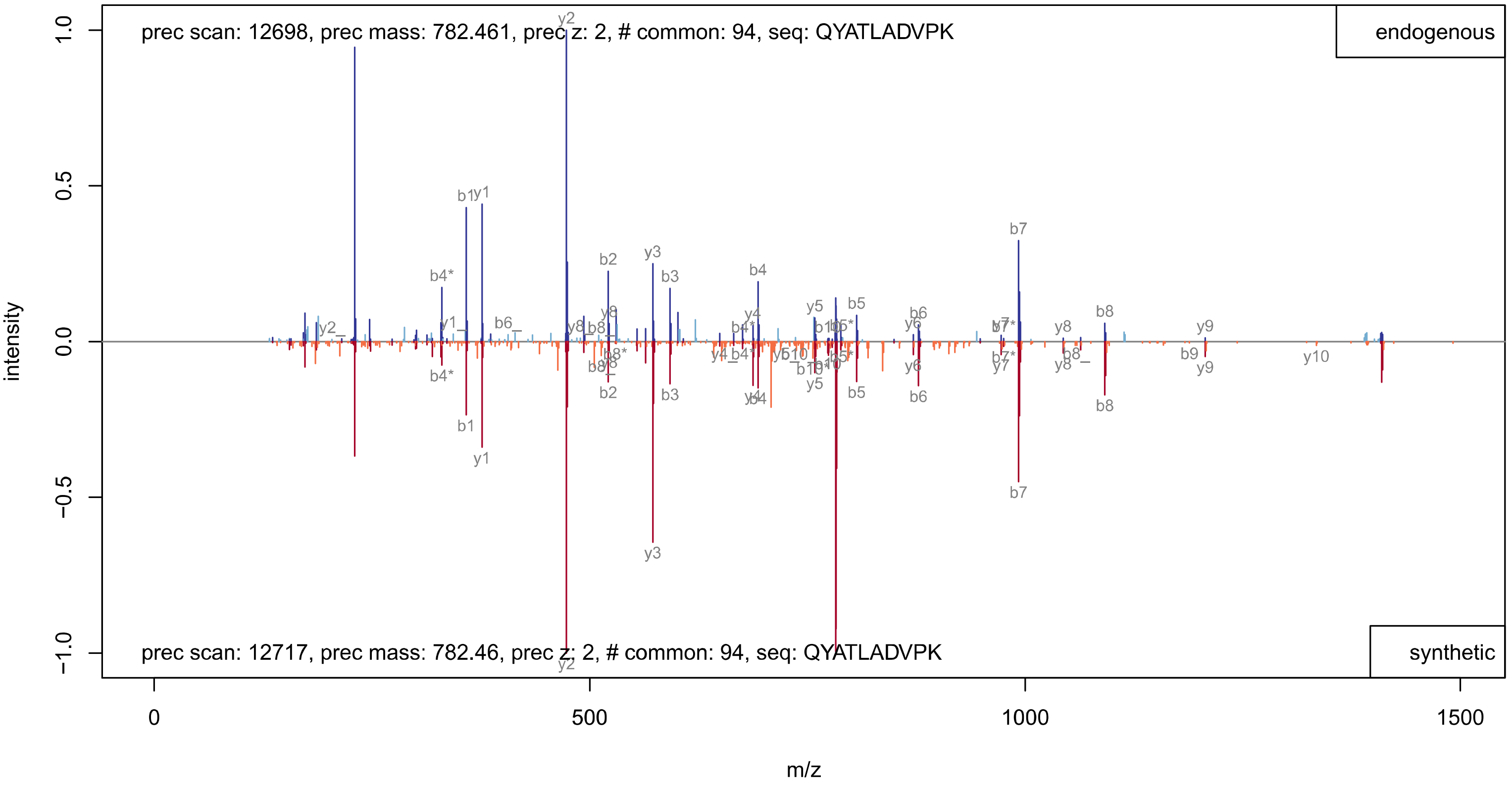

CENPF_R2729Q

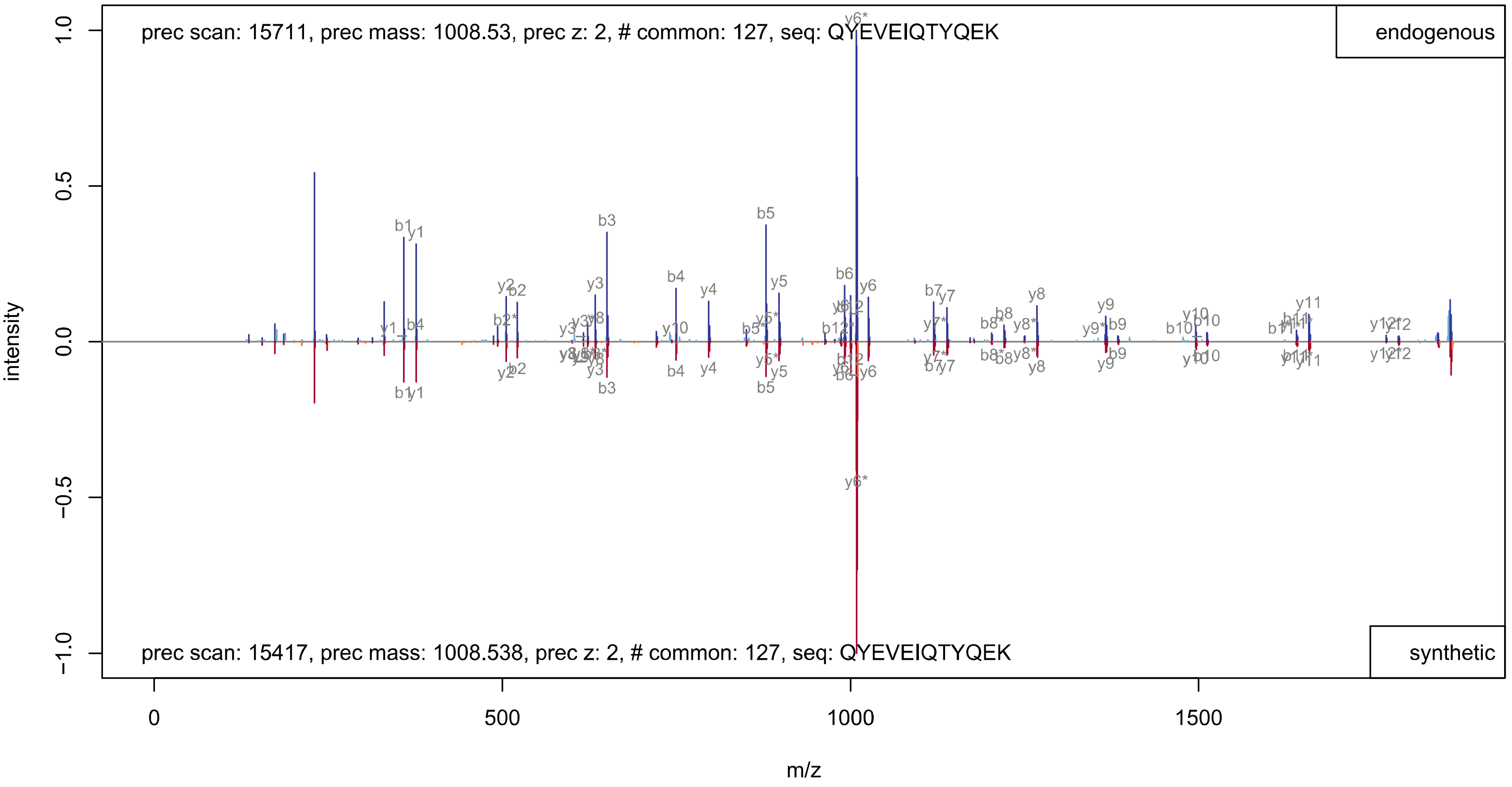

chr20_30135385

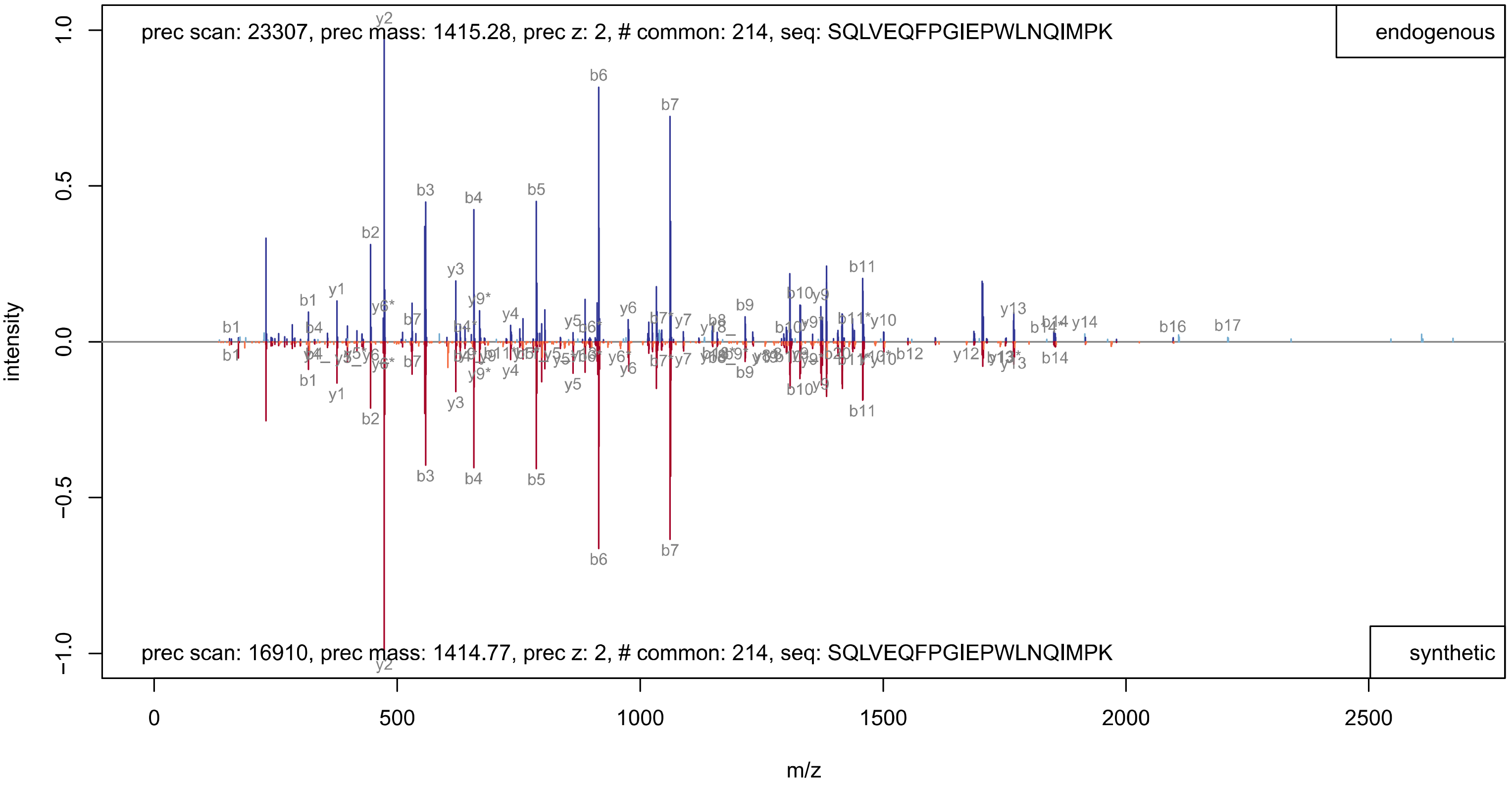

lnc−FUBP1−2:4

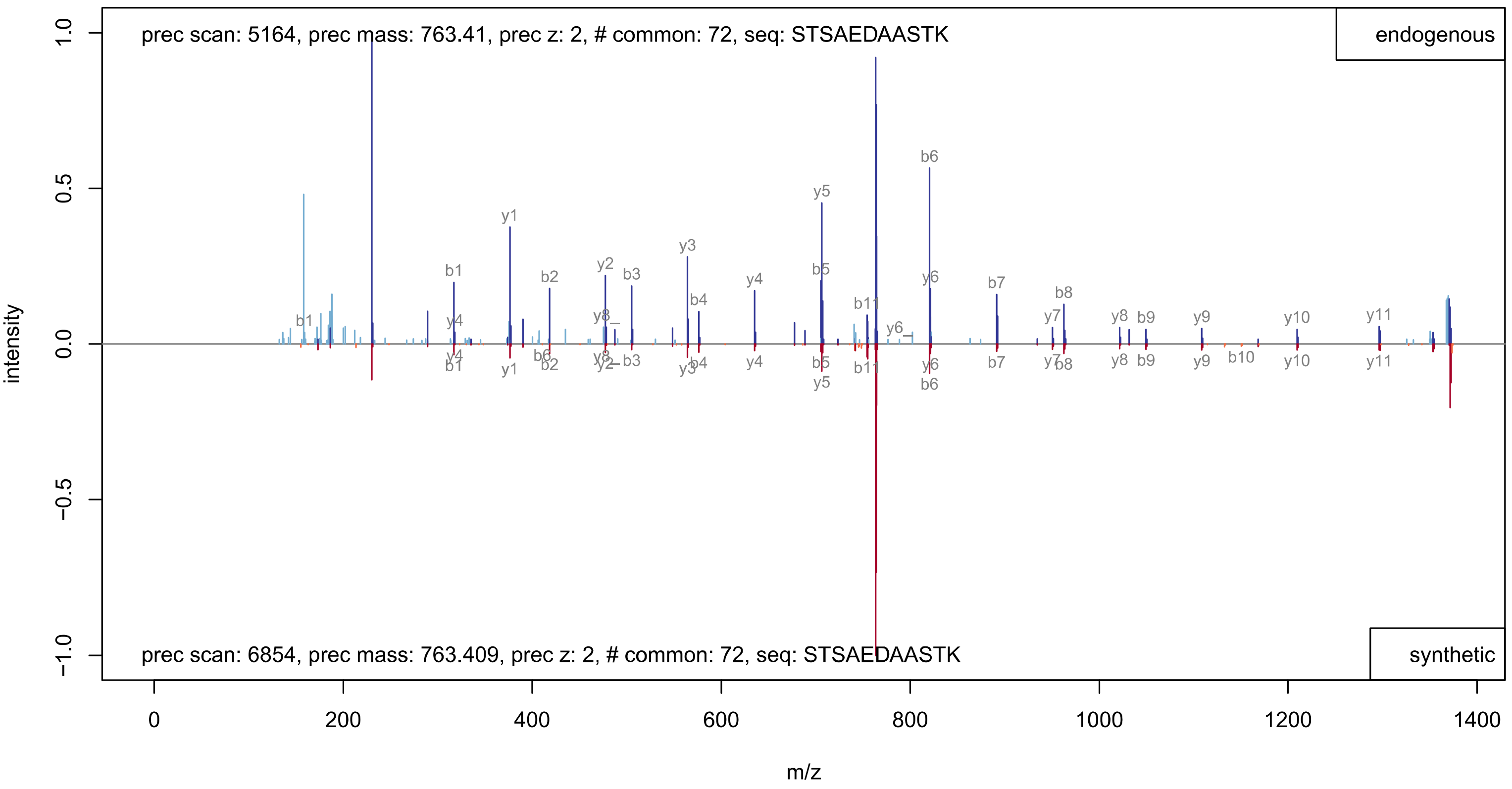

lnc−SIDT2−1:1

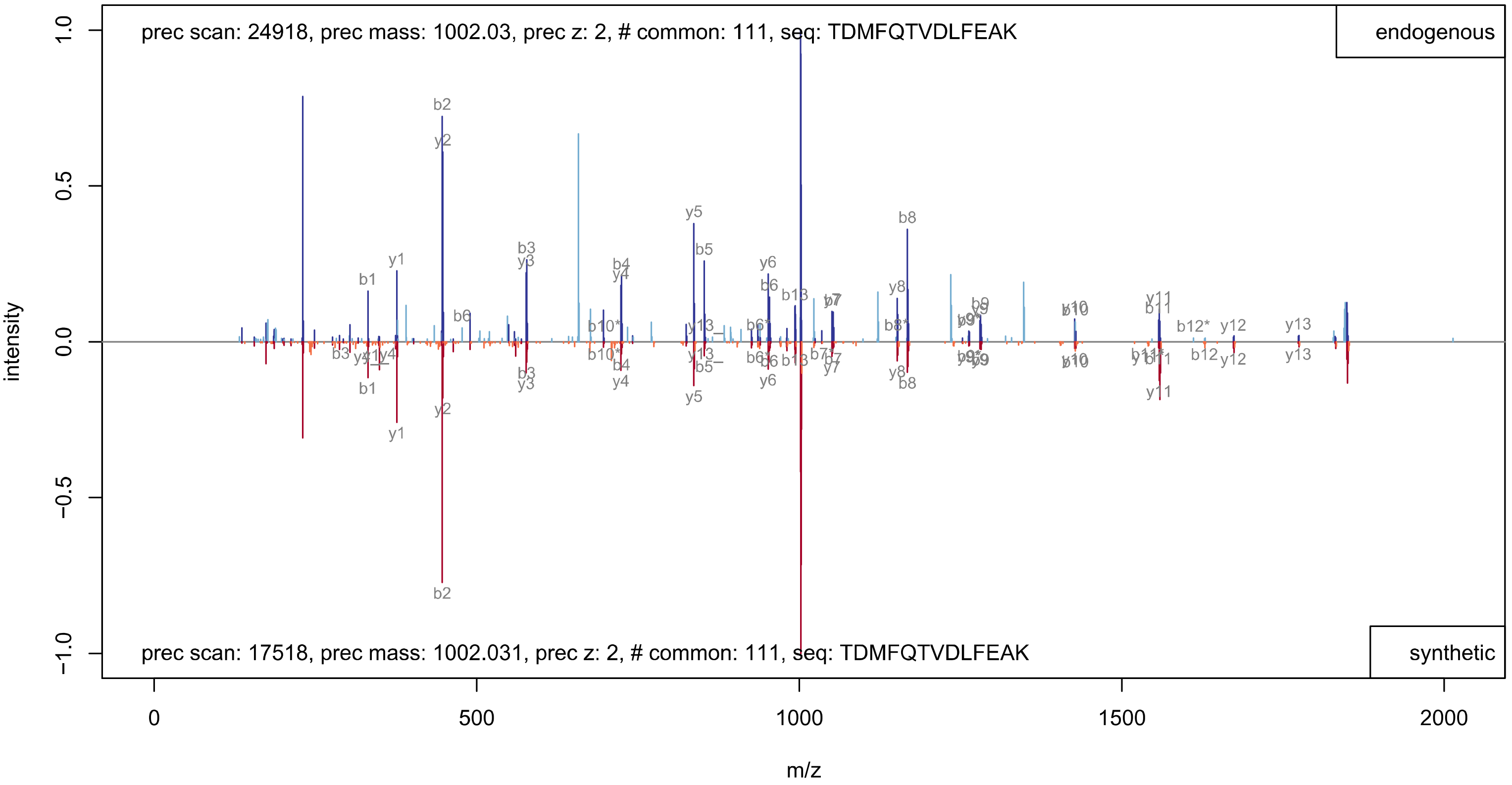

chr6_116579733

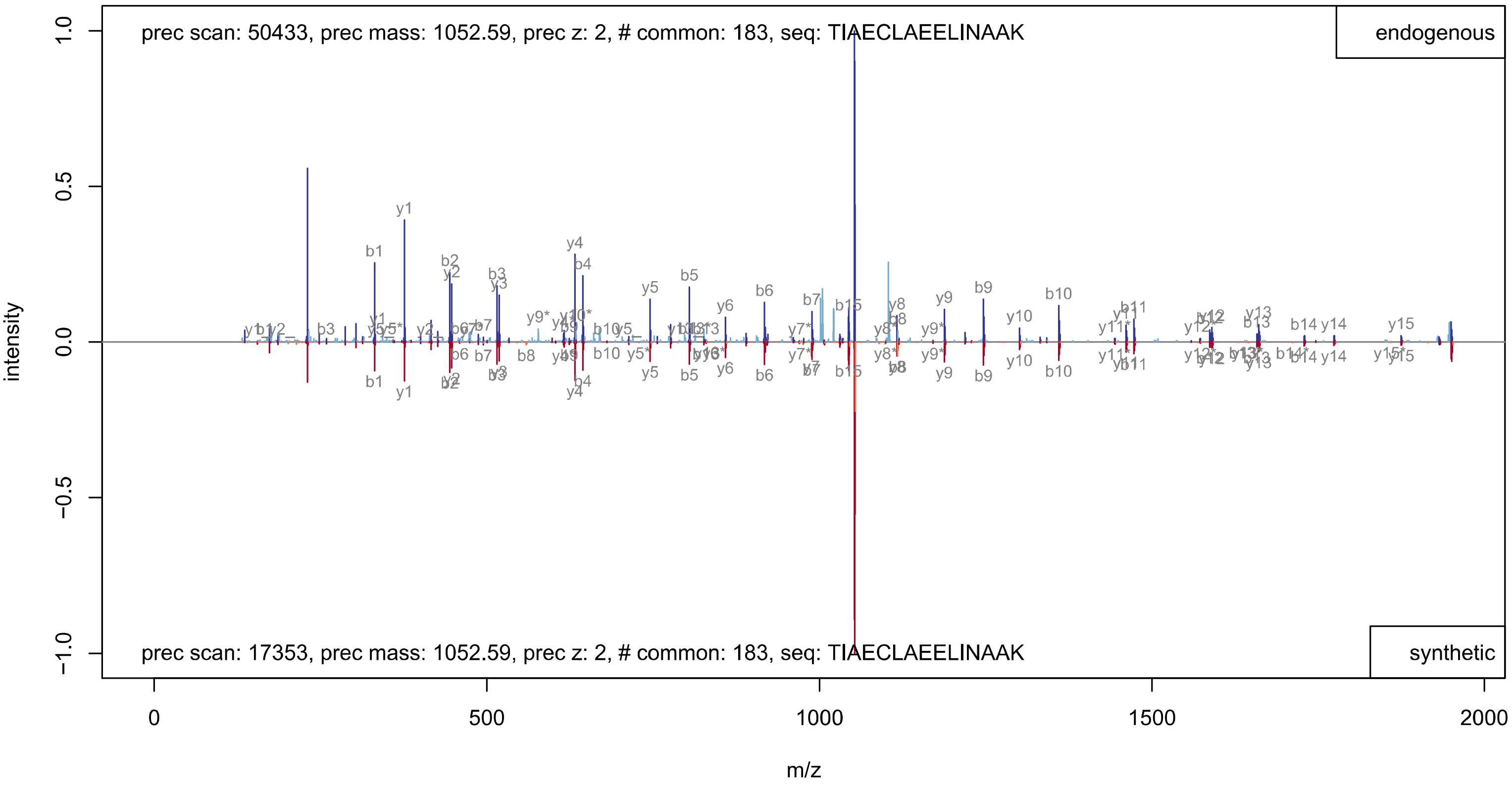

KRT17_D417N

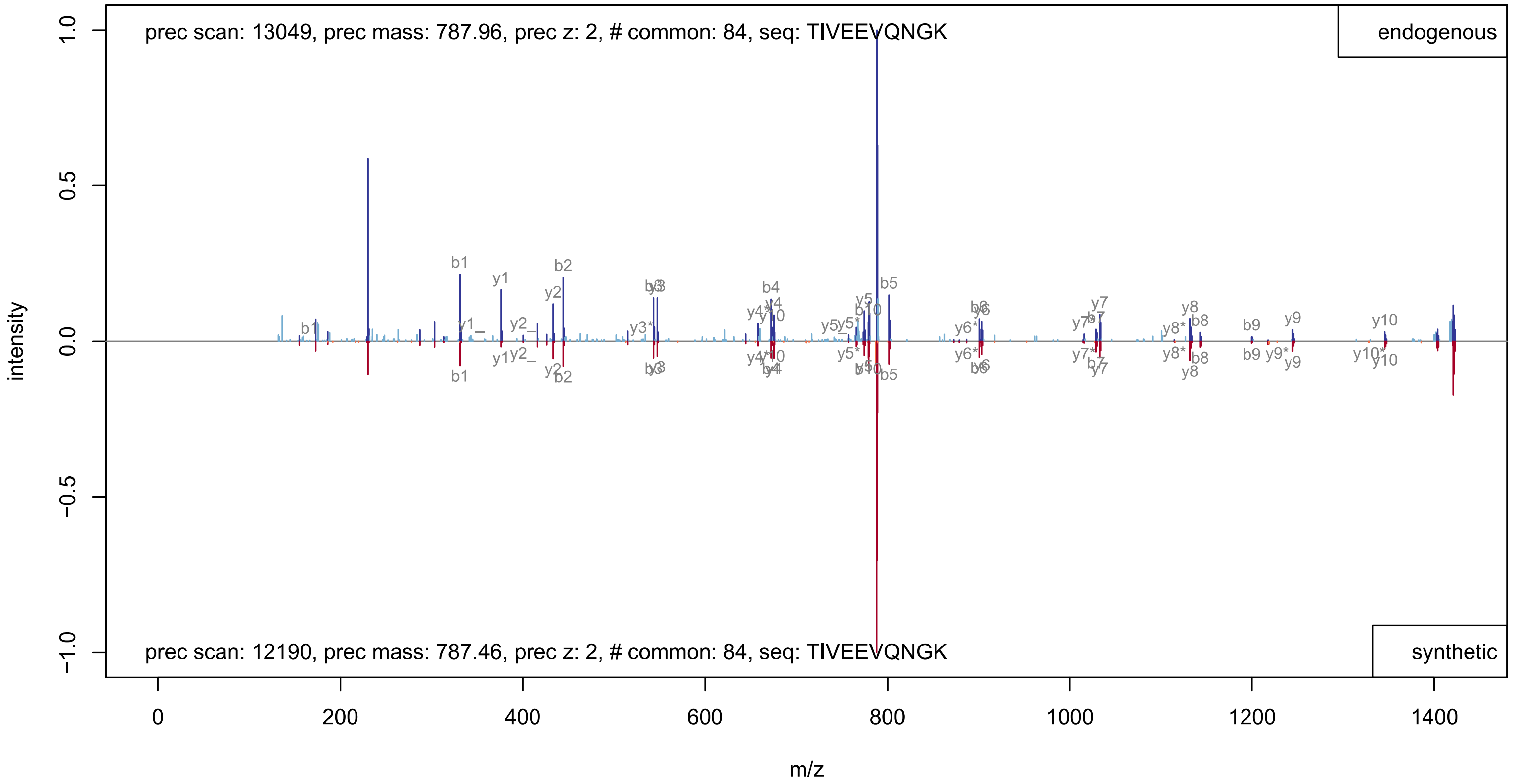

ERBB2_P8T

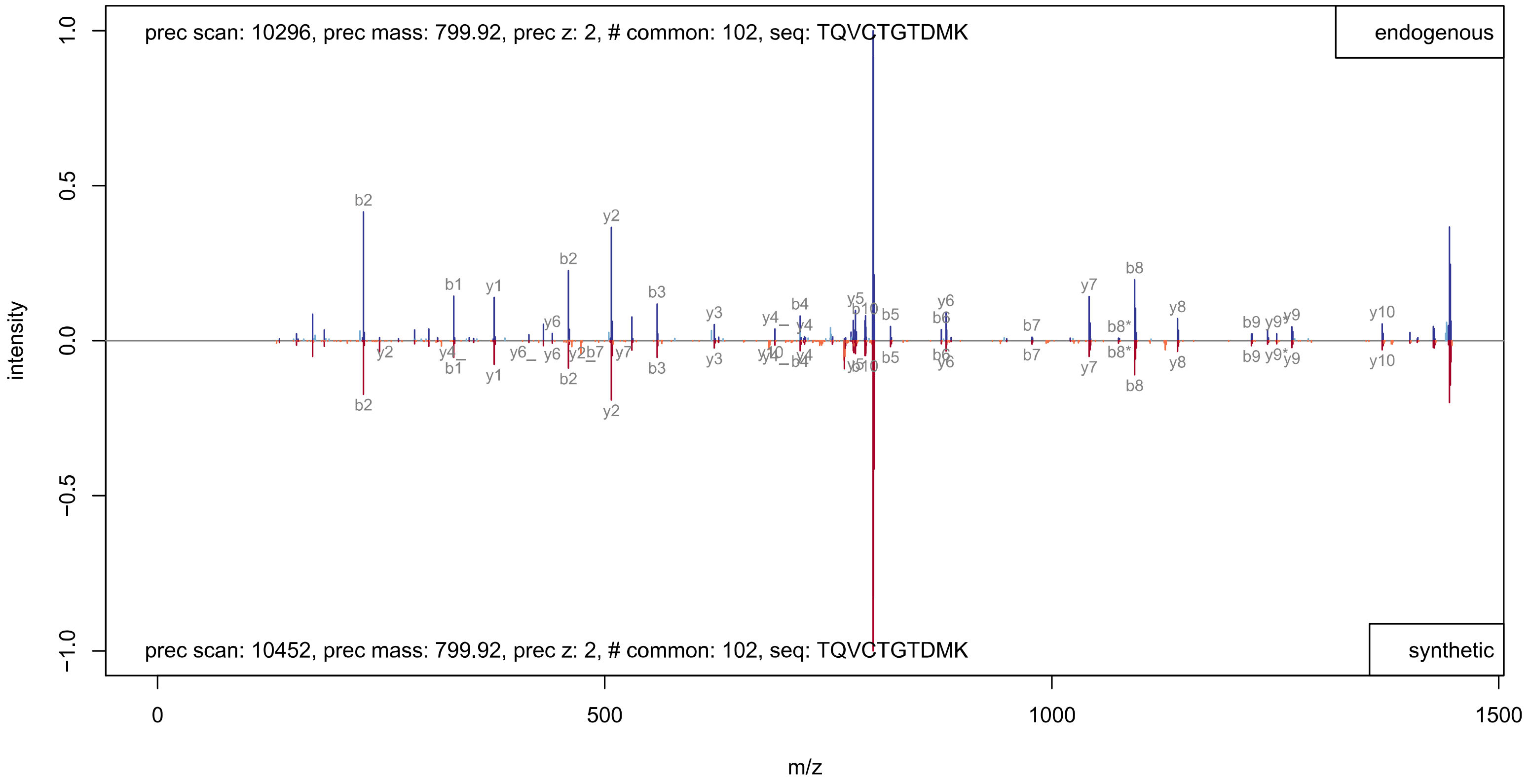

chrX_47078379

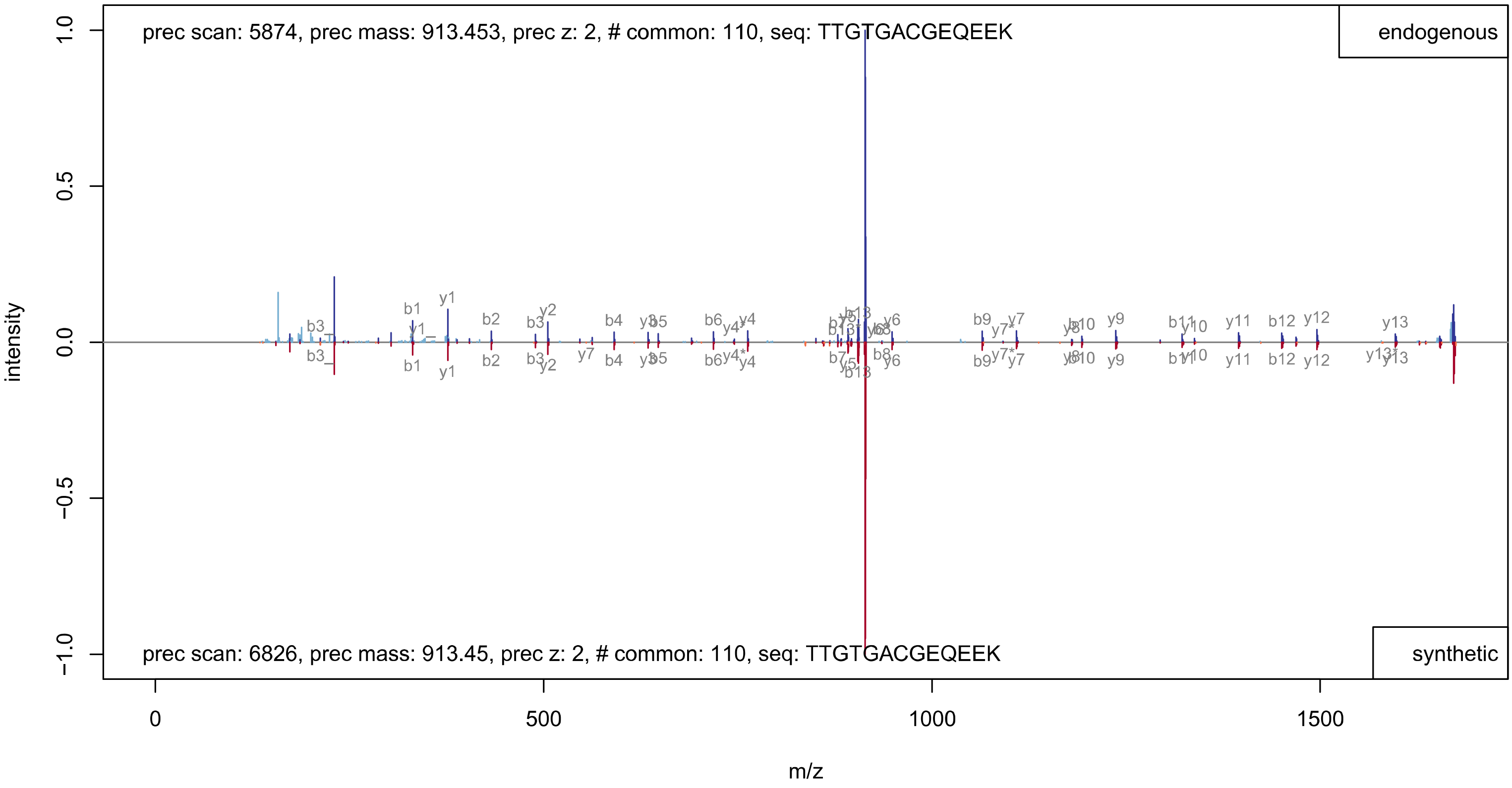

PGOHUM_ENST00000405405.2_GAPDHP63

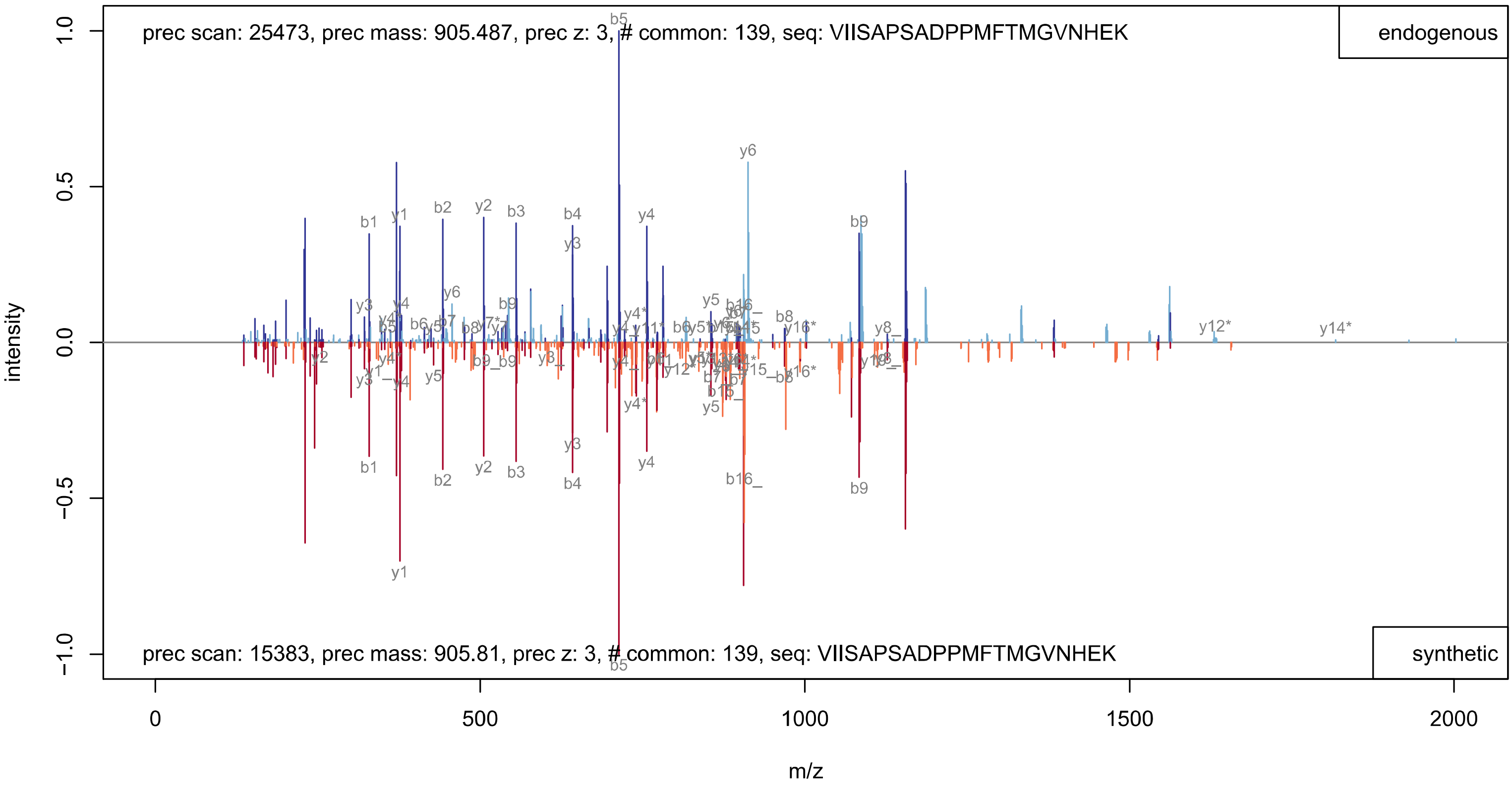

CDH11_ENST00000394156_T255M

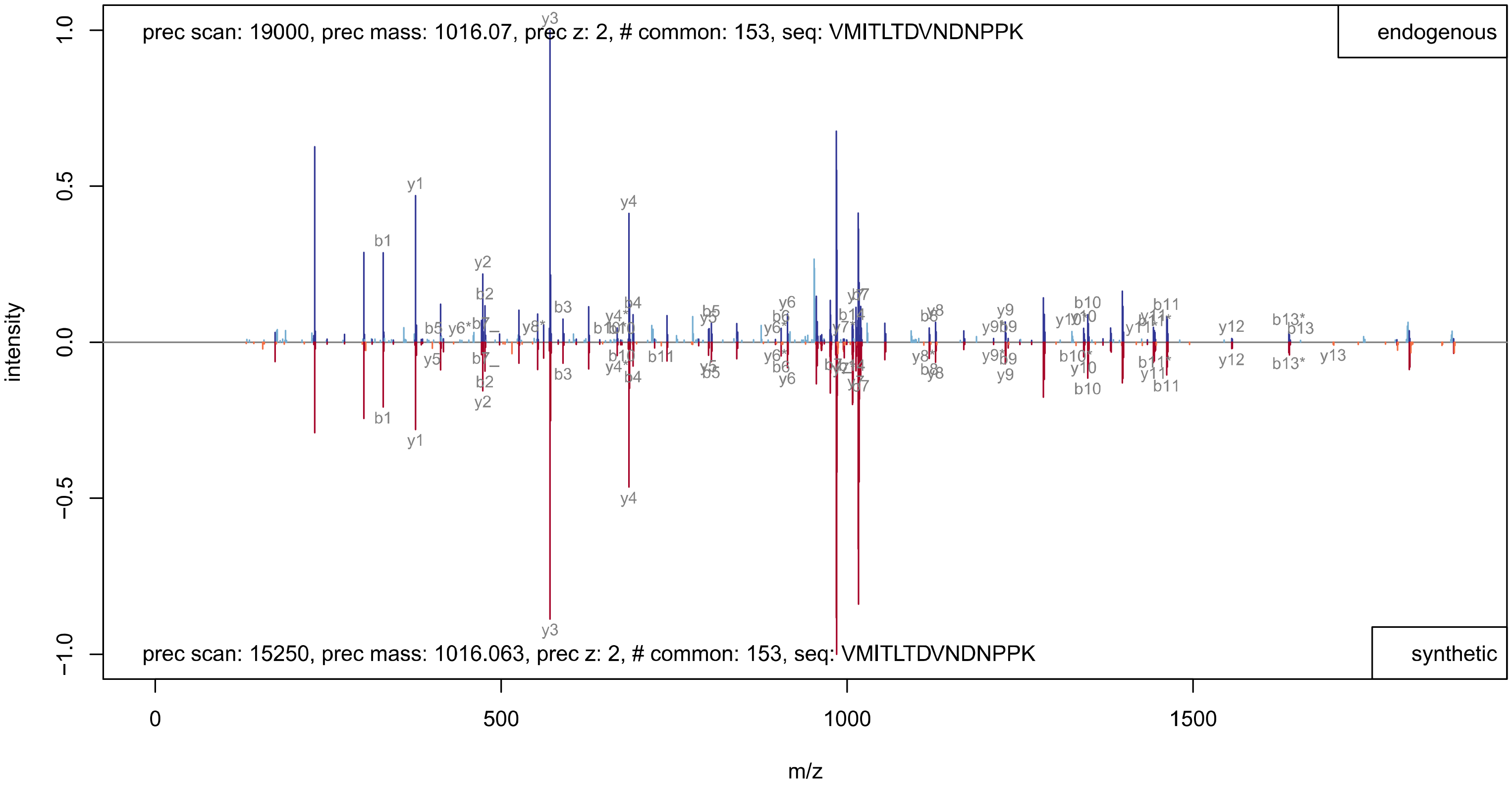

chr19_45737990

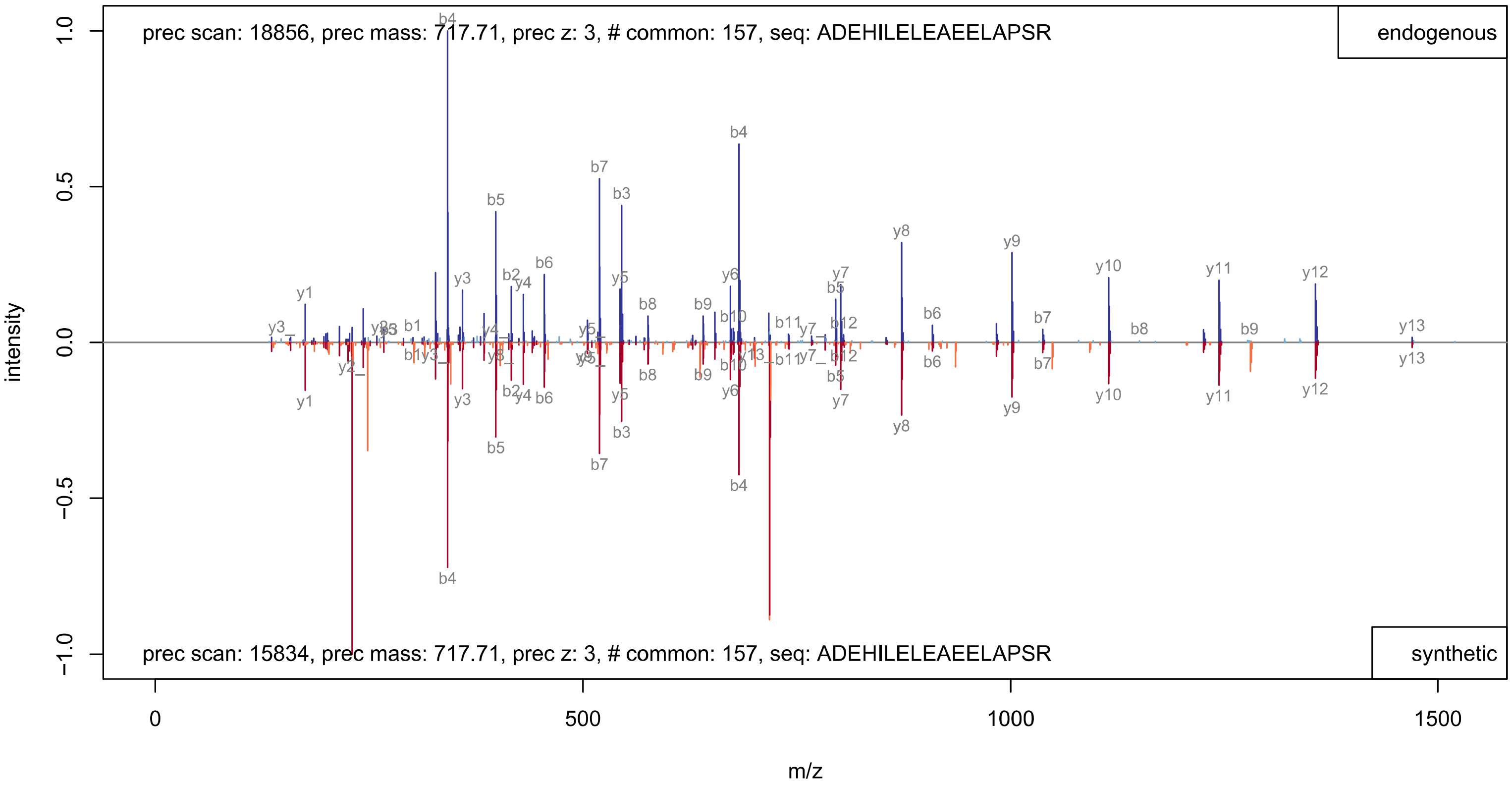

HNRNPA2B1.new.Nterm

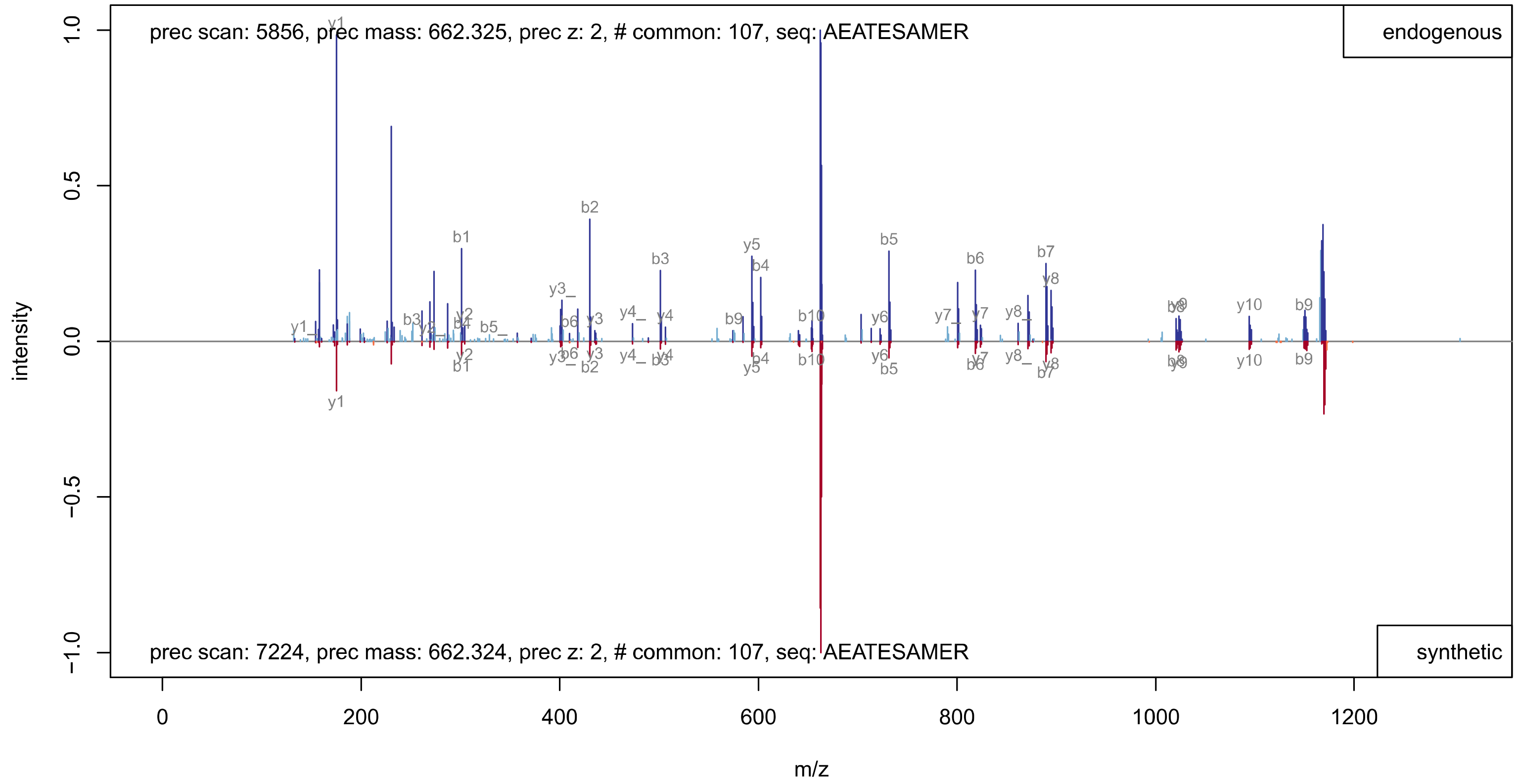

chr7_100802448

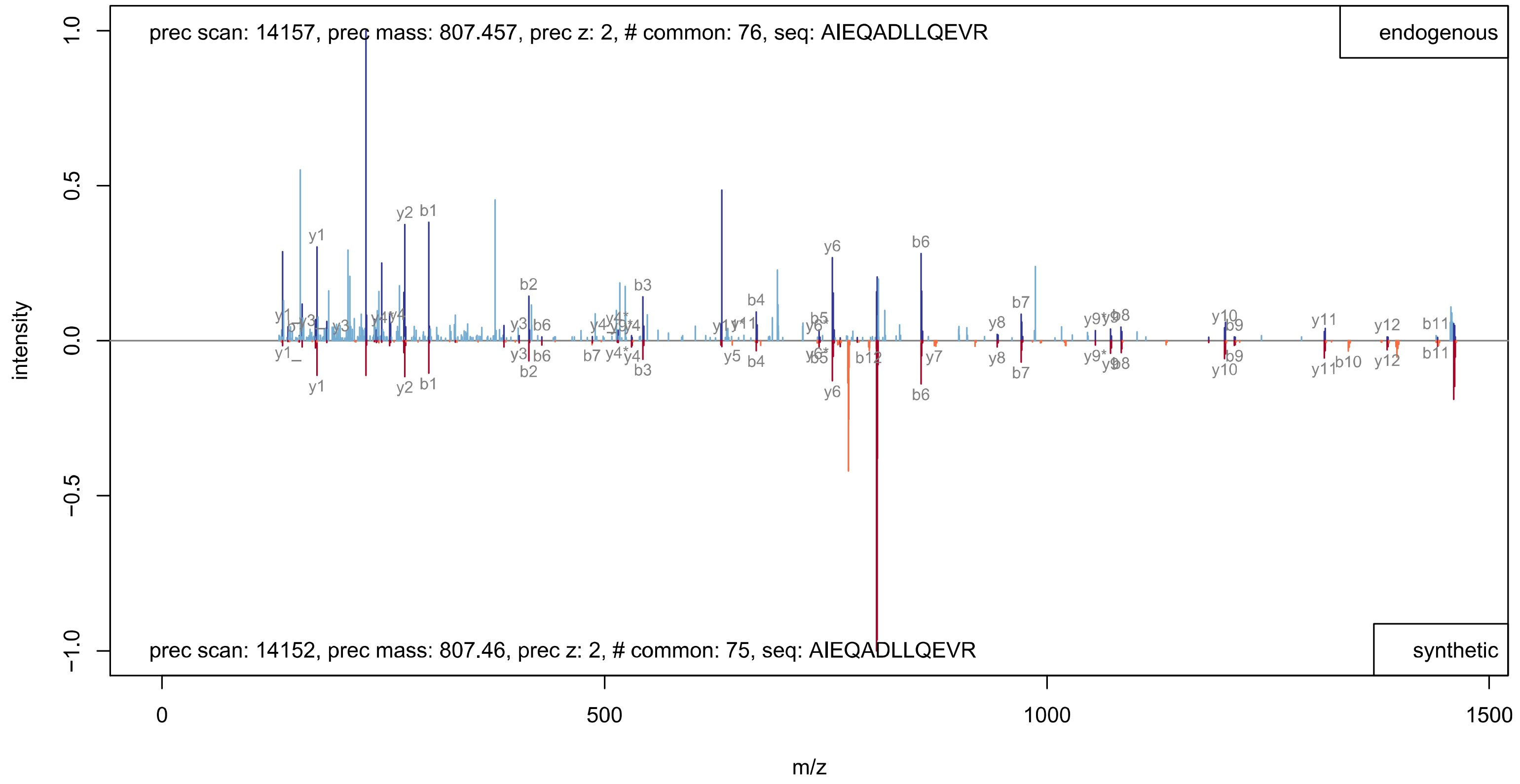

MMP11_A38V

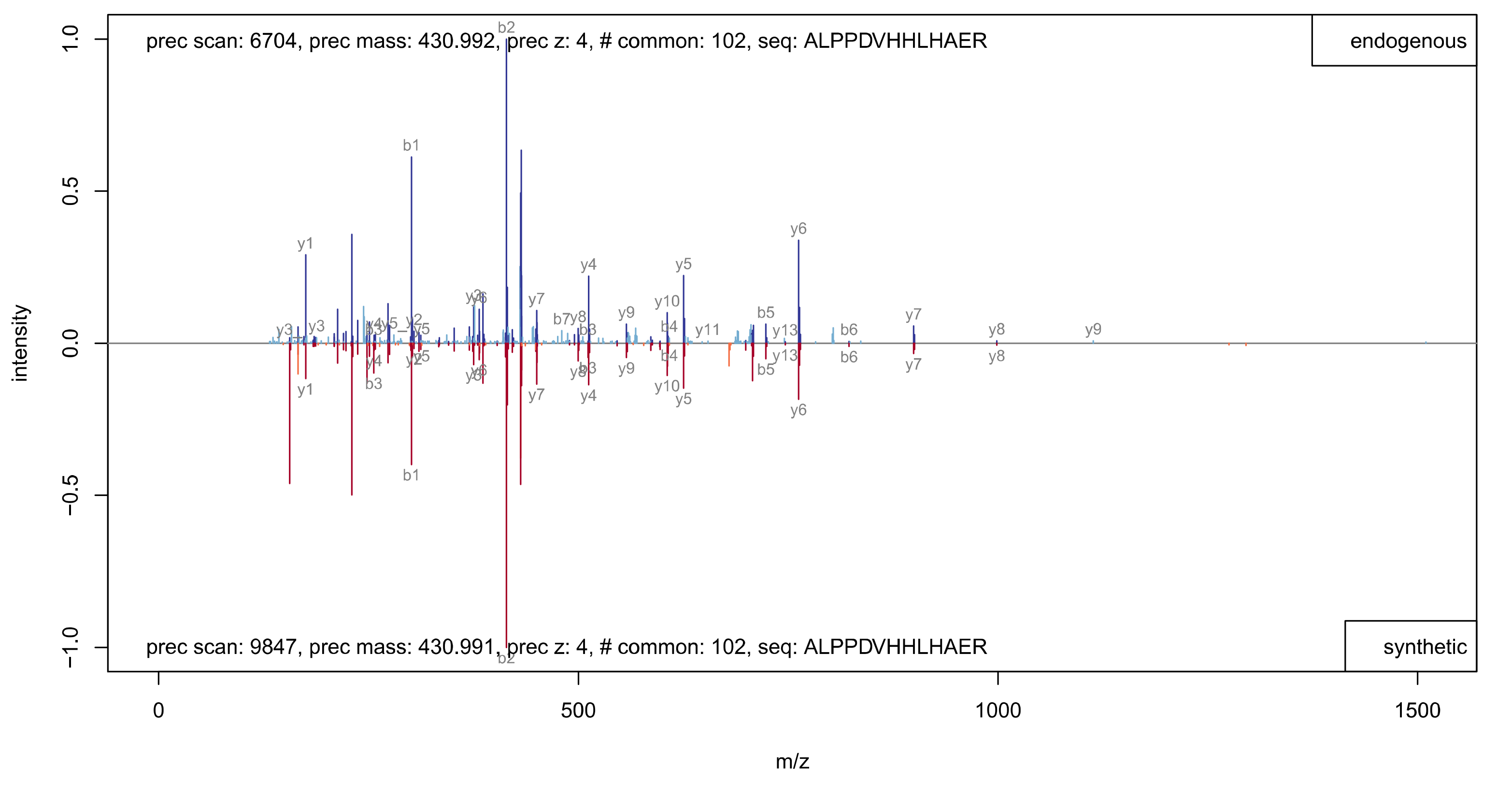

chr17_20674603

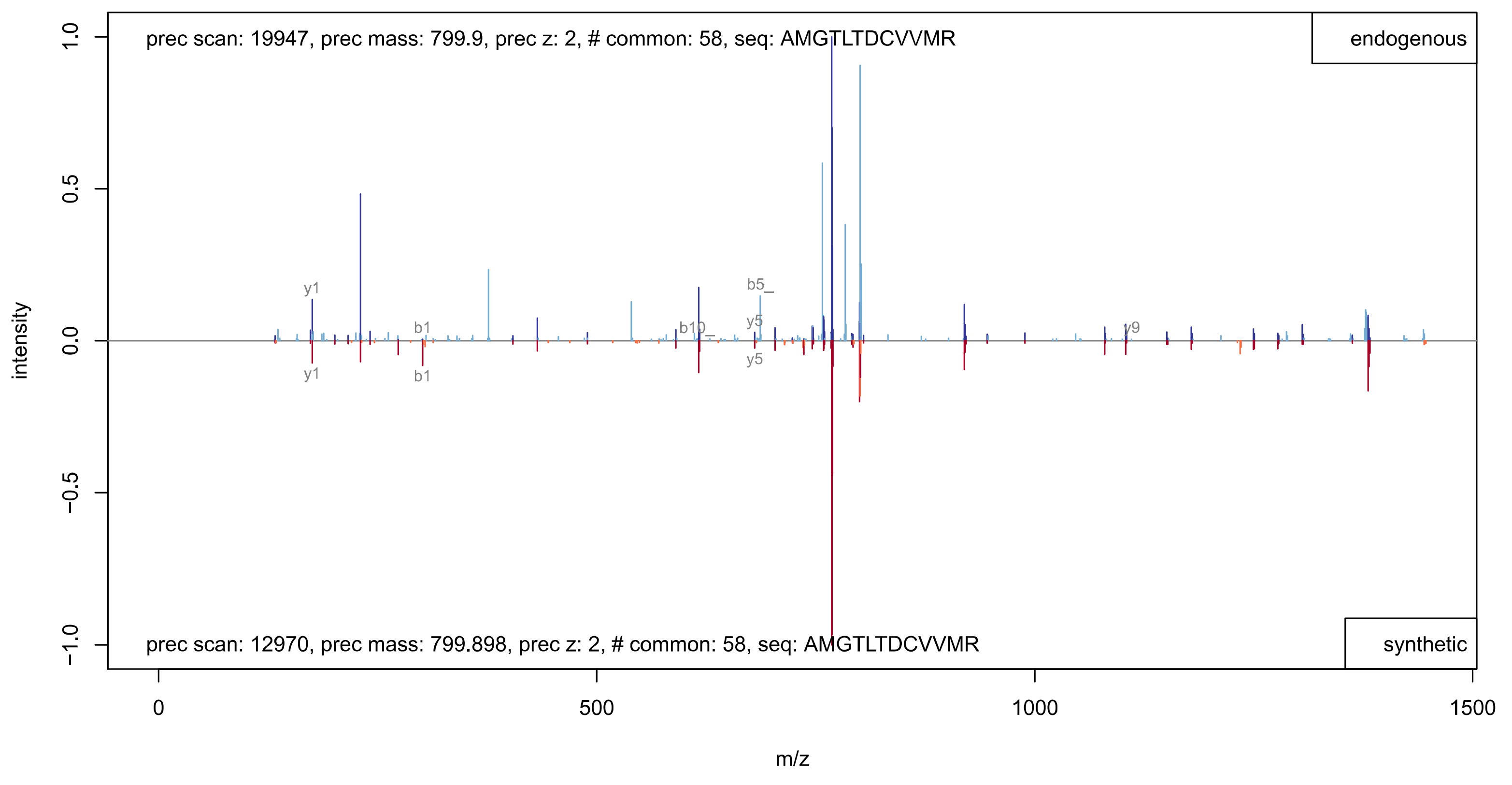

chr1_224621964

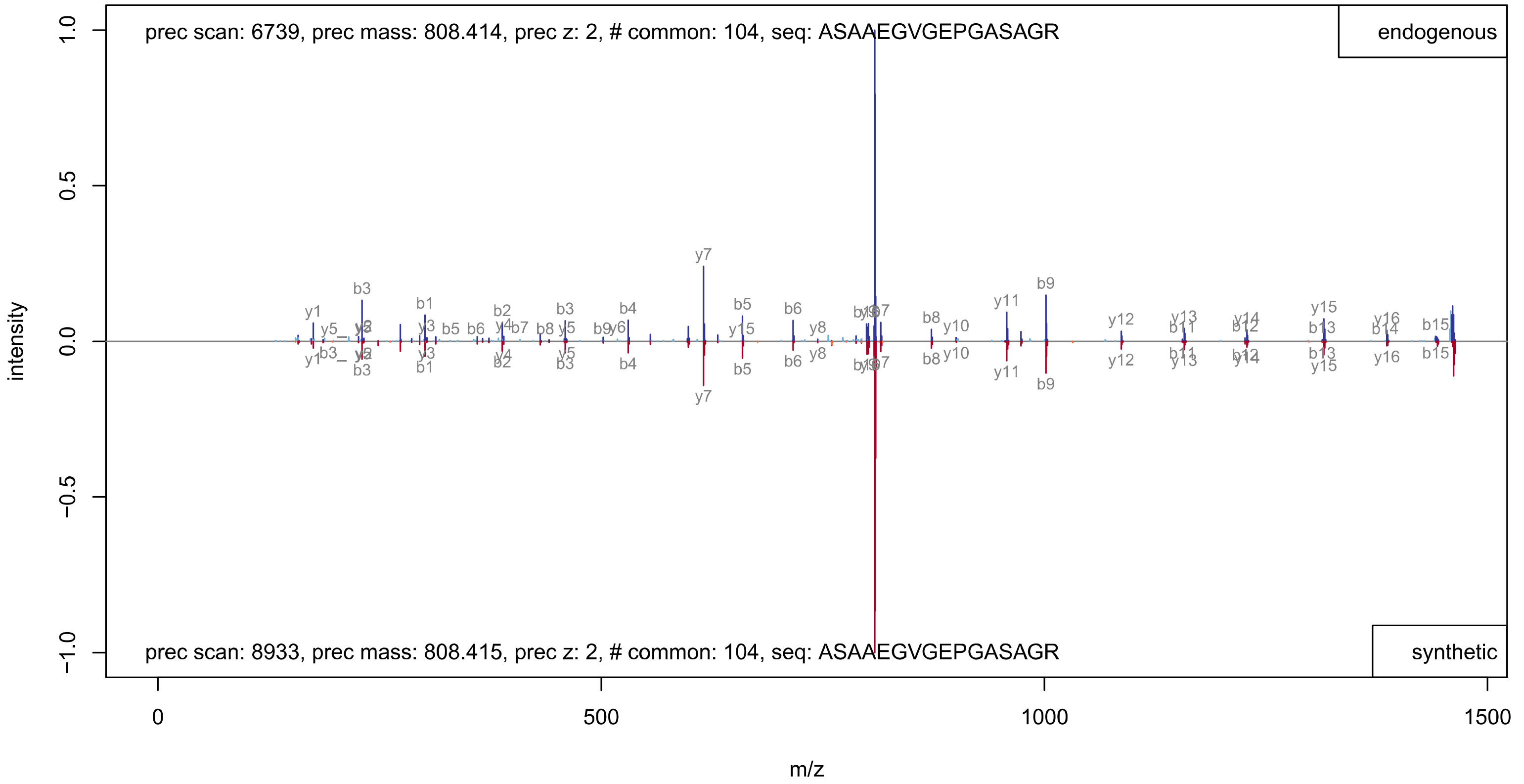

chr10_8556078

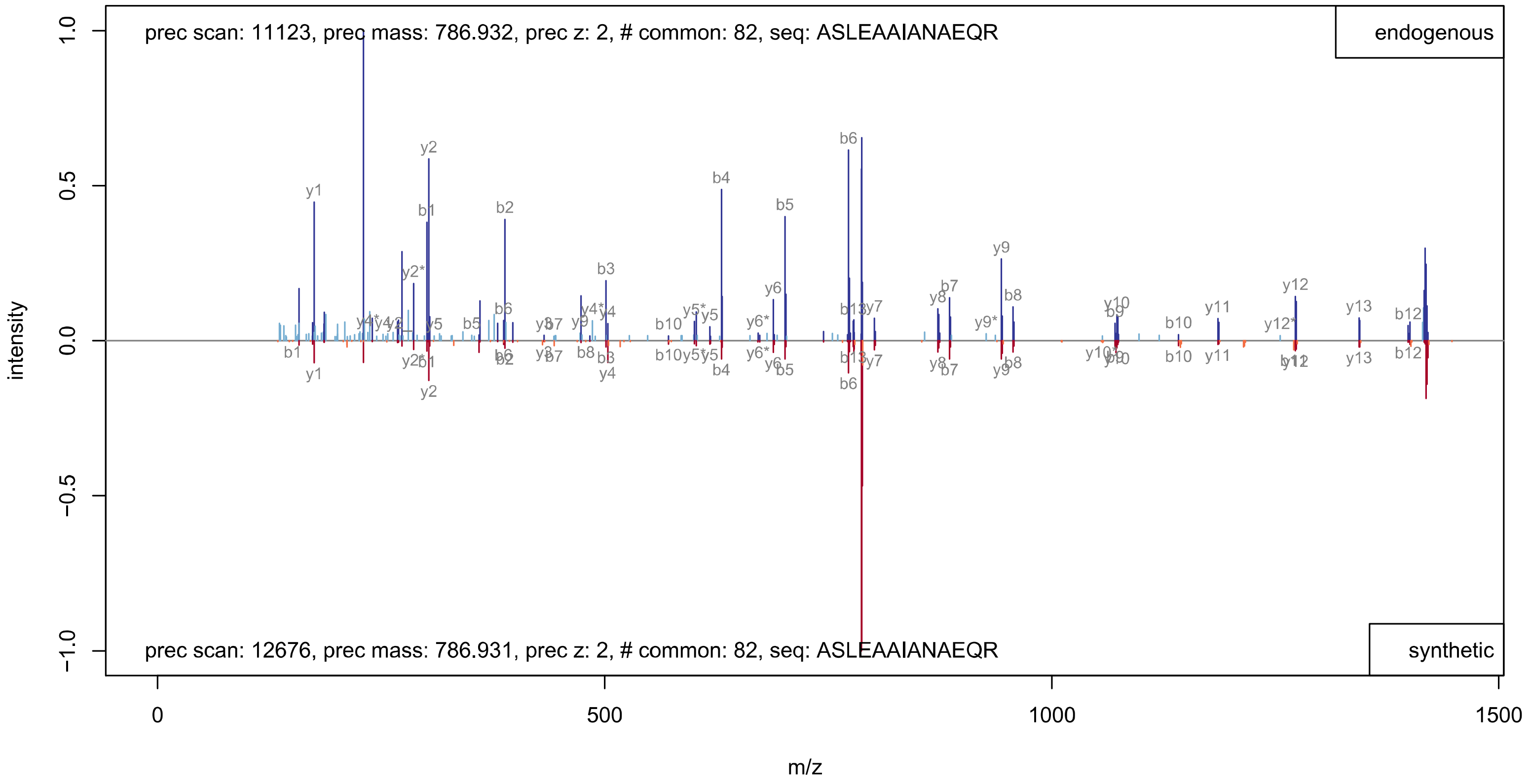

chr11_803527

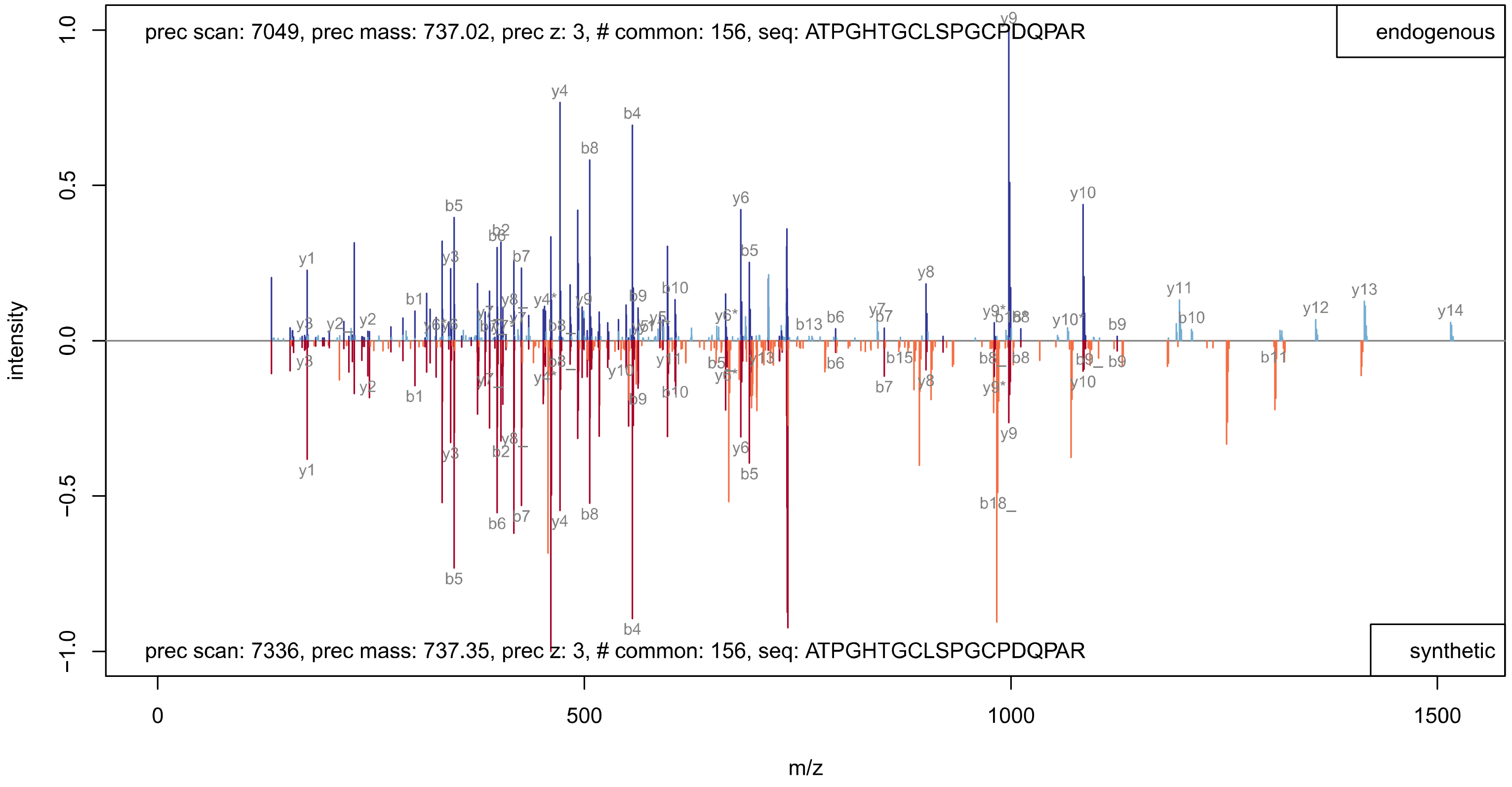

chr11_65686972

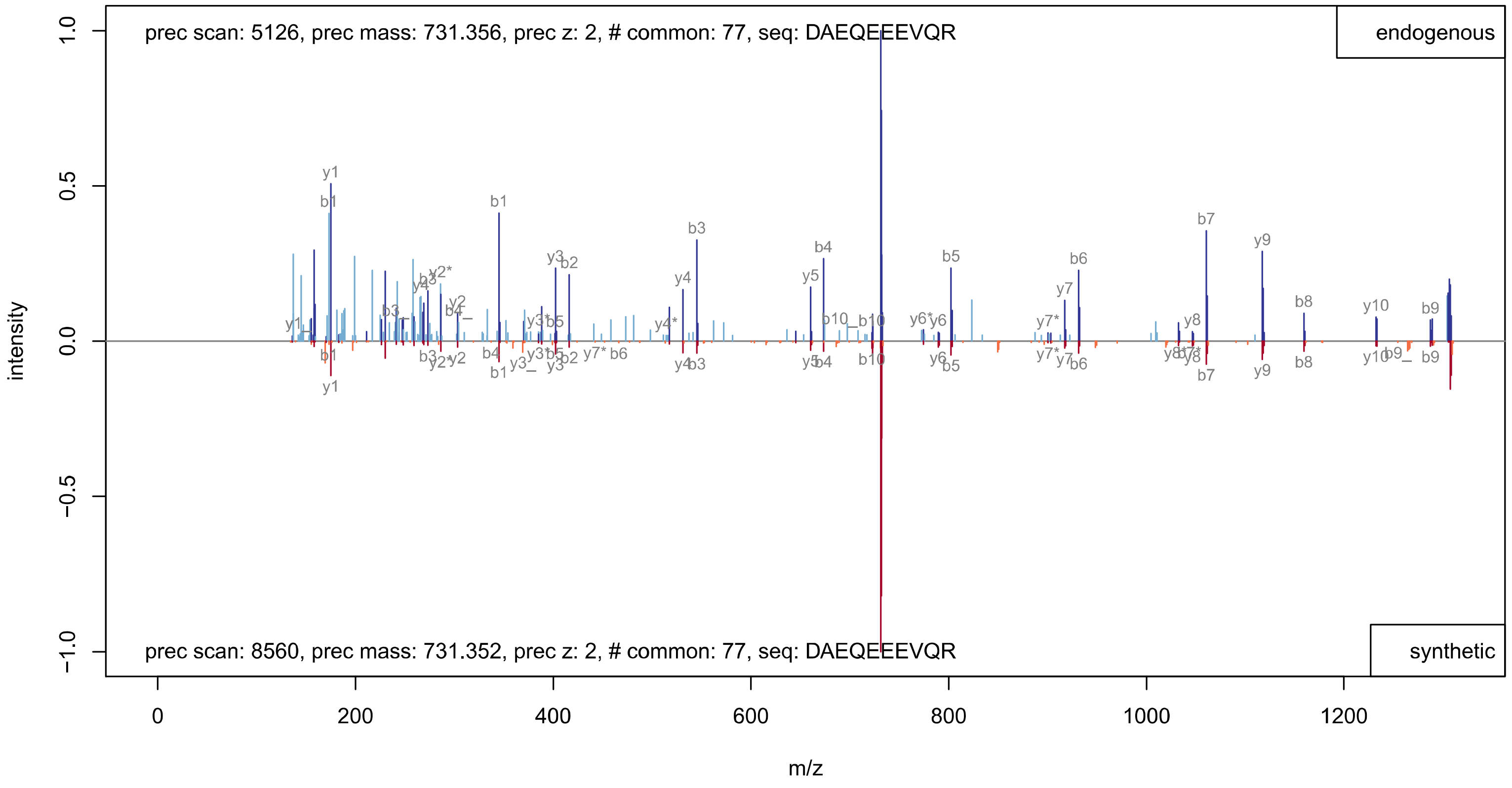

PGOHUM_ENST00000426373.1_RP11−108B14.4

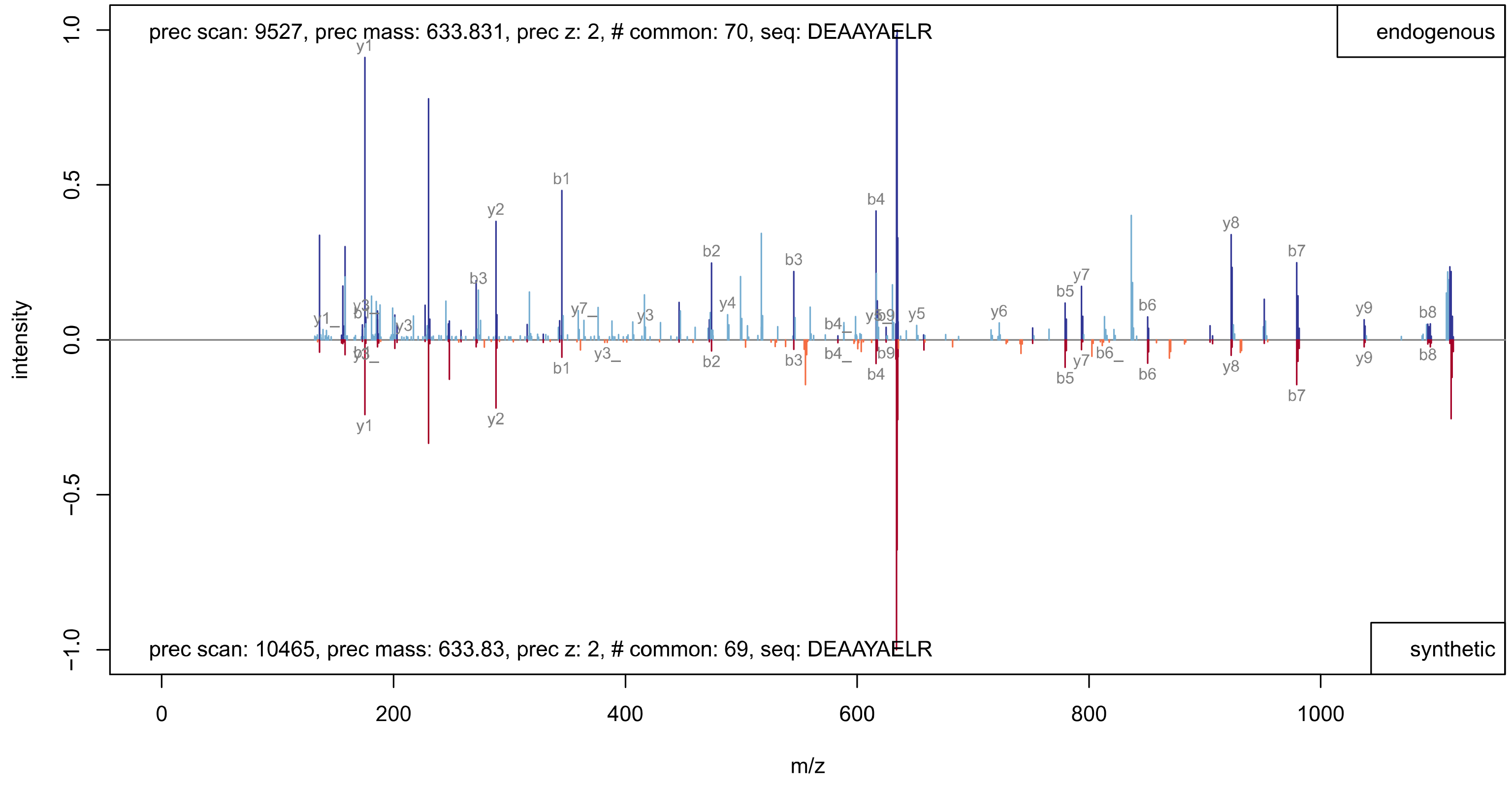

MYBBP1A_Q8E

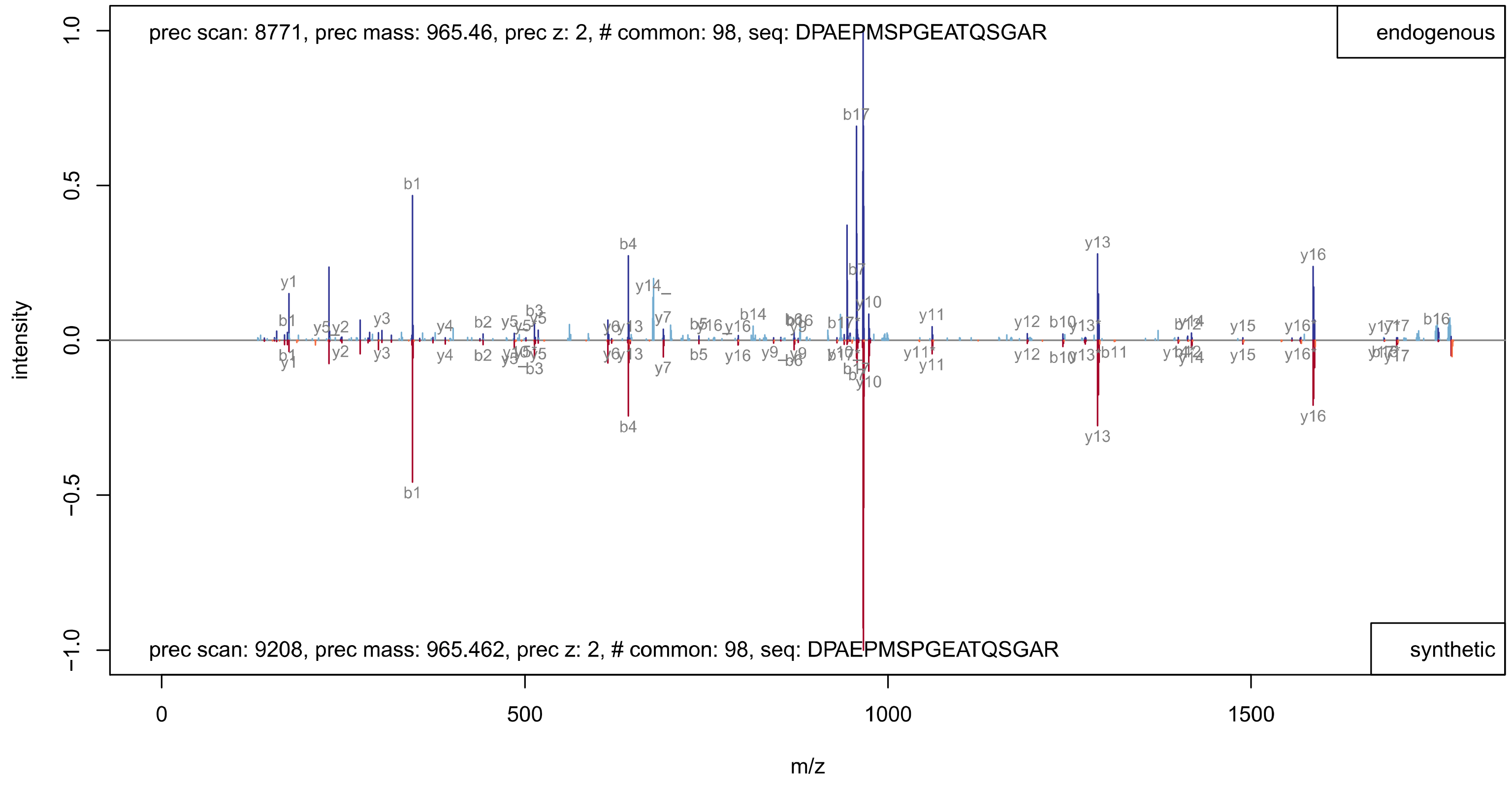

MYBBP1A_Q8E

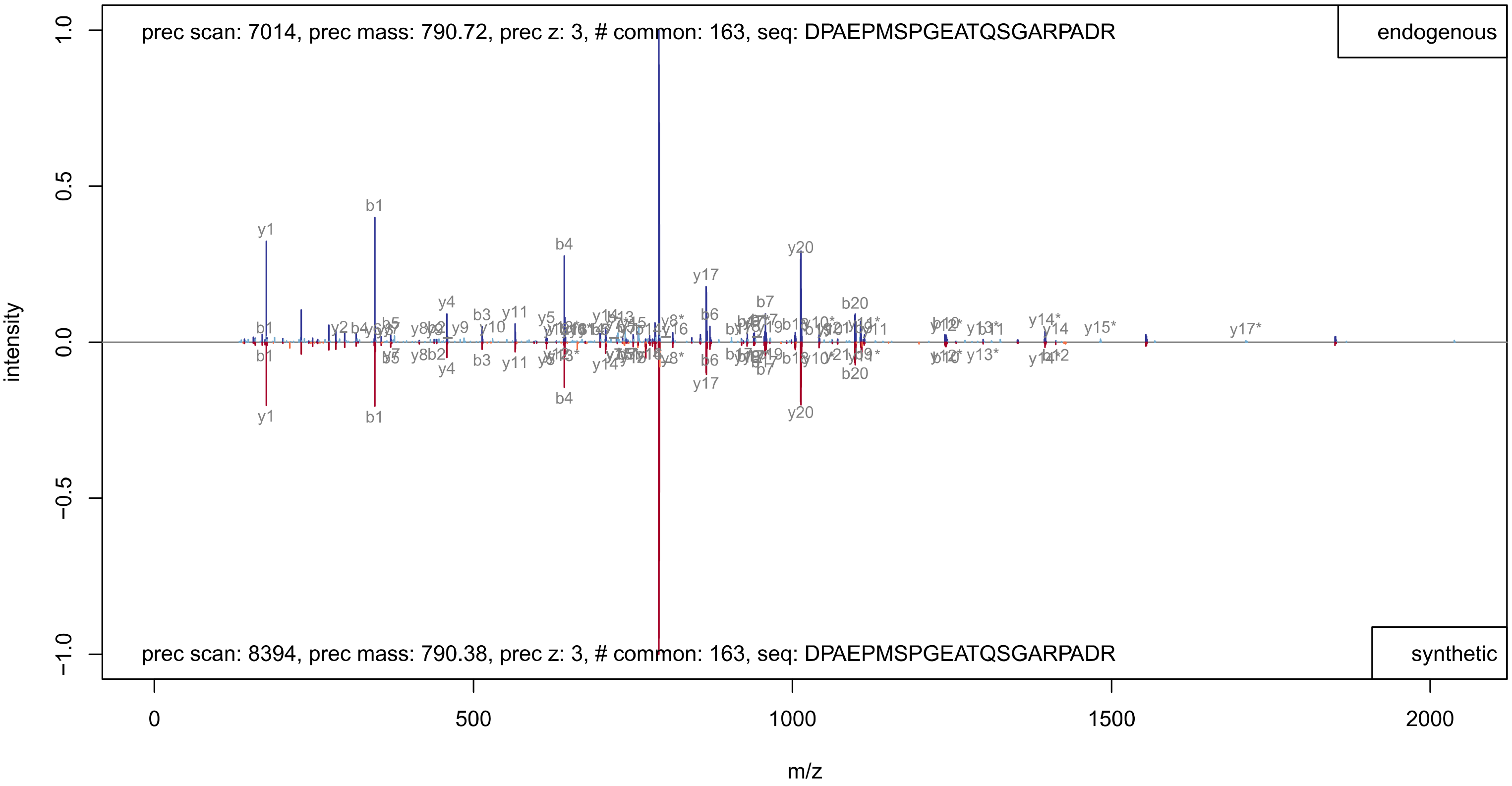

NAT1_ENST00000545197_V211I

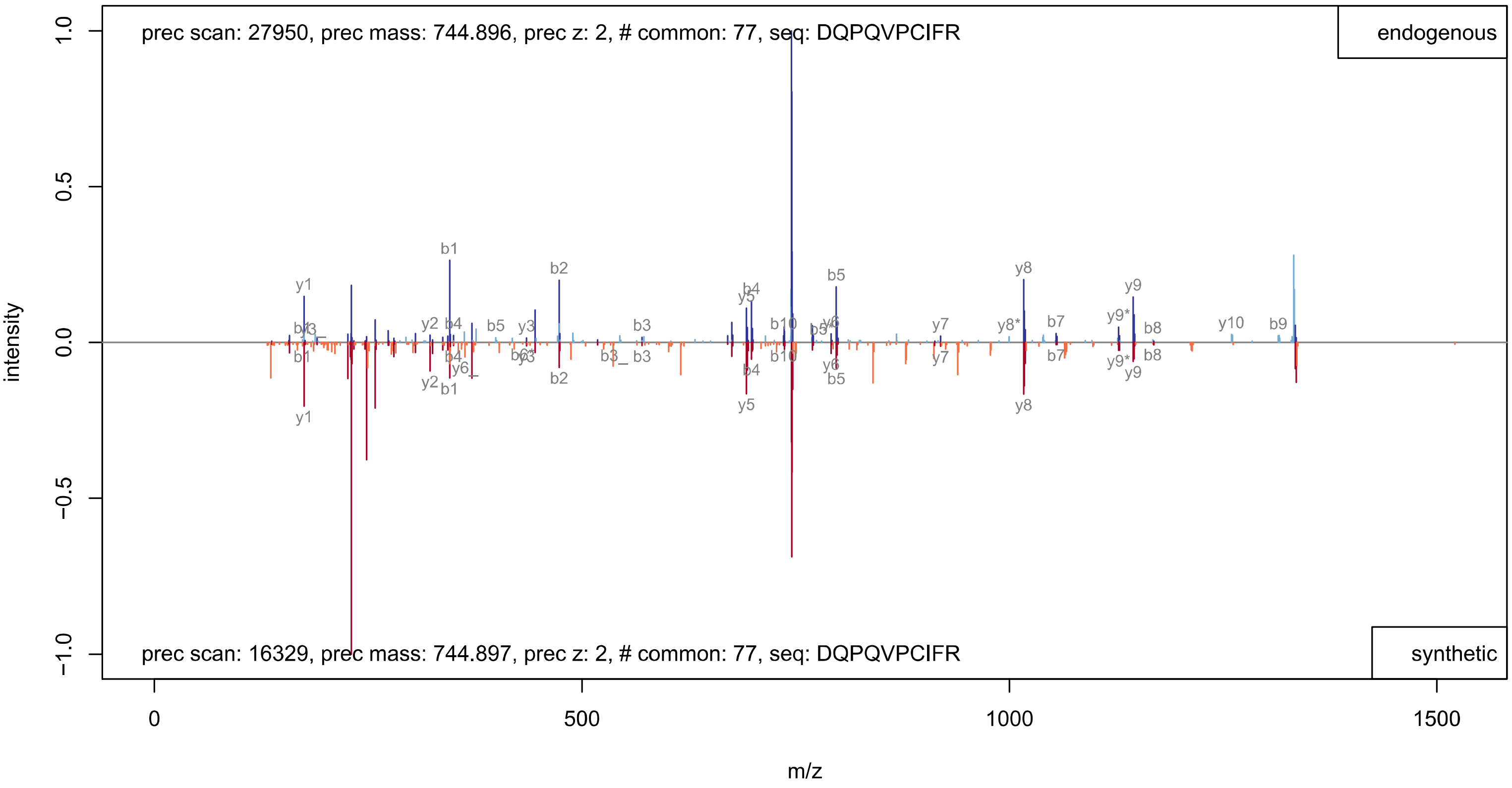

chr6_32055308

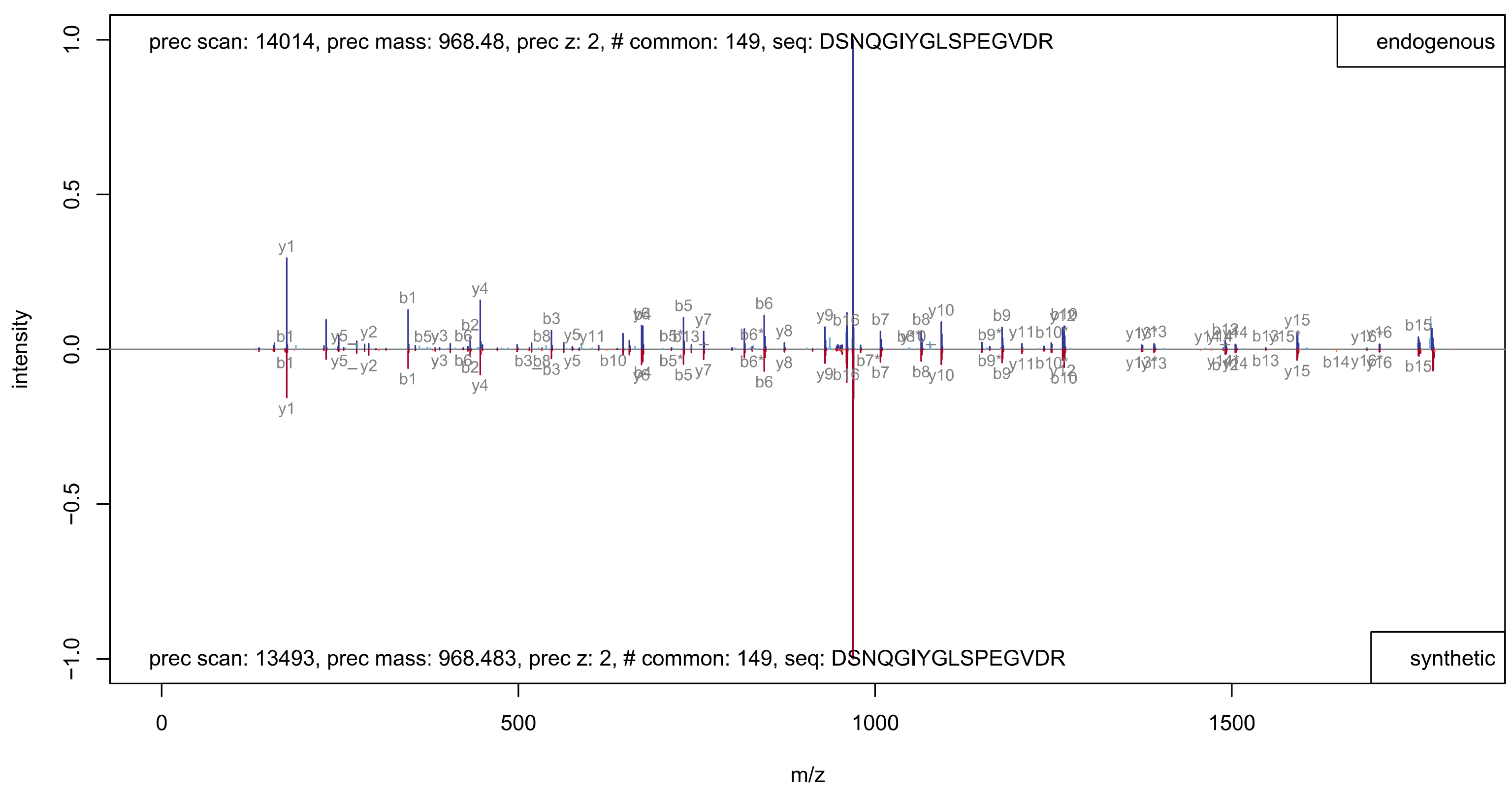

chr11_62494678

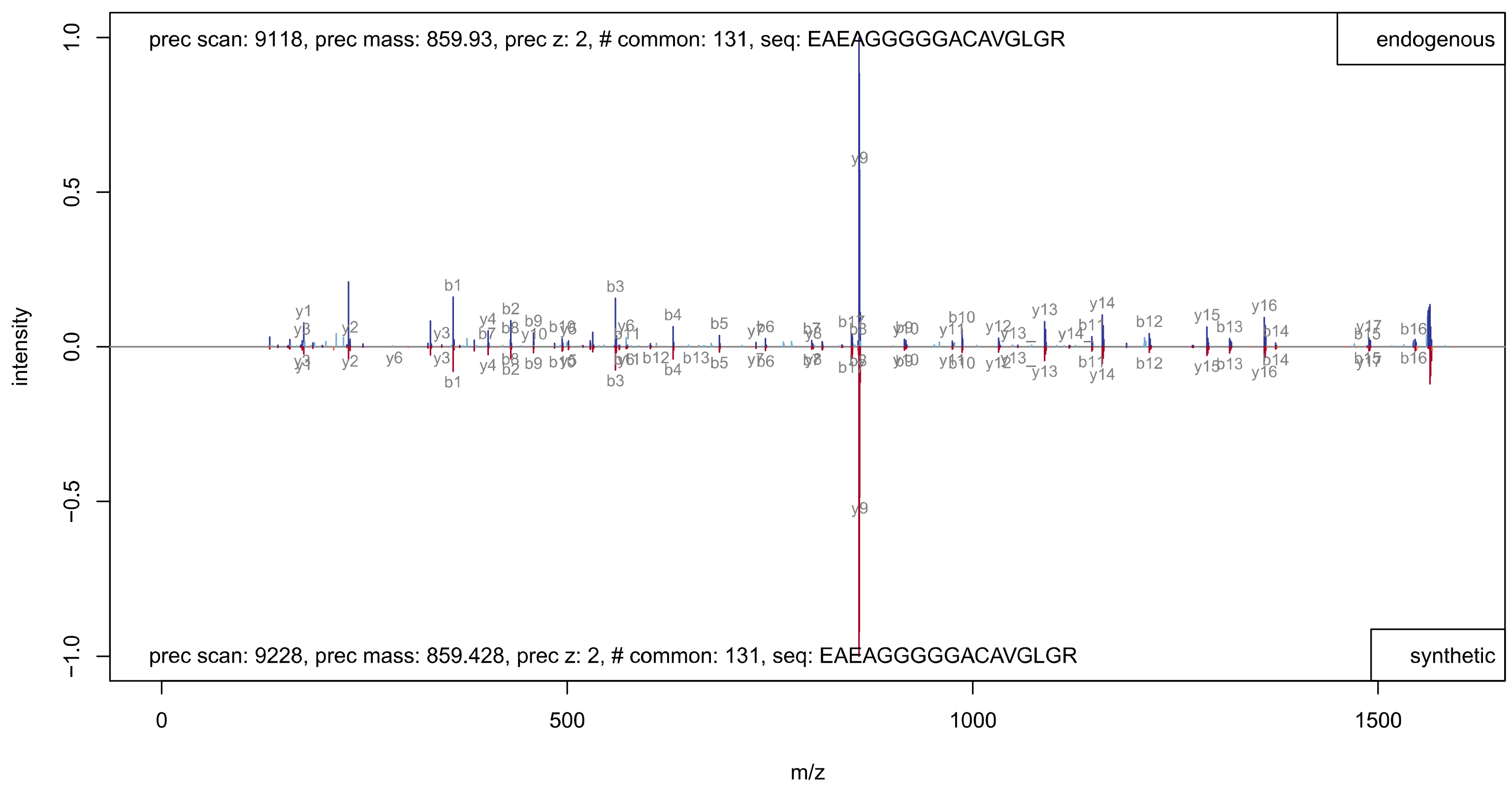

lnc−PDE6G−2:1

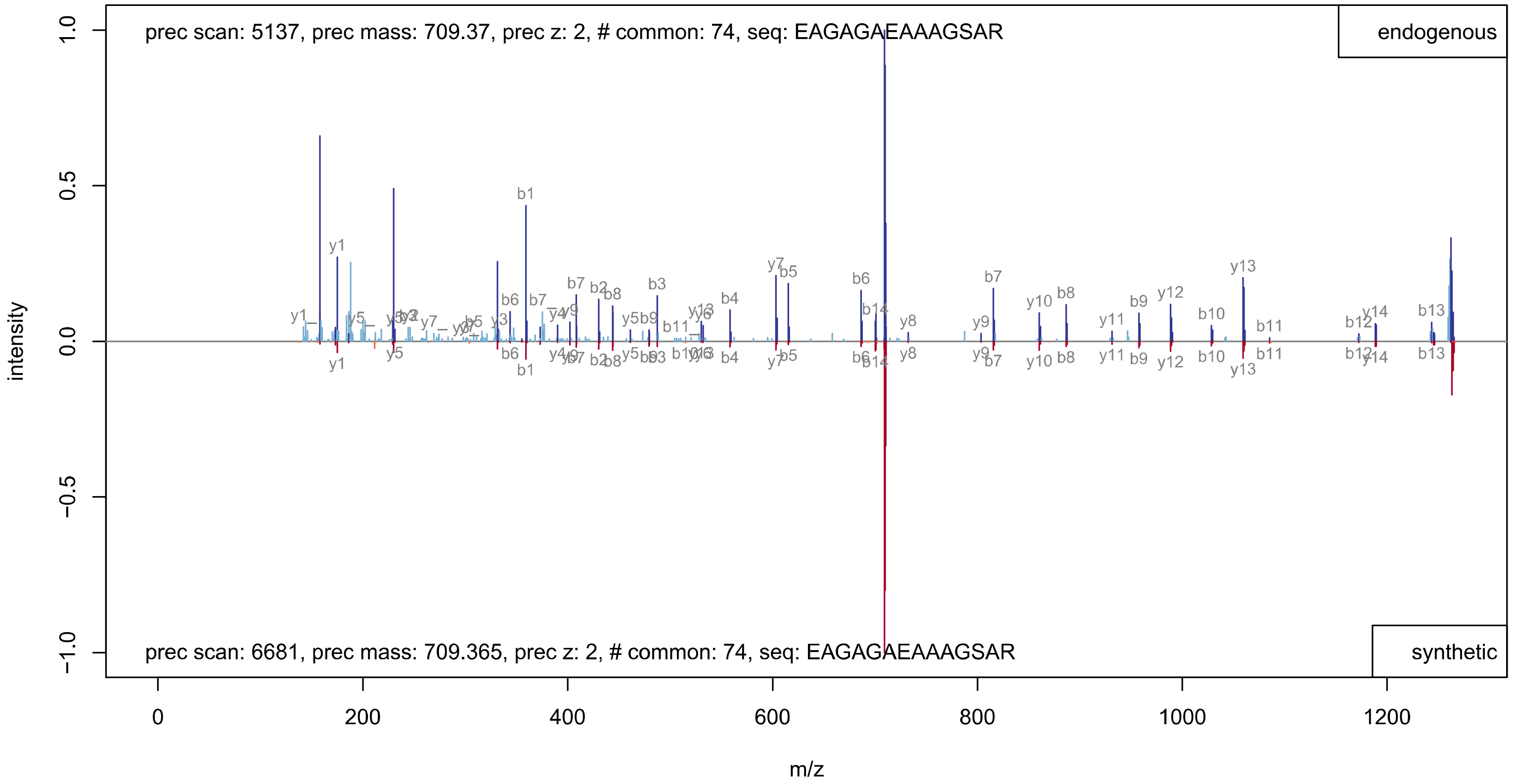

chr17_79604005

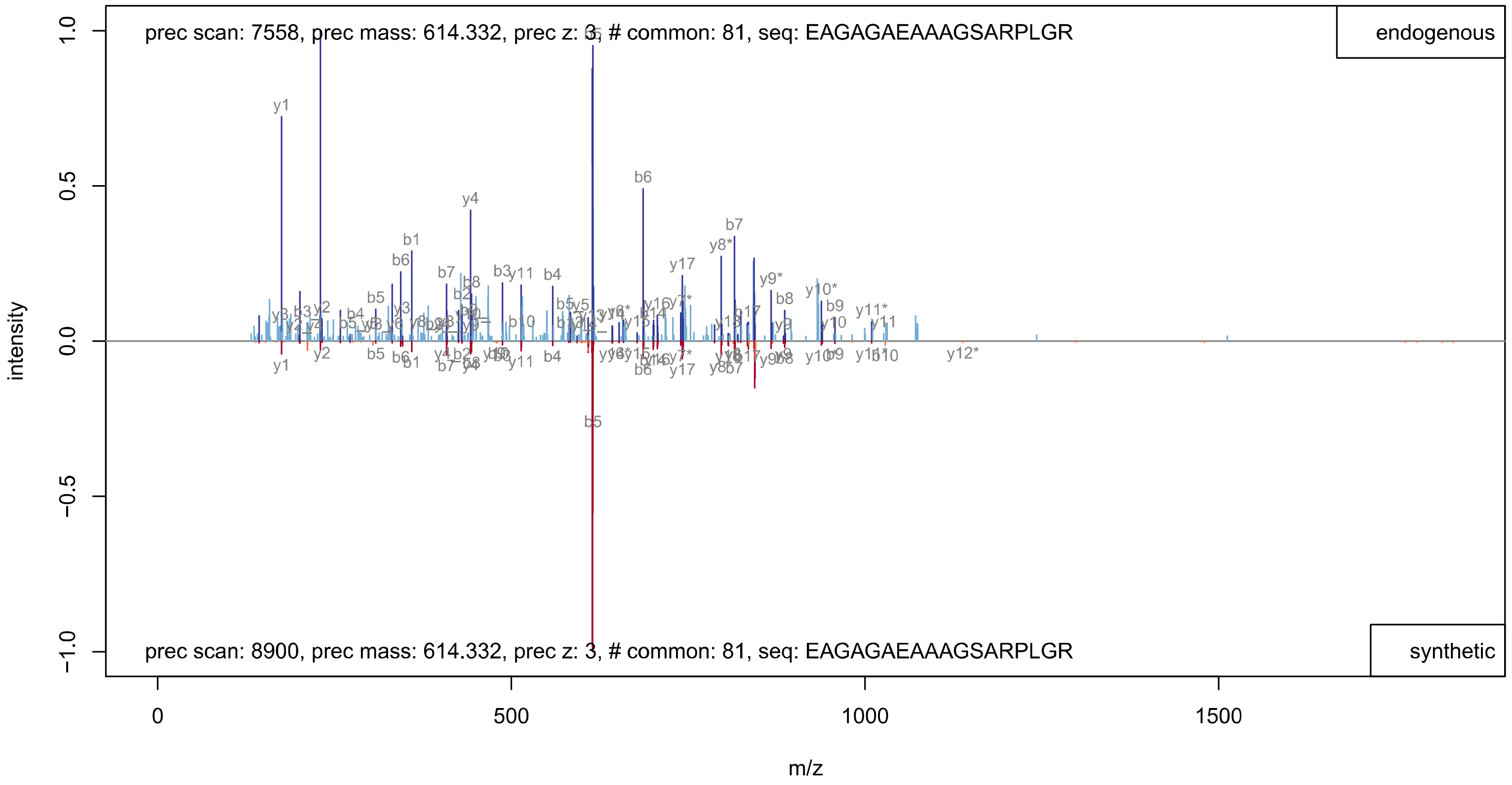

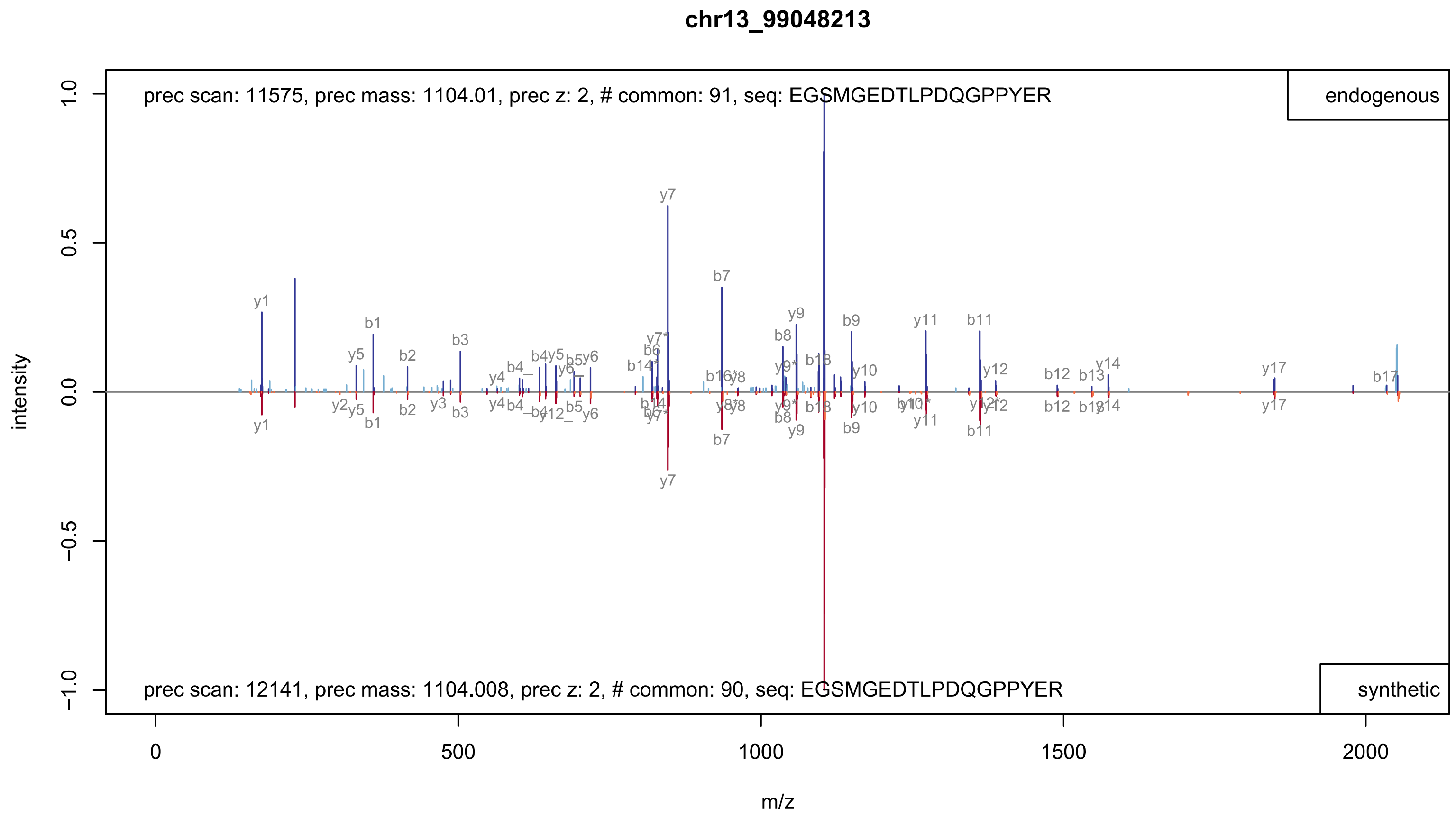
chr13_99048213
prec scan: 11575, prec mass: 1104.01, prec z: 2, # common: 91, seq: EGSMGEDTLPDQGPPYER
endogenous
prec scan: 12141, prec mass: 1104.008, prec z: 2, # common: 90, seq: EGSMGEDTLPDQGPPYER
synthetic
intensity
m/z

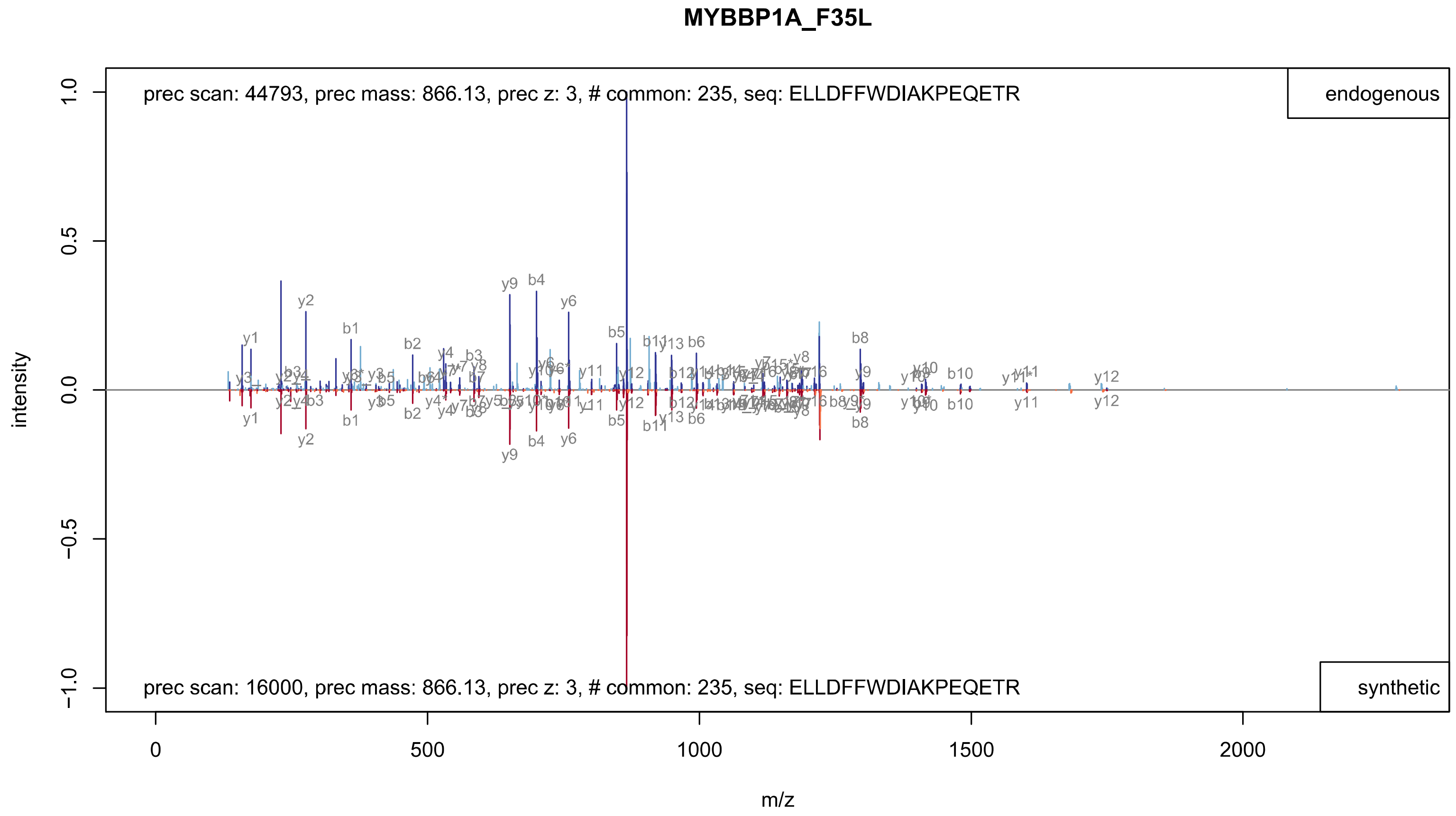
MYBBP1A_F35L
prec scan: 44793, prec mass: 866.13, prec z: 3, # common: 235, seq: ELLDFFWDIAKPEQETR
endogenous
prec scan: 16000, prec mass: 866.13, prec z: 3, # common: 235, seq: ELLDFFWDIAKPEQETR
synthetic
intensity
m/z

PGOHUM_ENST00000414254.1_KRT8P14

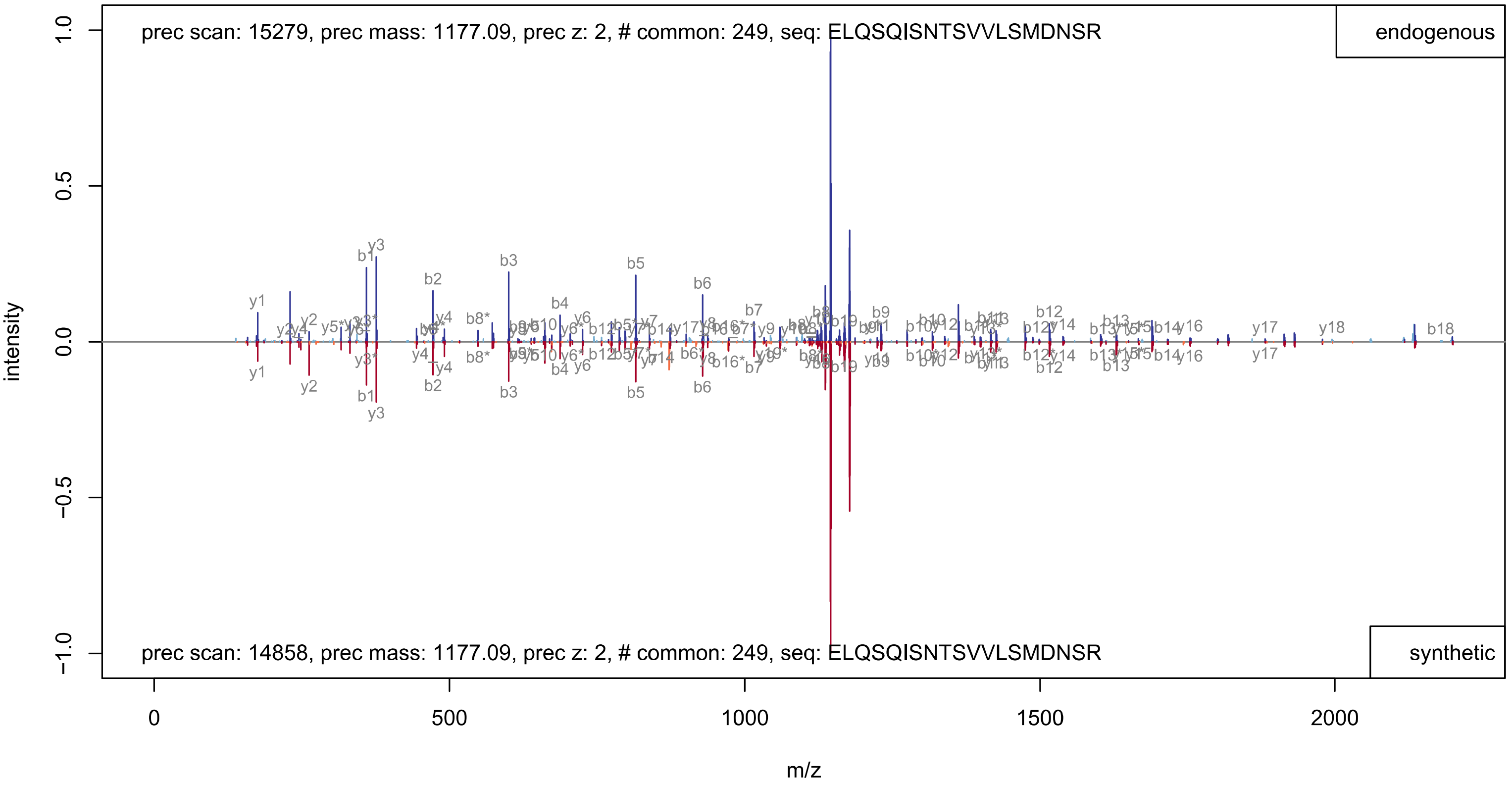

PGOHUM_ENST00000509955.1_RP11−341G5.2

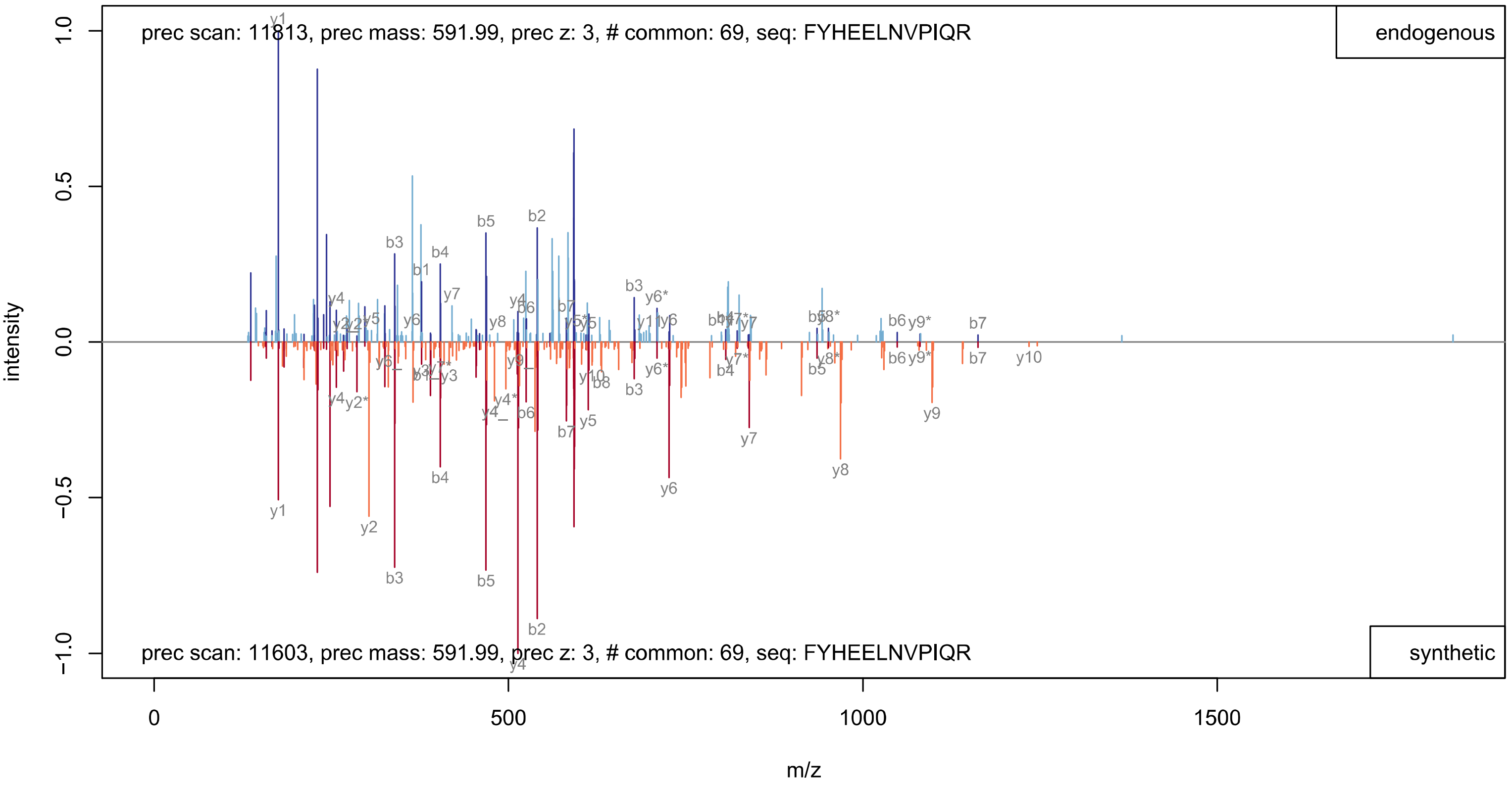

chr7_35076881

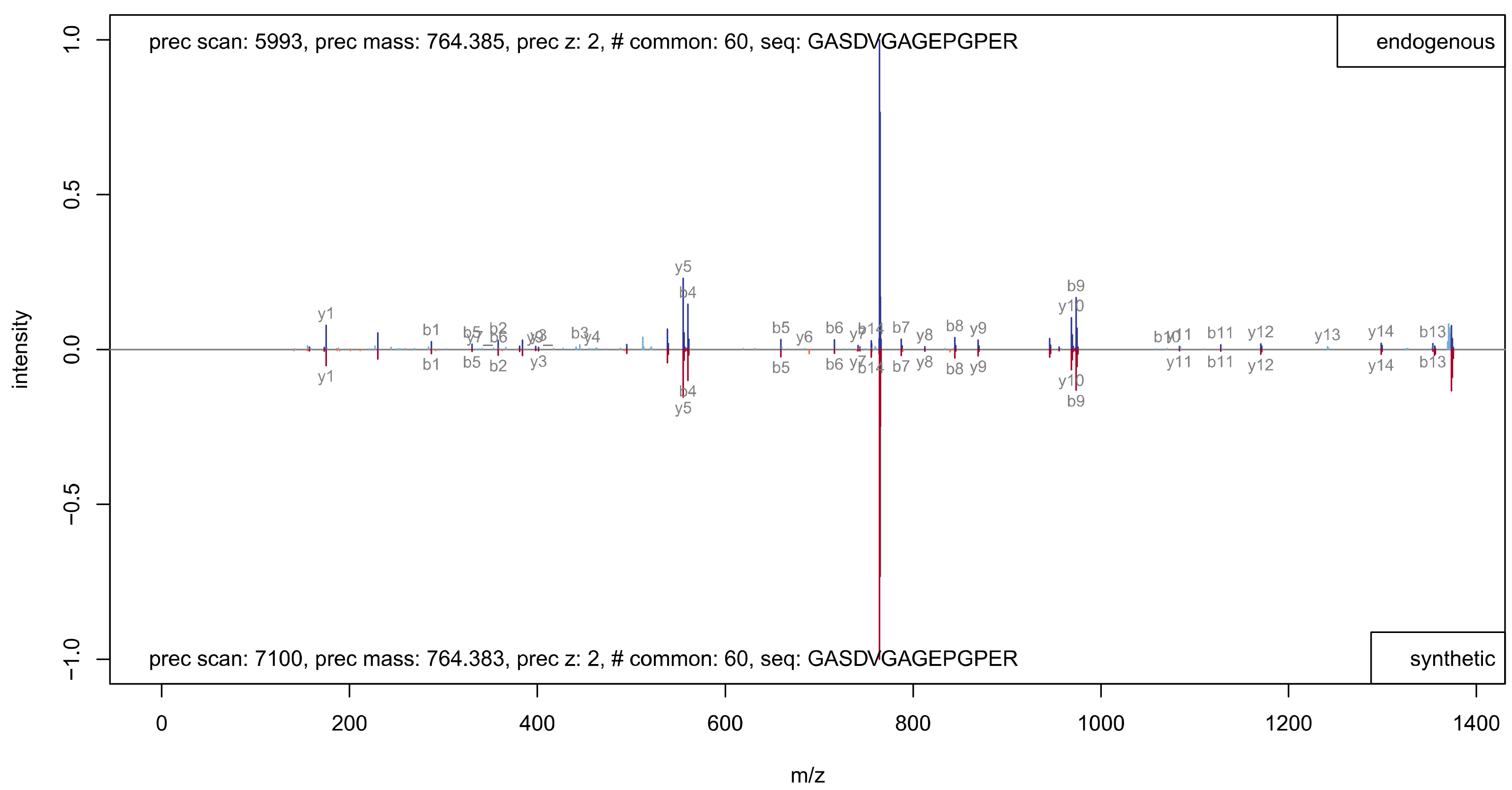

chrX_119125482

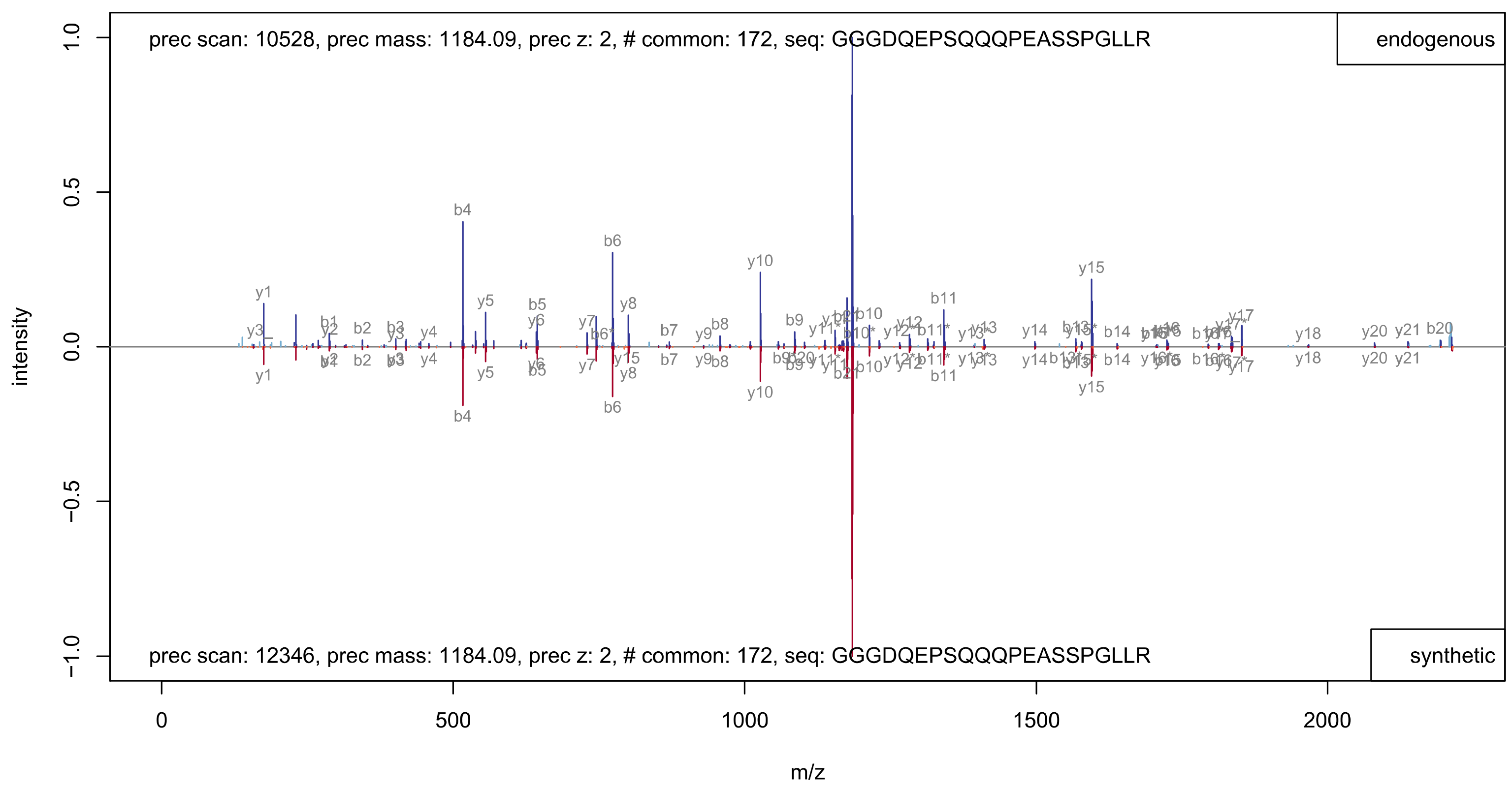

TRAP1_D395E

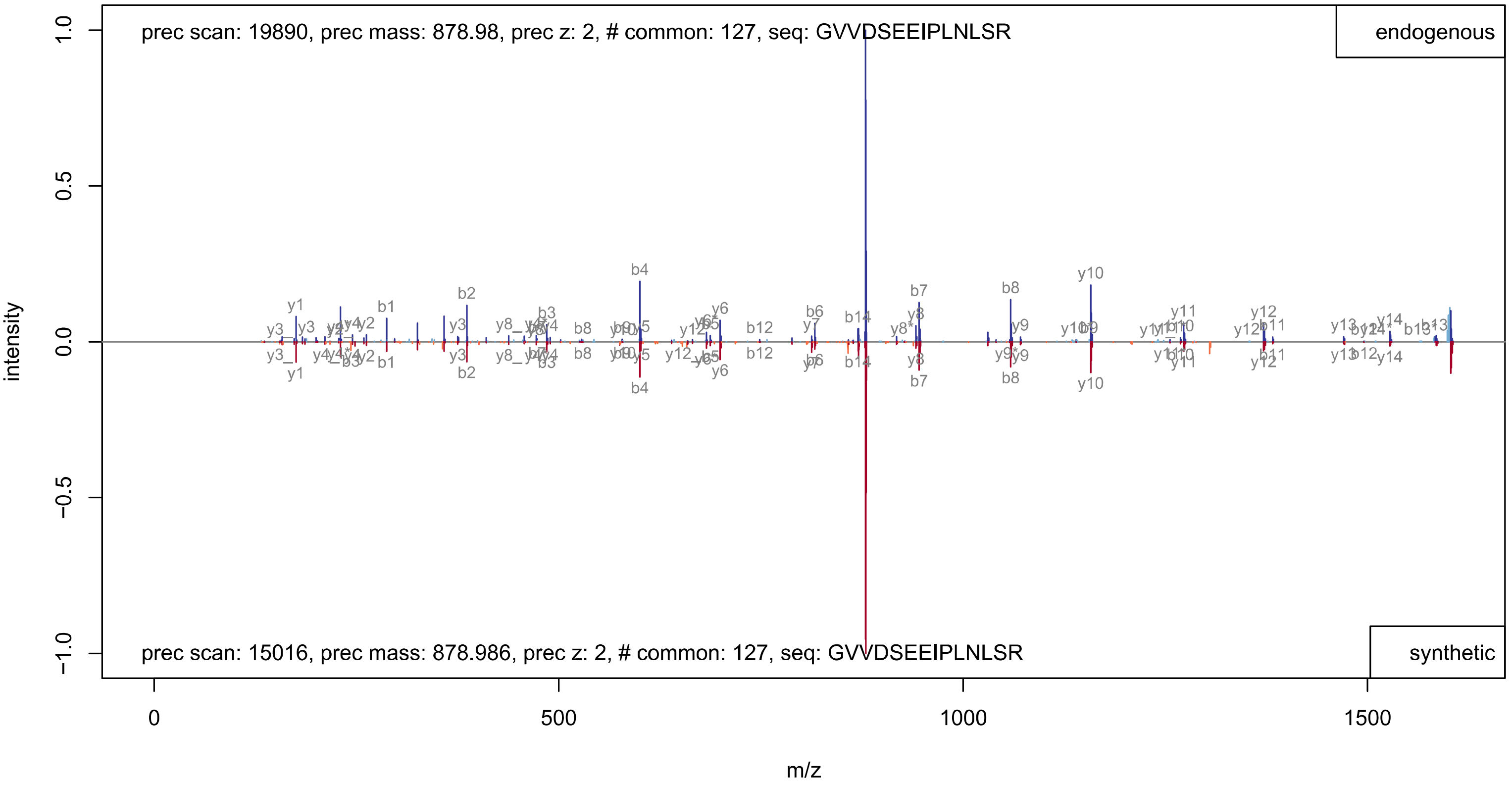

chr4_13339972

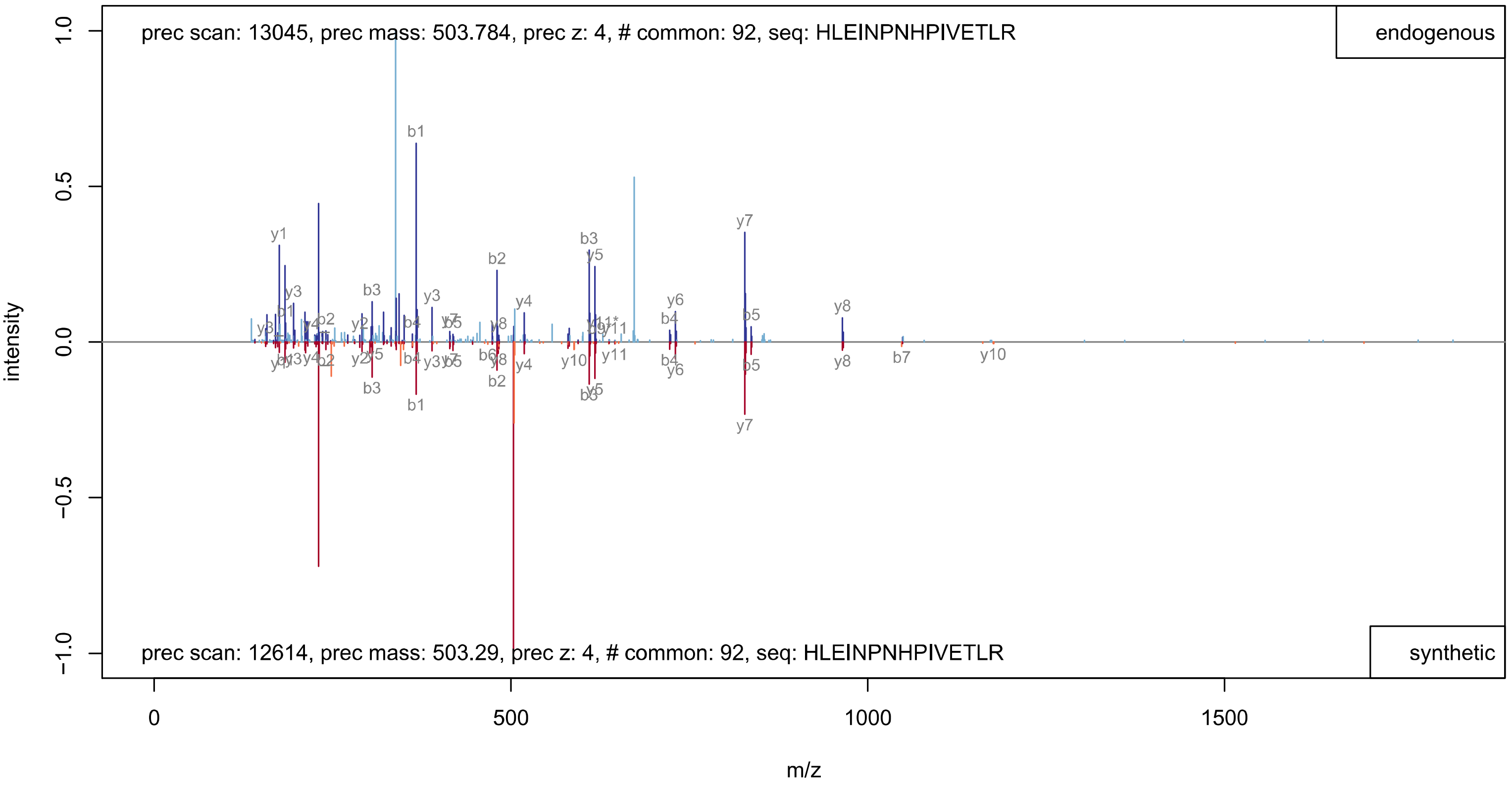

APOBEC3G_Q275E

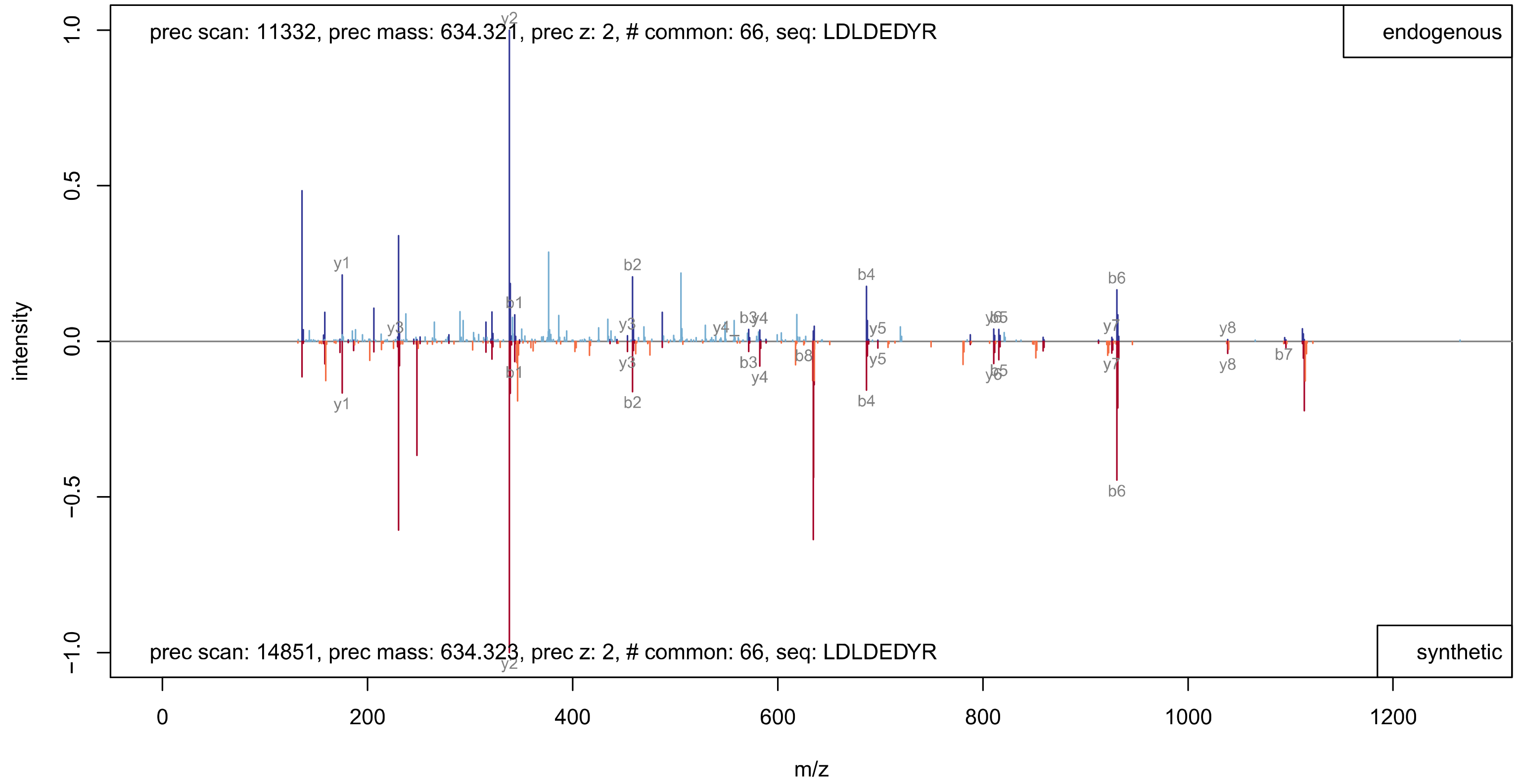

PGOHUM_ENST00000414254.1_KRT8P14

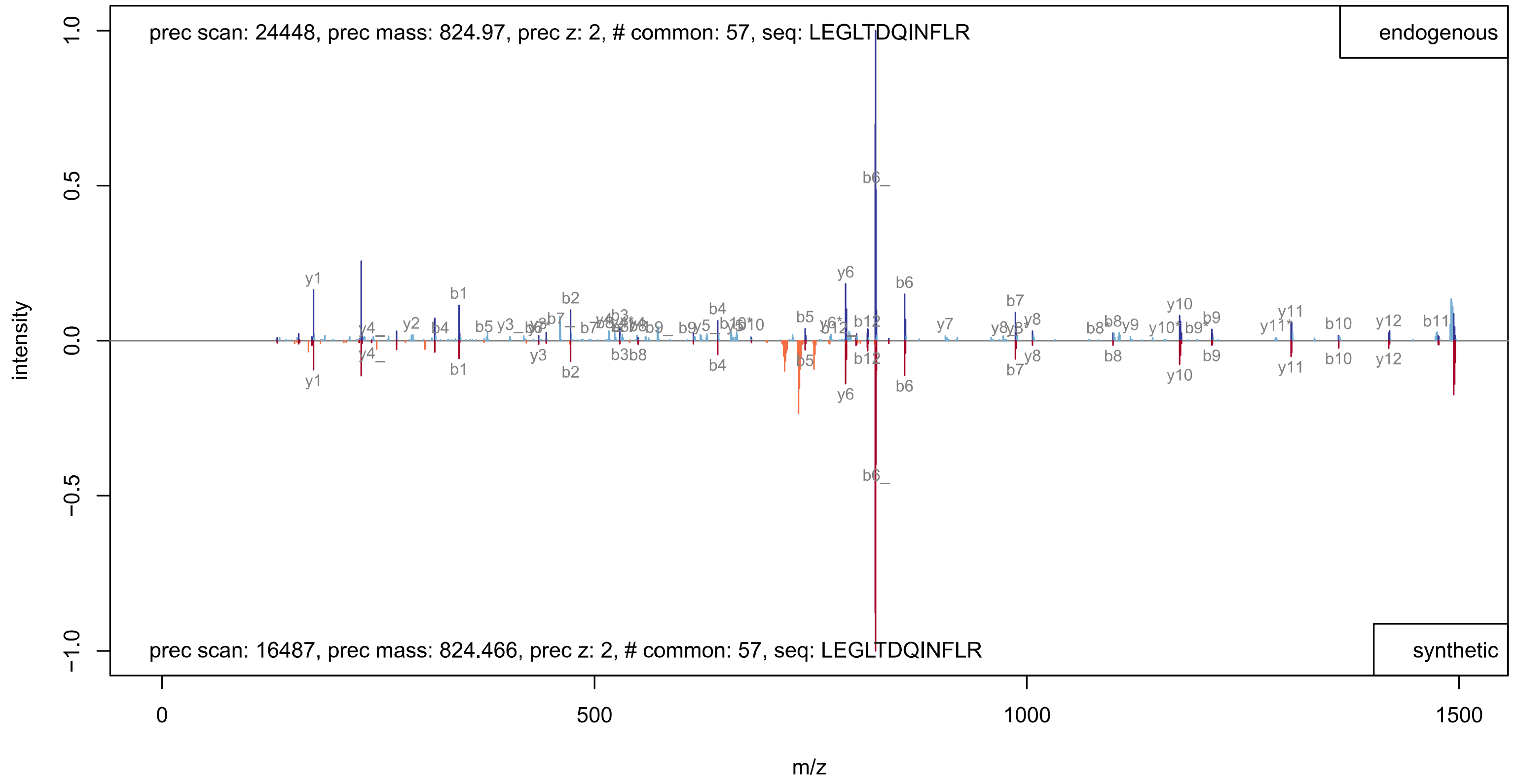

lnc−EIF2AK3−4:20

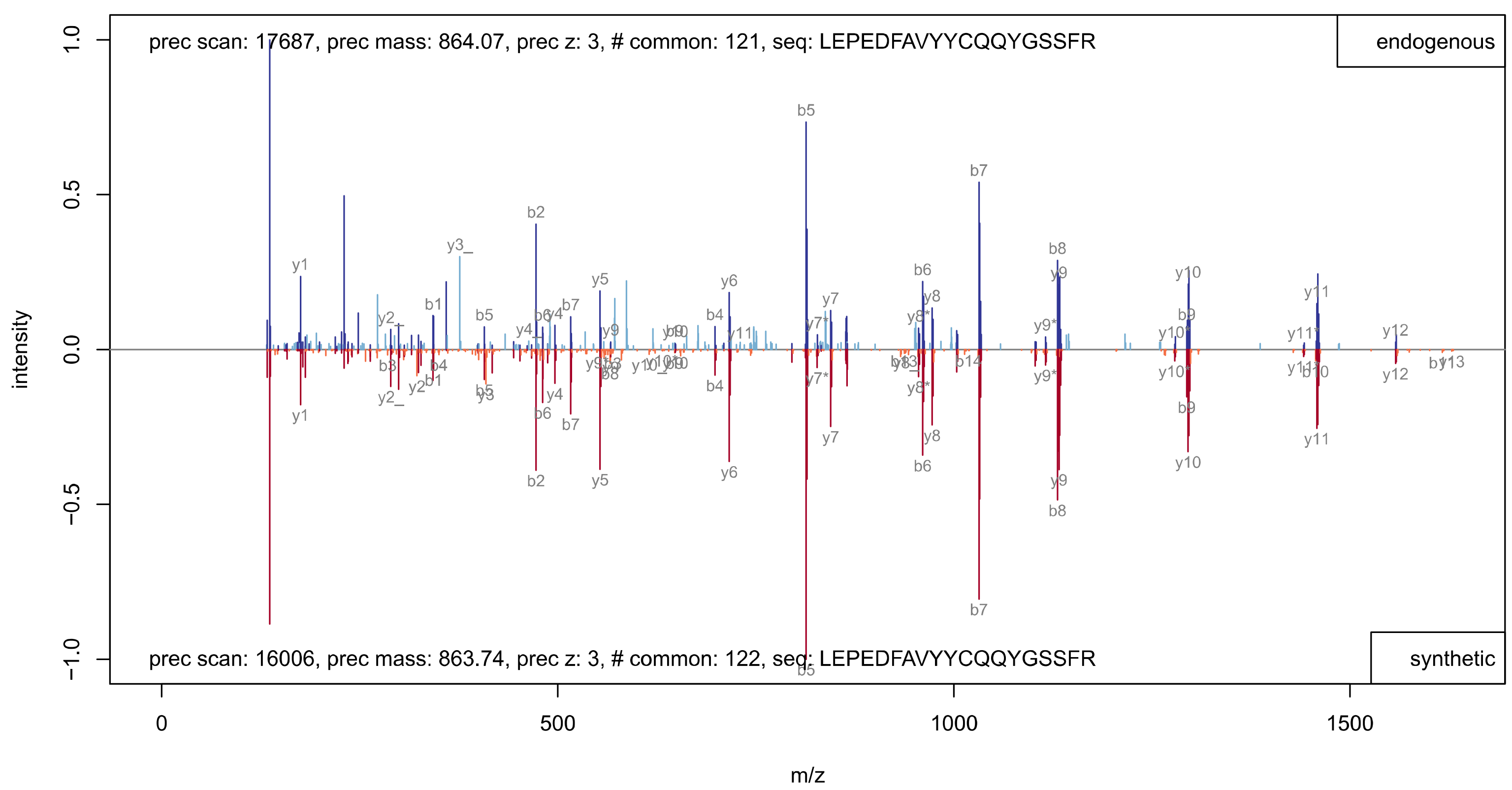

KRT14_A413T

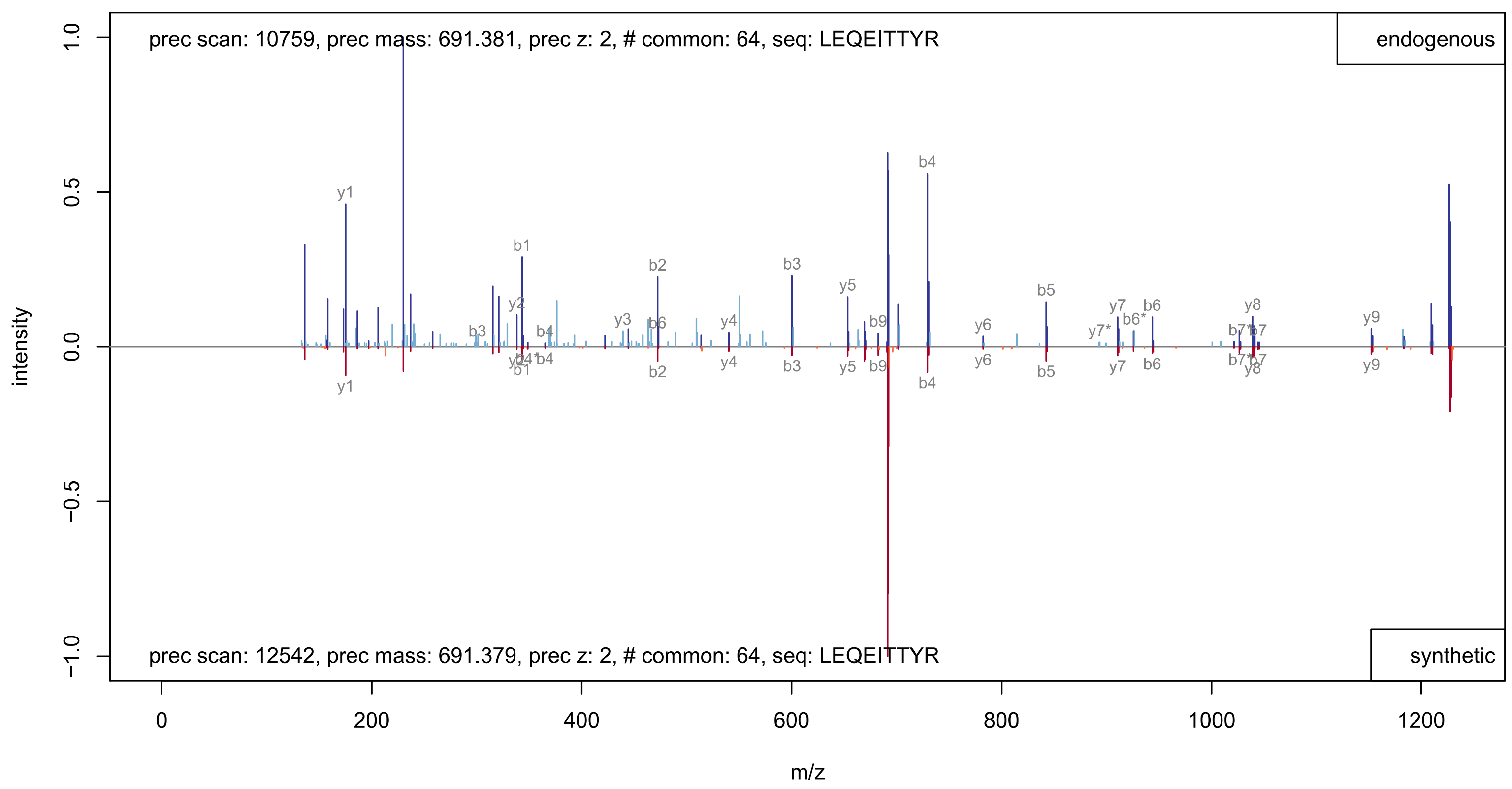

MLPH_L153P

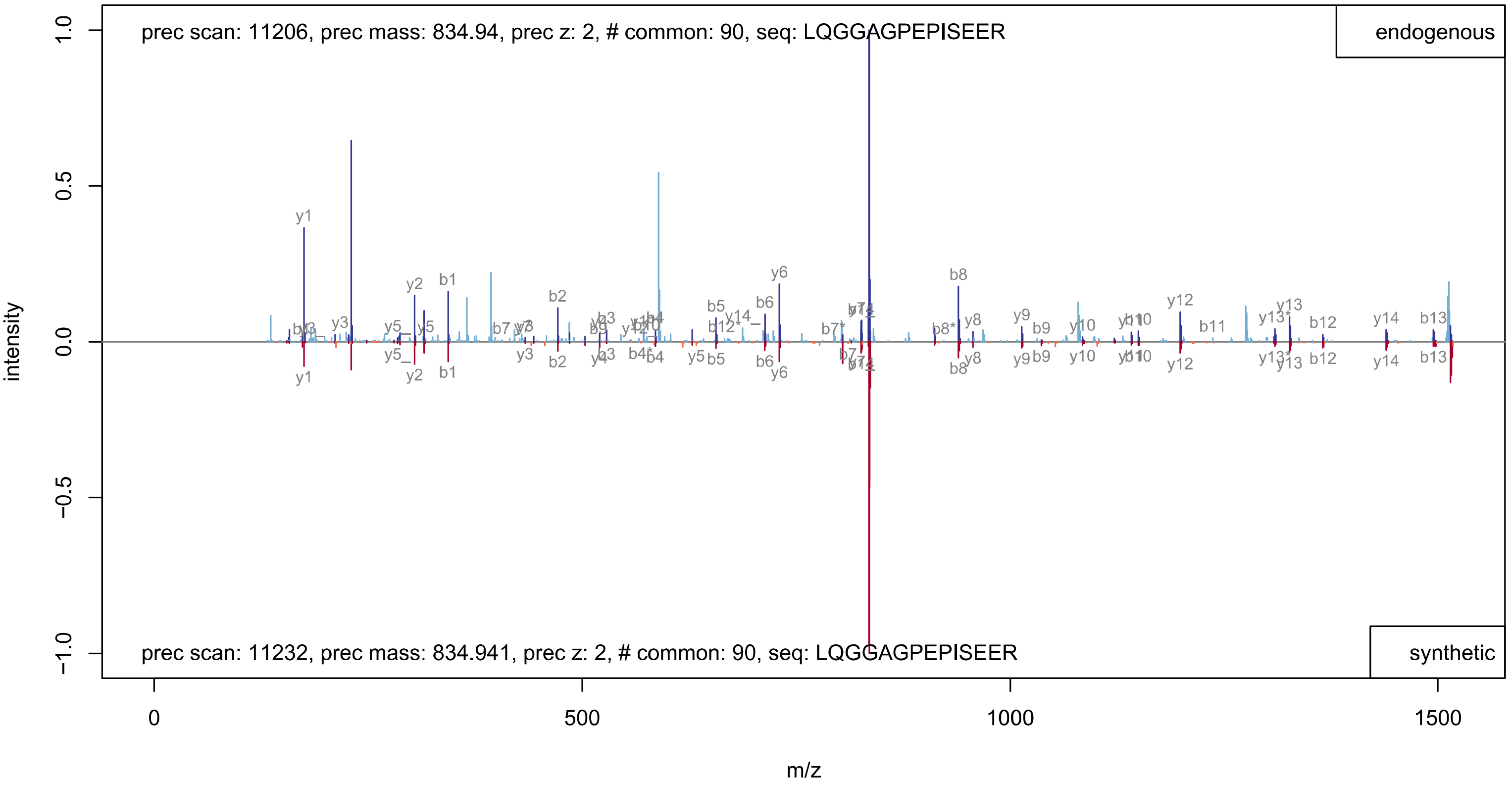

FOXA1_S448N

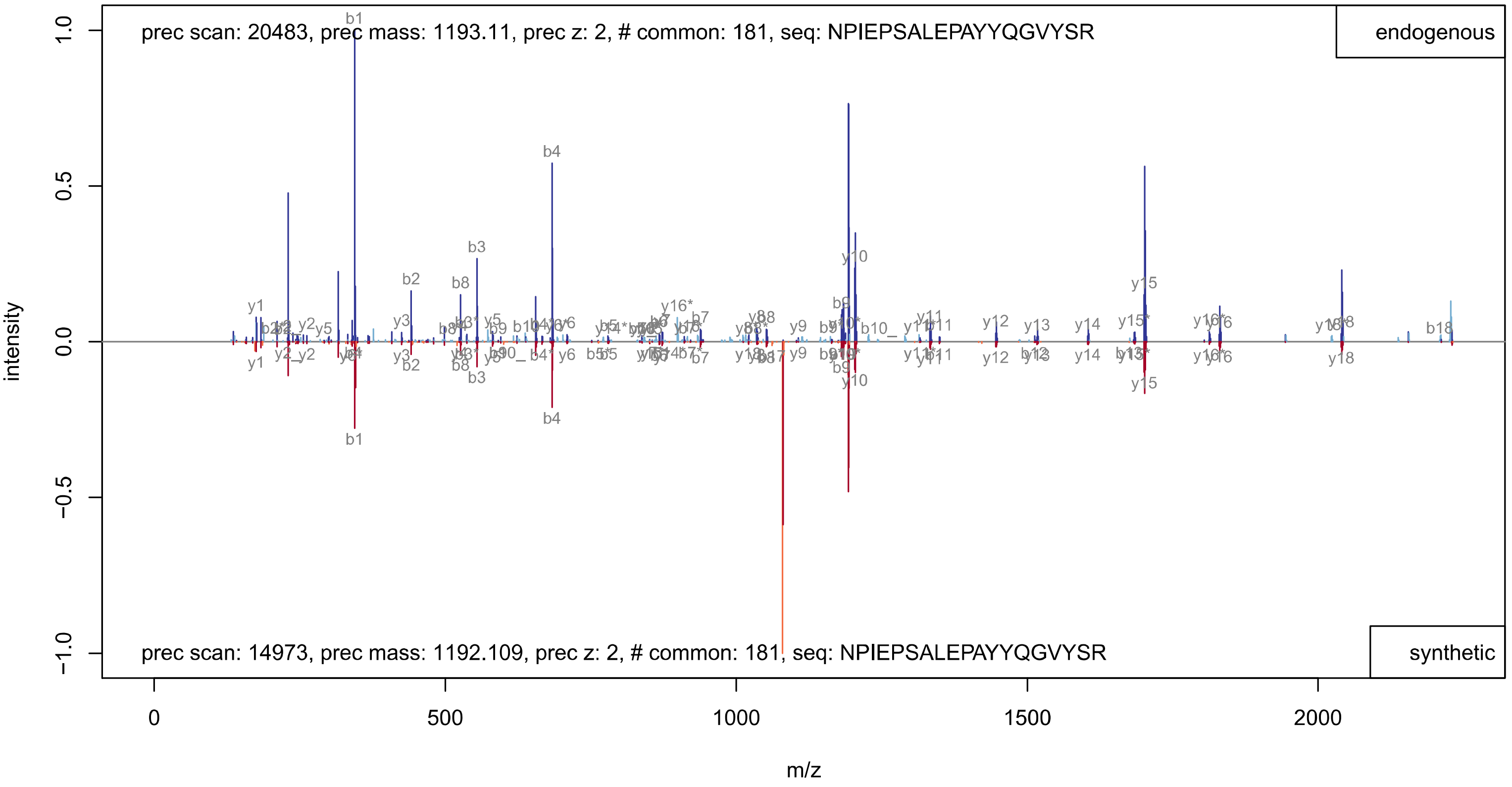

lnc−EIF2AK3−4:26

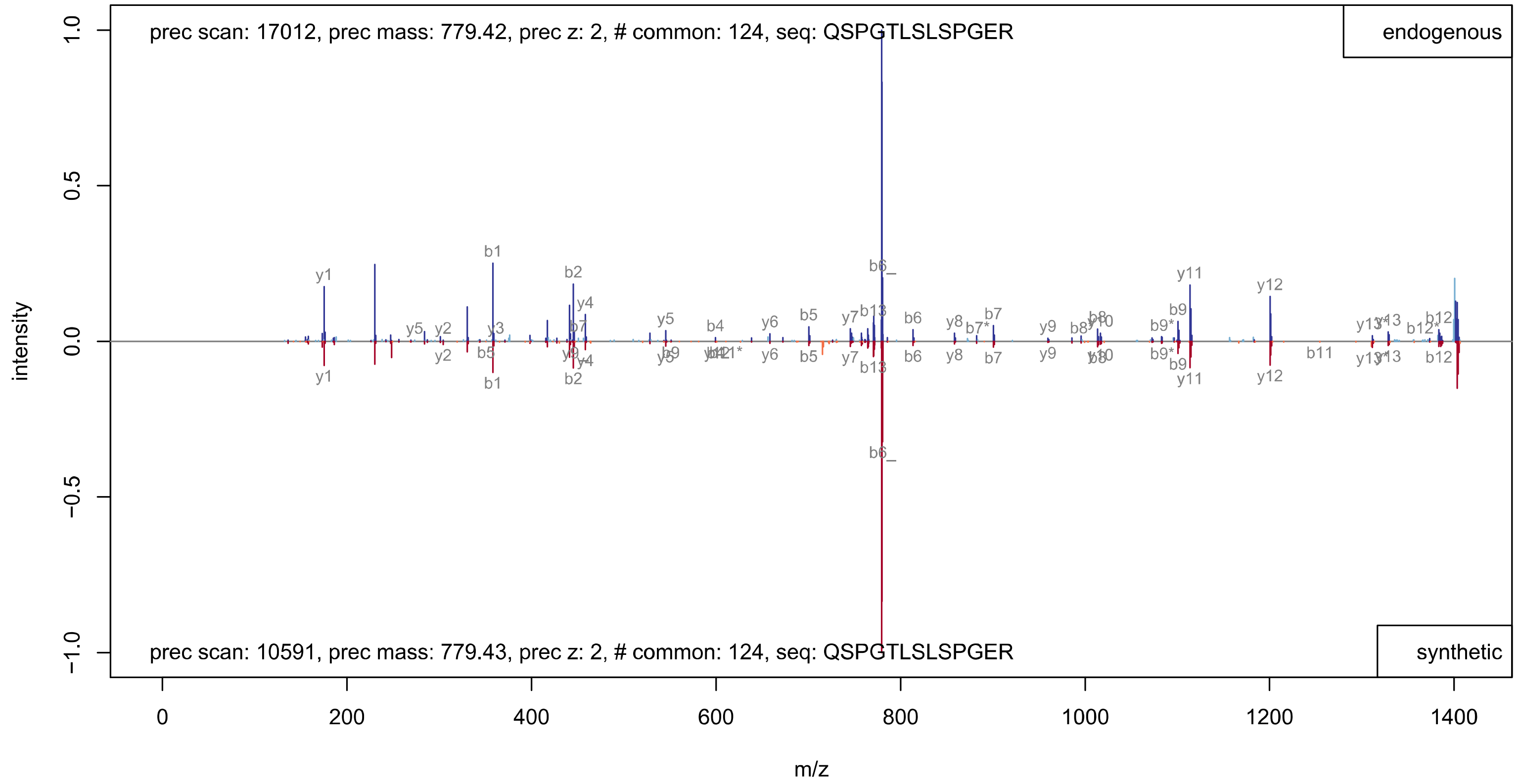

MKI67_K3217E

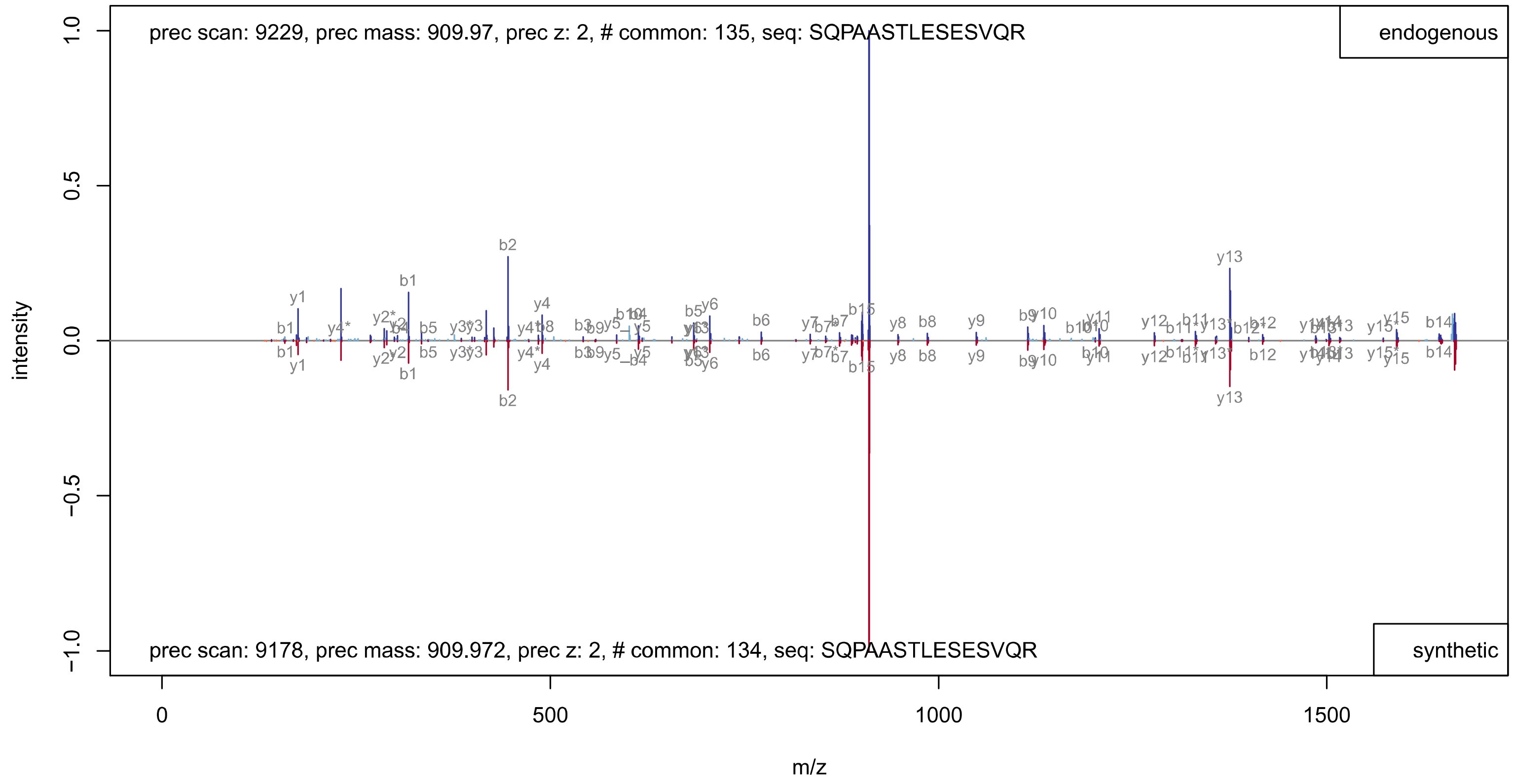

chr13_99047859

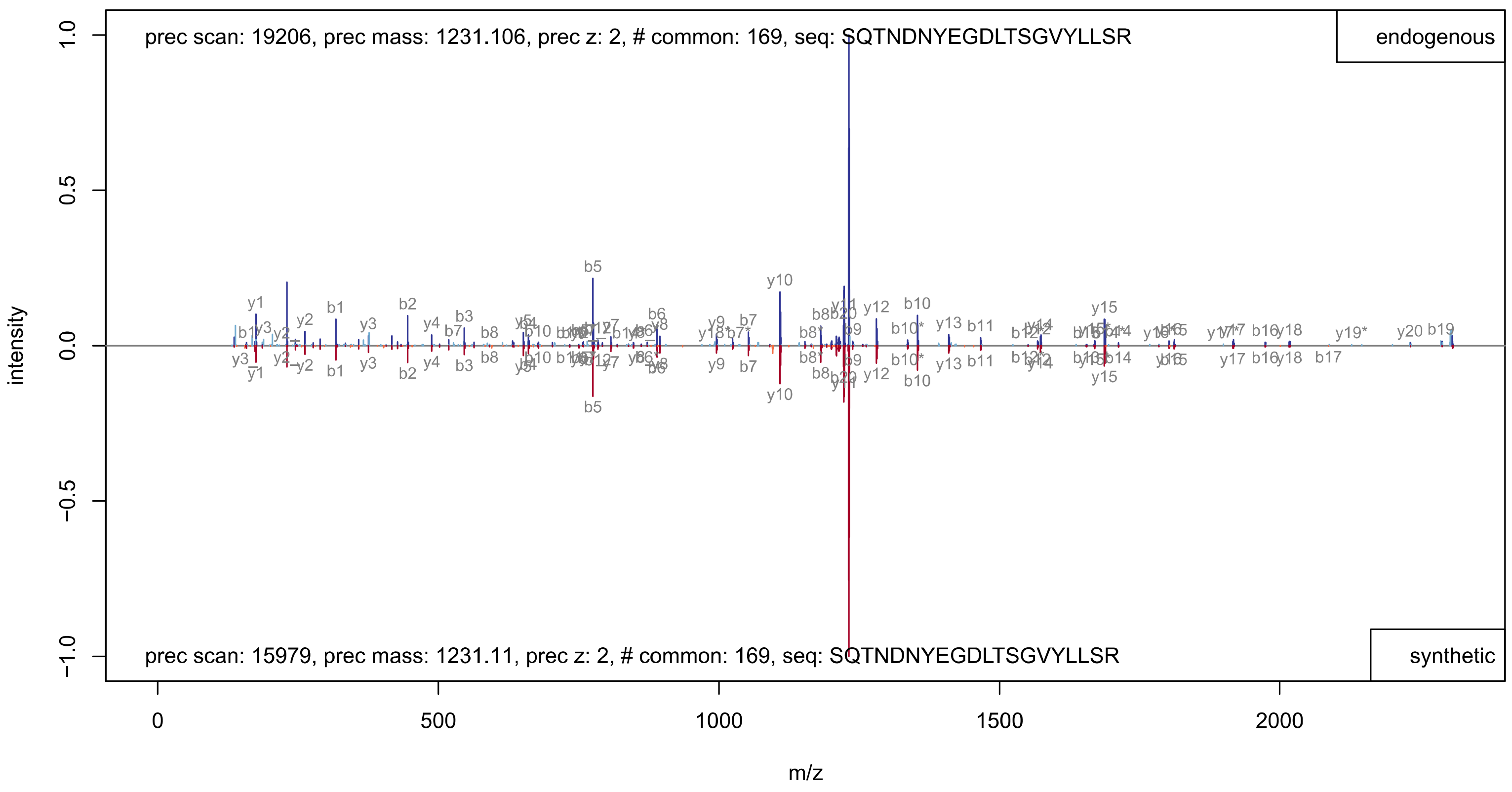

chr17_18575943

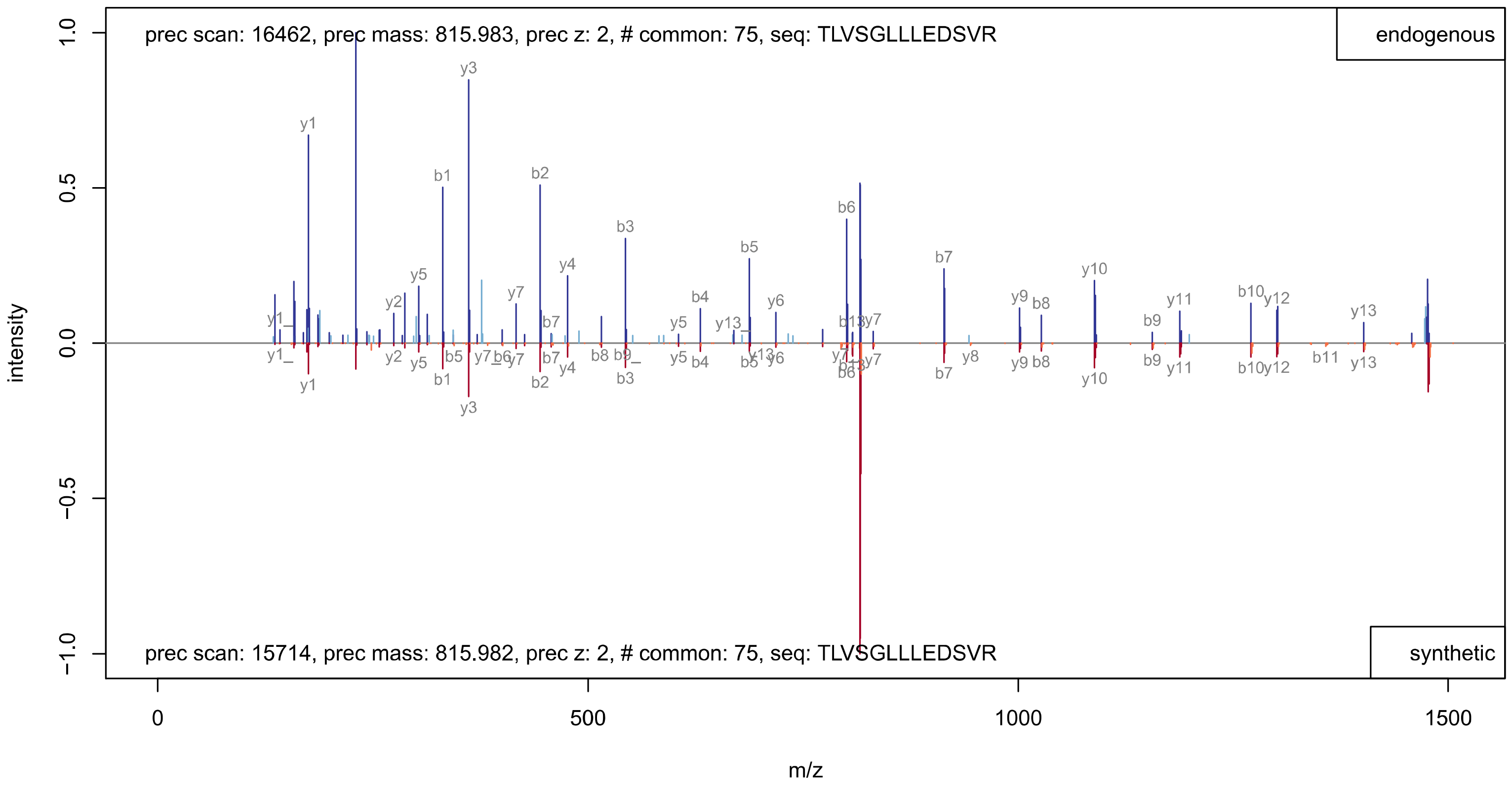

CDKN1B_V109G

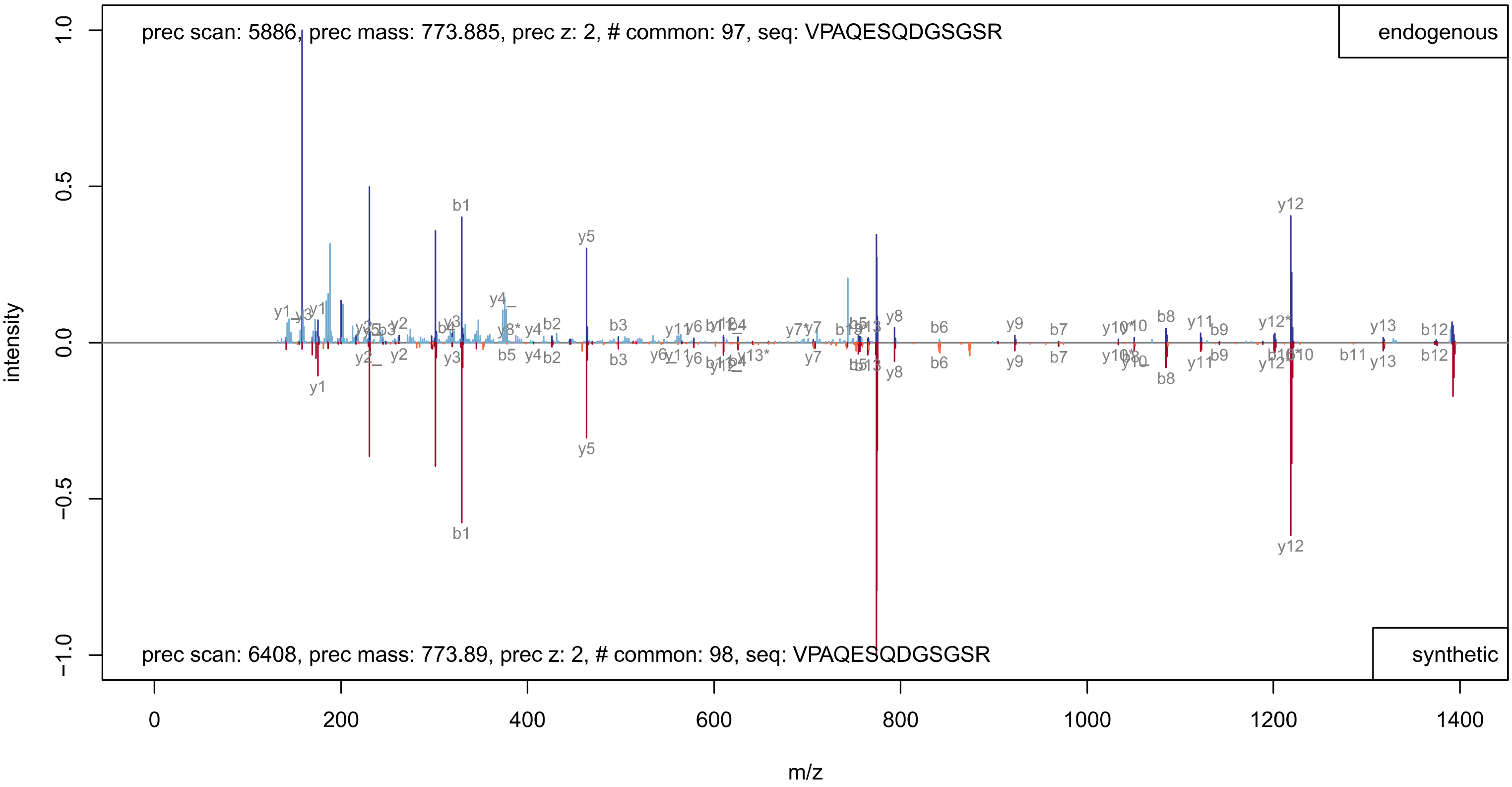

PGOHUM_ENST00000451609.1_KRT8P37

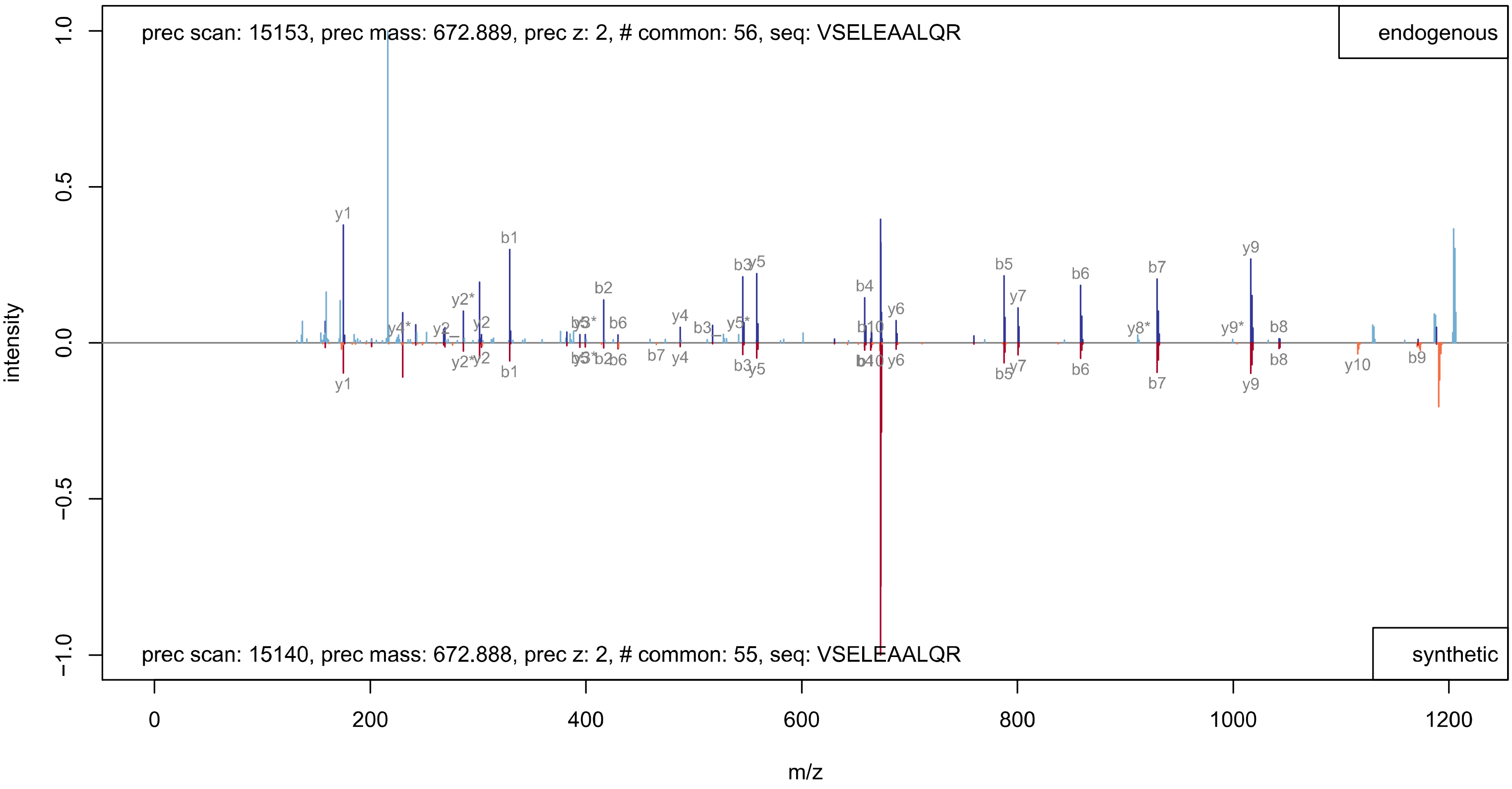

MKI67_N104S

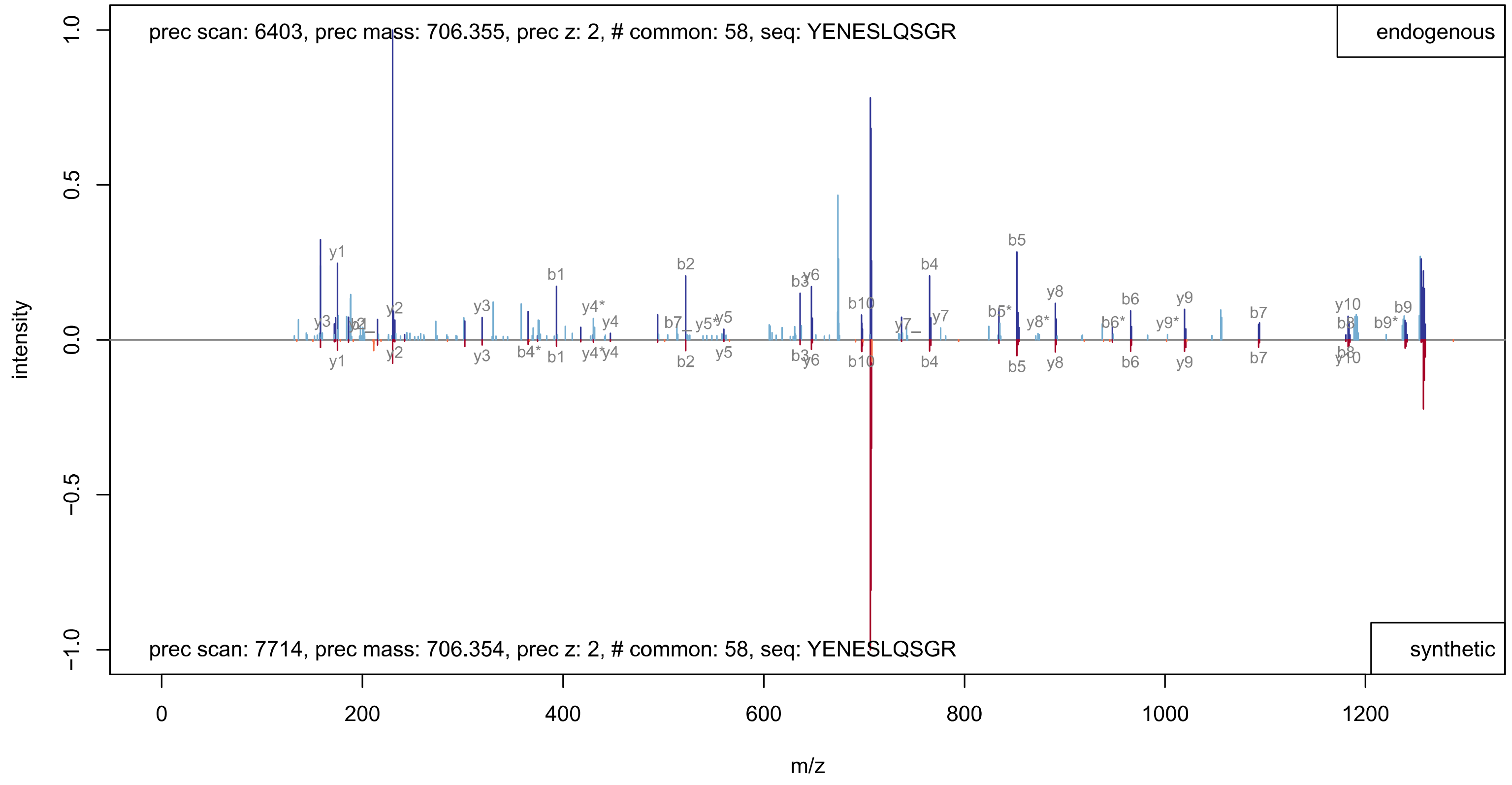

chr17_17041391

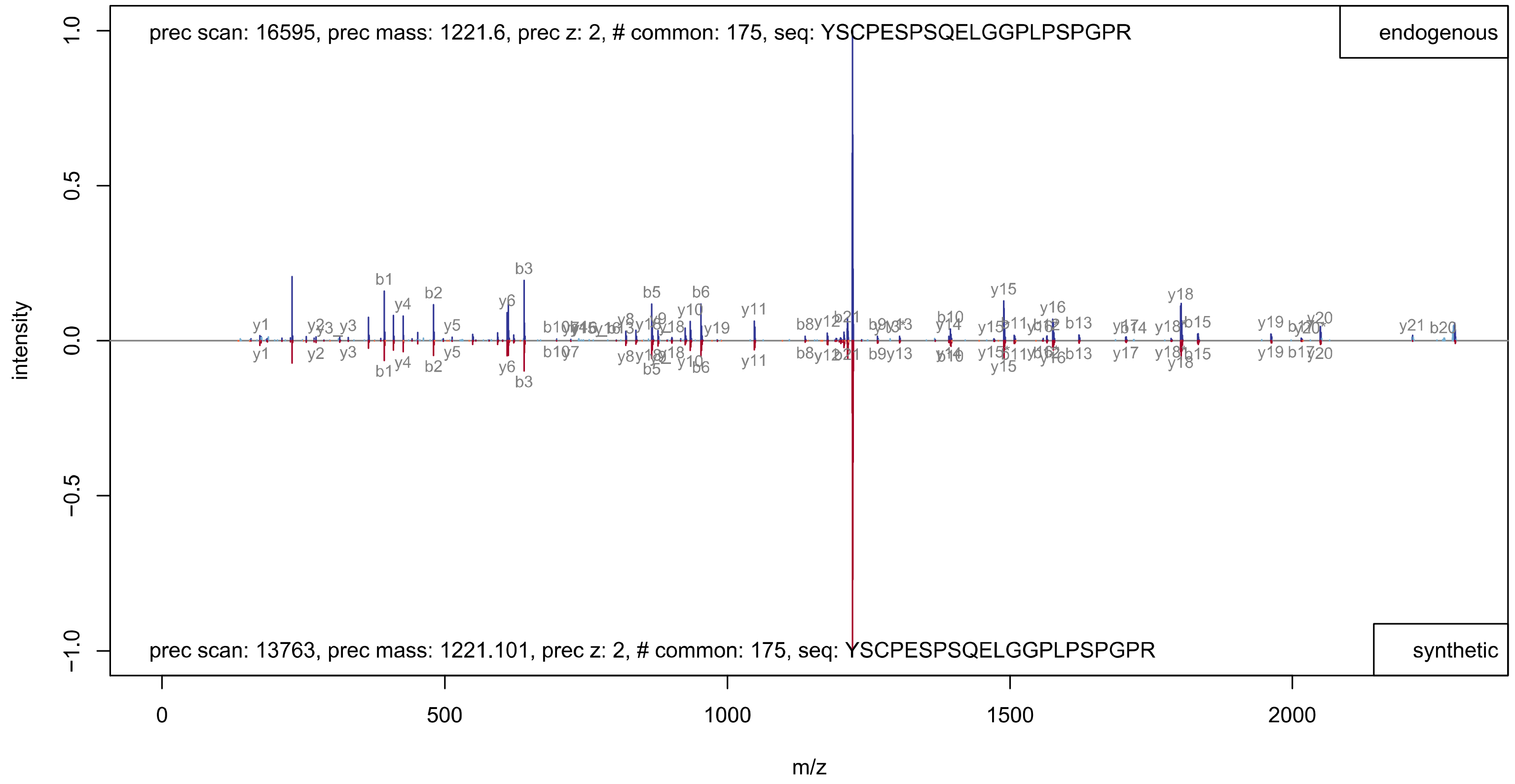

PGOHUM_ENST00000443360.1_RPS7P3

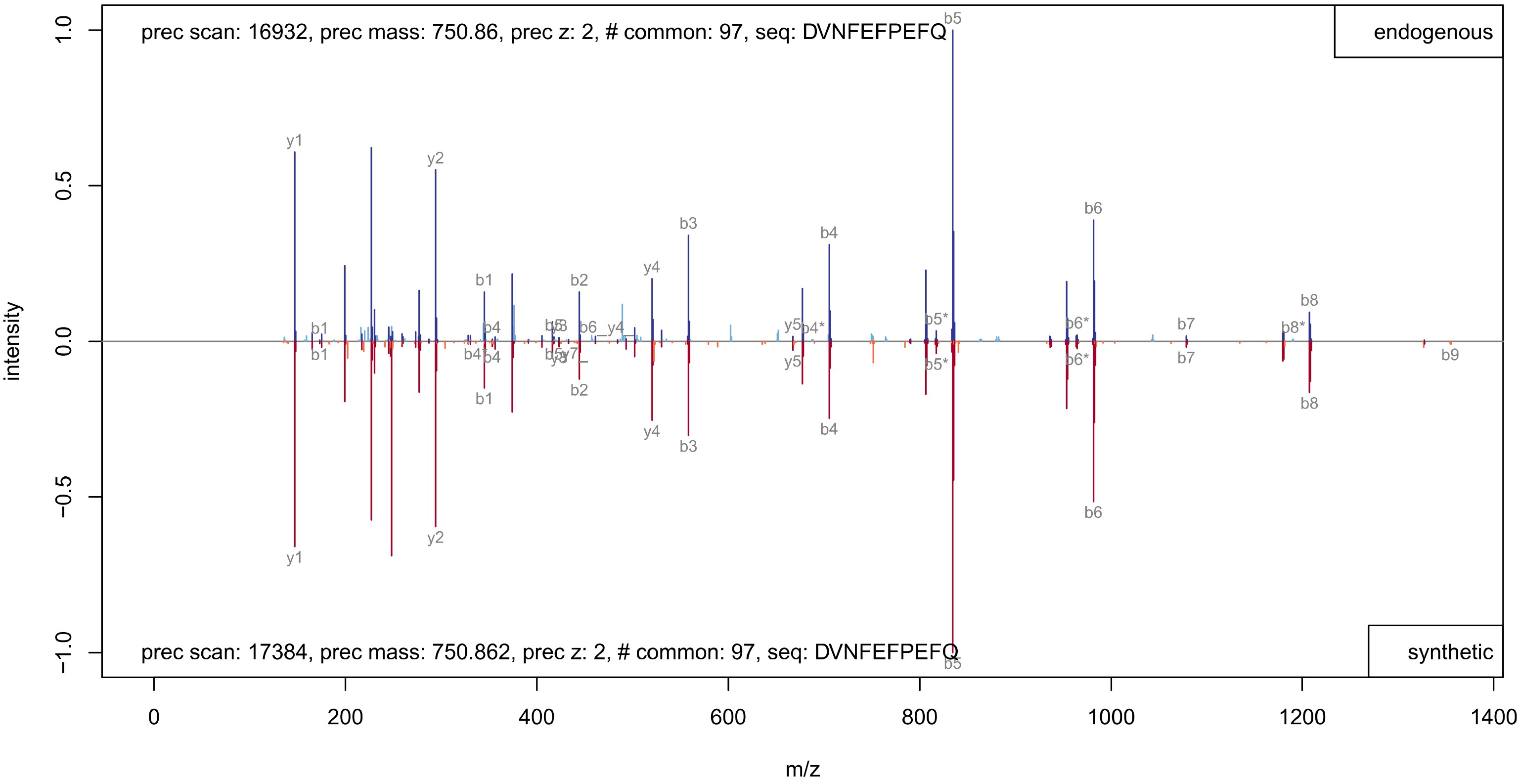

chr9_103738150

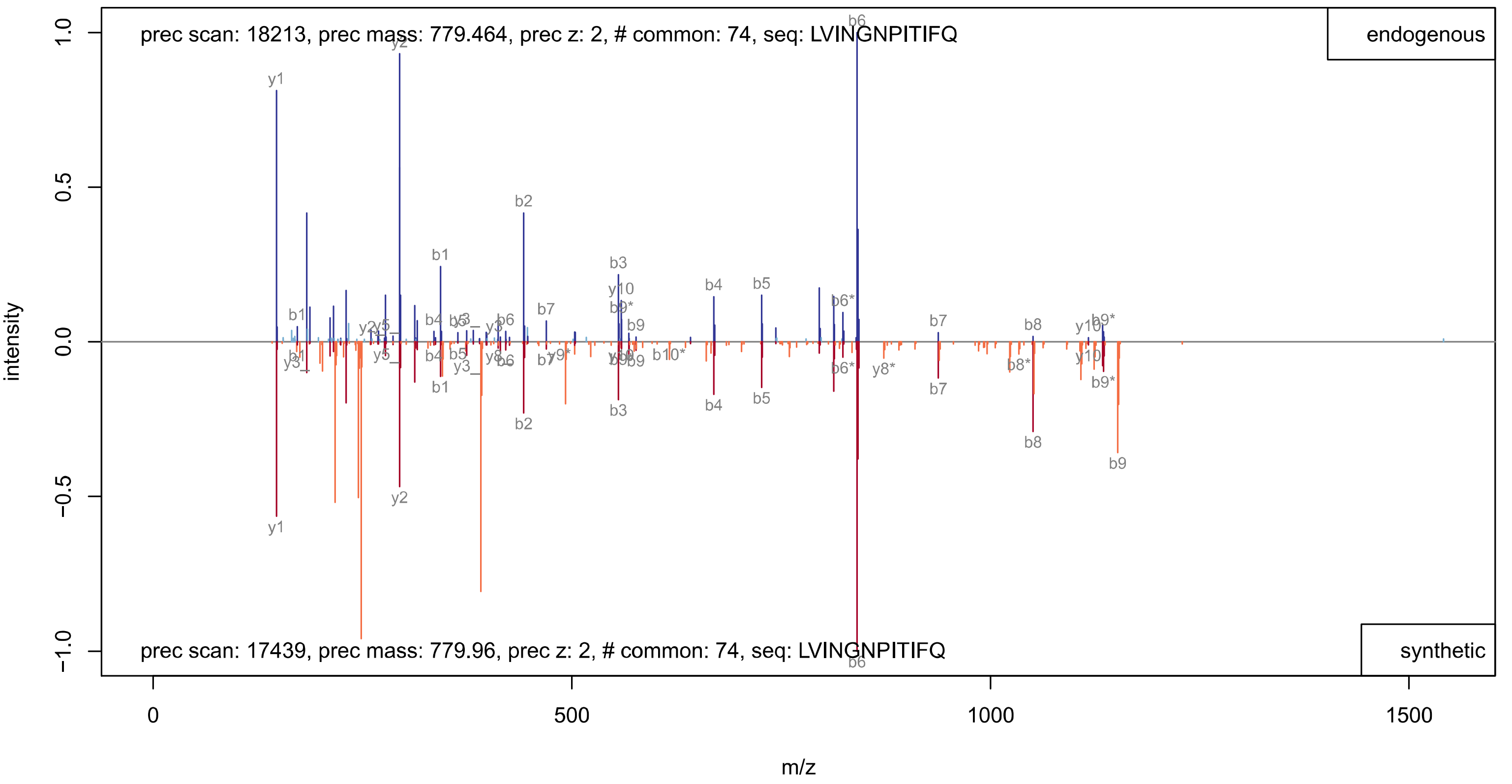

chr1_38273773

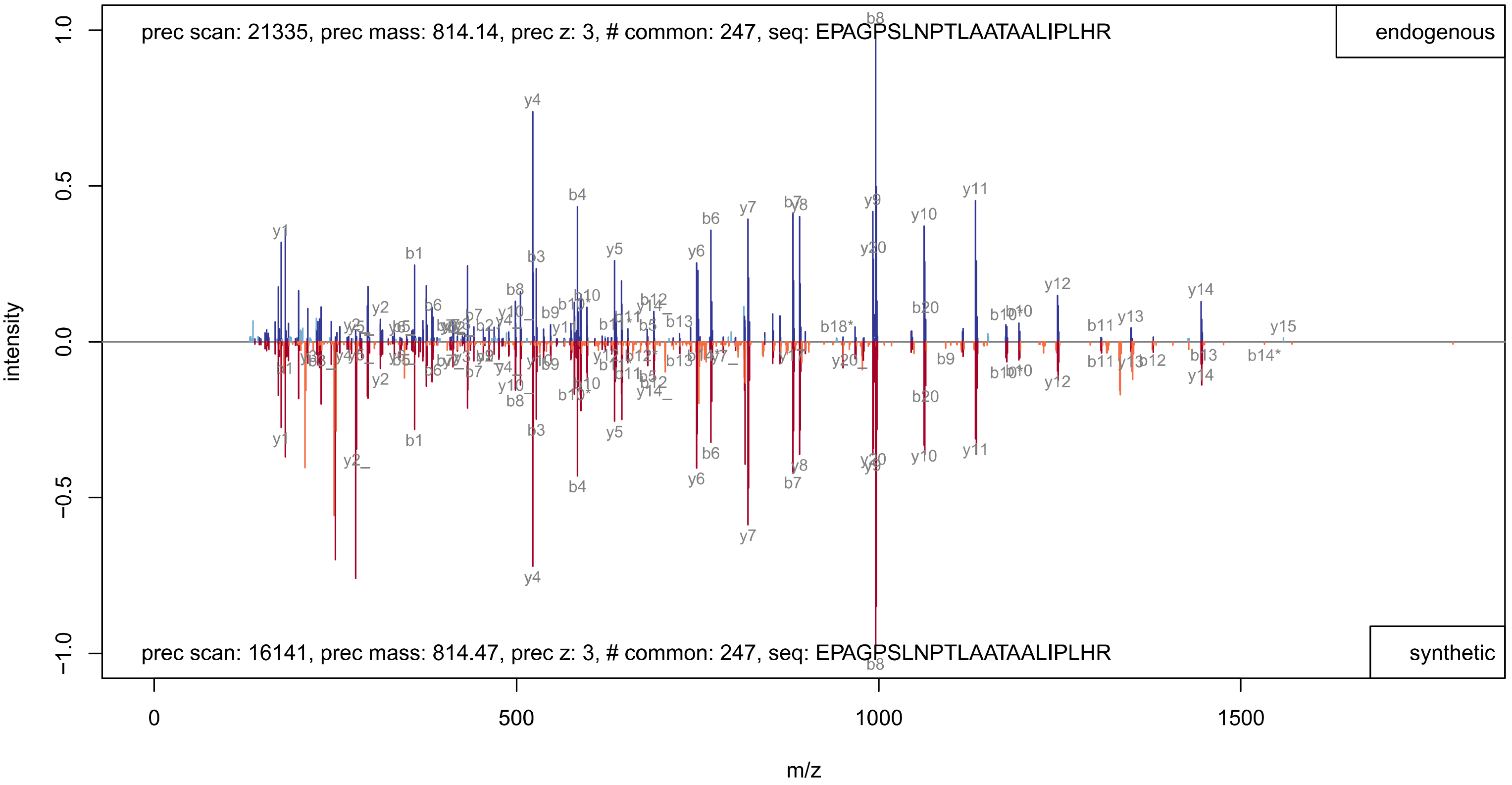

chr6_32055224

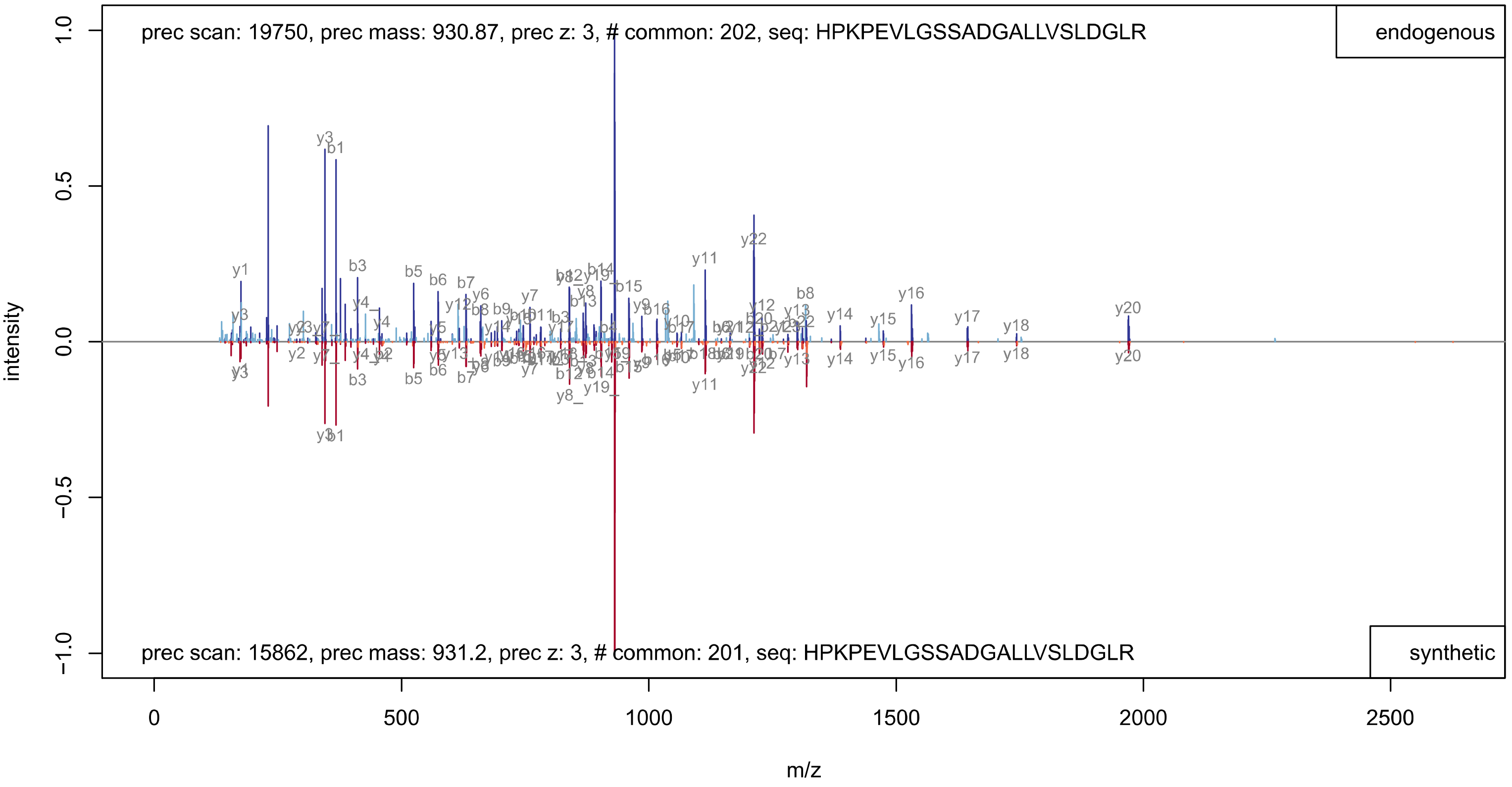

lnc−EIF2AK3−4:14

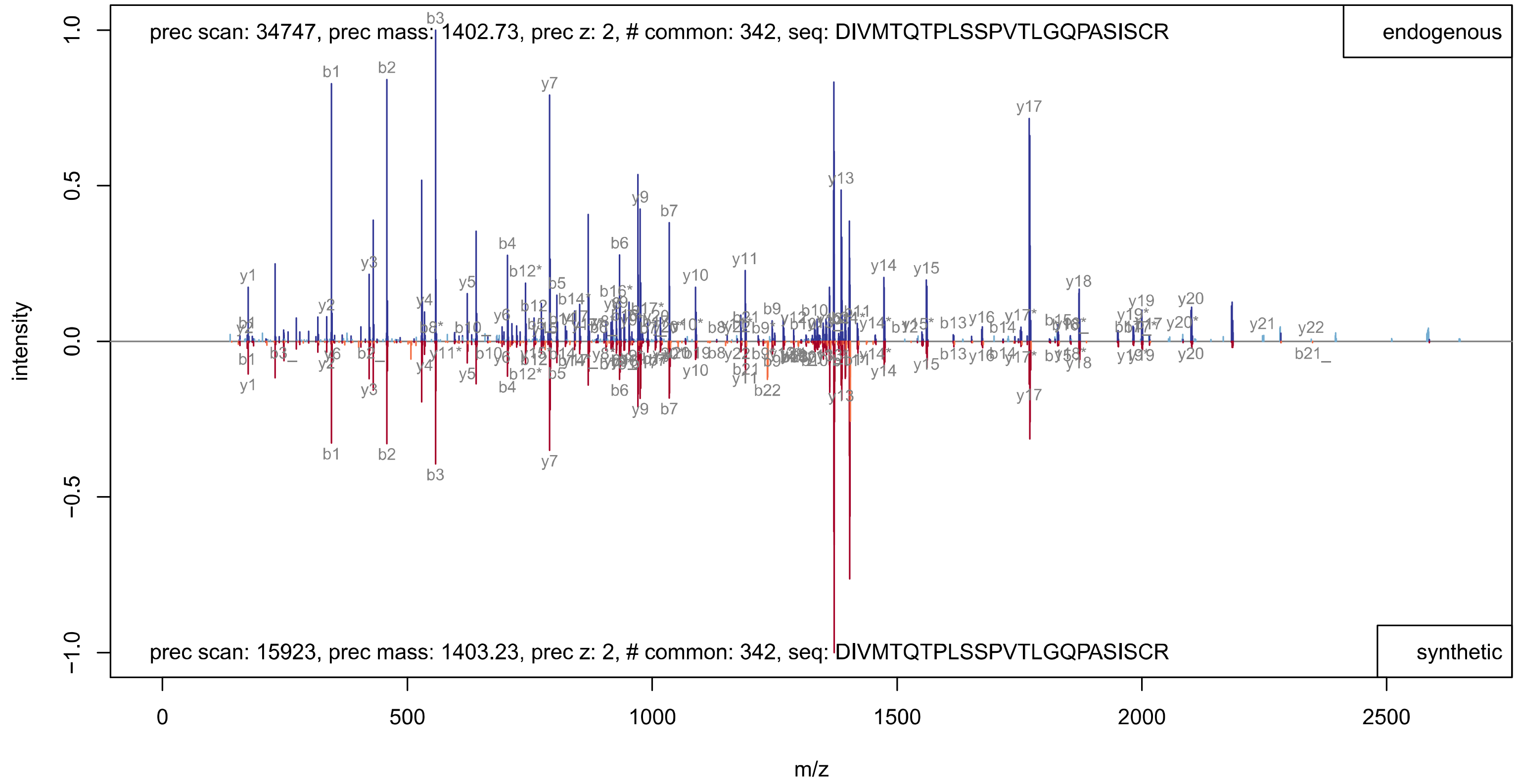

PGOHUM_ENST00000397642.3_RP11−488L18.1

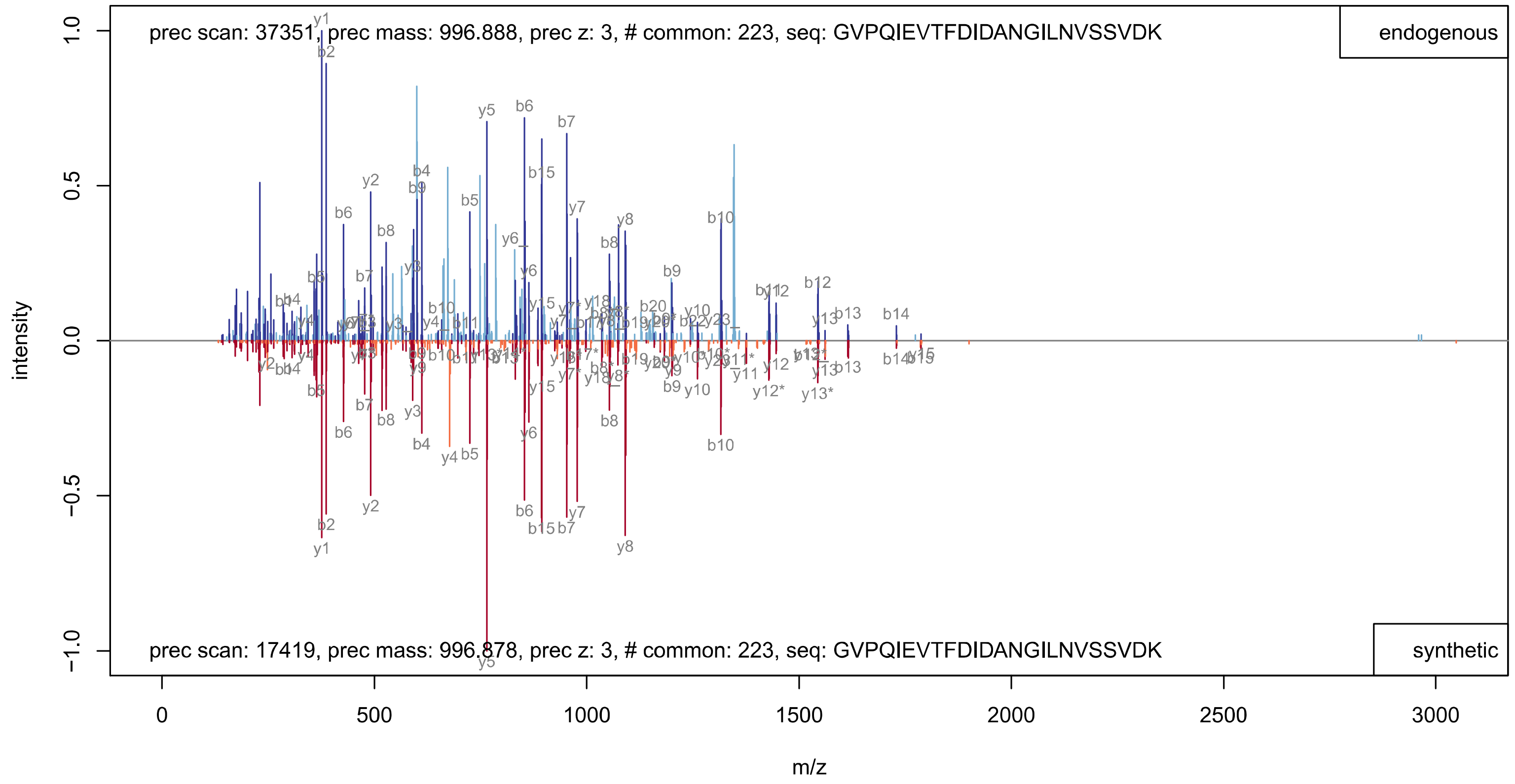

PGOHUM_ENST00000425843.1_HSPA8P1

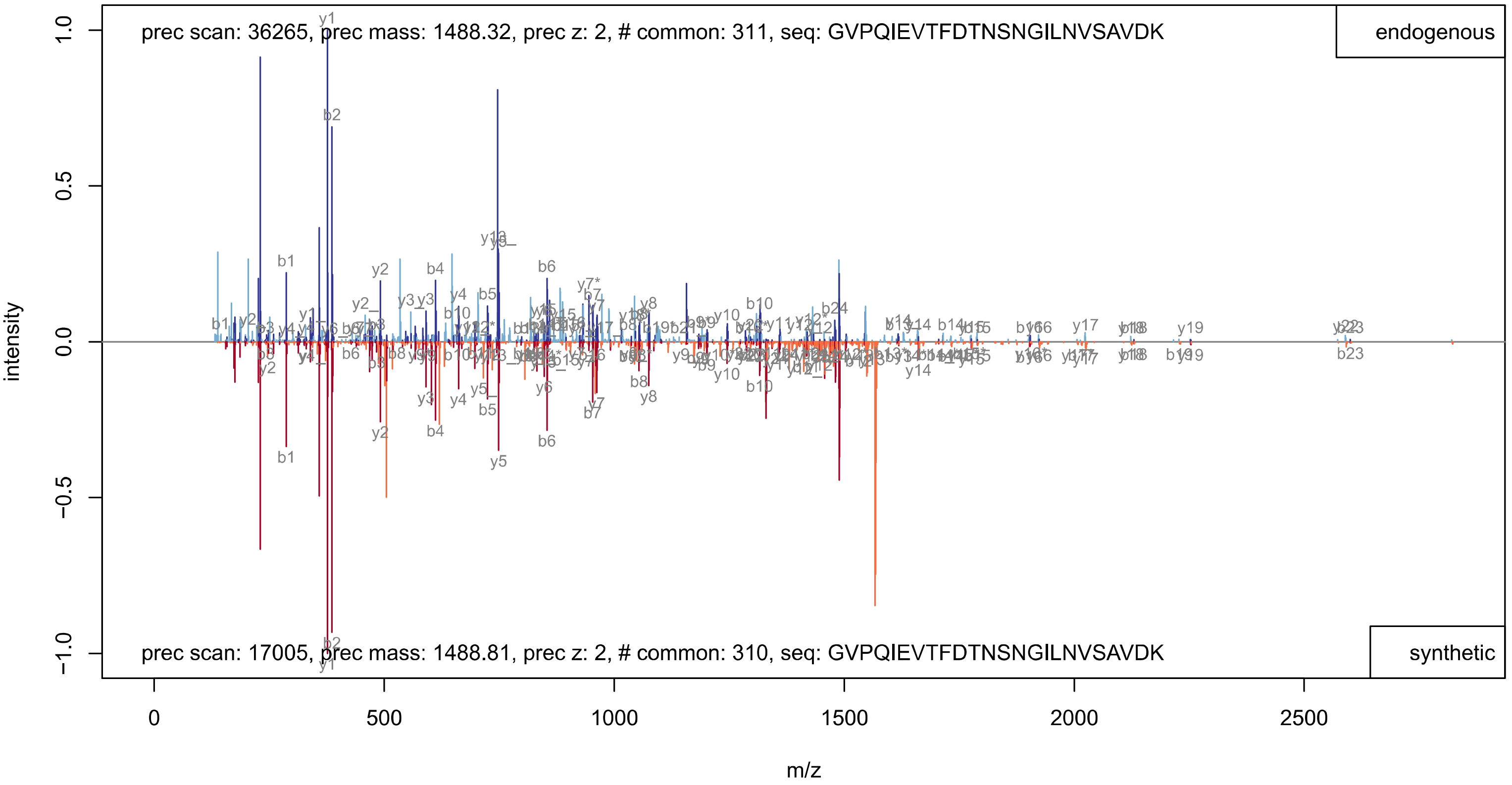

chr11_62494600

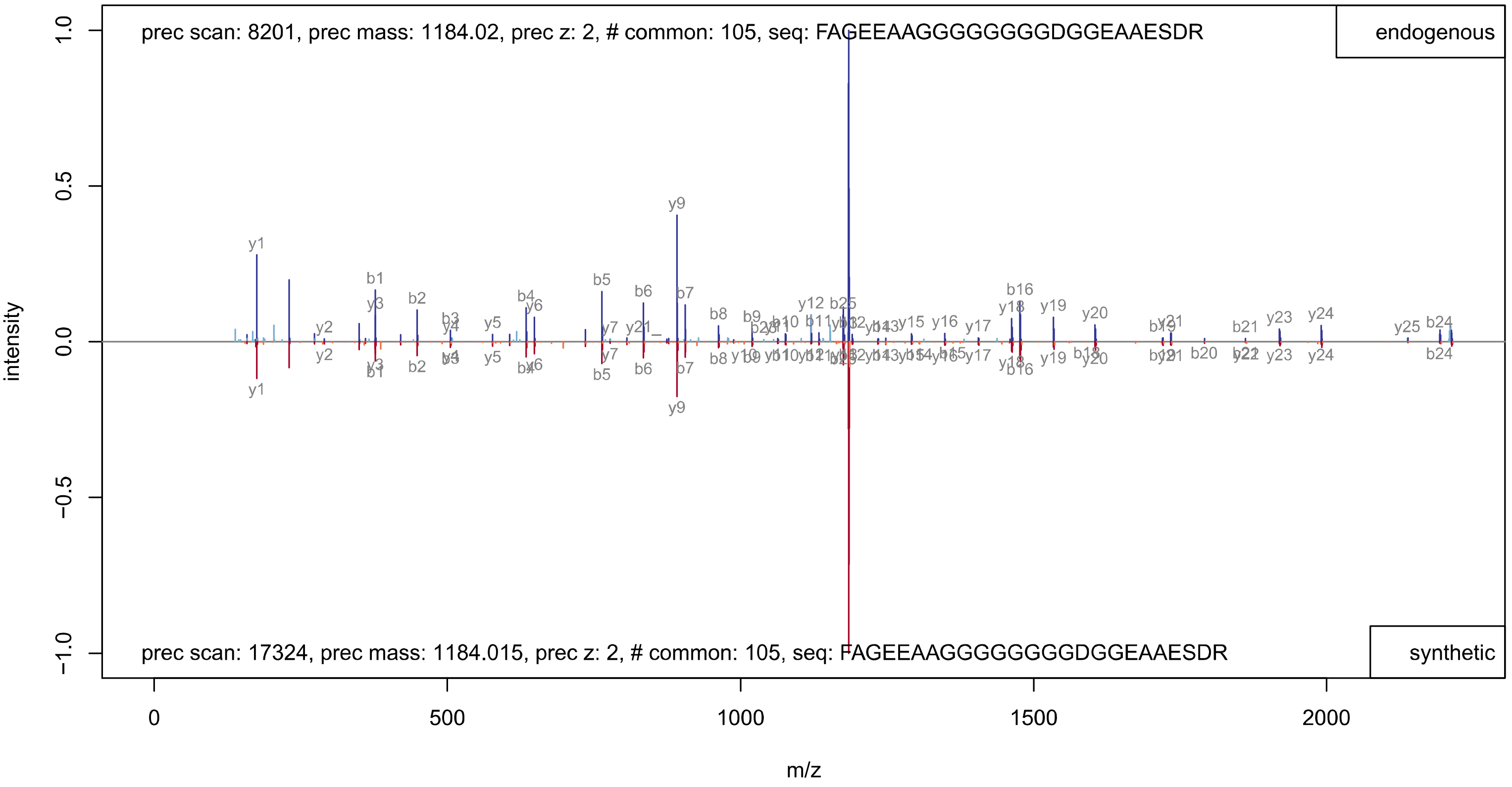

chr11_804093

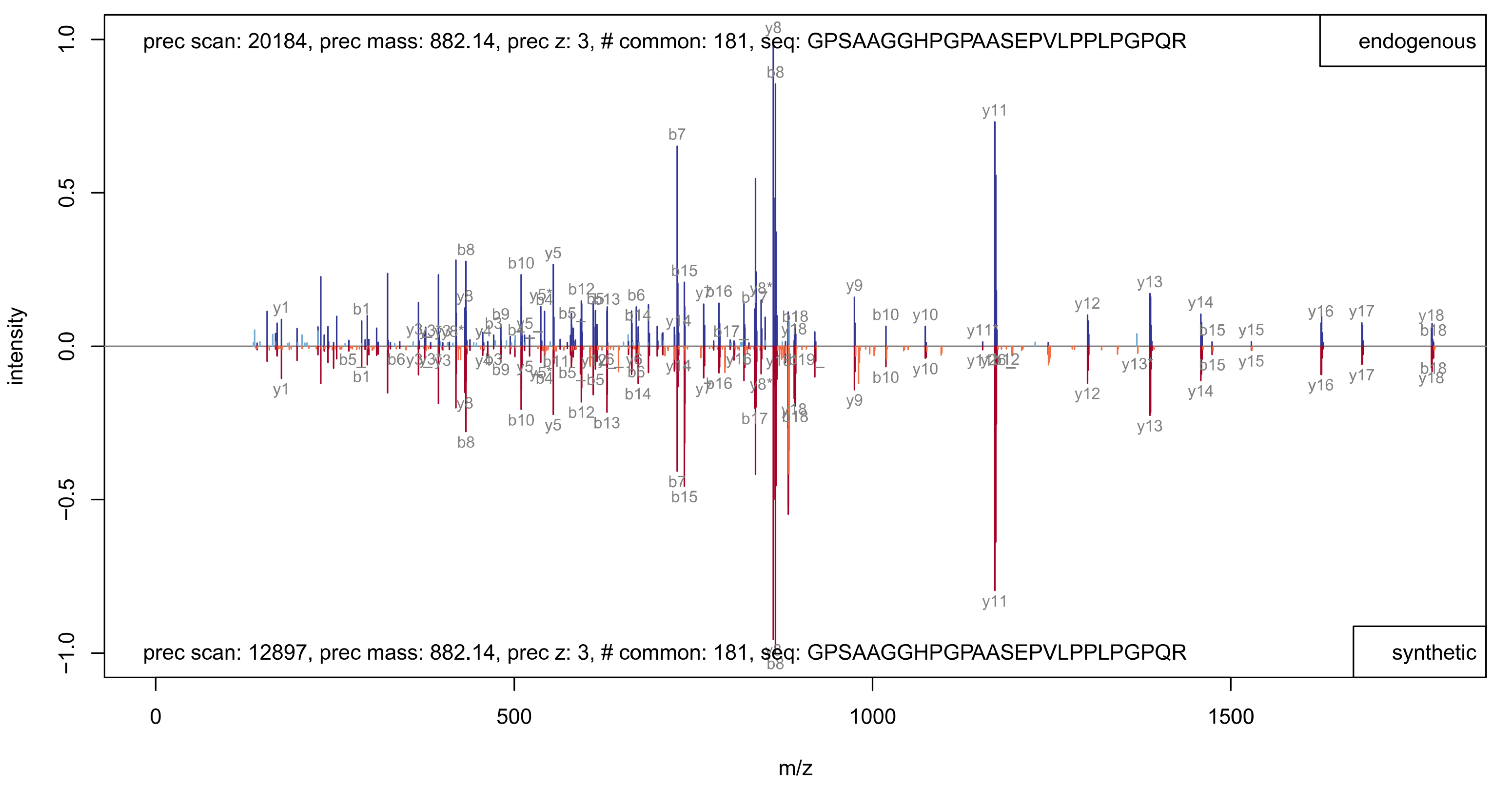

chr1_38273842

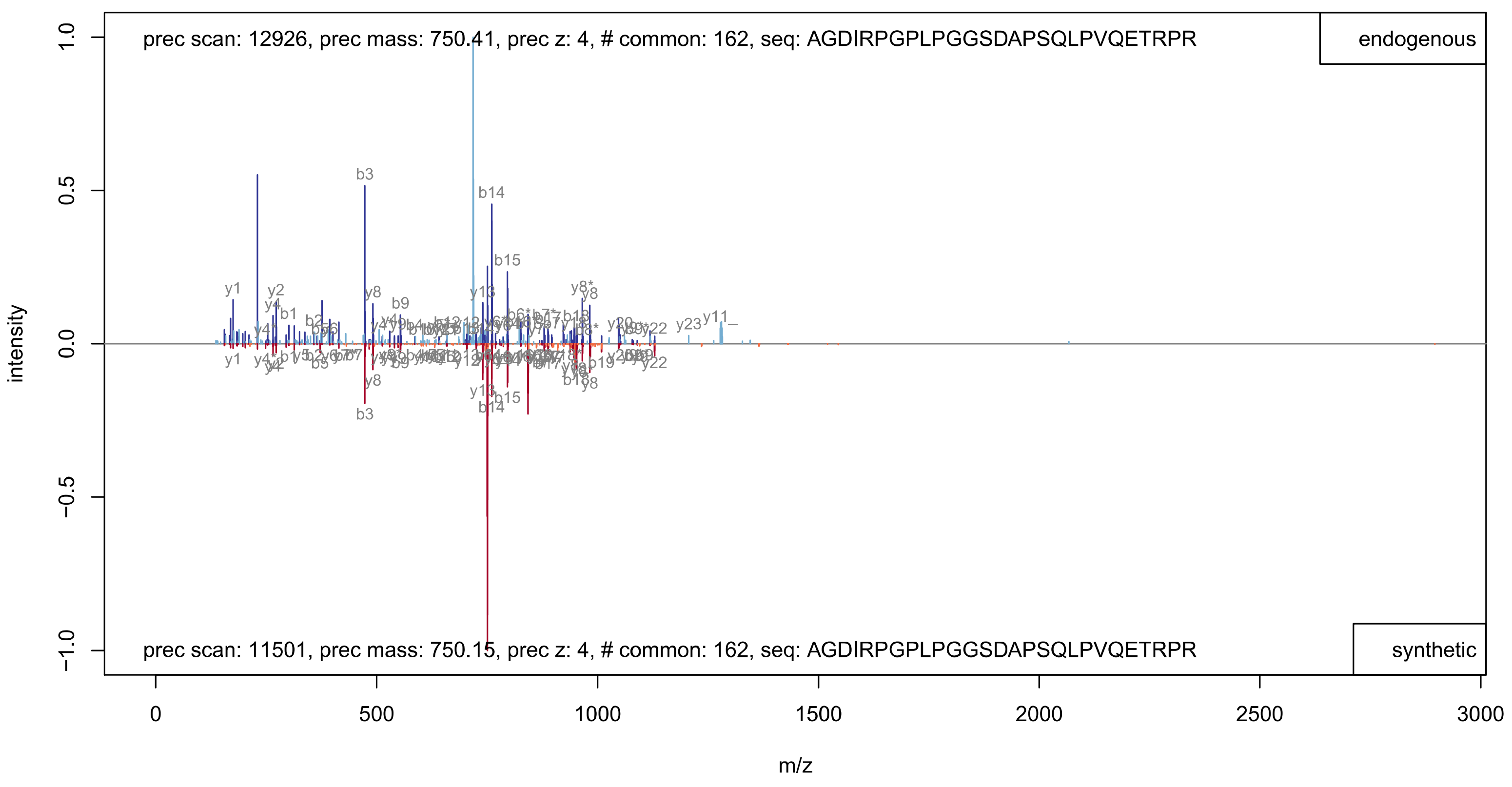

chr11_804171

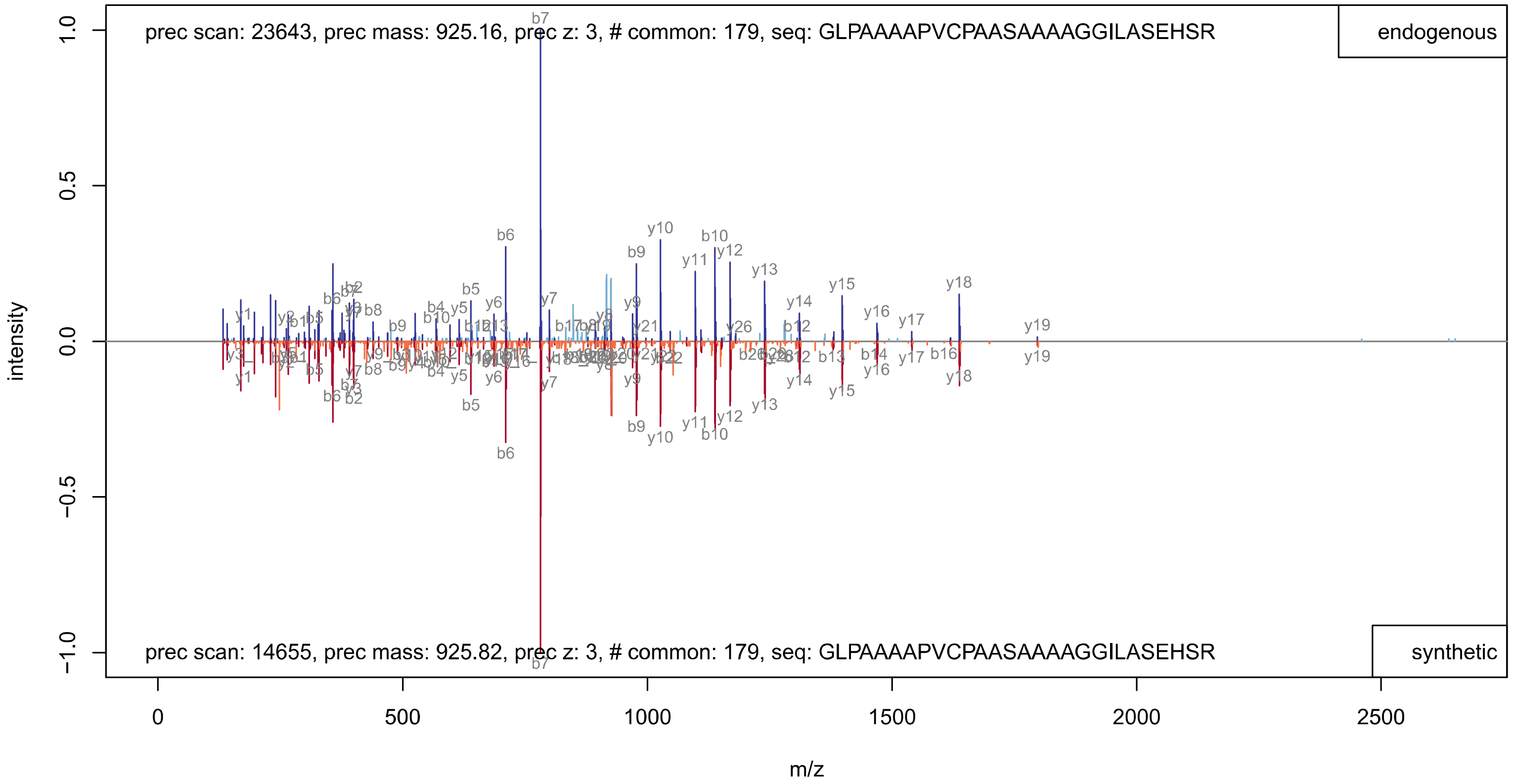

PGOHUM_ENST00000502424.1_RBBP4P1

incorrect match

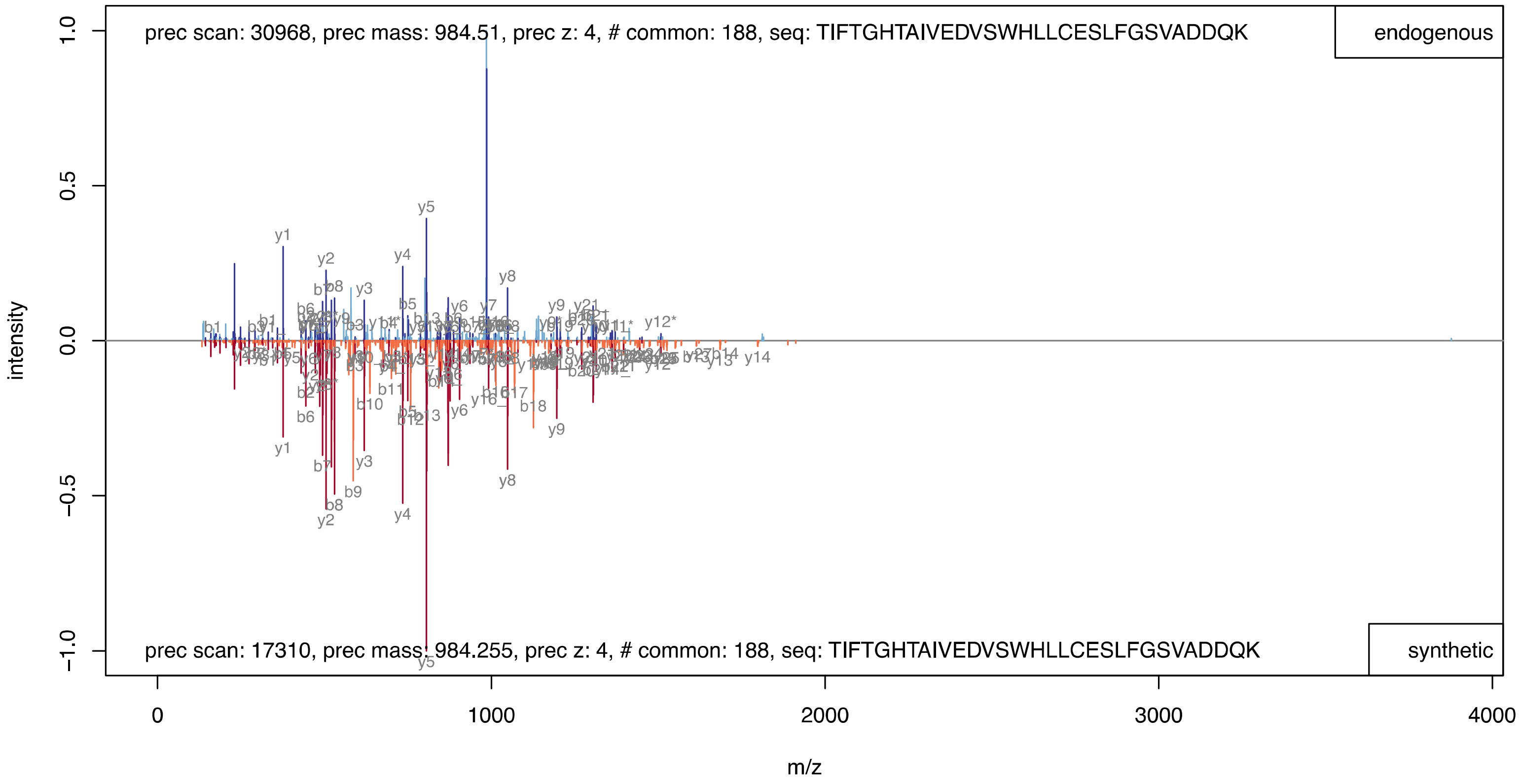

PGOHUM_ENST00000534819.1_AP003068.18

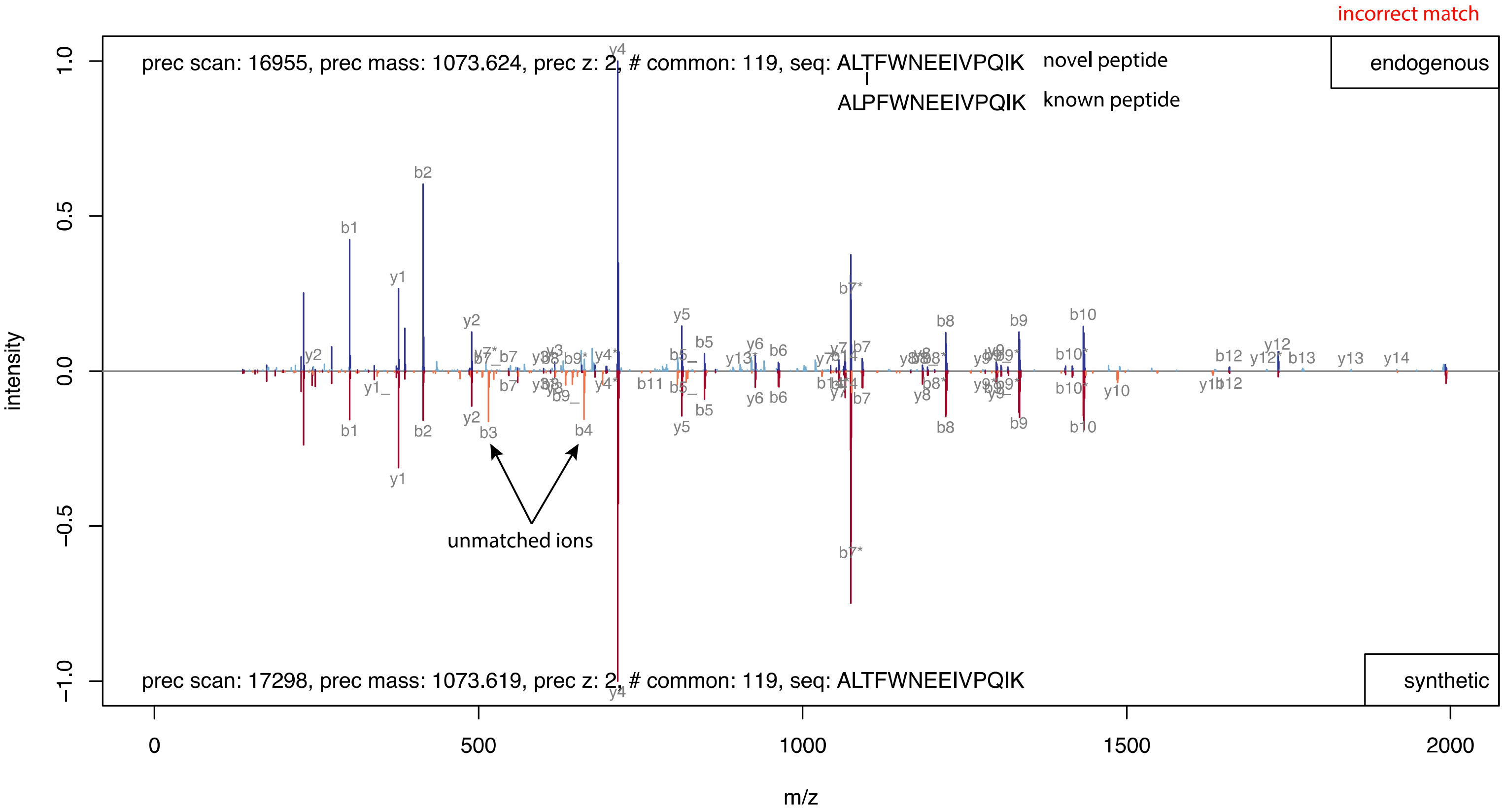

KRT14_V145L

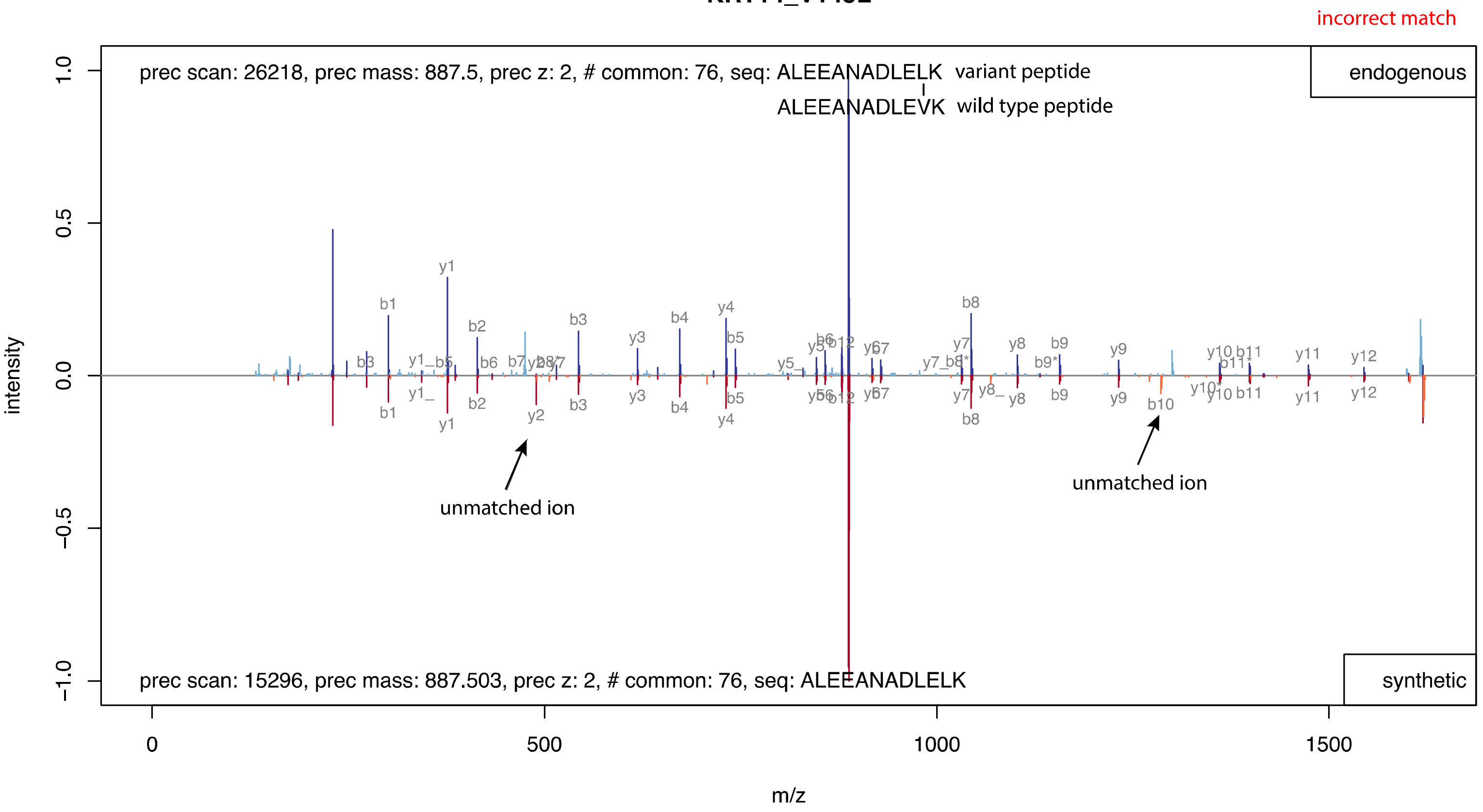

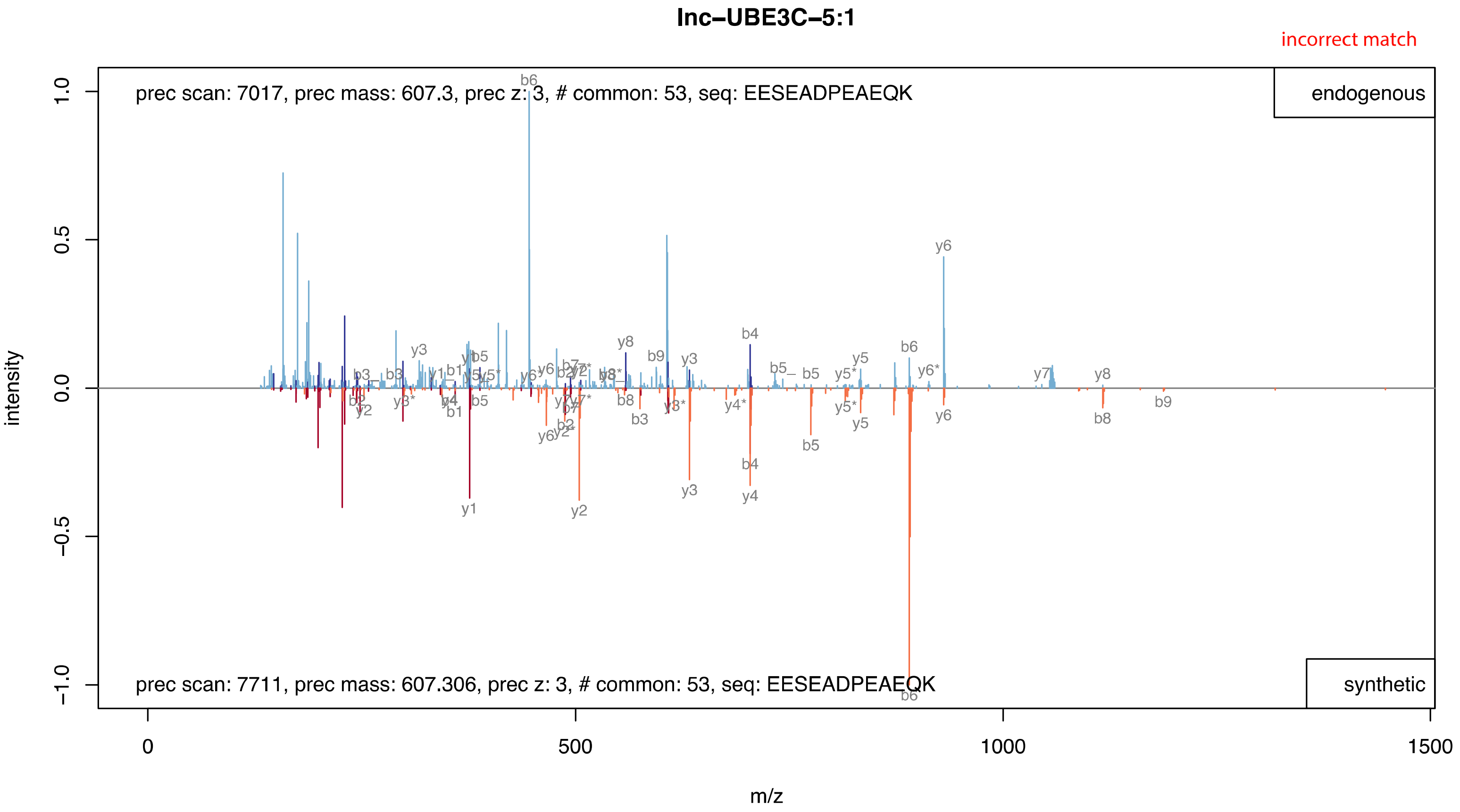
lnc−UBE3C−5:1
incorrect match
prec scan: 7017, prec mass: 607.3, prec z: 3, # common: 53, seq: EESEADPEAEQK
endogenous
prec scan: 7711, prec mass: 607.306, prec z: 3, # common: 53, seq: EESEADPEAEQK
synthetic
intensity
m/z

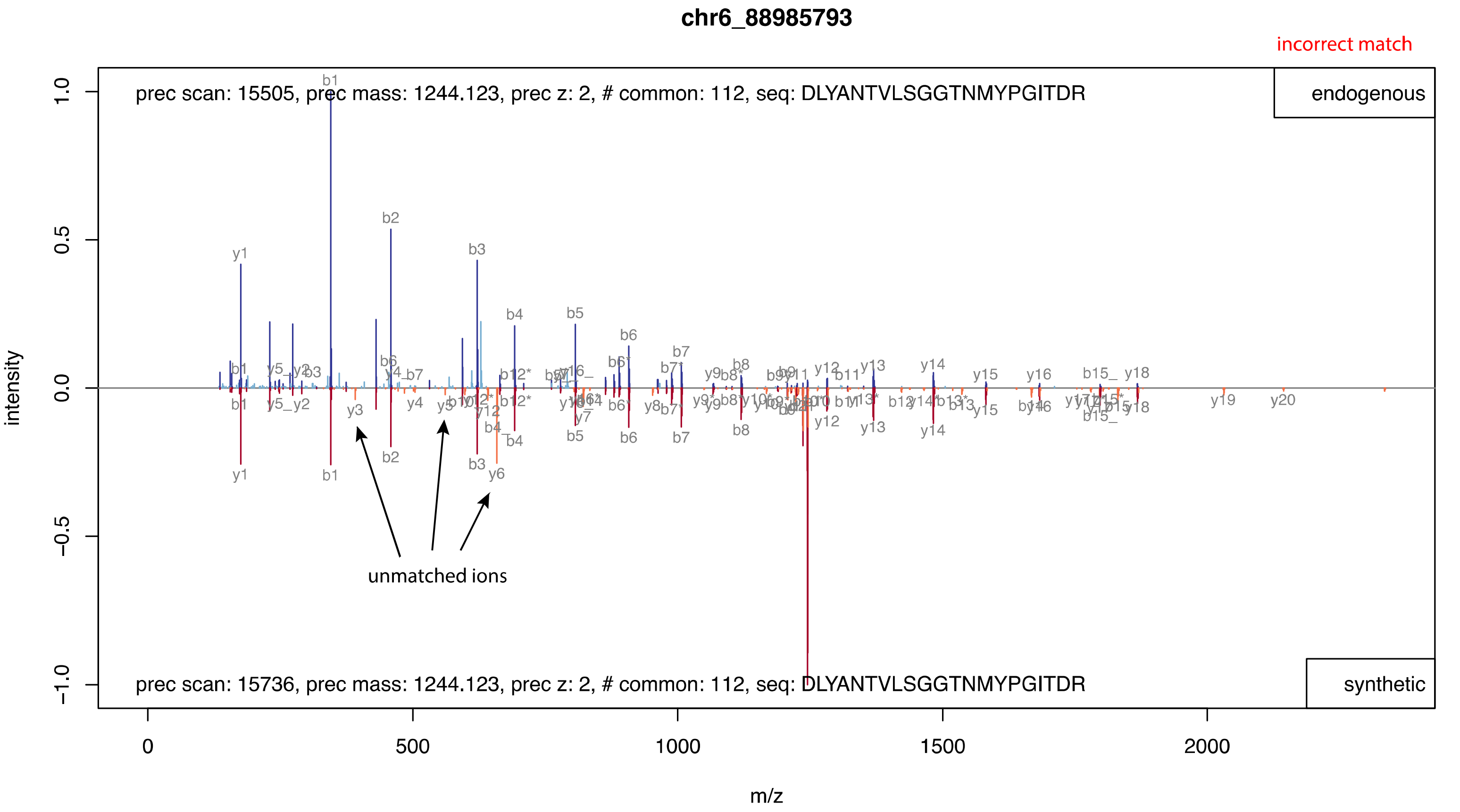
chr6_88985793
incorrect match
prec scan: 15505, prec mass: 1244.123, prec z: 2, # common: 112, seq: DLYANTVLSGGTNMYPGITDR
endogenous
prec scan: 15736, prec mass: 1244.123, prec z: 2, # common: 112, seq: DLYANTVLSGGTNMYPGITDR
synthetic
unmatched ions
intensity
m/z

PGOHUM_ENST00000511530.1_GAPDHP71

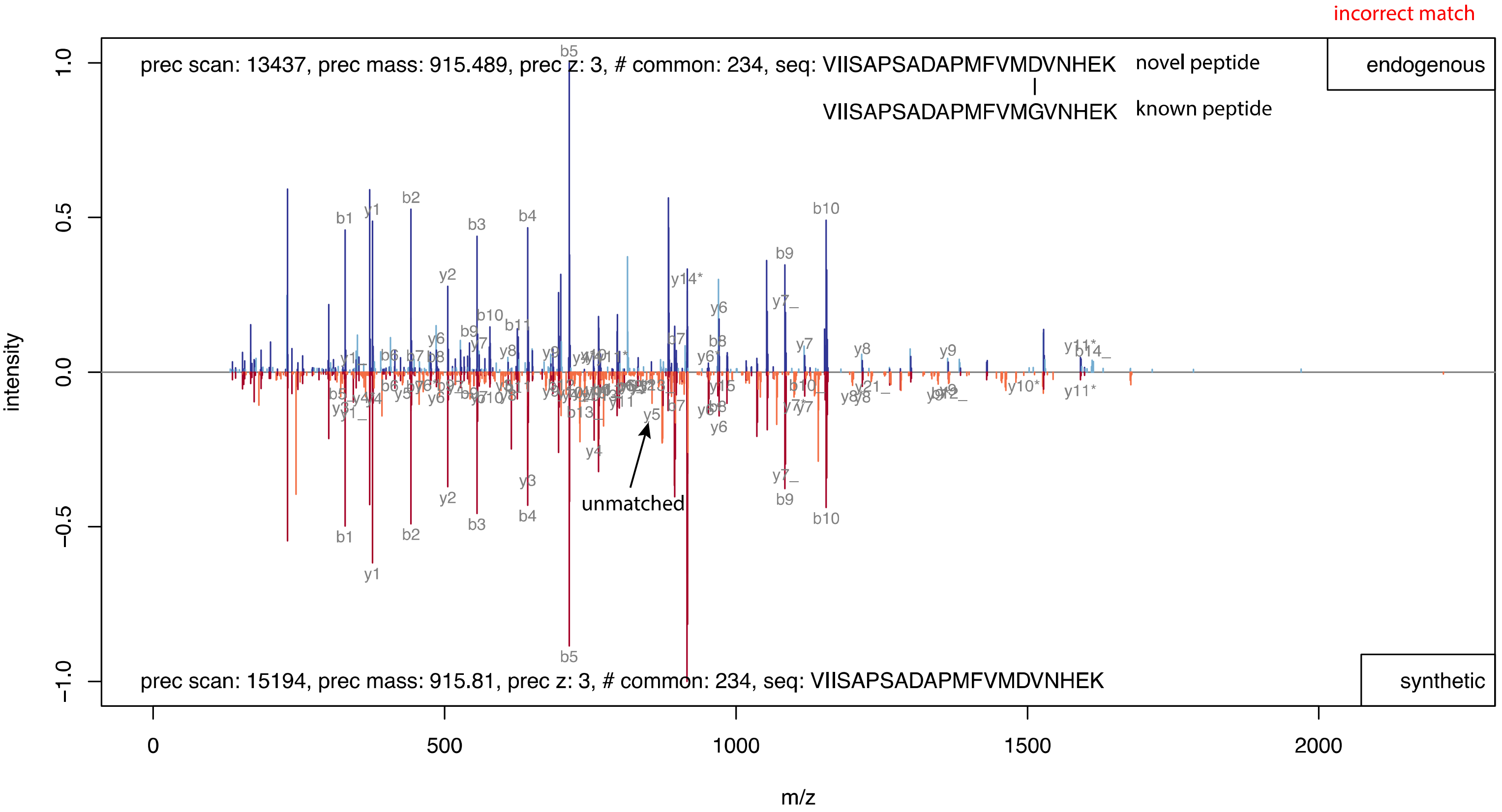

lncRNA_ENST00000340585.6

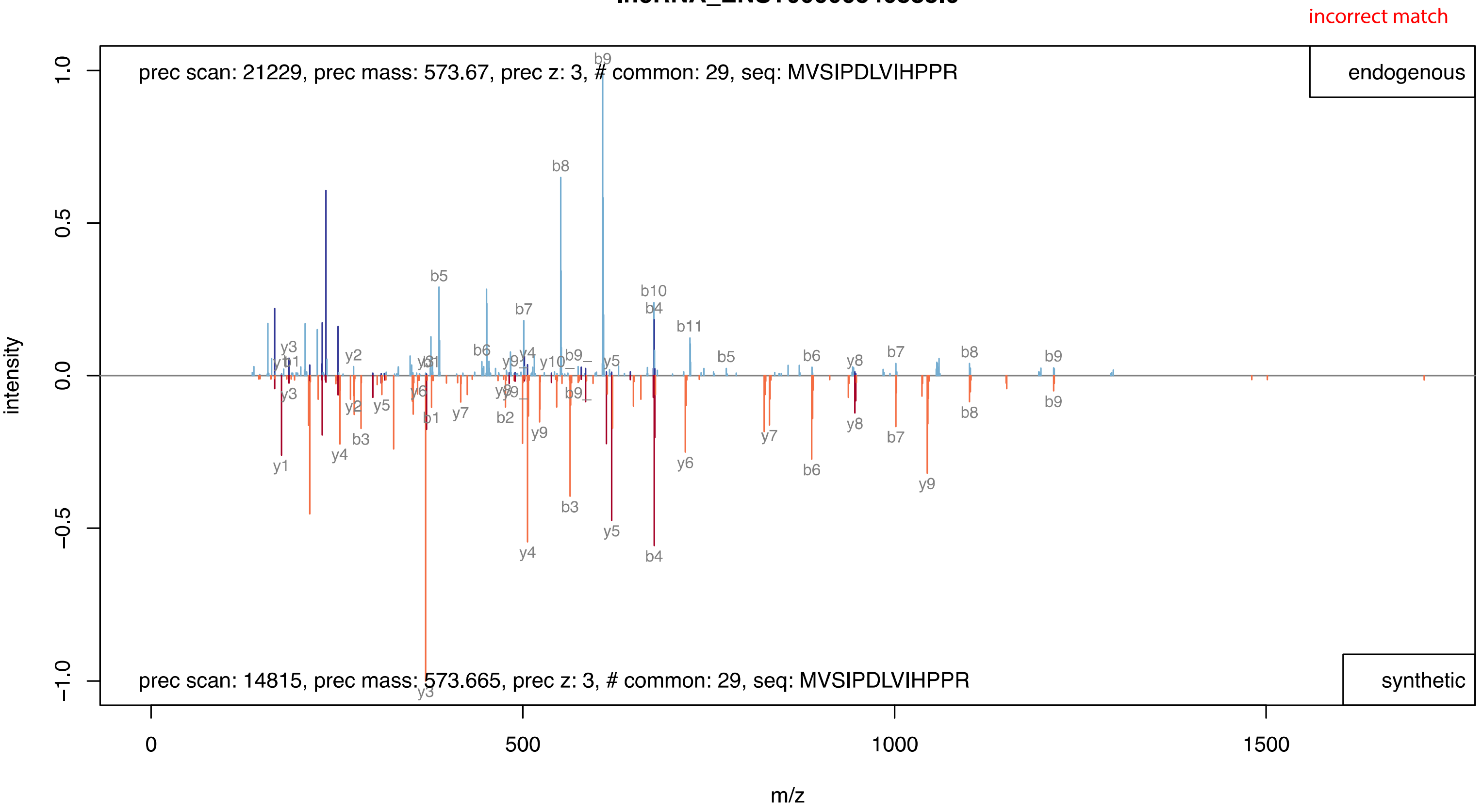

KRT17_A228T

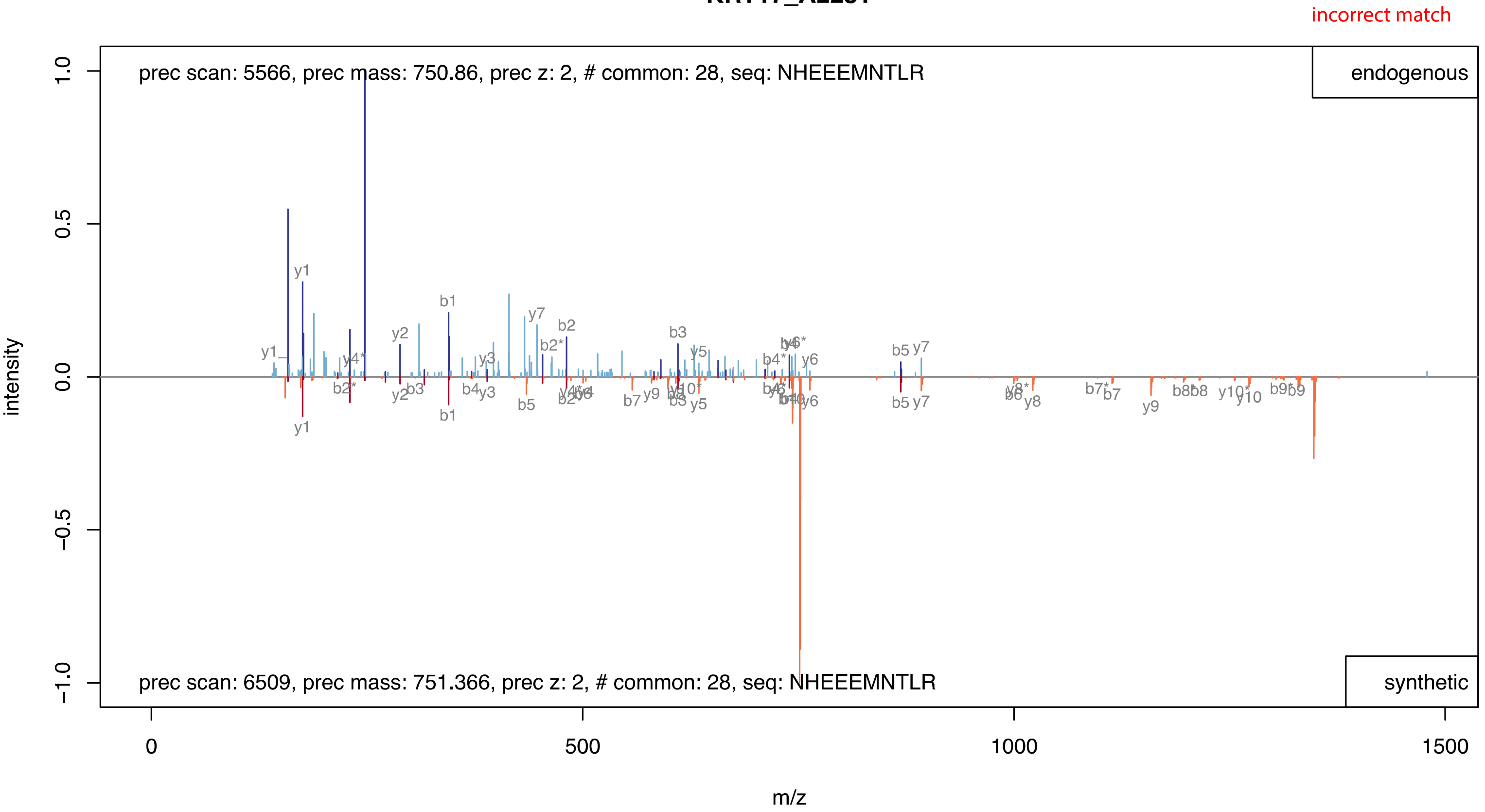

AKT2_Q429E

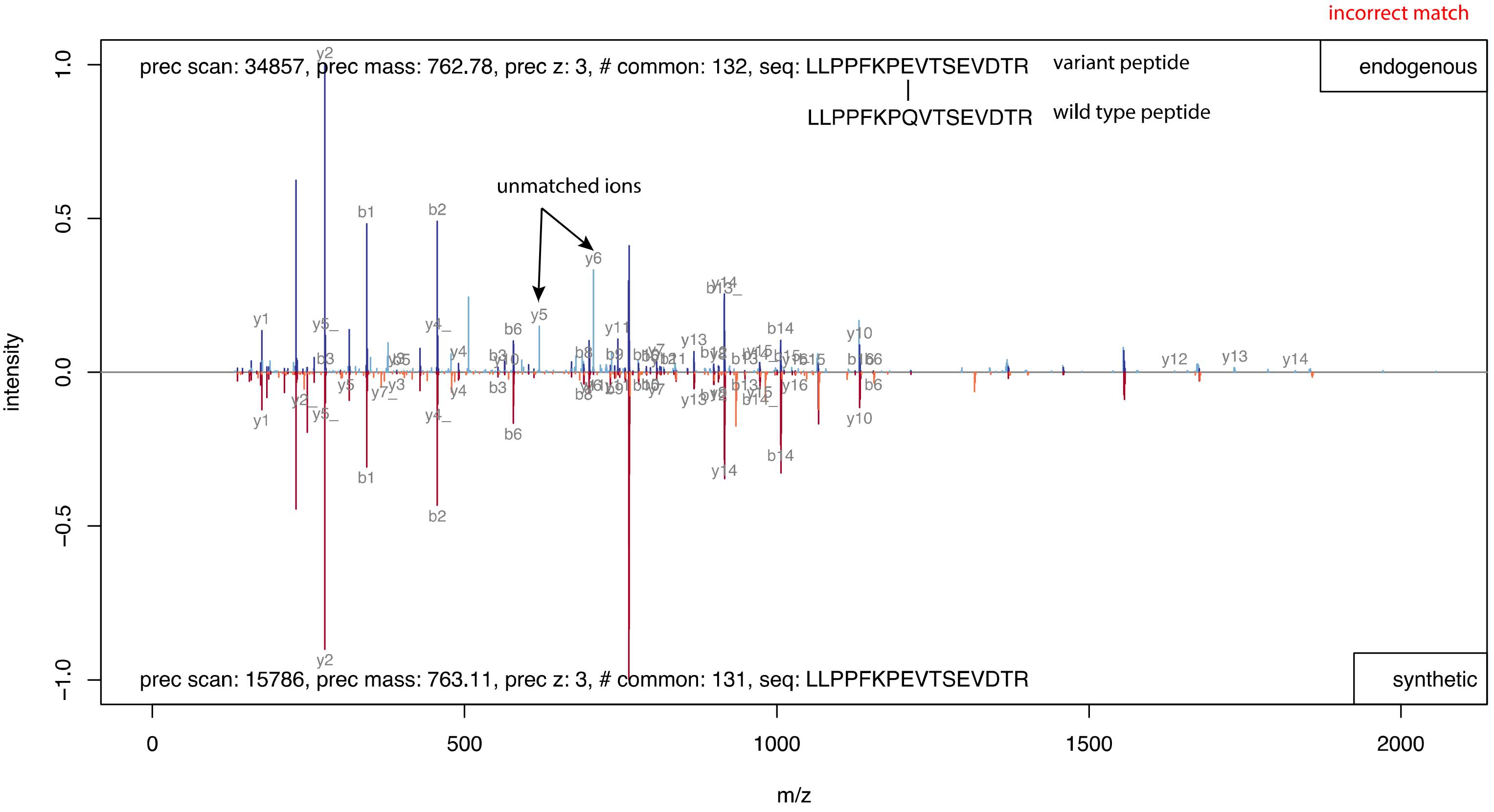

Supplement: Supplementary file 9 — Supplementary Data 7 [file 41467_2019_9018_MOESM9_ESM.pdf]
