# Supplementary material for: Breast cancer quantitative proteome and proteogenomic landscape
Source: Nat Commun. 2019 Apr 8;10:1600. doi: 10.1038/s41467-019-09018-y (PMC6453966; doi:10.1038/s41467-019-09018-y)
Supplement: Supplementary file 11 — Reporting Summary [file 41467_2019_9018_MOESM11_ESM.pdf]

nature research

Corresponding author(s): Janne Lehtiö

Last updated by author(s): Feb 5, 2019

Reporting Summary

Nature Research wishes to improve the reproducibility of the work that we publish. This form provides structure for consistency and transparency in reporting. For further information on Nature Research policies, see Authors & Referees and the Editorial Policy Checklist.

Statistics

For all statistical analyses, confirm that the following items are present in the figure legend, table legend, main text, or Methods section.

n/a	Confirmed	
☐	☒	The exact sample size (*n*) for each experimental group/condition, given as a discrete number and unit of measurement
☐	☒	A statement on whether measurements were taken from distinct samples or whether the same sample was measured repeatedly
☐	☒	The statistical test(s) used AND whether they are one- or two-sided<br>*Only common tests should be described solely by name; describe more complex techniques in the Methods section.*
☒	☐	A description of all covariates tested
☐	☒	A description of any assumptions or corrections, such as tests of normality and adjustment for multiple comparisons
☐	☒	A full description of the statistical parameters including central tendency (e.g. means) or other basic estimates (e.g. regression coefficient) AND variation (e.g. standard deviation) or associated estimates of uncertainty (e.g. confidence intervals)
☐	☒	For null hypothesis testing, the test statistic (e.g. *F*, *t*, *r*) with confidence intervals, effect sizes, degrees of freedom and *P* value noted<br>*Give P values as exact values whenever suitable.*
☒	☐	For Bayesian analysis, information on the choice of priors and Markov chain Monte Carlo settings
☒	☐	For hierarchical and complex designs, identification of the appropriate level for tests and full reporting of outcomes
☒	☐	Estimates of effect sizes (e.g. Cohen's *d*, Pearson's *r*), indicating how they were calculated

Our web collection on statistics for biologists contains articles on many of the points above.

Software and code

Policy information about availability of computer code

Data collection	Xcalibur version 4.0, msConvert, MSGF+ (v10072), Percolator (v2.08), OpenMS project's IsobaricAnalyzer (v2.0)
Data analysis	The list is also given in Supplementary table 2.<br>Gene-E<br>https://software.broadinstitute.org/GENE-E/<br>Morpheus<br>https://software.broadinstitute.org/morpheus/<br>Graphpad Prism 7.01<br>https://www.graphpad.com/scientific-software/prism/<br>Association of correlation to protein interactions<br>https://github.com/aleferna/BCLandscape<br>Script: PearsonVsBioGrid.R script<br>Correlation network<br>https://github.com/aleferna/BCLandscape<br>Script: Generate_Clustering_and_Network.R & gephi.script<br>Consensus clustering<br>https://github.com/aleferna/BCLandscape<br>Script: Generate_Clustering_and_Network.R<br>Shiny Web Portal Tools<br>https://github.com/aleferna/BCLandscape_Shiny<br>GSEA<br>http://software.broadinstitute.org/gsea/index.jsp<br>mRNA-protein correlation<br>https://doi.org/10.5281/zenodo.1466478<br>CNA-mRNA/protein

https://github.com/ioasia/Breast-Cancer
Gephi
https://gephi.org/
Proteogenomics analysis
https://github.com/yafeng/proteogenomics_python
SpectrumAI
https://github.com/yafeng/SpectrumAI
Metafer v3.12.7
https://metasystems-international.com/en/products/metafer/
VSlide v1.1.09
https://metasystems-international.com/en/products/solutions/tissue-imaging/
VSViewer
https://metasystems-international.com/en/products/solutions/tissue-imaging/

For manuscripts utilizing custom algorithms or software that are central to the research but not yet described in published literature, software must be made available to editors/reviewers. We strongly encourage code deposition in a community repository (e.g. GitHub). See the Nature Research guidelines for submitting code & software for further information.

Data

Policy information about availability of data

All manuscripts must include a data availability statement. This statement should provide the following information, where applicable:

- Accession codes, unique identifiers, or web links for publicly available datasets
- A list of figures that have associated raw data
- A description of any restrictions on data availability

Data availability is stated in the paper and summarized in supplementary table 3. All generated MS data are publicly available from ProteomeXchange with identifiers PXD008841 and PXD011385. The sources of the other datasets used in the paper are indicated in supplementary table 3.

Field-specific reporting

Please select the one below that is the best fit for your research. If you are not sure, read the appropriate sections before making your selection.

☒ Life sciences ☐ Behavioural & social sciences ☐ Ecological, evolutionary & environmental sciences

For a reference copy of the document with all sections, see nature.com/documents/nr-reporting-summary-flat.pdf

Life sciences study design

All studies must disclose on these points even when the disclosure is negative.

Sample size	The tumor samples were chosen to include 9 tumors from each of the 5 PAM50 subtypes in breast cancer to account for between and within variation. Tumor numbers were limited due to technical limitations at the onset of the project.
Data exclusions	No data was not excluded. Data was filtered in various analysis to address certain questions.
Replication	We have also validated our findings using orthogonal methods and other levels of data in independent tumor cohorts. However, there is only one additional comprehensive breast tumor proteome study so far, which limits how the proteome data can be validated for reproducibility.
Randomization	The tumor samples were chosen to include 9 tumors from each of the 5 PAM50 subtypes in breast cancer. This was done to investigate the potential proteome differences between these subtypes. When the subtypes could be confounding factors they have been accounted for.
Blinding	Clinical information and PAM50 assignments were available, but the MS data were acquired in an unbiased way without knowledge of the tumor properties.

Reporting for specific materials, systems and methods

We require information from authors about some types of materials, experimental systems and methods used in many studies. Here, indicate whether each material, system or method listed is relevant to your study. If you are not sure if a list item applies to your research, read the appropriate section before selecting a response.

Materials & experimental systems

n/a	Involved in the study
☐	☒ Antibodies
☒	☐ Eukaryotic cell lines
☒	☐ Palaeontology
☒	☐ Animals and other organisms
☒	☐ Human research participants
☒	☐ Clinical data

Methods

n/a	Involved in the study
☒	☐ ChIP-seq
☒	☐ Flow cytometry
☒	☐ MRI-based neuroimaging

Antibodies

Antibodies used

Antibodies used are described in Supplementary table 1.

Validation

All antibodies were initially validated on cell lines to replicate known (via MS) high and low protein levels. Secondly, antibodies were tested on normal and tumor material and compared to expected staining patterns in literature.
